# Time-Zero Kidney Biopsy and Deceased-Donor Transplant Outcomes: A Systematic Review and Meta-Analysis of Prognostic Value, Prediction, and Clinical Utility

**DOI:** 10.3390/medsci14050566

**Published:** 2026-09-12

**Authors:** Livier Sainz-Bravo, Benjamín Gómez-Navarro, Diana Laura Figueroa-Gamiño, Jenniffer Topacio Tapia-Báez, José David González-Barajas, Manuel Luis Prieto-Magallanes, José Ángel Solís-Rojas, Edgar Bautista-Reyes, Juan Alberto Gómez-Fregoso, Judith Carolina De Arcos-Jiménez, Ramón Medina-González, Violeta Aidee Camarena-Arteaga, Manuel Arizaga-Napoles, Jaime Briseno-Ramirez

**Affiliations:** 1 Hospital Civil de Oriente, Tonalá 45425, Mexico; lsainz@hcg.gob.mx (L.S.-B.); jdgonzalez@hcg.gob.mx (J.D.G.-B.); mlprieto@hcg.gob.mx (M.L.P.-M.); judith.dearcos@academicos.udg.mx (J.C.D.A.-J.); 2025009@correo.opdhcg.net (V.A.C.-A.); 2 Consejo Estatal de Trasplantes de Jalisco (CETRAJAL), Guadalajara 45177, Mexico; gonfre@gmail.com; 3 Centro Médico Puerta de Hierro Norte, Zapopan 45116, Mexico; 4 Hospital Juárez de México, Mexico City 07760, Mexico; aprptdiana@gmail.com; 5 Hospital General de México “Dr. Eduardo Liceaga”, Mexico City 06720, Mexico; tapiabaez07@gmail.com; 6 Centro Universitario de Ciencias de la Salud, Universidad de Guadalajara, Guadalajara 44340, Mexico; drjuangomezf@gmail.com; 7 Centro Universitario de Tlajomulco, Universidad de Guadalajara, Tlajomulco 45641, Mexico; ramon.medinagonzalez@academicos.udg.mx; 8 Hospital Regional de Alta Especialidad del Instituto de Seguridad y Servicios Sociales de los Trabajadores del Estado (ISSSTE), Acapulco 39850, Mexico; joseangel19100@gmail.com; 9 Centro Médico Nacional 20 de Noviembre, Instituto de Seguridad y Servicios Sociales de los Trabajadores del Estado (ISSSTE), Mexico City 03229, Mexico; edgbr.3@gmail.com; 10 Antiguo Hospital Civil de Guadalajara “Fray Antonio Alcalde”, Guadalajara 44280, Mexico; manarizaganapoles@gmail.com

**Keywords:** kidney transplantation, deceased donor, procurement biopsy, delayed graft function, prognosis, decision utility, meta-analysis

## Abstract

**Background/Objectives:** Time-zero biopsy informs deceased-donor kidney implantation or discard, but its prognostic value is contested. We systematically reviewed pre-reperfusion morphological biopsy evidence in adult deceased-donor kidney transplantation, keeping four questions separate: prognostic association, incremental prediction, clinical utility and measurement reproducibility. **Methods:** We searched PubMed/MEDLINE, Embase, Scopus, Web of Science, CENTRAL and three trial registries through 25 July 2026 without date or language restriction. One estimate per non-overlapping cohort was selected under a prespecified hierarchy; random-effects models used restricted maximum likelihood with Hartung–Knapp inference; GRADE assessed certainty. **Results:** Of 45,676 records, 25,746 were screened; 203 eligible reports (159 study investigations and 154 cohort families) and 22 contextual reports were included. Vascular injury was associated with delayed graft function (odds ratio: 1.85, 95% CI 1.11–3.06), glomerulosclerosis with graft failure (hazard ratio: 2.03, 1.22–3.39) and vascular injury with graft failure (hazard ratio: 1.48, 1.07–2.04); glomerulosclerosis with delayed graft function was inconclusive (odds ratio: 1.13, 0.59–2.17). All 12 pooled bodies were of very low certainty. **Conclusions:** Current evidence does not support time-zero histology as a stand-alone rule for accepting or discarding a deceased-donor kidney. Whether standardized histology adds predictive information beyond established clinical variables remains open and should be addressed in linked registry analyses before biopsy findings guide management.

## 1. Introduction

Kidney transplantation offers longer survival and better quality of life than maintenance dialysis for most patients with kidney failure [1,2]. Demand nonetheless exceeds the deceased-donor supply, and many candidates die or are removed from the waiting list before a suitable organ becomes available [3]. To narrow that gap, transplant programs have progressively accepted more marginal organs—from expanded-criteria donors (ECDs), from donation after circulatory death (DCD), and from donors whose quality is expressed on continuous scales such as the Kidney Donor Risk Index and the Kidney Donor Profile Index (KDPI) [4,5]. Yet a substantial proportion of recovered kidneys is still discarded, acceptance thresholds vary widely between health systems, and there is evidence that organs which would have functioned are being declined [6,7,8]. Deciding which recovered kidney to implant and which to discard is therefore among the most frequent and most consequential judgments in transplantation, and the instrument used to make it deserves formal prognostic scrutiny.

In most programs, that instrument is the time-zero biopsy—the procurement, back-table or preimplantation specimen obtained before reperfusion—and its reading is often the proximate determinant of whether an organ is used [9]. Its findings are expressed either as individual lesions, chiefly the percentage of glomerulosclerosis, interstitial fibrosis and tubular atrophy (IFTA), acute tubular injury and vascular or arteriolar lesions, or as composite scores that aggregate several compartments, including the Remuzzi and Karpinski/Maryland Aggregate Pathology Index scores [10,11]. The conditions under which these readings are produced are unfavorable to precision: specimens are frequently frozen wedge sections, interpreted under time pressure, often by pathologists who are not renal subspecialists, and agreement between observers and between processing methods is poor [12,13]. Sampling error and processing artifact may therefore alter both the lesion that is measured and the decision that follows it. Despite these limitations, isolated glomerulosclerosis thresholds remain a dominant trigger for discard [14].

Whether time-zero histology actually forecasts graft outcome remains unresolved. The most influential previous systematic review synthesized the evidence narratively and found no consistent association between biopsy findings and graft outcomes; because the primary studies reported too heterogeneously to combine, it neither pooled the data nor reported a summary heterogeneity statistic, and it preceded current reporting standards and appraisal methods designed for prognosis research [15,16]. Several problems have persisted since. Single lesions and composite scores are seldom disentangled. Crude and confounder-adjusted estimates are routinely combined despite strong potential for confounding [17]. Overlapping registry and multicenter cohorts risk being counted more than once. Biopsy technique and tissue processing are rarely taken into account. Appraisal frameworks built for prognostic factors and for prediction models are seldom applied [18]. The literature on discard is affected by circularity, because in many series the biopsy was itself the rule that declined the organ. Evidence specific to ECD and DCD donors is sparse.

Four questions must also be kept apart, because they are commonly conflated and they carry different clinical consequences. A lesion may be associated with an outcome without improving prediction beyond routinely available clinical information; a model may predict without any evidence that acting on it benefits patients; and any rule built on a measurement requires that the measurement be reproducible. Donor age, donor function and KDPI are known before any biopsy is read, and age-matched allocation programs already structure practice, so any proposed role for histology should be judged by what it adds beyond these variables and the allocation context rather than by association with outcome alone. We therefore conducted a systematic review and meta-analysis of pre-reperfusion morphological biopsy evidence in adult deceased-donor kidney transplantation, addressing prognostic association, incremental predictive value beyond clinical variables, the clinical utility of biopsy-guided decisions, and the reproducibility of the measurement itself, and keeping the four bodies of evidence conceptually and analytically separate (PROSPERO CRD420261435405). To our knowledge, this is the first review in this field to pool lesion–outcome associations quantitatively while controlling cohort overlap, and the first to keep association, incremental prediction, clinical utility and measurement reproducibility analytically separate. The review was reported in accordance with PRISMA 2020 and PRISMA-S [19,20], used SWiM where meta-analysis was inappropriate [21], followed prognosis-review guidance [16], and rated certainty with GRADE adapted for prognostic factors [22].

## 2. Materials and Methods

### 2.1. Protocol and Registration

The review was registered in PROSPERO on 27 June 2026 (CRD420261435405; registration version 2.0 published 23 July 2026), before execution of the final search (25 July 2026) and before screening of the final record corpus, extraction and all analyses (analysis freeze 3 August 2026); an earlier registration (CRD420261361754, 28 April 2026) was superseded by the current record. The review is reported in accordance with PRISMA 2020 and, for the search, the PRISMA-S extension [19,20]. Synthesis of results that could not be meta-analyzed follows the Synthesis Without Meta-analysis guideline [21]; appraisal and certainty were framed for prognosis research [16,17], prediction evidence additionally against TRIPOD for systematic reviews [23], and the review audited itself against AMSTAR for prognostic factors [24]. Completed PRISMA 2020, PRISMA 2020 for Abstracts, PRISMA-S, SWiM and TRIPOD-SRMA checklists, together with the AMSTAR-PF self-audit, are appended to the Supplementary Material.

### 2.2. Eligibility Criteria

We included studies of adult recipients of deceased-donor kidney transplants and studies of kidneys recovered from deceased donors for transplantation. Cohorts that combined living and deceased donors, or kidney and other organ transplants, were eligible only when kidney-specific, deceased-donor data could be separated.

The review addressed four questions that are frequently conflated and were kept separate throughout. For the question of prognostic association, a study had to report morphological histology, or a histological score, from a procurement, back-table or preimplantation biopsy taken before reperfusion, together with a post-transplant outcome; evidence from post-reperfusion biopsies, or of mixed timing that could not be separated, was treated as contextual rather than analytic. For incremental predictive value, a study had to develop, validate or evaluate the added value of a prediction model. For clinical utility, it had to compare strategies for obtaining, processing, communicating or acting on biopsy information. For measurement, it had to address specimen adequacy, reproducibility, technique, processing, agreement between pre- and post-reperfusion readings, or computational pathology derived from morphology.

Routine morphology, composite scores and morphology-derived computational pathology were eligible exposures. Blood, urine and molecular-only tissue biomarkers were excluded from the core review. We excluded reports restricted to children or to living donors, non-kidney studies, non-original work and conference-only material, although a linked complete peer-reviewed report remained eligible. Descriptive studies required at least 20 kidneys or recipients. Neither language, publication date, a missing abstract, nor an unavailable full text was in itself treated as a scientific reason for exclusion.

### 2.3. Information Sources and Search

We searched PubMed/MEDLINE, Embase.com, Scopus, the Web of Science Core Collection and CENTRAL from inception to 25 July 2026, without restriction by date or language; CENTRAL was limited to its Trials source set. ClinicalTrials.gov, ISRCTN and the WHO ICTRP supplemented the bibliographic databases. Three purpose-built search streams addressed prognostic morphology, prediction, and the availability or decision impact of biopsy. Before final execution, an external reviewer independent of strategy development and reporting five years of search-review experience or training assessed the primary PubMed strategies against all six PRESS 2015 domains [25]. The reviewer also examined database translations and registry logic. Required revisions were implemented in version 2.4.2 and reconfirmed before the final searches; the complete strategies, source execution and review-response record are reported in Supplementary Table S1a–c.

The protocol prescribed backward and forward citation searching. Citation-derived evidence of identity was used during report-family reconciliation and targeted retrieval.

### 2.4. Study Selection and Data Collection

Records were grouped into families of related reports before screening. After deduplication, 25,746 records entered screening: prespecified deterministic title-and-abstract rules routed 23,469 records, while the residual 2277 records, grouped into 2212 work units, required semantic assessment. A score-based decision cascade was applied to all 25,746 screened records. Screening also included rapid review of proposed exclusions and targeted adjudication of ambiguous units by the four coauthor groups named below. Proposed automated exclusions were promoted only after deliberation among coauthors and subsequent authorization by the corresponding author; all were therefore author-confirmed. Decisions made by automated methods, decisions made by an individual reviewer, authorities preserved from revision rounds, registries that established identity only, contextual traces and retrieval outcomes were each labeled separately and kept distinct.

Extraction operated at the level of the individual reported estimate and was divided among the same four coauthor groups: LSB, BGN, DLFG and VACA; JTTB, JDGB and MLPM; JASR, EBR and JAGF; and JCDAJ, RMG and MAN. The corresponding author consulted the relevant group about ambiguous source-level items and proposed corrections before authorizing the final extraction. Extracted items covered donor and recipient strata, biopsy timing, technique, processing and reader expertise, the histological construct and its threshold, outcomes and horizons, estimates and their precision, adjustment sets, prediction performance and decision-utility estimands. Only confidence intervals, standard errors, exact two-sided *p* values and exact contingency tables were admitted as routes for reconstructing a variance.

Reports were linked into study and cohort families using identifiers, authorship, centers, recruitment periods, registries and cohort evidence specific to each source. Distinct recruitment periods, or mutually exclusive strata, could count separately; overlap that was demonstrated, or that could not be resolved, was prevented from increasing the number of cohorts contributing to any analysis.

### 2.5. Outcomes and Effect Selection

The co-primary outcomes were delayed graft function (DGF), preferentially defined as dialysis within seven days, and death-censored graft failure. Death-censored and definition-unclear graft failure were combined only within an explicitly labeled family, and all-cause graft failure was kept separate throughout. Reported DGF definitions, censoring rules and follow-up horizons were preserved as published; dialysis indications were not standardized across reports, and cause of failure was not inferred from its timing. Primary non-function, 12-month eGFR, rejection, patient survival, and organ utilization, acceptance and discard were secondary or specific to one question. Odds ratios, hazard ratios, risk ratios and continuous measures were never combined.

Within each analysis—one outcome, one lesion, one effect measure—a single estimate was retained per non-overlapping cohort, chosen by a preference order fixed in advance: adjusted for baseline confounders without post-transplant mediators; adjusted for baseline confounders with an incomplete non-mediator set; crude as reported; and crude recalculated from an exact contingency table. Estimates adjusted for DGF, rejection, post-transplant renal function or any other post-exposure mediator were excluded from the primary analysis. Because the resulting pools therefore mix adjusted with crude estimates, adjustment status is displayed for every contributing effect, and the pooled values are described throughout as average associations across heterogeneous studies rather than as adjusted or independent effects. Clinical compatibility was decided before modeling and never inferred from heterogeneity.

### 2.6. Synthesis Methods

Random-effects models were fitted in R version 4.5.3 using the metafor package version 4.8-0, by restricted maximum likelihood with Hartung–Knapp inference [26]. An analysis containing at least five cohorts was treated as a principal pool and reported with a prediction interval; three or four cohorts were exploratory; two cohorts were displayed without a pooled summary; and a single cohort was summarized narratively. Because Hartung–Knapp intervals can be unstable with only three or four cohorts, the small-k pools were interpreted as exploratory and descriptive rather than confirmatory. Every principal analysis required a sensitivity analysis restricted to adjusted estimates. A moderator analysis of adjustment status required at least ten cohorts, and where it could not be run, this was recorded rather than omitted.

Sensitivity analyses were prespecified for strict outcome definitions, adjustment, leave-one-out omission, sample size, risk of bias, national registries, Banff era, tissue processing, sampling, reader expertise, clustering, and expanded-criteria, donation-after-circulatory-death and high kidney donor profile index strata. Analyses that could not be estimated, and the reasons why, were retained and reported rather than dropped. Funnel-plot asymmetry tests, publication-bias models and meta-regression on adjustment were not performed for any analysis with fewer than ten cohorts.

For eligible DGF odds ratios, we calculated illustrative absolute effects at baseline risks of 20%, 30% and 40%. These are associational scenarios based on hypothetical baseline risks fixed in advance, not observed control-group risks. Graft-failure hazard ratios were not converted to absolute risks, because no compatible baseline survival function and common time horizon were available.

### 2.7. Risk of Bias and Certainty of Evidence

Each design was appraised with the instrument built for it: QUIPS for prognostic factors [17]; CHARMS with PROBAST+AI for prediction models [27,28]; RoB 2, or its cluster variant, for randomized evidence [29,30]; ROBINS-I for non-randomized comparisons [31]; and QAREL where a reliability judgment applied [32]. A historical 27-study PROBAST subset received a second independent methodological review with complete agreement, and a 19-study QUIPS update used an assessor-and-verifier workflow in which five disagreements were reconciled.

Certainty followed GRADE adapted for prognostic factors [22,33], starting at high for each pooled body before downgrading for risk of bias, inconsistency, indirectness, imprecision and publication bias. We took care not to count the same concern about confounding twice across the QUIPS rating, the mixture of adjusted and crude estimates, and the insufficiency of the adjusted-only analysis. For categorical DGF odds ratios, imprecision was judged at a 30% illustrative baseline risk against a prespecified author-judgment threshold of 50 events per 1000, treated as the minimal important difference: a 5-percentage-point absolute increase in delayed graft function was fixed before synthesis as the smallest shift the author group considered decision-relevant, given the association of DGF with additional dialysis, monitoring and subsequent graft outcomes [34,35]; smaller differences were not regarded as sufficient to justify declining an organ. An interval crossing both the null and effects beyond that threshold was downgraded twice, and one crossing either boundary once. Where no valid absolute translation existed, one level was applied if the relative interval crossed unity or if the ratio of its upper to lower limit exceeded four, and two levels if both held. Intervention evidence was rated with conventional GRADE. Prediction evidence was characterized by validation phase, risk of bias and applicability, and measurement evidence was summarized narratively rather than forced into a GRADE rating.

## 3. Results

### 3.1. Study Selection

We identified 45,676 records. After 19,930 duplicates were removed, 25,746 records were screened, of which 25,475 did not advance after score-based title-and-abstract triage and author confirmation. A total of 269 reports were assessed in full text, of which 44 were excluded with reasons: 16 were reviews, editorials, letters or commentaries without original data; 9 were of ineligible design; eight were living-donor-only reports without an extractable deceased-donor subgroup, and in 1 further report, that subgroup could not be separated; 4 informed no eligible review question; 3 reported no relevant time-zero morphological biopsy or biopsy-decision exposure; and 1 each was a consensus or guideline without original data, molecular-only tissue evidence without eligible morphology, and a non-original publication without eligible primary data.

**Figure 1:**
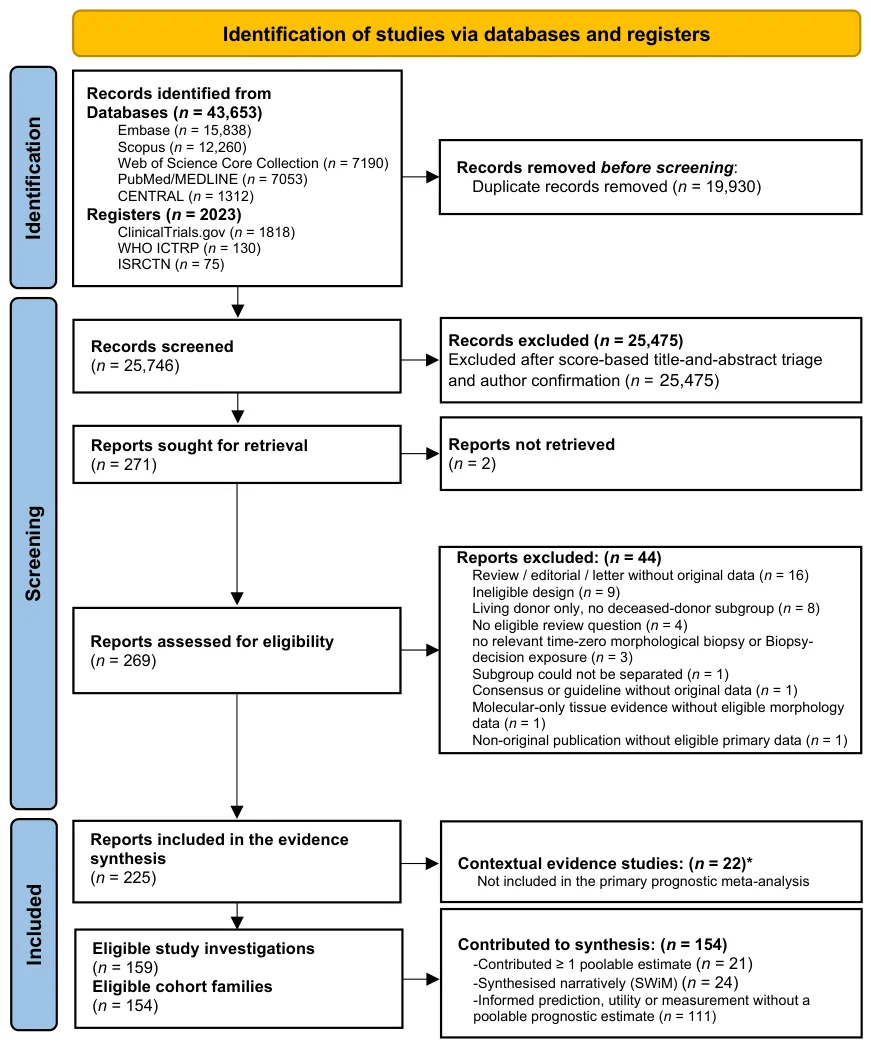
PRISMA 2020 flow diagram. The asterisk (*) identifies the 22 contextual reports, which were excluded from the primary prognostic meta-analysis. The synthesis-contribution categories are not mutually exclusive: two cohort families contributed both a pooled estimate and a separate single-cohort SWiM cell, so the 21, 24 and 111 category counts sum to 156 contributions across 154 unique cohort families. Abbreviations and symbols: CENTRAL, Cochrane Central Register of Controlled Trials; ISRCTN, International Standard Randomized Controlled Trial Number registry; MEDLINE, Medical Literature Analysis and Retrieval System Online; *n*, number of records, reports, studies or cohort families, as indicated; PRISMA, Preferred Reporting Items for Systematic Reviews and Meta-Analyses; SWiM, synthesis without meta-analysis; WHO ICTRP, World Health Organization International Clinical Trials Registry Platform.

A total of 225 reports were included: 203 eligible and 22 contextual. These represented 159 eligible study investigations and 154 eligible cohort families, the units that determine how many independent populations can contribute to any single analysis (Figure 1). Additionally, 180 reports informed the question of prognostic association, 34 incremental prediction, 27 clinical utility and 32 measurement (Table 1).

**Table 1:** Studies contributing to each review question.

Review Question	Question Addressed	Reports	Studies	Cohort Families	Principal Designs	Appraisal	Synthesis
Prognostic association	Does pre-reperfusion morphology associate with post-transplant outcomes?	180 [9,12,13,14,36,37,38,39,40,41,42,43,44,45,46,47,48,49,50,51,52,53,54,55,56,57,58,59,60,61,62,63,64,65,66,67,68,69,70,71,72,73,74,75,76,77,78,79,80,81,82,83,84,85,86,87,88,89,90,91,92,93,94,95,96,97,98,99,100,101,102,103,104,105,106,107,108,109,110,111,112,113,114,115,116,117,118,119,120,121,122,123,124,125,126,127,128,129,130,131,132,133,134,135,136,137,138,139,140,141,142,143,144,145,146,147,148,149,150,151,152,153,154,155,156,157,158,159,160,161,162,163,164,165,166,167,168,169,170,171,172,173,174,175,176,177,178,179,180,181,182,183,184,185,186,187,188,189,190,191,192,193,194,195,196,197,198,199,200,201,202,203,204,205,206,207,208,209,210,211]	139	135	Prospective, retrospective, and registry cohorts	QUIPS; GRADE prognostic factors	One effect per nonoverlapping cohort and analysis; adjusted-only and exactly-matched sensitivities
Incremental prediction	Does histology improve prediction beyond clinical information?	34 [13,164,165,166,167,168,169,170,171,172,173,174,175,176,177,178,179,180,181,182,183,184,185,186,187,188,189,190,209,211,212,213,214,215]	33	33	Model development, internal/external validation, incremental-value studies	CHARMS and PROBAST+AI	Validation phase, performance, calibration, applicability, and narrative synthesis
Clinical utility	Does obtaining or using biopsy information improve utilization or outcomes?	27 [202,203,204,205,206,207,208,210,211,216,217,218,219,220,221,222,223,224,225,226,227,228,229,230,231,232,233]	26	25	Cluster-randomized and comparative nonrandomized strategy studies	RoB 2 cluster or ROBINS-I; conventional GRADE	Design-specific effects and SWiM; no transfer from prognostic associations
Measurement	How reproducible, adequate, or technically valid is the measurement?	32 [191,192,193,194,195,196,197,198,199,200,201,209,210,211,215,234,235,236,237,238,239,240,241,242,243,244,245,246,247,248,249,250]	31	31	Reliability, method-comparison, pre/post timing, and computational pathology studies	QAREL or adapted method-comparison appraisal	Narrative applicability and measurement synthesis

Report, study-investigation, and cohort-family counts overlap across review questions and must not be summed; a report informing more than one question is cited in each. The six contextual reports outside these review-question rows are documented in Supplementary Table S2 [251,252,253,254,255,256]. Abbreviations and symbols: CHARMS, Critical Appraisal and Data Extraction for Systematic Reviews of Prediction Modeling Studies; GRADE, Grading of Recommendations Assessment, Development and Evaluation; PROBAST+AI, Prediction model Risk Of Bias ASsessment Tool plus Artificial Intelligence; QAREL, Quality Appraisal for Reliability Studies; QUIPS, Quality In Prognosis Studies; RoB 2, revised Cochrane risk-of-bias tool for randomized trials; ROBINS-I, Risk Of Bias In Non-randomized Studies of Interventions; SWiM, synthesis without meta-analysis.

### 3.2. Study Characteristics

The evidence base ranged from single-center series to national registry analyses and was predominantly retrospective. Donor populations were overwhelmingly mixed deceased-donor or donation after brain death, with too few exclusively donation-after-circulatory-death cohorts and no stratified expanded-criteria reporting to permit a donor-type-specific synthesis. Biopsy technique, tissue processing and scoring conventions varied widely across studies. Among the effects that entered pooled models, technique was unknown for 17, processing for 16 and reader context for 33. The contribution of each included report—the questions it informed, the route by which it entered the synthesis, and the analyses to which it contributed—is given in Supplementary Table S2; detailed characteristics for prognostic association, incremental prediction, clinical utility and measurement are in Supplementary Tables S3–S5 and S6A,B.

### 3.3. Effect Selection

Reconciliation yielded a universe of 2063 effect rows. Source verification produced 619 field-level corrections across the retained rows, concentrated in 95 reclassified rows; a total of 12 corrections (1.9%) changed a numeric effect-size or variance value, and the remainder updated outcome, lesion or timing semantics, cohort-identity labels, analytic routing or provenance bookkeeping. Of 551 candidates for selection, 440 were quantitatively eligible, and 211 were retained—one per cohort per analysis—across 143 analyses. A total of 7 reached the threshold of five cohorts and were treated as principal pools; 5 were exploratory; 15 contained two cohorts and are displayed without a pooled summary; and 116 contained a single cohort and were summarized narratively (Supplementary Tables S3 and S7; Supplementary Figure S1).

### 3.4. Risk of Bias

**Figure 2:**
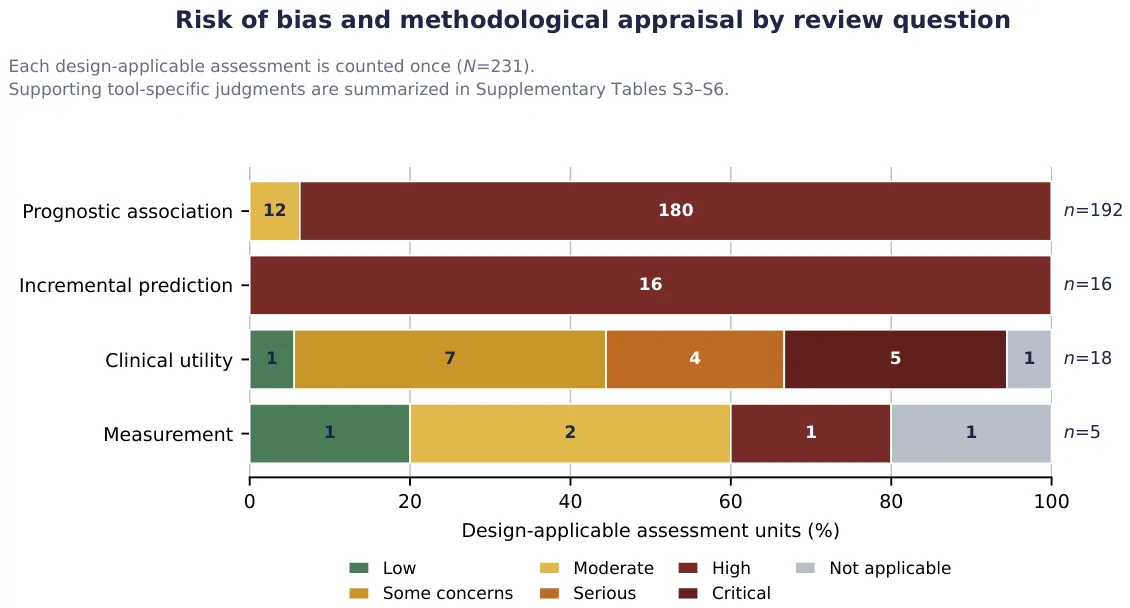
Risk of bias and methodological appraisal by review question. Bars show 231 unique design-applicable assessment units. Tools were QUIPS, CHARMS/PROBAST+AI, RoB 2 or ROBINS-I, and QAREL or an adapted method-comparison appraisal. Percentages exclude two context-only units without an applicable appraisal. Supporting tool-specific judgments are summarized in Supplementary Tables S3–S6. Abbreviations and symbols: CHARMS, Critical Appraisal and Data Extraction for Systematic Reviews of Prediction Modeling Studies; *n*, number of assessment units in each review question; *N*, total number of assessment units; PROBAST+AI, Prediction model Risk Of Bias ASsessment Tool plus Artificial Intelligence; QAREL, Quality Appraisal for Reliability Studies; QUIPS, Quality In Prognosis Studies; RoB 2, revised Cochrane risk-of-bias tool for randomized trials; ROBINS-I, Risk Of Bias In Non-randomized Studies of Interventions.

Risk of bias was high across essentially the whole evidence base. Of the 65 effects contributing to the 12 pooled analyses, 53 were at high risk under QUIPS, and no body composed only of studies at low or moderate risk reached even three cohorts. All four reports providing prediction evidence were at high risk under PROBAST+AI. Appraisal across the four questions is summarized in Figure 2; supporting tool-specific judgments and the appraisal-unit crosswalk are summarized in Supplementary Tables S3–S6.

### 3.5. Delayed Graft Function

**Figure 3:**
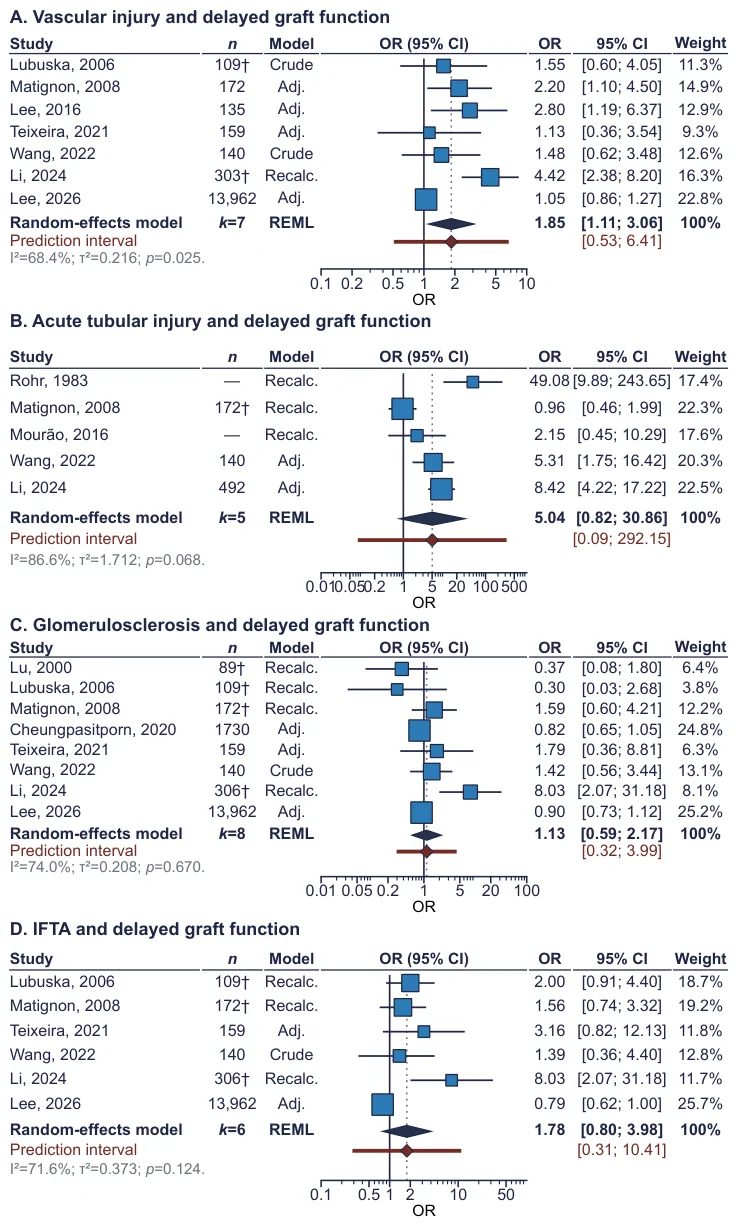
Principal meta-analyses of time-zero kidney morphology and delayed graft function. (**A**), vascular injury; (**B**), acute tubular injury; (**C**), glomerulosclerosis; (**D**), IFTA. Columns give author–year, source-typed *n*, adjustment, OR, 95% CI and weight. The symbol ^†^ marks an exact contingency total; unmarked *n* is the cohort size. These effect-specific source denominators need not sum to the body totals in Supplementary Table S14A. *p* values are two-sided tests of the pooled association, not tests of heterogeneity. Squares show study estimates, with area proportional to weight; horizontal lines through the squares show confidence intervals. Diamonds show REML–Hartung–Knapp pooled estimates, and the separate lines labeled Prediction interval show prediction intervals. All prediction intervals cross the null; mixed-adjustment pools estimate average, not independent, associations. Studies shown: Lubuska, 2006 [47]; Matignon, 2008 [56]; Lee, 2016 [83]; Teixeira, 2021 [181]; Wang, 2022 [184]; Li, 2024 [148]; Lee, 2026 [159]; Rohr, 1983 [192]; Mourão, 2016 [87]; Lu, 2000 [36]; Cheungpasitporn, 2020 [120]. Abbreviations and symbols: τ^2^, between-study variance on the analysis scale; Adj., adjusted estimate; CI, confidence interval; I^2^, percentage of total variability attributable to between-study heterogeneity; IFTA, interstitial fibrosis and tubular atrophy; *k*, number of cohorts in the analytic cell; *n*, reported sample size; OR, odds ratio; Recalc., estimate recalculated from source data; REML, restricted maximum likelihood.

Four principal analyses addressed DGF (Figure 3; Table 2; Supplementary Table S8; Supplementary Figure S2). Vascular injury was associated with higher odds of DGF (seven cohorts; odds ratio: 1.85, 95% CI 1.11–3.06; *p* = 0.025), with substantial heterogeneity (I^2^ = 68.4%) and a prediction interval spanning 0.53 to 6.41.

The other three were inconclusive. Acute tubular injury was extremely imprecise (five cohorts; odds ratio: 5.04, 95% CI 0.82–30.86; I^2^ = 86.6%; prediction interval 0.09–292.15). Its upper tail and much of its heterogeneity were driven by Rohr et al. (1983), a pre-Banff report with an odds ratio of 49.08 (95% CI 9.89–243.65) [192]; omission reduced the pooled estimate to 3.14 (0.62–15.79; four cohorts; I^2^ = 79.5%) without resolving the imprecision (Supplementary Tables S3 and S12; Supplementary Figure S6). Glomerulosclerosis did not exclude the null (eight cohorts; odds ratio: 1.13, 0.59–2.17; I^2^ = 74.0%; prediction interval 0.32–3.99), nor did interstitial fibrosis and tubular atrophy (six cohorts; odds ratio: 1.78, 0.80–3.98; I^2^ = 71.6%; prediction interval 0.31–10.41). Certainty was very low for all four.

Complete leave-one-cohort-out analyses for the glomerulosclerosis and fibrosis bodies are shown in Supplementary Figure S3. The heterogeneity of the glomerulosclerosis analysis is attributable to Li et al. (2024) [148], whose data also influence, without explaining, the heterogeneity of the fibrosis analysis. The Li et al. [148] study contributes numerically identical estimates to both lesions (odds ratio: 8.03, 95% CI 2.07–31.18) because its source table gives identical grade distributions for glomerulosclerosis and interstitial fibrosis; the published data do not allow their covariance, or participant-level concordance between the two lesions, to be estimated, so the two estimates cannot be read as independent corroboration across lesions. Omitting Li et al. [148] leaves glomerulosclerosis at an odds ratio of 0.88 (95% CI 0.73–1.06; seven cohorts; I^2^ = 0.0%) and fibrosis at 1.37 (0.72–2.62; five cohorts; I^2^ = 58.1%).

### 3.6. Graft Failure

**Figure 4:**
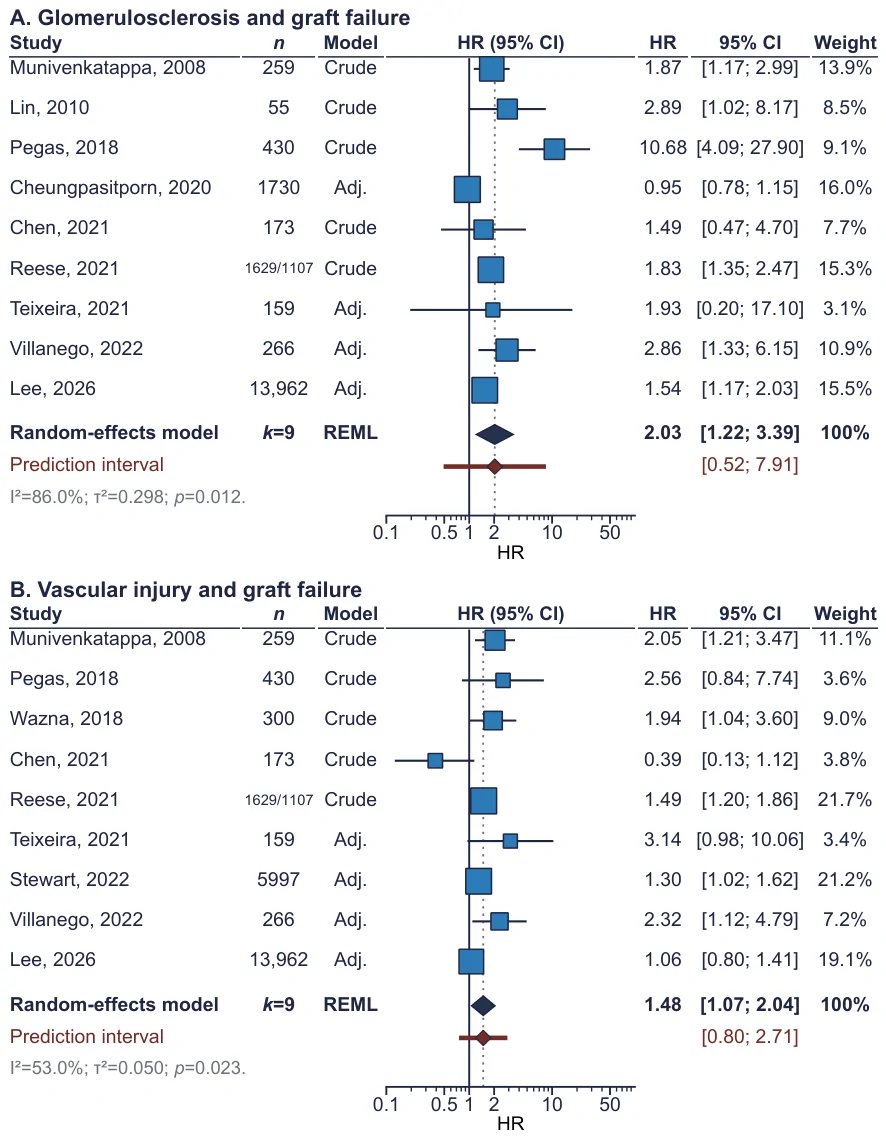
Association of time-zero kidney morphology with graft failure. (**A**), glomerulosclerosis (*k* = 9); (**B**), vascular injury (*k* = 9). Squares show study estimates, with area proportional to weight; horizontal lines show confidence intervals. Diamonds show pooled estimates, and the separate lines labeled Prediction interval show prediction intervals. Unmarked *n* is the source cohort size; *p* values are two-sided tests of the pooled association. The Reese et al. [180] entry shows 1629 participants in the French development cohort and 1107 in the Belgian validation cohort. Both prediction intervals cross the null, and the mixed-adjustment pools estimate average, not independent, associations. Studies shown: Munivenkatappa, 2008 [167]; Lin, 2010 [62]; Pegas, 2018 [96]; Cheungpasitporn, 2020 [120]; Chen, 2021 [179]; Reese, 2021 [180]; Teixeira, 2021 [181]; Villanego, 2022 [131]; Lee, 2026 [159]; Wazna, 2018 [97]; Stewart, 2022 [133]. Abbreviations and symbols: τ^2^, between-study variance on the analysis scale; Adj., adjusted estimate; CI, confidence interval; HR, hazard ratio; I^2^, percentage of total variability attributable to between-study heterogeneity; *k*, number of cohorts in the analytic cell; *n*, reported sample size; REML, restricted maximum likelihood.

Three principal analyses addressed death-censored or definition-unclear graft failure (Figure 4). Glomerulosclerosis (nine cohorts; hazard ratio 2.03, 95% CI 1.22–3.39; *p* = 0.012) and vascular injury (nine cohorts; hazard ratio 1.48, 1.07–2.04; *p* = 0.023) were associated with graft failure on average; interstitial fibrosis and tubular atrophy were not (six cohorts; hazard ratio 1.64, 0.96–2.82). Heterogeneity was high for glomerulosclerosis (I^2^ = 86.0%) and moderate for the other two (53.0% and 50.9%). Certainty was very low for all three.

Every principal prediction interval crossed the null—for glomerulosclerosis, 0.52 to 7.91; for vascular injury, 0.80 to 2.71—so the evidence does not support expecting a consistently non-null association in a new comparable population. All-cause graft failure was kept separate and remained too fragmented to pool, with 24 selected effects spread across 20 analyses and never more than two cohorts in any one.

### 3.7. Clinical Magnitude

At the prespecified illustrative baseline DGF risk of 30%, the corresponding absolute scenarios are shown in Table 2 and Supplementary Table S9. The vascular-injury estimate corresponds to 142 additional events per 1000 (95% interval, 23 to 267 more): the point estimate exceeds the prespecified author-judgment threshold of 50 per 1000, but its lower limit does not. The glomerulosclerosis estimate corresponds to 26 additional events per 1000 (98 fewer to 181 more), a point estimate below that threshold with an interval encompassing both no association and substantially larger effects. These are associational scenarios based on hypothetical baseline risks, not observed control-group risks. Graft-failure hazard ratios were not converted to absolute effects, because no compatible baseline survival function and common horizon were available (Supplementary Table S10).

### 3.8. Sensitivity Analyses

No principal analysis retained five cohorts when restricted to confounder-adjusted estimates, and none of the adjusted-only estimates excluded the null: glomerulosclerosis–DGF, odds ratio: 0.87 (95% CI 0.67–1.13, three cohorts); vascular injury–DGF, 1.55 (0.71–3.37, four cohorts); glomerulosclerosis–graft failure, hazard ratio 1.48 (0.69–3.17, four cohorts); and vascular injury–graft failure, 1.42 (0.75–2.71, four cohorts). The remaining three principal analyses retained two cohorts each (Supplementary Table S11; Supplementary Figure S4). Descriptive adjusted-versus-crude subgroup displays are provided without a moderation claim in Supplementary Figure S5.

On leave-one-out omission, glomerulosclerosis–graft failure retained an interval excluding the null after all nine omissions, vascular injury–graft failure after seven of nine, and vascular injury–DGF after four of seven (Supplementary Table S12; Supplementary Figure S6). Under the strict definition of DGF as dialysis within seven days, the vascular association persisted (five cohorts; odds ratio: 2.37, 95% CI 1.24–4.54), whereas the corresponding glomerulosclerosis and fibrosis analyses did not exclude the null. Excluding national registries preserved the three average associations whose principal intervals excluded the null, but reduced the evidence base; registry strata contributed between 22.8% and 50.0% of the random-effects weight in six of the seven principal analyses (Supplementary Figure S4).

**Figure 5:**
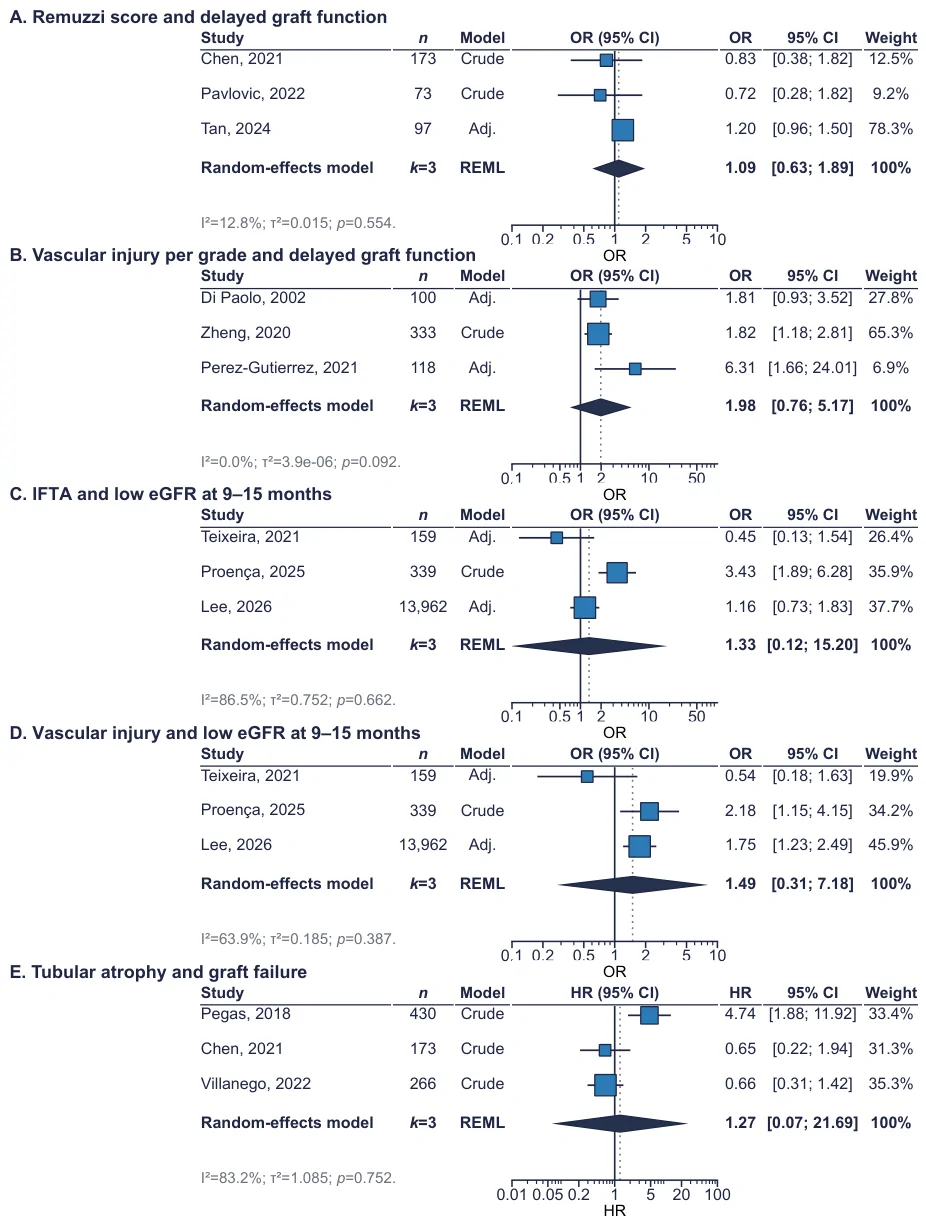
Exploratory meta-analyses of time-zero kidney morphology and transplant outcomes. (**A**), Remuzzi score–DGF; (**B**), vascular injury (per grade)–DGF; (**C**), IFTA–low eGFR; (**D**), vascular injury–low eGFR; (**E**), tubular atrophy–graft failure. Squares show study estimates, with area proportional to weight; horizontal lines show confidence intervals, and diamonds show pooled estimates. Unmarked *n* is the source cohort size; *p* values are two-sided tests of the pooled association. Each pool has *k* = 3; all confidence intervals cross the null and certainty is very low. Studies shown: Chen, 2021 [179]; Pavlovic, 2022 [135]; Tan, 2024 [147]; Di Paolo, 2002 [39]; Zheng, 2020 [119]; Perez-Gutierrez, 2021 [125]; Teixeira, 2021 [181]; Proença, 2025 [188]; Lee, 2026 [159]; Pegas, 2018 [96]; Villanego, 2022 [131]. Abbreviations and symbols: τ^2^, between-study variance on the analysis scale; Adj., adjusted estimate; CI, confidence interval; DGF, delayed graft function; eGFR, estimated glomerular filtration rate; HR, hazard ratio; I^2^, percentage of total variability attributable to between-study heterogeneity; IFTA, interstitial fibrosis and tubular atrophy; *k*, number of cohorts in the analytic cell; *n*, reported sample size; OR, odds ratio; REML, restricted maximum likelihood.

None of the five exploratory pools excluded the null: Remuzzi score with DGF (three cohorts; odds ratio: 1.09, 95% CI 0.63–1.89), vascular injury (per grade) with DGF (1.98, 0.76–5.17), fibrosis with low eGFR (1.33, 0.12–15.20), vascular injury with low eGFR (1.49, 0.31–7.18), and tubular atrophy with graft failure (hazard ratio 1.27, 0.07–21.69), each from three cohorts and all of very low certainty (Figure 5; Supplementary Tables S8 and S14A,B). Within the Proença et al. (2025) cohort, the reported low-eGFR associations for interstitial fibrosis (ci1 versus ci0; odds ratio: 3.43, 95% CI 1.89–6.28) and tubular atrophy (ct1 versus ct0; 3.44, 1.88–6.28) were nearly identical [188]. As with the duplicated Li et al. [148] lesion distributions, these same-cohort estimates should not be read as independent corroboration across lesions.

**Figure 6:**
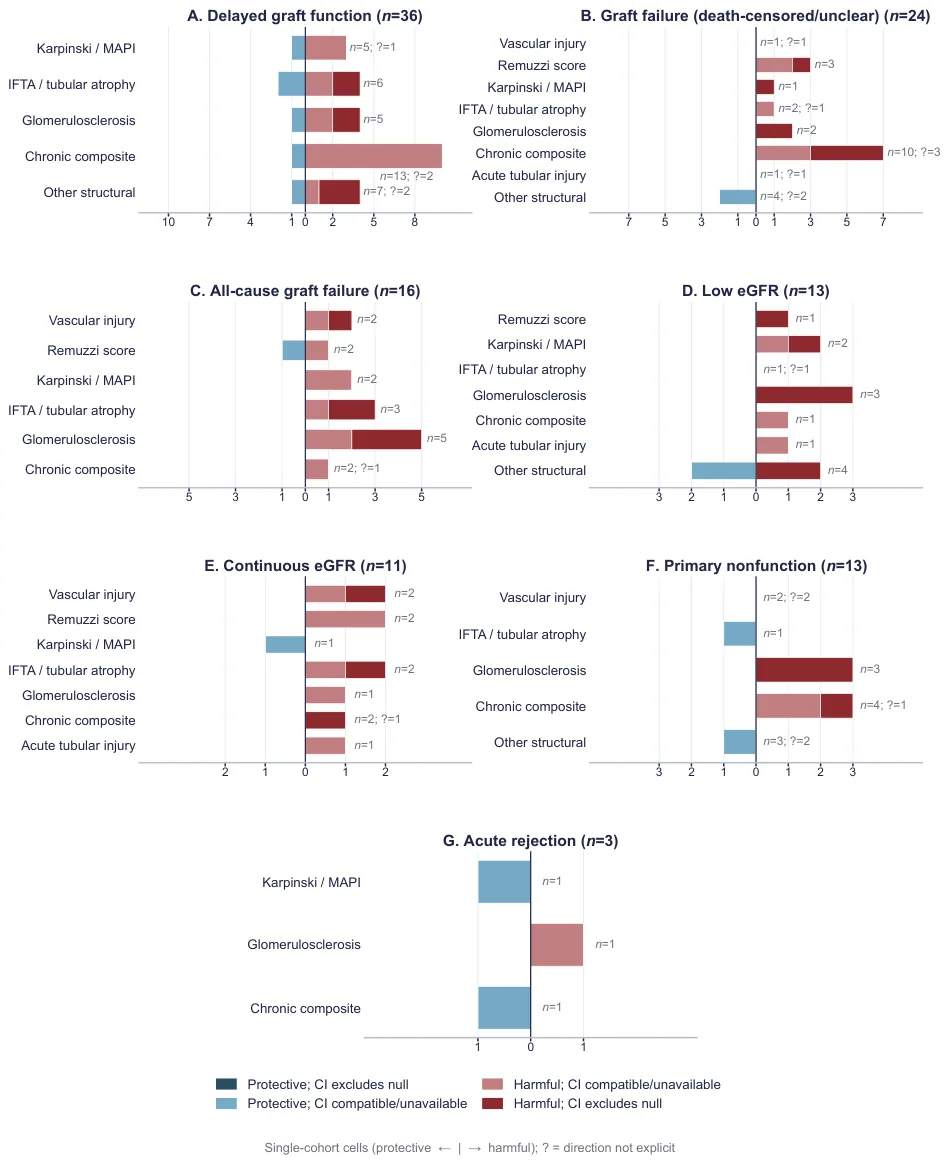
Synthesis without meta-analysis direction map for 116 single-cohort analytic cells. Rows group cells by outcome and lesion family; bars point left for protective and right for harmful. Dark segments denote confidence intervals excluding the null; light segments denote intervals compatible with the null or unavailable precision, and question marks denote uncertain direction. Counts describe evidence architecture, not a pooled or precision-weighted effect. Abbreviations and symbols: CI, confidence interval; eGFR, estimated glomerular filtration rate; IFTA, interstitial fibrosis and tubular atrophy; MAPI, Maryland Aggregate Pathology Index; *n*, number of single-cohort analytic cells in the displayed group.

The 116 single-cohort cells were synthesized without meta-analysis (SWiM) because no compatible second cohort was available (Figure 6; Supplementary Table S3). Of 95 cells with an explicitly orientable contrast, 79 pointed toward harm (35 with a confidence interval excluding the null and 44 compatible with the null or without reported precision) and 16 toward protection, none with a confidence interval excluding the null; a total of 21 source contrasts did not support secure direction assignment. These counts describe evidence architecture only: a report can contribute to more than one outcome–lesion cell, cells are not weighted by precision or sample size, and direction counts are not an estimate of a common effect.

Exact clinical replication was rare: the 65 modeled effects formed 63 distinct combinations of population, lesion definition, outcome and horizon, none of which contained three cohorts. No analysis reached 10 cohorts, so funnel-plot asymmetry tests, publication-bias models and meta-regression on adjustment could not be run; publication bias should be regarded as unassessed rather than absent (Supplementary Table S13; Supplementary Figure S7).

### 3.9. Prediction, Clinical Utility and Measurement

A total of 34 reports were classified as relevant to the incremental-prediction question; only 4 provided extractable phase-specific model-performance data, yielding 16 assessments spanning model development, apparent performance and external evaluation [174,176,185,214]. Two numerical comparisons illustrate why association should not be equated with incremental prediction. In Ernst et al. [214], the external DGF AUC was 0.702 for the clinical model and 0.701 after the histology update, while the calibration slope changed from 0.628 to 0.388. In Traynor et al. [174], adding the Karpinski score changed the apparent five-year low-eGFR AUC/C-statistic from 0.64 to 0.67, with no evidence of improvement (*p* = 0.65). All four were at high risk of bias. Calibration was absent or poor where reported, and no decision-curve analysis, prospective impact study or randomized evaluation established added clinical value (Supplementary Table S4).

A total of 27 reports were classified as relevant to clinical utility, and 7 supplied the 17 extractable strategy–outcome rows detailed in Supplementary Table S5. The only randomized evaluation of policy tested access to a national digital biopsy service rather than the validity of any lesion [233]. It did not demonstrate improvement in transplantation at first offer (adjusted odds ratio: 0.91, 97.5% CI 0.60–1.39; moderate certainty), in overall transplantation at last offer (0.99, 95% CI 0.70–1.42; low certainty), in DGF (1.12, 95% CI 0.67–1.86; very low certainty) or in 12-month eGFR (mean difference 1.53 mL/min/1.73 m^2^, 97.5% CI −2.33 to 5.40; low certainty). Non-randomized comparisons of strategy were at serious or critical risk of bias and were kept separate. Selected certainty profiles are summarized in Table 3.

A total of 32 reports were classified as relevant to measurement, and 16 contributed the detailed evidence rows in Supplementary Table S6A,B. They described agreement between readers, comparisons of frozen with paraffin sections and of wedge with core sampling, change between pre- and post-reperfusion biopsies, and computational segmentation. Six prespecified measurement questions ranged from low to moderate quality or did not admit a reliability judgment, and were synthesized narratively. Technical measurement performance was not carried over into claims about clinical prediction.

### 3.10. Certainty of Evidence

**Figure 7:**
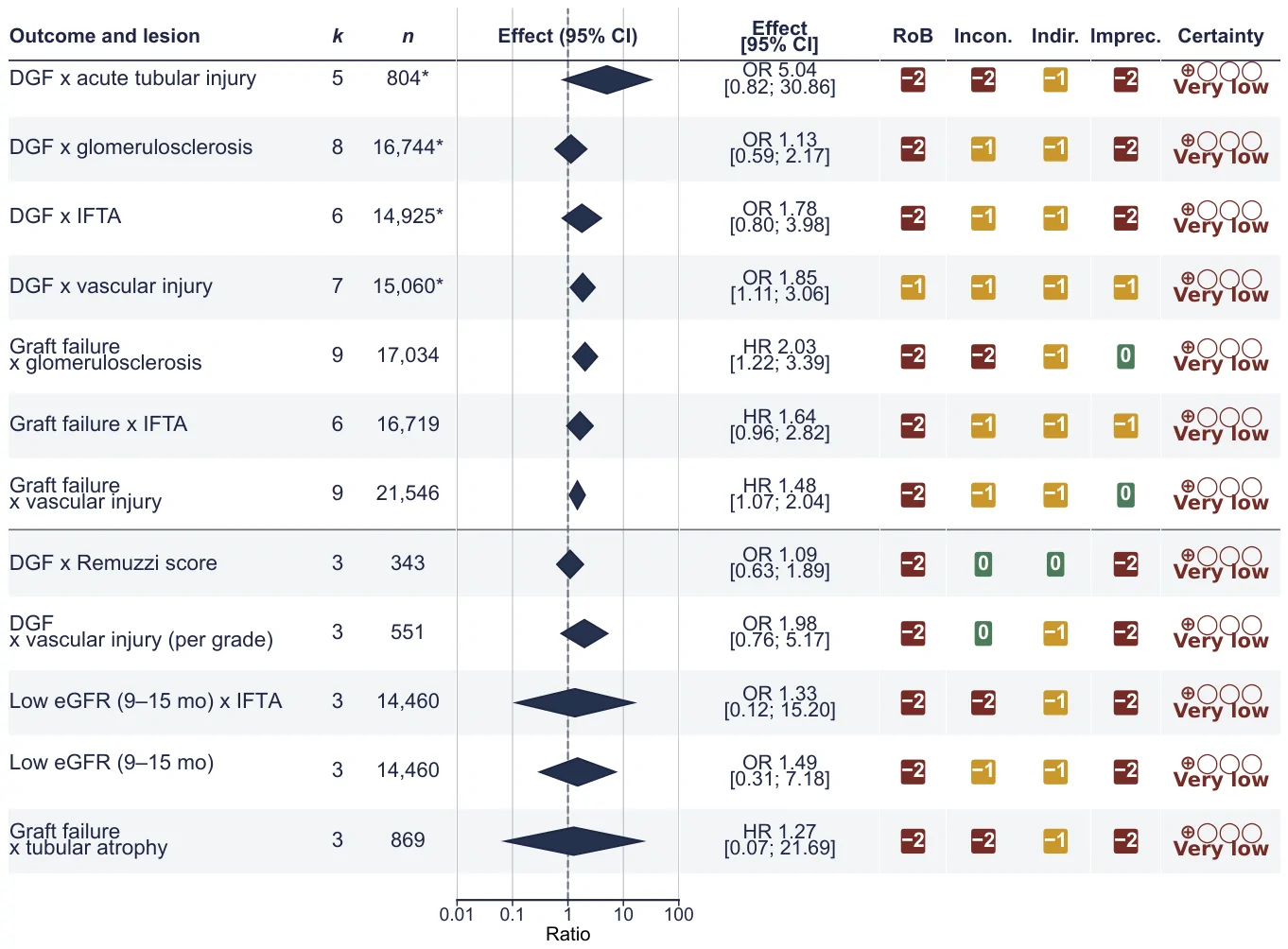
Prognostic GRADE evidence profile. Diamonds show pooled estimates; badges report downgrading for risk of bias, inconsistency, indirectness and imprecision. The *n* column sums available numeric analytic denominators; an asterisk (*) indicates that one or more additional cohorts had an unknown analytic denominator, so the displayed *n* is a minimum documented total. All 12 bodies were very low certainty; publication bias was unassessable because *k* < 10. Abbreviations and symbols: CI, confidence interval; DGF, delayed graft function; eGFR, estimated glomerular filtration rate; GRADE, Grading of Recommendations Assessment, Development and Evaluation; HR, hazard ratio; IFTA, interstitial fibrosis and tubular atrophy; Imprec., imprecision; Incon., inconsistency; Indir., indirectness; *k*, number of cohorts in the analytic cell; mo, months; *n*, reported sample size; OR, odds ratio; RoB, risk of bias.

All 12 pooled prognostic bodies were rated very low certainty, driven by high risk of bias throughout, by inconsistency, and by imprecision judged against the prespecified author-judgment threshold. No body was rated up, and none reached moderate or high certainty (Supplementary Table S14A,B; Figure 7).

## 4. Discussion

### 4.1. Principal Findings and Strengths

This review identified three average prognostic associations whose confidence intervals excluded the null: vascular injury with DGF, glomerulosclerosis with graft failure, and vascular injury with graft failure. These signals do not constitute validated decision rules. Every principal prediction interval included the null, no adjusted-only analysis retained five cohorts or excluded the null, and all 12 pooled bodies were of very low certainty. The evidence therefore supports a firm distinction between association and information that is sufficiently reliable to guide organ acceptance or discard.

The three signals differed in robustness. Vascular injury–DGF persisted under the strictest outcome definition (odds ratio: 2.37, 95% CI 1.24–4.54; five cohorts) but lost null exclusion after three of seven leave-one-out omissions; glomerulosclerosis–graft failure retained null exclusion after all nine omissions despite high heterogeneity (I^2^ = 86.0%), and vascular injury–graft failure lost it after two of nine. Clinical magnitude also differed: at an illustrative 30% baseline DGF risk, the vascular estimate corresponded to 142 additional events per 1000 (95% interval, 23 to 267 more), exceeding the prespecified 50-per-1000 threshold at the point estimate but not at its lower limit, whereas glomerulosclerosis–DGF (26 per 1000; 98 fewer to 181 more) was inconclusive on both counts. The heterogeneity of that cell (I^2^ = 74.0%) was attributable almost entirely to Li et al. [148], whose source table reports identical grade distributions for glomerulosclerosis and IFTA; with that study omitted, the cell was homogeneous and compatible with the null (odds ratio: 0.88, 95% CI 0.73–1.06). These findings sharpen rather than overturn the earlier systematic review, which found no consistent association without pooling: the present cohort-level synthesis identifies three average associations while confirming that none has been validated as a predictor [15,148].

Methodological safeguards strengthen that interpretation. Effects were synthesized by cohort rather than report, overlap was explicitly controlled, appraisal tools were matched to study design, and one effect was selected per cohort and analytic question. All pooled models were independently reproduced, failed sensitivity analyses were reported, and estimates with unusable variances were not silently repaired. Death-censored and all-cause graft failure remained separate; SWiM was used when pooling was inappropriate, and certainty was assessed with GRADE adapted for prognosis [21,22,26].

### 4.2. Association Versus Incremental Prediction and Utility

The numerical prediction results make the distinction clinically concrete. In the external DGF evaluation by Ernst et al. [214], adding histology changed the AUC from 0.702 to 0.701 and the calibration slope from 0.628 to 0.388. In Traynor et al. [174], adding the Karpinski score changed the apparent five-year low-eGFR AUC/C-statistic from 0.64 to 0.67, without evidence of improvement (*p* = 0.65). Thus, a lesion can be associated with outcome while adding little or no predictive information to clinical variables [174,214].

Donor age, function and KDPI are available before biopsy interpretation, and age-based allocation already structures practice in programs such as the Eurotransplant Senior Program. Evidence from older-donor allocation also indicates that biopsy-based differential allocation has not demonstrated an advantage over allocation based on simple clinical indications. The relevant benchmark is therefore not whether histology is associated with outcome, but whether it improves prediction, calibration or decisions beyond these established variables and the allocation context [95,152].

Clinical-utility evidence does not bridge this gap. Discard studies are vulnerable to incorporation bias because biopsy often determines the outcome being studied. In a setting where histology was not used for allocation, it did not materially improve graft-failure discrimination. The randomized PITHIA policy trial evaluated access to a national digital biopsy service, not a lesion-based rule, and did not demonstrate improvement in transplantation, DGF or 12-month eGFR [7,180,233].

### 4.3. Outcome Interpretation and Clinical Implications

DGF is a clinically important but multicausal endpoint. Dialysis within seven days provides a reproducible time window, yet the decision to initiate dialysis depends partly on recipient-specific conditions such as hyperkalemia or volume overload and on clinician judgment. The strict-definition sensitivity analysis therefore improves consistency of timing but cannot make DGF specific to pre-existing donor histology. This helps explain why even a biologically plausible biopsy signal may have limited predictive specificity.

Graft failure is also heterogeneous in causal meaning. Early failure may follow surgical, vascular or ureteral complications, infection or acute rejection that is not represented by preimplantation histology, whereas later failure may be more compatible with pre-existing chronic damage, hypertension or drug toxicity. The included reports did not provide sufficiently consistent timing and cause data to separate these mechanisms. The pooled graft-failure estimates should consequently be interpreted as average associations across heterogeneous horizons, not as evidence that baseline histology caused an individual failure.

The practice implication is direct: time-zero histology should not be used as a stand-alone gate for accepting or discarding a deceased-donor kidney, and isolated glomerulosclerosis should not determine discard. Histology is better interpreted alongside clinical data and donor-quality indices such as the KDPI, and dual-kidney allocation remains a reasonable option for selected marginal organs. Current evidence does not show that changing perioperative care or machine-perfusion management in response to vascular injury improves outcomes; vascular injury is therefore a hypothesis for future prospective investigation, not a current management indication [222,257].

### 4.4. Limitations

The principal limitation is the high risk of bias across the evidence base. All seven principal pools mixed adjusted and crude estimates, and no adjusted-only or lower-risk body provided pooled confirmation. Exact replication was rare: the 65 modeled effects represented 63 combinations of population, lesion, outcome and horizon. Lesion thresholds, sampling, processing, reader expertise, donor strata and follow-up varied substantially; technique was unknown for 17 effects, processing for 16 and reader context for 33. The five exploratory pools each contained only three cohorts, and no cell reached the threshold for funnel-plot or meta-regression procedures. Publication bias is therefore unassessed, not absent.

Outcome limitations add further indirectness. DGF definitions did not standardize indications for dialysis, and graft-failure reports could not be separated reliably by timing or cause. Few cohorts were restricted to DCD donors, ECD results were not adequately stratified, and discarded kidneys cannot contribute observed post-transplant outcomes. These constraints precluded credible post hoc analyses by dialysis indication, early versus late failure, or biopsy value beyond donor age; attempting them would have created unsupported precision rather than resolving these limitations.

### 4.5. Focused Research Priorities

The priority is not another series reporting an unadjusted lesion–outcome association. Linked national or multicenter registries should combine standardized acquisition, adequacy criteria and expert histologic assessment with prespecified clinical comparators, including donor age, kidney function, KDPI and allocation program. Analyses should report time-resolved and, where possible, cause-specific graft failure; explicitly document dialysis indications; quantify incremental discrimination and calibration; and validate performance across health systems [12,258].

Only if standardized histology demonstrates reproducible incremental value should prospective studies test whether biopsy-guided changes in allocation, perioperative care or machine perfusion improve patient and graft outcomes. Such studies should distinguish access to biopsy, receipt and technical adequacy of the specimen, disclosure of the result, and the action taken. This sequence addresses the unresolved decision question without reopening the already well-described question of prognostic association.

## 5. Conclusions

Current evidence does not support time-zero histology as a stand-alone rule for accepting or discarding a deceased-donor kidney. The three pooled association signals remain of very low certainty and do not establish incremental prediction or clinical utility. The unresolved question is narrower: whether standardized histologic assessment adds useful information beyond donor age, kidney function, KDPI and allocation context. That question should be evaluated in linked national or multicenter registries before biopsy findings are used to direct perioperative or machine-perfusion management. 

## Figures and Tables

**Table 2:** Principal prognostic associations and sensitivity analyses.

Outcome and Construct	*k*	Effect (95% CI)	I^2^ (%)	Prediction Interval	Certainty	Illustrative Absolute Effect at 30% DGF Risk	Adjusted-Only Sensitivity	Interpretation
DGF—acute tubular injury	5	OR 5.04 (0.82–30.86)	86.6	0.09–292.15	Very low	+384 per 1000 (−39 to +630); hypothetical baseline, associational	*k* = 2; no pooled model	Principal CI and PI cross the null; adjusted-only evidence falls below exploratory pooling.
DGF—glomerulosclerosis	8	OR 1.13 (0.59–2.17)	74.0	0.32–3.99	Very low	+26 per 1000 (−98 to +181); hypothetical baseline, associational	*k* = 3; OR 0.87 (0.67–1.13)	Point estimate is below the 50-per-1000 MID, but the CI spans both the null and effects larger than the MID.
DGF—IFTA	6	OR 1.78 (0.80–3.98)	71.6	0.31–10.41	Very low	+133 per 1000 (−45 to +330); hypothetical baseline, associational	*k* = 2; no pooled model	Principal CI and PI cross the null; adjusted-only evidence falls below exploratory pooling.
DGF—vascular injury	7	OR 1.85 (1.11–3.06)	68.4	0.53–6.41	Very low	+142 per 1000 (+23 to +267); hypothetical baseline, associational	*k* = 4; OR 1.55 (0.71–3.37)	Point estimate exceeds the 50-per-1000 MID, but the lower CI does not; null exclusion is retained in 4/7 leave-one-out runs.
Graft failure—glomerulosclerosis	9	HR 2.03 (1.22–3.39)	86.0	0.52–7.91	Very low	Not estimable: no aligned baseline survival and common horizon.	*k* = 4; HR 1.48 (0.69–3.17)	Null exclusion is retained in 9/9 leave-one-out runs, but the PI crosses the null and outcome definition is death-censored or unclear.
Graft failure—IFTA	6	HR 1.64 (0.96–2.82)	50.9	0.64–4.20	Very low	Not estimable: no aligned baseline survival and common horizon.	*k* = 2; no pooled model	Principal CI and PI cross the null; adjusted-only evidence falls below exploratory pooling.
Graft failure—vascular injury	9	HR 1.48 (1.07–2.04)	53.0	0.80–2.71	Very low	Not estimable: no aligned baseline survival and common horizon.	*k* = 4; HR 1.42 (0.75–2.71)	Null exclusion is retained in 7/9 leave-one-out runs; the PI crosses the null and outcome definition is death-censored or unclear.

All principal pools mix adjustment states. DGF absolute effects use a hypothetical 30% baseline risk and are associational, not causal. The 50-per-1000 MID was a prespecified author-judgment threshold. Abbreviations and symbols: CI, confidence interval; DGF, delayed graft function; HR, hazard ratio; I^2^, percentage of total variability attributable to between-study heterogeneity; IFTA, interstitial fibrosis and tubular atrophy; *k*, number of cohorts in the analytic cell; MID, minimal important difference; OR, odds ratio; PI, prediction interval.

**Table 3:** Summary of findings for selected outcomes.

Review Question	Question or Outcome	*k* or Design	Effect	Certainty	Interpretation
Prognostic association	DGF—glomerulosclerosis	*k* = 8; principal analysis	OR 1.13 (95% CI 0.59–2.17)	Very low	The average association is inconclusive; the illustrative 30% baseline-risk difference is +26 per 1000 (−98 to +181), spanning the null and clinically important effects.
Prognostic association	DGF—vascular injury	*k* = 7; principal analysis	OR 1.85 (95% CI 1.11–3.06)	Very low	The average association excludes the null, but the PI crosses it; the illustrative 30% baseline-risk difference is +142 per 1000 (+23 to +267), and the lower limit does not reach the 50-per-1000 MID.
Prognostic association	Graft failure—glomerulosclerosis	*k* = 9; principal analysis	HR 2.03 (95% CI 1.22–3.39)	Very low	The average association excludes the null and is leave-one-out stable, but heterogeneity is high, the PI crosses the null, adjusted-only *k* is four, and the outcome is death-censored or definition-unclear.
Prognostic association	Graft failure—vascular injury	*k* = 9; principal analysis	HR 1.48 (95% CI 1.07–2.04)	Very low	The average association excludes the null, but the PI crosses it, null exclusion is lost in two leave-one-out runs, adjusted-only *k* is four, and the outcome is death-censored or definition-unclear.
Clinical utility	Transplantation at first offer	Stepped-wedge cluster-randomized registry trial	Adjusted OR 0.91 (97.5% CI 0.60–1.39); RD −0.01 (97.5% CI −0.08 to 0.06)	Moderate	The co-primary policy outcome includes potentially important decreases and increases; the estimand is access to the biopsy service, not receipt of a biopsy.
Clinical utility	Overall transplantation at last offer	Stepped-wedge cluster-randomized registry trial	Adjusted OR 0.99 (95% CI 0.70–1.42)	Low	The interval includes potentially important decreases and increases; the estimand is policy access and intervention uptake was incomplete.
Clinical utility	DGF—dialysis within 7 days	Stepped-wedge cluster-randomized registry trial	Adjusted OR 1.12 (95% CI 0.67–1.86); RD −0.01 (95% CI −0.10 to 0.08)	Very low	The interval spans substantial benefit and harm for an important early graft outcome; the estimand is policy access and intervention uptake was incomplete.
Clinical utility	eGFR at 12 months	Stepped-wedge cluster-randomized registry trial	Adjusted MD 1.53 mL/min/1.73 m^2^ (97.5% CI −2.33 to 5.40)	Low	The interval includes no difference, and a clinically important improvement was not demonstrated; the estimand is policy access and intervention uptake was incomplete.

Prognostic bodies began at high certainty under prognostic-factor GRADE and reached the categorical floor of very low. Confounding-related QUIPS, mixed-adjustment and adjusted-only limitations were combined by their maximum supported downgrade, not summed; inconsistency and imprecision remained separate. DGF imprecision used a hypothetical 30% baseline risk and a prespecified author-judgment threshold of 50 per 1000, treated as the minimal important difference: crossing both null and important-effect boundaries incurred two levels, either boundary one. Otherwise, one level applied for null crossing or a CI ratio above 4, and two for both. Publication bias was unassessable (*k* < 10), not absent. The supplement gives the complete 12-row profile, uncapped totals and cell-specific rationales. Abbreviations and symbols: CI, confidence interval; DGF, delayed graft function; eGFR, estimated glomerular filtration rate; GRADE, Grading of Recommendations Assessment, Development and Evaluation; HR, hazard ratio; *k*, number of cohorts in the analytic cell; m^2^, square meters; MD, mean difference; MID, minimal important difference; min, minutes; mL, milliliters; OR, odds ratio; PI, prediction interval; QUIPS, Quality In Prognosis Studies; RD, risk difference.

## Data Availability

The versioned analysis package supporting this review is deposited at Zenodo (version 3.0.1; DOI 10.5281/zenodo.22289139, https://doi.org/10.5281/zenodo.22289139; the concept DOI 10.5281/zenodo.21812528 resolves to the latest version). The package contains the statistical synthesis code, input and output validators, an independent numerical recomputation, the extraction table (2063 effect rows), the selected-effect input (211 effects), the screening decision ledger (25,746 records), the generated model outputs (12 pooled models, 216 sensitivity routes, 65 leave-one-out analyses), the certainty assessments (12 GRADE bodies), and a data dictionary. Version 3.0.1 adds the public column names required by the distributed scripts so that the documented wrapper runs without modification; no selected effect or estimate changed.

## Supplementary Materials

The following supporting information can be downloaded at: https://www.mdpi.com/article/10.3390/medsci14050566/s1. Table S1a. Search strategies as executed; Table S1b. Final source execution; Table S1c. PRESS 2015-guided peer-review assessment, response and final disposition; Table S2. Characteristics of included reports and their contribution to the synthesis; Table S3. Figure 2 appraisal crosswalk and prognostic-association evidence selected for synthesis; Table S4. Incremental-prediction model phase, performance, risk of bias and applicability; Table S5. Clinical-utility risk-of-bias assessments, effects and outcome-level certainty; Table S6A. Measurement evidence; Table S6B. Measurement applicability and quality; Table S7. Effect selection, dependency decisions and final analysis routing; Table S8. All REML–Hartung–Knapp pooled results; Table S9. Illustrative absolute effects for delayed graft function; Table S10. Why graft-failure absolute effects were not estimable; Table S11. Prespecified sensitivity-analysis completion; Table S12. Complete leave-one-out and influence summary; Table S13. All exactly matched clinical strata; Table S14A. Complete prognostic Summary of Findings; Table S14B. Complete prognostic certainty-domain profile; Figure S1. Two-cohort lesion–outcome comparisons without pooled summaries; Figure S2. Complete principal meta-analyses of delayed graft function and graft failure; Figure S3. Focused leave-one-cohort-out analyses of glomerulosclerosis and IFTA with delayed graft function; Figure S4. Prespecified sensitivity analyses for all pooled lesion–outcome bodies; Figure S5. Adjustment-stratified analyses of all pooled lesion–outcome bodies; Figure S6. Complete leave-one-cohort-out analyses of all pooled lesion–outcome bodies; Figure S7. Funnel-plot eligibility audit. Completed PRISMA 2020, PRISMA 2020 for Abstracts, PRISMA-S, SWiM and TRIPOD-SRMA reporting checklists; AMSTAR-PF self-audit. The reports documented in the supplementary tables and figures are included in the main reference list [9,12,13,14,36,37,38,39,40,41,42,43,44,45,46,47,48,49,50,51,52,53,54,55,56,57,58,59,60,61,62,63,64,65,66,67,68,69,70,71,72,73,74,75,76,77,78,79,80,81,82,83,84,85,86,87,88,89,90,91,92,93,94,95,96,97,98,99,100,101,102,103,104,105,106,107,108,109,110,111,112,113,114,115,116,117,118,119,120,121,122,123,124,125,126,127,128,129,130,131,132,133,134,135,136,137,138,139,140,141,142,143,144,145,146,147,148,149,150,151,152,153,154,155,156,157,158,159,160,161,162,163,164,165,166,167,168,169,170,171,172,173,174,175,176,177,178,179,180,181,182,183,184,185,186,187,188,189,190,191,192,193,194,195,196,197,198,199,200,201,202,203,204,205,206,207,208,209,210,211,212,213,214,215,216,217,218,219,220,221,222,223,224,225,226,227,228,229,230,231,232,233,234,235,236,237,238,239,240,241,242,243,244,245,246,247,248,249,250,251,252,253,254,255,256]. The supplementary search, appraisal, certainty and reporting materials use the corresponding methodological guidance [17,19,20,21,22,23,24,25,27,28,29,30,31,32,33].

## Abbreviations

The following abbreviations are used in this manuscript:

AMSTAR	A MeaSurement Tool to Assess systematic Reviews
CENTRAL	Cochrane Central Register of Controlled Trials
CHARMS	Critical Appraisal and Data Extraction for Systematic Reviews of Prediction Modeling Studies
CI	Confidence interval
DCD	Donation after circulatory death
DGF	Delayed graft function
ECD	Expanded-criteria donor
eGFR	Estimated glomerular filtration rate
GRADE	Grading of Recommendations Assessment, Development and Evaluation
HR	Hazard ratio
ICTRP	International Clinical Trials Registry Platform
IFTA	Interstitial fibrosis and tubular atrophy
KDPI	Kidney Donor Profile Index
MID	Minimal important difference
OR	Odds ratio
PRESS	Peer Review of Electronic Search Strategies
PRISMA	Preferred Reporting Items for Systematic Reviews and Meta-Analyses
PROBAST	Prediction model Risk Of Bias ASsessment Tool
PROSPERO	International Prospective Register of Systematic Reviews
QAREL	Quality Appraisal for Reliability Studies
QUIPS	Quality In Prognosis Studies
REML	Restricted maximum likelihood
ROBINS-I	Risk Of Bias In Non-randomized Studies of Interventions
SWiM	Synthesis Without Meta-analysis
TRIPOD	Transparent Reporting of a multivariable prediction model for Individual Prognosis Or Diagnosis
