# Supplementary material for: Time-Zero Kidney Biopsy and Deceased-Donor Transplant Outcomes: A Systematic Review and Meta-Analysis of Prognostic Value, Prediction, and Clinical Utility
Source: Med Sci (Basel). 2026 Sep 12;14(5):566. doi: 10.3390/medsci14050566 (PMC13609387; doi:10.3390/medsci14050566)
Supplement: Supplementary file 1 [file medsci-14-00566-s001.zip › medsci-4530175_Supplementary_Materials_Revised 9.18.pdf]

Supplementary Materials

Time-Zero Kidney Biopsy and Deceased-Donor Transplant Outcomes: A Systematic Review and Meta-Analysis of Prognostic Value, Prediction, and Clinical Utility

Livier Sainz-Bravo [1], Benjamín Gómez-Navarro [2,3], Diana Laura Figueroa-Gamiño [4], Jenniffer Topacio Tapia-Báez [5], José David González-Barajas [1,6], Manuel Luis Prieto-Magallanes [1,7], José Ángel Solís-Rojas [8], Edgar Bautista-Reyes [9], Juan Alberto Gómez-Fregoso [6,10], Judith Carolina De Arcos-Jiménez [1,7], Ramón Medina-González [7,10], Violeta Aidee Camarena-Arteaga [1], Manuel Arizaga-Napoles [10] and Jaime Briseno-Ramirez [1,7,*]

[1] Hospital Civil de Oriente, Tonalá 45425, Mexico; lsainz@hcg.gob.mx (L.S.-B.); jdgonzalez@hcg.gob.mx (J.D.G.-B.); mlprieto@hcg.gob.mx (M.L.P.-M.); judith.dearcos@academicos.udg.mx (J.C.D.A.-J.); 2025009@correo.opdhcg.net (V.A.C.-A.)
[2] Consejo Estatal de Trasplantes de Jalisco (CETRAJAL), Guadalajara 45177, Mexico; gonfre@gmail.com
[3] Centro Médico Puerta de Hierro Norte, Zapopan 45116, Mexico
[4] Hospital Juárez de México, Mexico City 07760, Mexico; aprptdiana@gmail.com
[5] Hospital General de México “Dr. Eduardo Liceaga”, Mexico City 06720, Mexico; tapiabaez07@gmail.com
[6] Centro Universitario de Ciencias de la Salud, Universidad de Guadalajara, Guadalajara 44340, Mexico; drjuangomezf@gmail.com
[7] Centro Universitario de Tlajomulco, Universidad de Guadalajara, Tlajomulco 45641, Mexico; ramon.medinagonzalez@academicos.udg.mx
[8] Hospital Regional de Alta Especialidad del Instituto de Seguridad y Servicios Sociales de los Trabajadores del Estado (ISSSTE), Acapulco 39850, Mexico; joseangel19100@gmail.com
[9] Centro Médico Nacional 20 de Noviembre, Instituto de Seguridad y Servicios Sociales de los Trabajadores del Estado (ISSSTE), Mexico City 03229, Mexico; edgbr.3@gmail.com
[10] Antiguo Hospital Civil de Guadalajara “Fray Antonio Alcalde”, Guadalajara 44280, Mexico; manarizaganapoles@gmail.com
* Correspondence: jaime.briseno@academicos.udg.mx; Tel.: +52-33-1175-3259

Supplementary Materials index

S1a. Search strategies as executed	2	S14A. Complete prognostic Summary of Findings	48
S1b. Final source execution	7	S14B. Certainty-domain profile	50
S1c. PRESS 2015 peer-review assessment	8	Figure S1: Two-cohort evidence	51
S2. Characteristics of included reports	9	Figure S2: Principal pooled analyses	53
S3. Appraisal crosswalk and prognostic evidence	20	Figure S3: Focused DGF leave-one-cohort-out analyses	55
S4. Incremental-prediction evidence	30	Figure S4: Prespecified sensitivity analyses	56
S5. Clinical-utility evidence	31	Figure S5: Adjustment-stratified analyses	59
S6A. Measurement evidence	33	Figure S6: Complete leave-one-cohort-out analyses	61
S6B. Measurement applicability and quality	34	Figure S7: Funnel-plot eligibility audit	64
S7. Effect selection and analysis routing	35	PRISMA 2020	65
S8. All pooled results	37	PRISMA 2020 for Abstracts	67
S9. Illustrative DGF absolute effects	38	PRISMA-S	68
S10. Non-estimable graft-failure absolute effects	39	SWiM	69
S11. Sensitivity-analysis completion	40	TRIPOD-SRMA	70
S12. Leave-one-out and influence summary	43	AMSTAR-PF self-audit	72
S13. Exactly matched clinical strata			

Table S1a. Search strategies as executed

The 28 strategies below are the exact V2.4.2 strings executed on each interface, reproduced verbatim from the frozen search authority; line breaks internal to a strategy are not significant and have been closed up. Streams A, B and C are the three purpose-built searches; for the registries the master query was the eligibility source and A, B and C were classification memberships.

Source	Stream	Query string as executed
PubMed/MEDLINE	A. Prognostic morphology	((("Kidney Transplantation"[Mesh] OR "kidney transplant*"[tiab] OR "renal transplant*"[tiab] OR "kidney allograft*"[tiab] OR "renal allograft*"[tiab] OR "kidney graft*"[tiab] OR "renal graft*"[tiab] OR "deceased donor kidney*"[tiab] OR "cadaveric kidney*"[tiab] OR "cadaver kidney*"[tiab] OR "renal homograft*"[tiab] OR "kidney transplant recipient*"[tiab] OR "renal transplant recipient*"[tiab] OR "donor kidney*"[tiab] OR "kidney donor*"[tiab] OR "recovered kidney*"[tiab] OR "donated kidney*"[tiab] OR "kidney recover*"[tiab] OR "kidney offer*"[tiab]) AND ("zero-time biops*"[tiab] OR "time-zero biops*"[tiab] OR "time zero biops*"[tiab] OR "zero-hour biops*"[tiab] OR "zero hour biops*"[tiab] OR "implantation biops*"[tiab] OR "pre-implantation biops*"[tiab] OR "preimplantation biops*"[tiab] OR "pre-implant biops*"[tiab] OR "preimplant biops*"[tiab] OR "procurement biops*"[tiab] OR "donor biops*"[tiab] OR "donor kidney biops*"[tiab] OR "kidney donor biops*"[tiab] OR "pretransplant biops*"[tiab] OR "pre-transplant biops*"[tiab] OR "baseline biops*"[tiab] OR "reperfusion biops*"[tiab] OR "post-reperfusion biops*"[tiab] OR "postreperfusion biops*"[tiab] OR "frozen section biops*"[tiab] OR "frozen-section biops*"[tiab] OR "wedge biops*"[tiab] OR "core biops*"[tiab] OR "needle biops*"[tiab] OR "tru-cut biops*"[tiab] OR ((biops*[tiab] OR histolog*[tiab] OR histopatholog*[tiab] OR patholog*[tiab] OR morpholog*[tiab] OR morphometr*[tiab] OR histomorphometr*[tiab] OR "frozen section"[tiab] OR glomerulosclero*[tiab] OR "glomerular sclerosis"[tiab] OR "interstitial fibrosis"[tiab] OR "tubular atrophy"[tiab] OR IFTA[tiab] OR arteriosclero*[tiab] OR arteriolosclero*[tiab] OR "arteriolar hyalinosis"[tiab] OR "fibrous intimal thickening"[tiab] OR "intimal fibrosis"[tiab] OR "wall to lumen"[tiab] OR "acute tubular injury"[tiab] OR "acute tubular necrosis"[tiab] OR microthromb*[tiab] OR Remuzzi[tiab] OR Karpinski[tiab] OR MAPI[tiab] OR CADI[tiab] OR Banff[tiab] OR "histological score*"[tiab] OR "pathology score*"[tiab] OR "biopsy score*"[tiab] OR "chronicity score*"[tiab]) AND (procurement[tiab] OR "donor kidney"[tiab] OR "kidney donor"[tiab] OR preimplant*[tiab] OR pretransplant*[tiab] OR "pre transplant*"[tiab] OR prereperfusion*[tiab] OR "pre reperfusion*"[tiab] OR preperfusion*[tiab] OR "pre perfusion*"[tiab] OR periimplant*[tiab] OR "peri implant*"[tiab] OR postexplant*[tiab] OR "post explant*"[tiab] OR "back table"[tiab] OR implantation[tiab] OR baseline[tiab] OR reperfusion[tiab] OR postreperfusion[tiab] OR "post reperfusion"[tiab] OR postperfusion[tiab] OR "post perfusion"[tiab] OR postrevascularization[tiab] OR "post revascularization"[tiab] OR time-zero[tiab] OR "time zero"[tiab] OR zero-time[tiab] OR "zero time"[tiab] OR zero-hour[tiab] OR "zero hour"[tiab] OR day-zero[tiab] OR one-hour[tiab] OR "one hour"[tiab] OR 1-hour[tiab] OR "1 hour"[tiab])))) OR ("Kidney Transplantation"[Mesh] AND ("Biopsy"[Mesh] OR "Kidney/pathology"[Mesh] OR biops*[ti]) AND ("Cadaver"[Mesh] OR "Tissue Donors"[Mesh] OR "Tissue and Organ Procurement"[Mesh])))
PubMed/MEDLINE	B. Incremental prediction	("Kidney Transplantation"[Mesh] OR "kidney transplant*"[tiab] OR "renal transplant*"[tiab] OR "kidney allograft*"[tiab] OR "renal allograft*"[tiab] OR "kidney graft*"[tiab] OR "renal graft*"[tiab] OR "deceased donor kidney*"[tiab] OR "cadaveric kidney*"[tiab] OR "cadaver kidney*"[tiab] OR "renal homograft*"[tiab] OR "kidney transplant recipient*"[tiab] OR "renal transplant recipient*"[tiab] OR "donor kidney*"[tiab] OR "kidney donor*"[tiab] OR "recovered kidney*"[tiab] OR "donated kidney*"[tiab] OR "kidney recover*"[tiab] OR "kidney offer*"[tiab]) AND (procurement[tiab] OR "donor kidney"[tiab] OR "kidney donor"[tiab] OR "donor biops*"[tiab] OR "donor histolog*"[tiab] OR "donor patholog*"[tiab] OR preimplant*[tiab] OR pretransplant*[tiab] OR "pre transplant*"[tiab] OR prereperfusion*[tiab] OR "pre reperfusion*"[tiab] OR periimplant*[tiab] OR "peri implant*"[tiab] OR postexplant*[tiab] OR "post explant*"[tiab] OR "back table"[tiab] OR implantation[tiab] OR baseline[tiab] OR reperfusion[tiab] OR postreperfusion[tiab] OR "post reperfusion"[tiab] OR time-zero[tiab] OR "time zero"[tiab] OR zero-hour[tiab] OR "zero hour"[tiab] OR day-zero[tiab] OR "day zero"[tiab]) AND (biops*[tiab] OR histolog*[tiab] OR histopatholog*[tiab] OR morphometr*[tiab] OR histomorphometr*[tiab] OR glomerulosclero*[tiab] OR "glomerular sclerosis"[tiab] OR "interstitial fibrosis"[tiab] OR "tubular atrophy"[tiab] OR IFTA[tiab] OR arteriosclero*[tiab] OR arteriolosclero*[tiab] OR "arteriolar hyalinosis"[tiab] OR "fibrous intimal thickening"[tiab] OR "intimal fibrosis"[tiab] OR "wall to lumen"[tiab] OR "acute tubular injury"[tiab] OR "acute tubular necrosis"[tiab] OR microthromb*[tiab] OR Remuzzi[tiab] OR Karpinski[tiab] OR MAPI[tiab] OR CADI[tiab] OR Banff[tiab] OR "histological score*"[tiab] OR "pathology score*"[tiab] OR "biopsy score*"[tiab] OR "chronicity score*"[tiab] OR "digital pathology"[tiab] OR "computational pathology"[tiab] OR "whole slide imag*"[tiab] OR "image analys*"[tiab]) AND (predict*[tiab] OR prognos*[tiab] OR model*[tiab] OR score*[tiab] OR validat*[tiab] OR discriminat*[tiab] OR calibrat*[tiab] OR ROC[tiab] OR AUC[tiab] OR AUROC[tiab] OR "C statistic"[tiab] OR C-statistic[tiab] OR "C index"[tiab] OR C-index[tiab] OR reclassif*[tiab] OR "decision curve"[tiab] OR nomogram*[tiab] OR increment*[tiab] OR "added value"[tiab] OR "incremental value"[tiab] OR "predictive performance"[tiab] OR "external validat*"[tiab] OR "internal validat*"[tiab] OR cross-validat*[tiab] OR "area under the curve"[tiab] OR "receiver operating characteristic*"[tiab] OR "calibration slope"[tiab] OR "calibration in the large"[tiab] OR Brier[tiab] OR "net benefit"[tiab] OR "net reclassification improvement"[tiab] OR NRI[tiab] OR "integrated discrimination improvement"[tiab] OR IDI[tiab] OR algorithm*[tiab] OR classifier*[tiab] OR "machine learning"[tiab] OR "deep learning"[tiab] OR "artificial intelligence"[tiab] OR "whole slide"[tiab] OR WSI[tiab] OR "convolutional neural network*"[tiab] OR CNN[tiab])
PubMed/MEDLINE	C. Availability, utilization or decision impact	("Kidney Transplantation"[Mesh] OR "kidney transplant*"[tiab] OR "renal transplant*"[tiab] OR "kidney allograft*"[tiab] OR "renal allograft*"[tiab] OR "kidney graft*"[tiab] OR "renal graft*"[tiab] OR "deceased donor kidney*"[tiab] OR "cadaveric kidney*"[tiab] OR "cadaver kidney*"[tiab] OR "renal homograft*"[tiab] OR "kidney transplant recipient*"[tiab] OR "renal transplant recipient*"[tiab] OR "donor kidney*"[tiab] OR "kidney donor*"[tiab] OR "recovered kidney*"[tiab] OR "donated kidney*"[tiab] OR "kidney recover*"[tiab] OR "kidney offer*"[tiab]) AND ("procurement biopsy"[tiab] OR "donor biopsy"[tiab] OR "donor kidney biopsy"[tiab] OR "preimplantation biopsy"[tiab] OR "pre-implantation biopsy"[tiab] OR "implantation biopsy"[tiab] OR "pretransplant biops*"[tiab] OR "pre-transplant biops*"[tiab] OR "time-zero biopsy"[tiab] OR "time zero biopsy"[tiab] OR "biopsy service"[tiab] OR "frozen section biops*"[tiab] OR "frozen-section biops*"[tiab] OR "wedge biops*"[tiab] OR "core biops*"[tiab] OR "needle biops*"[tiab] OR "tru-cut biops*"[tiab] OR ((biops*[tiab] OR histolog*[tiab] OR histopatholog*[tiab] OR patholog*[tiab] OR morpholog*[tiab] OR morphometr*[tiab] OR histomorphometr*[tiab] OR glomerulosclero*[tiab] OR "glomerular sclerosis"[tiab] OR "interstitial fibrosis"[tiab] OR "tubular atrophy"[tiab] OR IFTA[tiab] OR arteriosclero*[tiab] OR arteriolosclero*[tiab] OR "arteriolar hyalinosis"[tiab] OR "fibrous intimal thickening"[tiab] OR "intimal fibrosis"[tiab] OR "wall to lumen"[tiab] OR "acute tubular injury"[tiab] OR "acute tubular necrosis"[tiab] OR microthromb*[tiab] OR Remuzzi[tiab] OR Karpinski[tiab] OR MAPI[tiab] OR CADI[tiab] OR Banff[tiab] OR "histological score*"[tiab] OR "pathology score*"[tiab] OR "biopsy score*"[tiab] OR "chronicity score*"[tiab]) AND (procurement[tiab] OR donor[tiab] OR preimplant*[tiab] OR pretransplant*[tiab] OR prereperfusion*[tiab] OR "pre reperfusion*"[tiab] OR periimplant*[tiab] OR postexplant*[tiab] OR "back table"[tiab] OR implantation[tiab]))) AND (discard*[tiab] OR accept*[tiab] OR allocat*[tiab] OR utilization[tiab] OR utilisation[tiab] OR nonuse[tiab] OR "non-use"[tiab] OR nonutilization[tiab] OR non-utilisation[tiab] OR turndown*[tiab] OR "turn down"[tiab] OR declin*[tiab] OR refus*[tiab] OR "kidney yield"[tiab] OR "organ yield"[tiab] OR "organ offer*"[tiab] OR "organ placement"[tiab] OR "kidney placement"[tiab] OR "transplant rate*"[tiab] OR "acceptance rate*"[tiab] OR "discard rate*"[tiab] OR "biopsy use"[tiab] OR "biopsy practice*"[tiab] OR "biopsy rate*"[tiab] OR decision*[tiab] OR policy[tiab] OR biopsy-guided[tiab] OR "biopsy availability"[tiab] OR "biopsy service*"[tiab] OR disclos*[tiab] OR "biopsy result*"[tiab] OR "biopsy report*"[tiab] OR "histology report*"[tiab] OR "pathology report*"[tiab] OR communicat*[tiab] OR turnaround[tiab] OR telepatholog*[tiab] OR centraliz*[tiab] OR centralis*[tiab] OR "on call"[tiab] OR "24 hour"[tiab] OR "pathology coverage"[tiab] OR "single kidney"[tiab] OR "dual kidney"[tiab] OR implement*[tiab] OR evaluat*[tiab] OR impact*[tiab] OR random*[tiab] OR trial[tiab] OR "stepped wedge"[tiab] OR stepped-wedge[tiab] OR cluster*[tiab])
Embase.com	A. Prognostic morphology	((('kidney transplantation'/exp OR 'kidney transplant*':ti,ab,kw OR 'renal transplant*':ti,ab,kw OR 'kidney allograft*':ti,ab,kw OR 'renal allograft*':ti,ab,kw OR 'kidney graft*':ti,ab,kw OR 'renal graft*':ti,ab,kw OR 'deceased donor kidney*':ti,ab,kw OR 'cadaveric kidney*':ti,ab,kw OR 'cadaver kidney*':ti,ab,kw OR 'renal homograft*':ti,ab,kw OR 'kidney transplant recipient*':ti,ab,kw OR 'renal transplant recipient*':ti,ab,kw OR 'donor kidney*':ti,ab,kw OR 'kidney donor*':ti,ab,kw OR 'recovered kidney*':ti,ab,kw OR 'donated kidney*':ti,ab,kw OR 'kidney recover*':ti,ab,kw OR 'kidney offer*':ti,ab,kw) AND ('zero-time biopsy':ti,ab,kw OR 'time-zero biopsy':ti,ab,kw OR 'time zero biopsy':ti,ab,kw OR 'zero-hour biopsy':ti,ab,kw OR 'zero hour biopsy':ti,ab,kw OR 'day-zero biopsy':ti,ab,kw OR 'implantation biopsy':ti,ab,kw OR 'pre-implantation biopsy':ti,ab,kw OR 'preimplantation biopsy':ti,ab,kw OR 'pre-implant biopsy':ti,ab,kw OR 'preimplant biopsy':ti,ab,kw OR 'procurement biopsy':ti,ab,kw OR 'donor biopsy':ti,ab,kw OR 'donor kidney biopsy':ti,ab,kw OR 'kidney donor biopsy':ti,ab,kw OR 'pretransplant biops*':ti,ab,kw OR 'pre-transplant biops*':ti,ab,kw OR 'baseline biopsy':ti,ab,kw OR 'reperfusion biopsy':ti,ab,kw OR 'post-reperfusion biopsy':ti,ab,kw OR 'postreperfusion biopsy':ti,ab,kw OR 'frozen section biops*':ti,ab,kw OR 'frozen-section biops*':ti,ab,kw OR 'wedge biops*':ti,ab,kw OR 'core biops*':ti,ab,kw OR 'needle biops*':ti,ab,kw OR 'tru-cut biops*':ti,ab,kw OR ((biops*:ti,ab,kw OR histolog*:ti,ab,kw OR histopatholog*:ti,ab,kw OR patholog*:ti,ab,kw OR morpholog*:ti,ab,kw OR morphometr*:ti,ab,kw OR histomorphometr*:ti,ab,kw OR 'frozen section':ti,ab,kw OR glomerulosclero*:ti,ab,kw OR 'glomerular sclerosis':ti,ab,kw OR 'interstitial fibrosis':ti,ab,kw OR 'tubular atrophy':ti,ab,kw OR ifta:ti,ab,kw OR arteriosclero*:ti,ab,kw OR arteriolosclero*:ti,ab,kw OR 'arteriolar hyalinosis':ti,ab,kw OR 'fibrous intimal thickening':ti,ab,kw OR 'intimal fibrosis':ti,ab,kw OR 'wall to lumen':ti,ab,kw OR 'acute tubular injury':ti,ab,kw OR 'acute tubular necrosis':ti,ab,kw OR microthromb*:ti,ab,kw OR remuzzi:ti,ab,kw OR karpinski:ti,ab,kw OR mapi:ti,ab,kw OR cadi:ti,ab,kw OR banff:ti,ab,kw OR 'histological score*':ti,ab,kw OR 'pathology score*':ti,ab,kw OR 'biopsy score*':ti,ab,kw OR 'chronicity score*':ti,ab,kw) AND (procurement:ti,ab,kw OR 'donor kidney':ti,ab,kw OR 'kidney donor':ti,ab,kw OR preimplant*:ti,ab,kw OR pretransplant*:ti,ab,kw OR 'pre transplant*':ti,ab,kw OR prereperfusion*:ti,ab,kw OR 'pre reperfusion*':ti,ab,kw OR preperfusion*:ti,ab,kw OR 'pre perfusion*':ti,ab,kw OR periimplant*:ti,ab,kw OR 'peri implant*':ti,ab,kw OR postexplant*:ti,ab,kw OR 'post explant*':ti,ab,kw OR 'back table':ti,ab,kw OR implantation:ti,ab,kw OR baseline:ti,ab,kw OR reperfusion:ti,ab,kw OR postreperfusion:ti,ab,kw OR 'post reperfusion':ti,ab,kw OR postperfusion:ti,ab,kw OR 'post perfusion':ti,ab,kw OR postrevascularization:ti,ab,kw OR 'post revascularization':ti,ab,kw OR 'time-

Source	Stream	Query string as executed
		zero':ti,ab,kw OR 'time zero':ti,ab,kw OR 'zero-hour':ti,ab,kw OR 'zero hour':ti,ab,kw OR 'day-zero':ti,ab,kw OR 'one-hour':ti,ab,kw OR 'one hour':ti,ab,kw OR '1-hour':ti,ab,kw OR '1 hour':ti,ab,kw)))) OR (((kidney* OR renal) NEAR/10 ('deceased donor*' OR 'cadaveric donor*' OR 'brain dead donor*' OR 'brain-dead donor*' OR 'brain death donor*' OR 'donor* with brain death')):ti,ab,kw AND ('procurement biops*':ti,ab,kw OR 'pretransplant biops*':ti,ab,kw OR 'pre-transplant biops*':ti,ab,kw OR 'pre transplant biops*':ti,ab,kw OR 'donor biops*':ti,ab,kw OR 'preimplantation biops*':ti,ab,kw OR 'pre-implantation biops*':ti,ab,kw OR (biops*:ti,ab,kw AND (procurement:ti,ab,kw OR pretransplant*:ti,ab,kw OR 'pre transplant*':ti,ab,kw OR preimplant*:ti,ab,kw)))) OR (('zero-time':ti,ab,kw OR 'zero time':ti,ab,kw) AND biops*:ti,ab,kw AND (kidney*:ti,ab,kw OR renal:ti,ab,kw) AND (transplant*:ti,ab,kw OR allograft*:ti,ab,kw OR graft*:ti,ab,kw OR donor*:ti,ab,kw)))
Embase.com	B. Incremental prediction	('kidney transplantation'/exp OR 'kidney transplant*':ti,ab,kw OR 'renal transplant*':ti,ab,kw OR 'kidney allograft*':ti,ab,kw OR 'renal allograft*':ti,ab,kw OR 'kidney graft*':ti,ab,kw OR 'renal graft*':ti,ab,kw OR 'deceased donor kidney*':ti,ab,kw OR 'cadaveric kidney*':ti,ab,kw OR 'cadaver kidney*':ti,ab,kw OR 'renal homograft*':ti,ab,kw OR 'kidney transplant recipient*':ti,ab,kw OR 'renal transplant recipient*':ti,ab,kw OR 'donor kidney*':ti,ab,kw OR 'kidney donor*':ti,ab,kw OR 'recovered kidney*':ti,ab,kw OR 'donated kidney*':ti,ab,kw OR 'kidney recover*':ti,ab,kw OR 'kidney offer*':ti,ab,kw) AND (procurement:ti,ab,kw OR 'donor kidney':ti,ab,kw OR 'kidney donor':ti,ab,kw OR 'donor biops*':ti,ab,kw OR 'donor histolog*':ti,ab,kw OR 'donor patholog*':ti,ab,kw OR preimplant*:ti,ab,kw OR pretransplant*:ti,ab,kw OR 'pre transplant*':ti,ab,kw OR prereperfusion*:ti,ab,kw OR 'pre reperfusion*':ti,ab,kw OR periimplant*:ti,ab,kw OR 'peri implant*':ti,ab,kw OR postexplant*:ti,ab,kw OR 'post explant*':ti,ab,kw OR 'back table':ti,ab,kw OR implantation:ti,ab,kw OR baseline:ti,ab,kw OR reperfusion:ti,ab,kw OR postreperfusion:ti,ab,kw OR 'post reperfusion':ti,ab,kw OR 'time-zero':ti,ab,kw OR 'time zero':ti,ab,kw OR 'zero-hour':ti,ab,kw OR 'zero hour':ti,ab,kw OR 'day-zero':ti,ab,kw OR 'day zero':ti,ab,kw) AND (biops*:ti,ab,kw OR histolog*:ti,ab,kw OR histopatholog*:ti,ab,kw OR morphometr*:ti,ab,kw OR histomorphometr*:ti,ab,kw OR glomerulosclero*:ti,ab,kw OR 'glomerular sclerosis':ti,ab,kw OR 'interstitial fibrosis':ti,ab,kw OR 'tubular atrophy':ti,ab,kw OR ifta:ti,ab,kw OR arteriosclero*:ti,ab,kw OR arteriolosclero*:ti,ab,kw OR 'arteriolar hyalinosis':ti,ab,kw OR 'fibrous intimal thickening':ti,ab,kw OR 'intimal fibrosis':ti,ab,kw OR 'wall to lumen':ti,ab,kw OR 'acute tubular injury':ti,ab,kw OR 'acute tubular necrosis':ti,ab,kw OR microthromb*:ti,ab,kw OR remuzzi:ti,ab,kw OR karpinski:ti,ab,kw OR mapi:ti,ab,kw OR cadi:ti,ab,kw OR banff:ti,ab,kw OR 'histological score*':ti,ab,kw OR 'pathology score*':ti,ab,kw OR 'biopsy score*':ti,ab,kw OR 'chronicity score*':ti,ab,kw OR 'digital pathology':ti,ab,kw OR 'computational pathology':ti,ab,kw OR 'whole slide imag*':ti,ab,kw OR 'image analys*':ti,ab,kw) AND (predict*:ti,ab,kw OR prognos*:ti,ab,kw OR model*:ti,ab,kw OR score*:ti,ab,kw OR validat*:ti,ab,kw OR discriminat*:ti,ab,kw OR calibrat*:ti,ab,kw OR roc:ti,ab,kw OR auc:ti,ab,kw OR auroc:ti,ab,kw OR 'C statistic':ti,ab,kw OR 'C-statistic':ti,ab,kw OR 'C index':ti,ab,kw OR 'C-index':ti,ab,kw OR reclassif*:ti,ab,kw OR 'decision curve':ti,ab,kw OR nomogram*:ti,ab,kw OR increment*:ti,ab,kw OR 'added value':ti,ab,kw OR 'incremental value':ti,ab,kw OR 'predictive performance':ti,ab,kw OR 'external validat*':ti,ab,kw OR 'internal validat*':ti,ab,kw OR cross-validat*:ti,ab,kw OR 'area under the curve':ti,ab,kw OR 'receiver operating characteristic*':ti,ab,kw OR 'calibration slope':ti,ab,kw OR 'calibration in the large':ti,ab,kw OR brier:ti,ab,kw OR 'net benefit':ti,ab,kw OR 'net reclassification improvement':ti,ab,kw OR nri:ti,ab,kw OR 'integrated discrimination improvement':ti,ab,kw OR idi:ti,ab,kw OR algorithm*:ti,ab,kw OR classifier*:ti,ab,kw OR 'machine learning':ti,ab,kw OR 'deep learning':ti,ab,kw OR 'artificial intelligence':ti,ab,kw OR 'whole slide':ti,ab,kw OR wsi:ti,ab,kw OR 'convolutional neural network*':ti,ab,kw OR cnn:ti,ab,kw)
Embase.com	C. Availability, utilization or decision impact	('kidney transplantation'/exp OR 'kidney transplant*':ti,ab,kw OR 'renal transplant*':ti,ab,kw OR 'kidney allograft*':ti,ab,kw OR 'renal allograft*':ti,ab,kw OR 'kidney graft*':ti,ab,kw OR 'renal graft*':ti,ab,kw OR 'deceased donor kidney*':ti,ab,kw OR 'cadaveric kidney*':ti,ab,kw OR 'cadaver kidney*':ti,ab,kw OR 'renal homograft*':ti,ab,kw OR 'kidney transplant recipient*':ti,ab,kw OR 'renal transplant recipient*':ti,ab,kw OR 'donor kidney*':ti,ab,kw OR 'kidney donor*':ti,ab,kw OR 'recovered kidney*':ti,ab,kw OR 'donated kidney*':ti,ab,kw OR 'kidney recover*':ti,ab,kw OR 'kidney offer*':ti,ab,kw) AND ('procurement biopsy':ti,ab,kw OR 'donor biopsy':ti,ab,kw OR 'donor kidney biopsy':ti,ab,kw OR 'preimplantation biopsy':ti,ab,kw OR 'pre-implantation biopsy':ti,ab,kw OR 'implantation biopsy':ti,ab,kw OR 'pretransplant biops*':ti,ab,kw OR 'pre-transplant biops*':ti,ab,kw OR 'time-zero biopsy':ti,ab,kw OR 'time zero biopsy':ti,ab,kw OR 'biopsy service':ti,ab,kw OR 'frozen section biops*':ti,ab,kw OR 'frozen-section biops*':ti,ab,kw OR 'wedge biops*':ti,ab,kw OR 'core biops*':ti,ab,kw OR 'needle biops*':ti,ab,kw OR 'tru-cut biops*':ti,ab,kw OR ((biops*:ti,ab,kw OR histolog*:ti,ab,kw OR histopatholog*:ti,ab,kw OR patholog*:ti,ab,kw OR morpholog*:ti,ab,kw OR morphometr*:ti,ab,kw OR histomorphometr*:ti,ab,kw OR glomerulosclero*:ti,ab,kw OR 'glomerular sclerosis':ti,ab,kw OR 'interstitial fibrosis':ti,ab,kw OR 'tubular atrophy':ti,ab,kw OR ifta:ti,ab,kw OR arteriosclero*:ti,ab,kw OR arteriolosclero*:ti,ab,kw OR 'arteriolar hyalinosis':ti,ab,kw OR 'fibrous intimal thickening':ti,ab,kw OR 'intimal fibrosis':ti,ab,kw OR 'wall to lumen':ti,ab,kw OR 'acute tubular injury':ti,ab,kw OR 'acute tubular necrosis':ti,ab,kw OR microthromb*:ti,ab,kw OR remuzzi:ti,ab,kw OR karpinski:ti,ab,kw OR mapi:ti,ab,kw OR cadi:ti,ab,kw OR banff:ti,ab,kw OR 'histological score*':ti,ab,kw OR 'pathology score*':ti,ab,kw OR 'biopsy score*':ti,ab,kw OR 'chronicity score*':ti,ab,kw) AND (procurement:ti,ab,kw OR donor:ti,ab,kw OR preimplant*:ti,ab,kw OR pretransplant*:ti,ab,kw OR prereperfusion*:ti,ab,kw OR 'pre reperfusion*':ti,ab,kw OR periimplant*:ti,ab,kw OR postexplant*:ti,ab,kw OR 'back table':ti,ab,kw OR implantation:ti,ab,kw))) AND (discard*:ti,ab,kw OR accept*:ti,ab,kw OR allocat*:ti,ab,kw OR utilization:ti,ab,kw OR utilisation:ti,ab,kw OR nonuse:ti,ab,kw OR 'non-use':ti,ab,kw OR nonutilization:ti,ab,kw OR non-utilisation:ti,ab,kw OR turndown*:ti,ab,kw OR 'turn down':ti,ab,kw OR declin*:ti,ab,kw OR refus*:ti,ab,kw OR 'kidney yield':ti,ab,kw OR 'organ yield':ti,ab,kw OR 'organ offer*':ti,ab,kw OR 'organ placement':ti,ab,kw OR 'kidney placement':ti,ab,kw OR 'transplant rate*':ti,ab,kw OR 'acceptance rate*':ti,ab,kw OR 'discard rate*':ti,ab,kw OR 'biopsy use':ti,ab,kw OR 'biopsy practice*':ti,ab,kw OR 'biopsy rate*':ti,ab,kw OR decision*:ti,ab,kw OR policy:ti,ab,kw OR 'biopsy-guided':ti,ab,kw OR 'biopsy availability':ti,ab,kw OR 'biopsy service*':ti,ab,kw OR disclos*:ti,ab,kw OR 'biopsy result*':ti,ab,kw OR 'biopsy report*':ti,ab,kw OR 'histology report*':ti,ab,kw OR 'pathology report*':ti,ab,kw OR communicat*:ti,ab,kw OR turnaround:ti,ab,kw OR telepatholog*:ti,ab,kw OR centraliz*:ti,ab,kw OR centralis*:ti,ab,kw OR 'on call':ti,ab,kw OR '24 hour':ti,ab,kw OR 'pathology coverage':ti,ab,kw OR 'single kidney':ti,ab,kw OR 'dual kidney':ti,ab,kw OR implement*:ti,ab,kw OR evaluat*:ti,ab,kw OR impact*:ti,ab,kw OR random*:ti,ab,kw OR trial:ti,ab,kw OR 'stepped wedge':ti,ab,kw OR 'stepped-wedge':ti,ab,kw OR cluster*:ti,ab,kw)
Scopus	A. Prognostic morphology	TITLE-ABS-KEY(("kidney transplant*" OR "renal transplant*" OR "kidney allograft*" OR "renal allograft*" OR "kidney graft*" OR "renal graft*" OR "deceased donor kidney*" OR "cadaveric kidney*" OR "cadaver kidney*" OR "renal homograft*" OR "kidney transplant recipient*" OR "renal transplant recipient*" OR "donor kidney*" OR "kidney donor*" OR "recovered kidney*" OR "donated kidney*" OR "kidney recover*" OR "kidney offer*") AND ("zero-time biopsy" OR "time-zero biopsy" OR "time zero biopsy" OR "zero-hour biopsy" OR "zero hour biopsy" OR "day-zero biopsy" OR "implantation biopsy" OR "pre-implantation biopsy" OR "preimplantation biopsy" OR "pre-implant biopsy" OR "preimplant biopsy" OR "procurement biopsy" OR "donor biopsy" OR "donor kidney biopsy" OR "kidney donor biopsy" OR "pretransplant biops*" OR "pre-transplant biops*" OR "baseline biopsy" OR "reperfusion biopsy" OR "post-reperfusion biopsy" OR "postreperfusion biopsy" OR "frozen section biops*" OR "frozen-section biops*" OR "wedge biops*" OR "core biops*" OR "needle biops*" OR "tru-cut biops*" OR ((biops* OR histolog* OR histopatholog* OR patholog* OR morpholog* OR morphometr* OR histomorphometr* OR "frozen section" OR glomerulosclero* OR "glomerular sclerosis" OR "interstitial fibrosis" OR "tubular atrophy" OR IFTA OR arteriosclero* OR arteriolosclero* OR "arteriolar hyalinosis" OR "fibrous intimal thickening" OR "intimal fibrosis" OR "wall to lumen" OR "acute tubular injury" OR "acute tubular necrosis" OR microthromb* OR Remuzzi OR Karpinski OR MAPI OR CADI OR Banff OR "histological score*" OR "pathology score*" OR "biopsy score*" OR "chronicity score*") AND (procurement OR "donor kidney" OR "kidney donor" OR preimplant* OR pretransplant* OR "pre transplant*" OR prereperfusion* OR "pre reperfusion*" OR preperfusion* OR "pre perfusion*" OR periimplant* OR "peri implant*" OR postexplant* OR "post explant*" OR "back table" OR implantation OR baseline OR reperfusion OR postreperfusion OR "post reperfusion" OR postperfusion OR "post perfusion" OR postrevascularization OR "post revascularization" OR "time-zero" OR "time zero" OR "zero-time" OR "zero time" OR "zero-hour" OR "zero hour" OR "day-zero" OR "one-hour" OR "one hour" OR "1-hour" OR "1 hour"))))
Scopus	B. Incremental prediction	TITLE-ABS-KEY(("kidney transplant*" OR "renal transplant*" OR "kidney allograft*" OR "renal allograft*" OR "kidney graft*" OR "renal graft*" OR "deceased donor kidney*" OR "cadaveric kidney*" OR "cadaver kidney*" OR "renal homograft*" OR "kidney transplant recipient*" OR "renal transplant recipient*" OR "donor kidney*" OR "kidney donor*" OR "recovered kidney*" OR "donated kidney*" OR "kidney recover*" OR "kidney offer*") AND (procurement OR "donor kidney" OR "kidney donor" OR "donor biops*" OR "donor histolog*" OR "donor patholog*" OR preimplant* OR pretransplant* OR "pre transplant*" OR prereperfusion* OR "pre reperfusion*" OR periimplant* OR "peri implant*" OR postexplant* OR "post explant*" OR "back table" OR implantation OR baseline OR reperfusion OR postreperfusion OR "post reperfusion" OR "time-zero" OR "time zero" OR "zero-hour" OR "zero hour" OR "day-zero" OR "day zero") AND (biops* OR histolog* OR histopatholog* OR morphometr* OR histomorphometr* OR glomerulosclero* OR "glomerular sclerosis" OR "interstitial fibrosis" OR "tubular atrophy" OR IFTA OR arteriosclero* OR arteriolosclero* OR "arteriolar hyalinosis" OR "fibrous intimal thickening" OR "intimal fibrosis" OR "wall to lumen" OR "acute tubular injury" OR "acute tubular necrosis" OR microthromb* OR Remuzzi OR Karpinski OR MAPI OR CADI OR Banff OR "histological score*" OR "pathology score*" OR "biopsy score*" OR "chronicity score*" OR "digital pathology" OR "computational pathology" OR "whole slide imag*" OR "image analys*") AND (predict* OR prognos* OR model* OR score* OR validat* OR discriminat* OR calibrat* OR ROC OR AUC OR AUROC OR "C statistic" OR "C-statistic" OR "C index" OR "C-index" OR reclassif* OR "decision curve" OR nomogram* OR increment* OR "added value" OR "incremental value" OR "predictive performance" OR "external validat*" OR "internal validat*" OR cross-validat* OR "area under the curve" OR "receiver operating characteristic*" OR "calibration slope" OR "calibration in the large" OR Brier OR "net benefit" OR "net reclassification improvement" OR NRI OR "integrated discrimination improvement" OR IDI OR algorithm* OR classifier* OR "machine learning" OR "deep learning" OR "artificial intelligence" OR "whole slide" OR WSI OR "convolutional neural network*" OR CNN))
Scopus	C. Availability, utilization or decision impact	TITLE-ABS-KEY(("kidney transplant*" OR "renal transplant*" OR "kidney allograft*" OR "renal allograft*" OR "kidney graft*" OR "renal graft*" OR "deceased donor kidney*" OR "cadaveric kidney*" OR "cadaver kidney*" OR "renal homograft*" OR "kidney transplant recipient*" OR "renal transplant recipient*" OR "donor kidney*" OR "kidney donor*" OR "recovered kidney*" OR "donated kidney*" OR "kidney recover*" OR

Source	Stream	Query string as executed
		"kidney offer*") AND ("procurement biopsy" OR "donor biopsy" OR "donor kidney biopsy" OR "preimplantation biopsy" OR "pre-implantation biopsy" OR "implantation biopsy" OR "pretransplant biops*" OR "pre-transplant biops*" OR "time-zero biopsy" OR "time zero biopsy" OR "biopsy service" OR "frozen section biops*" OR "frozen-section biops*" OR "wedge biops*" OR "core biops*" OR "needle biops*" OR "tru-cut biops*" OR ((biops* OR histolog* OR histopatholog* OR patholog* OR morpholog* OR morphometr* OR histomorphometr* OR glomerulosclero* OR "glomerular sclerosis" OR "interstitial fibrosis" OR "tubular atrophy" OR IFTA OR arteriosclero* OR arteriolosclero* OR "arteriolar hyalinosis" OR "fibrous intimal thickening" OR "intimal fibrosis" OR "wall to lumen" OR "acute tubular injury" OR "acute tubular necrosis" OR microthromb* OR Remuzzi OR Karpinski OR MAPI OR CADI OR Banff OR "histological score*" OR "pathology score*" OR "biopsy score*" OR "chronicity score*") AND (procurement OR donor OR preimplant* OR pretransplant* OR prereperfusion* OR "pre reperfusion*" OR periimplant* OR postexplant* OR "back table" OR implantation))) AND (discard* OR accept* OR allocat* OR utilization OR utilisation OR nonuse OR "non-use" OR nonutilization OR non-utilisation OR turndown* OR "turn down" OR declin* OR refus* OR "kidney yield" OR "organ yield" OR "organ offer*" OR "organ placement" OR "kidney placement" OR "transplant rate*" OR "acceptance rate*" OR "discard rate*" OR "biopsy use" OR "biopsy practice*" OR "biopsy rate*" OR decision* OR policy OR "biopsy-guided" OR "biopsy availability" OR "biopsy service*" OR disclos* OR "biopsy result*" OR "biopsy report*" OR "histology report*" OR "pathology report*" OR communicat* OR turnaround OR telepatholog* OR centraliz* OR centralis* OR "on call" OR "24 hour" OR "pathology coverage" OR "single kidney" OR "dual kidney" OR implement* OR evaluat* OR impact* OR random* OR trial OR "stepped wedge" OR "stepped-wedge" OR cluster*))
Web of Science Core Collection	A. Prognostic morphology	# Documentation recipe for Web of Science Core Collection Query Builder. Enter each TS= expression without its #n label; Query Builder assigns the history-set numbers. #1 TS=("kidney transplant*" OR "renal transplant*" OR "kidney allograft*" OR "renal allograft*" OR "kidney graft*" OR "renal graft*" OR "deceased donor kidney*" OR "cadaveric kidney*" OR "cadaver kidney*" OR "renal homograft*" OR "kidney transplant recipient*" OR "renal transplant recipient*" OR "donor kidney*" OR "kidney donor*" OR "recovered kidney*" OR "donated kidney*" OR "kidney recover*" OR "kidney offer*") #2 TS=("zero-time biopsy" OR "time-zero biopsy" OR "time zero biopsy" OR "zero-hour biopsy" OR "zero hour biopsy" OR "day-zero biopsy" OR "implantation biopsy" OR "pre-implantation biopsy" OR "preimplantation biopsy" OR "pre-implant biopsy" OR "preimplant biopsy" OR "procurement biopsy" OR "donor biopsy" OR "donor kidney biopsy" OR "kidney donor biopsy" OR "pretransplant biops*" OR "pre-transplant biops*" OR "baseline biopsy" OR "reperfusion biopsy" OR "post-reperfusion biopsy" OR "postreperfusion biopsy" OR "frozen section biops*" OR "frozen-section biops*" OR "wedge biops*" OR "core biops*" OR "needle biops*" OR "tru-cut biops*") #3 TS=(biops* OR histolog* OR histopatholog* OR patholog* OR morpholog* OR morphometr* OR histomorphometr* OR "frozen section" OR glomerulosclero* OR "glomerular sclerosis" OR "interstitial fibrosis" OR "tubular atrophy" OR IFTA OR arteriosclero* OR arteriolosclero* OR "arteriolar hyalinosis" OR "fibrous intimal thickening" OR "intimal fibrosis" OR "wall to lumen" OR "acute tubular injury" OR "acute tubular necrosis" OR microthromb* OR Remuzzi OR Karpinski OR MAPI OR CADI OR Banff OR "histological score*" OR "pathology score*" OR "biopsy score*" OR "chronicity score*") #4 TS=(procurement OR "donor kidney" OR "kidney donor" OR preimplant* OR pretransplant* OR "pre transplant*" OR prereperfusion* OR "pre reperfusion*" OR preperfusion* OR "pre perfusion*" OR periimplant* OR "peri implant*" OR postexplant* OR "post explant*" OR "back table" OR implantation OR baseline OR reperfusion OR postreperfusion OR "post reperfusion" OR postperfusion OR "post perfusion" OR postrevascularization OR "post revascularization" OR "time-zero" OR "time zero" OR "zero-time" OR "zero time" OR "zero-hour" OR "zero hour" OR "day-zero" OR "one-hour" OR "one hour" OR "1-hour" OR "1 hour") #5 #1 AND (#2 OR (#3 AND #4))
Web of Science Core Collection	B. Incremental prediction	# Documentation recipe for Web of Science Core Collection Query Builder. Enter each TS= expression without its #n label; Query Builder assigns the history-set numbers. #1 TS=("kidney transplant*" OR "renal transplant*" OR "kidney allograft*" OR "renal allograft*" OR "kidney graft*" OR "renal graft*" OR "deceased donor kidney*" OR "cadaveric kidney*" OR "cadaver kidney*" OR "renal homograft*" OR "kidney transplant recipient*" OR "renal transplant recipient*" OR "donor kidney*" OR "kidney donor*" OR "recovered kidney*" OR "donated kidney*" OR "kidney recover*" OR "kidney offer*") #2 TS=(procurement OR "donor kidney" OR "kidney donor" OR "donor biops*" OR "donor histolog*" OR "donor patholog*" OR preimplant* OR pretransplant* OR "pre transplant*" OR prereperfusion* OR "pre reperfusion*" OR periimplant* OR "peri implant*" OR postexplant* OR "post explant*" OR "back table" OR implantation OR baseline OR reperfusion OR postreperfusion OR "post reperfusion" OR "time-zero" OR "time zero" OR "zero-hour" OR "zero hour" OR "day-zero" OR "day zero") #3 TS=(biops* OR histolog* OR histopatholog* OR morphometr* OR histomorphometr* OR glomerulosclero* OR "glomerular sclerosis" OR "interstitial fibrosis" OR "tubular atrophy" OR IFTA OR arteriosclero* OR arteriolosclero* OR "arteriolar hyalinosis" OR "fibrous intimal thickening" OR "intimal fibrosis" OR "wall to lumen" OR "acute tubular injury" OR "acute tubular necrosis" OR microthromb* OR Remuzzi OR Karpinski OR MAPI OR CADI OR Banff OR "histological score*" OR "pathology score*" OR "biopsy score*" OR "chronicity score*" OR "digital pathology" OR "computational pathology" OR "whole slide imag*" OR "image analys*") #4 TS=(predict* OR prognos* OR model* OR score* OR validat* OR discriminat* OR calibrat* OR ROC OR AUC OR AUROC OR "C statistic" OR "C-statistic" OR "C index" OR "C-index" OR reclassif* OR "decision curve" OR nomogram* OR increment* OR "added value" OR "incremental value" OR "predictive performance" OR "external validat*" OR "internal validat*" OR cross-validat* OR "area under the curve" OR "receiver operating characteristic*" OR "calibration slope" OR "calibration in the large" OR Brier OR "net benefit" OR "net reclassification improvement" OR NRI OR "integrated discrimination improvement" OR IDI OR algorithm* OR classifier* OR "machine learning" OR "deep learning" OR "artificial intelligence" OR "whole slide" OR WSI OR "convolutional neural network*" OR CNN) #5 #1 AND #2 AND #3 AND #4
Web of Science Core Collection	C. Availability, utilization or decision impact	# Documentation recipe for Web of Science Core Collection Query Builder. Enter each TS= expression without its #n label; Query Builder assigns the history-set numbers. #1 TS=("kidney transplant*" OR "renal transplant*" OR "kidney allograft*" OR "renal allograft*" OR "kidney graft*" OR "renal graft*" OR "deceased donor kidney*" OR "cadaveric kidney*" OR "cadaver kidney*" OR "renal homograft*" OR "kidney transplant recipient*" OR "renal transplant recipient*" OR "donor kidney*" OR "kidney donor*" OR "recovered kidney*" OR "donated kidney*" OR "kidney recover*" OR "kidney offer*") #2 TS=("procurement biopsy" OR "donor biopsy" OR "donor kidney biopsy" OR "preimplantation biopsy" OR "pre-implantation biopsy" OR "implantation biopsy" OR "pretransplant biops*" OR "pre-transplant biops*" OR "time-zero biopsy" OR "time zero biopsy" OR "biopsy service" OR "frozen section biops*" OR "frozen-section biops*" OR "wedge biops*" OR "core biops*" OR "needle biops*" OR "tru-cut biops*") #3 TS=(biops* OR histolog* OR histopatholog* OR patholog* OR morpholog* OR morphometr* OR histomorphometr* OR glomerulosclero* OR "glomerular sclerosis" OR "interstitial fibrosis" OR "tubular atrophy" OR IFTA OR arteriosclero* OR arteriolosclero* OR "arteriolar hyalinosis" OR "fibrous intimal thickening" OR "intimal fibrosis" OR "wall to lumen" OR "acute tubular injury" OR "acute tubular necrosis" OR microthromb* OR Remuzzi OR Karpinski OR MAPI OR CADI OR Banff OR "histological score*" OR "pathology score*" OR "biopsy score*" OR "chronicity score*") #4 TS=(procurement OR donor OR preimplant* OR pretransplant* OR prereperfusion* OR "pre reperfusion*" OR periimplant* OR postexplant* OR "back table" OR implantation) #5 TS=(discard* OR accept* OR allocat* OR utilization OR utilisation OR nonuse OR "non-use" OR nonutilization OR non-utilisation OR turndown* OR "turn down" OR declin* OR refus* OR "kidney yield" OR "organ yield" OR "organ offer*" OR "organ placement" OR "kidney placement" OR "transplant rate*" OR "acceptance rate*" OR "discard rate*" OR "biopsy use" OR "biopsy practice*" OR "biopsy rate*" OR decision* OR policy OR "biopsy-guided" OR "biopsy availability" OR "biopsy service*" OR disclos* OR "biopsy result*" OR "biopsy report*" OR "histology report*" OR "pathology report*" OR communicat* OR turnaround OR telepatholog* OR centraliz* OR centralis* OR "on call" OR "24 hour" OR "pathology coverage" OR "single kidney" OR "dual kidney" OR implement* OR evaluat* OR impact* OR random* OR trial OR "stepped wedge" OR "stepped-wedge" OR cluster*) #6 #1 AND (#2 OR (#3 AND #4)) AND #5
CENTRAL	A. Prognostic morphology	#1 [mh "Kidney Transplantation"] OR ((kidney NEXT transplant*) OR (renal NEXT transplant*) OR (kidney NEXT allograft*) OR (renal NEXT allograft*) OR (kidney NEXT graft*) OR (renal NEXT graft*) OR (deceased NEXT donor NEXT kidney*) OR (cadaveric NEXT kidney*) OR (cadaver NEXT kidney*) OR (renal NEXT homograft*) OR (kidney NEXT transplant NEXT recipient*) OR (renal NEXT transplant NEXT recipient*) OR (donor NEXT kidney*) OR (kidney NEXT donor*) OR (recovered NEXT kidney*) OR (donated NEXT kidney*) OR (kidney NEXT recover*) OR (kidney NEXT offer*)):ti,ab,kw #2 ("zero-time biopsy" OR "time-zero biopsy" OR "time zero biopsy" OR "zero-hour biopsy" OR "zero hour biopsy" OR "day-zero biopsy" OR "implantation biopsy" OR "pre-implantation biopsy" OR "preimplantation biopsy" OR "pre-implant biopsy" OR "preimplant biopsy" OR "procurement biopsy" OR "donor biopsy" OR "donor kidney biopsy" OR "kidney donor biopsy" OR (pretransplant NEXT biops*) OR (pre-transplant NEXT biops*) OR "baseline biopsy" OR "reperfusion biopsy" OR "post-reperfusion biopsy" OR "postreperfusion biopsy" OR (frozen NEXT section NEXT biops*) OR (frozen-section NEXT biops*) OR (wedge NEXT biops*) OR (core NEXT biops*) OR (needle NEXT biops*) OR (tru-cut NEXT biops*)):ti,ab,kw #3 (biops* OR histolog* OR histopatholog* OR patholog* OR morpholog* OR morphometr* OR histomorphometr* OR "frozen section" OR glomerulosclero* OR "glomerular sclerosis" OR "interstitial fibrosis" OR "tubular atrophy" OR IFTA OR arteriosclero* OR arteriolosclero* OR "arteriolar hyalinosis" OR "fibrous intimal thickening" OR "intimal fibrosis" OR "wall to lumen" OR "acute tubular injury" OR "acute tubular necrosis" OR microthromb* OR Remuzzi OR Karpinski OR MAPI OR CADI OR Banff OR (histological NEXT score*) OR (pathology NEXT score*) OR (biopsy NEXT score*) OR (chronicity NEXT score*)):ti,ab,kw #4 (procurement OR "donor kidney" OR "kidney donor" OR preimplant* OR pretransplant* OR (pre NEXT transplant*) OR prereperfusion* OR (pre NEXT reperfusion*) OR preperfusion* OR (pre NEXT perfusion*) OR periimplant* OR (peri NEXT implant*) OR postexplant* OR (post NEXT explant*) OR "back table" OR implantation OR baseline OR reperfusion OR postreperfusion OR "post reperfusion" OR postperfusion OR "post perfusion" OR postrevascularization OR "post revascularization" OR "time-zero" OR "time zero" OR "zero-time" OR "zero time" OR "zero-hour" OR "zero hour" OR "day-zero" OR "one-hour" OR "one hour" OR "1-hour" OR "1 hour"):ti,ab,kw #5 (#1 AND (#2 OR (#3 AND #4))) in Trials
CENTRAL	B. Incremental prediction	#1 [mh "Kidney Transplantation"] OR ((kidney NEXT transplant*) OR (renal NEXT transplant*) OR (kidney NEXT allograft*) OR (renal NEXT allograft*) OR (kidney NEXT graft*) OR (renal NEXT graft*) OR (deceased NEXT donor NEXT kidney*) OR (cadaveric NEXT kidney*) OR (cadaver NEXT kidney*) OR (renal NEXT homograft*) OR (kidney NEXT transplant NEXT recipient*) OR (renal NEXT transplant NEXT recipient*) OR (donor NEXT kidney*) OR (kidney NEXT donor*) OR (recovered NEXT kidney*) OR (donated NEXT kidney*) OR (kidney NEXT recover*) OR (kidney NEXT offer*)):ti,ab,kw #2 (procurement OR "donor kidney" OR

Source	Stream	Query string as executed
		"kidney donor" OR (donor NEXT biops*) OR (donor NEXT histolog*) OR (donor NEXT patholog*) OR preimplant* OR pretransplant* OR (pre NEXT transplant*) OR prereperfusion* OR (pre NEXT reperfusion*) OR periimplant* OR (peri NEXT implant*) OR postexplant* OR (post NEXT explant*) OR "back table" OR implantation OR baseline OR reperfusion OR postreperfusion OR "post reperfusion" OR "time-zero" OR "time zero" OR "zero-hour" OR "zero hour" OR "day-zero" OR "day zero"):ti,ab,kw #3 (biops* OR histolog* OR histopatholog* OR morphometr* OR histomorphometr* OR glomerulosclero* OR "glomerular sclerosis" OR "interstitial fibrosis" OR "tubular atrophy" OR IFTA OR arteriosclero* OR arteriolosclero* OR "arteriolar hyalinosis" OR "fibrous intimal thickening" OR "intimal fibrosis" OR "wall to lumen" OR "acute tubular injury" OR "acute tubular necrosis" OR microthromb* OR Remuzzi OR Karpinski OR MAPI OR CADI OR Banff OR (histological NEXT score*) OR (pathology NEXT score*) OR (biopsy NEXT score*) OR (chronicity NEXT score*) OR "digital pathology" OR "computational pathology" OR (whole NEXT slide NEXT imag*) OR (image NEXT analys*)):ti,ab,kw #4 (predict* OR prognos* OR model* OR score* OR validat* OR discriminat* OR calibrat* OR ROC OR AUC OR AUROC OR "C statistic" OR "C-statistic" OR "C index" OR "C-index" OR reclassif* OR "decision curve" OR nomogram* OR increment* OR "added value" OR "incremental value" OR "predictive performance" OR (external NEXT validat*) OR (internal NEXT validat*) OR cross-validat* OR "area under the curve" OR (receiver NEXT operating NEXT characteristic*) OR "calibration slope" OR "calibration in the large" OR Brier OR "net benefit" OR "net reclassification improvement" OR NRI OR "integrated discrimination improvement" OR IDI OR algorithm* OR classifier* OR "machine learning" OR "deep learning" OR "artificial intelligence" OR "whole slide" OR WSI OR (convolutional NEXT neural NEXT network*) OR CNN):ti,ab,kw #5 (#1 AND #2 AND #3 AND #4) in Trials
CENTRAL	C. Availability, utilization or decision impact	#1 [mh "Kidney Transplantation"] OR ((kidney NEXT transplant*) OR (renal NEXT transplant*) OR (kidney NEXT allograft*) OR (renal NEXT allograft*) OR (kidney NEXT graft*) OR (renal NEXT graft*) OR (deceased NEXT donor NEXT kidney*) OR (cadaveric NEXT kidney*) OR (cadaver NEXT kidney*) OR (renal NEXT homograft*) OR (kidney NEXT transplant NEXT recipient*) OR (renal NEXT transplant NEXT recipient*) OR (donor NEXT kidney*) OR (kidney NEXT donor*) OR (recovered NEXT kidney*) OR (donated NEXT kidney*) OR (kidney NEXT recover*) OR (kidney NEXT offer*)):ti,ab,kw #2 ("procurement biopsy" OR "donor biopsy" OR "donor kidney biopsy" OR "preimplantation biopsy" OR "pre-implantation biopsy" OR "implantation biopsy" OR (pretransplant NEXT biops*) OR (pre-transplant NEXT biops*) OR "time-zero biopsy" OR "time zero biopsy" OR "biopsy service" OR (frozen NEXT section NEXT biops*) OR (frozen-section NEXT biops*) OR (wedge NEXT biops*) OR (core NEXT biops*) OR (needle NEXT biops*) OR (tru-cut NEXT biops*)):ti,ab,kw #3 (biops* OR histolog* OR histopatholog* OR patholog* OR morpholog* OR morphometr* OR histomorphometr* OR glomerulosclero* OR "glomerular sclerosis" OR "interstitial fibrosis" OR "tubular atrophy" OR IFTA OR arteriosclero* OR arteriolosclero* OR "arteriolar hyalinosis" OR "fibrous intimal thickening" OR "intimal fibrosis" OR "wall to lumen" OR "acute tubular injury" OR "acute tubular necrosis" OR microthromb* OR Remuzzi OR Karpinski OR MAPI OR CADI OR Banff OR (histological NEXT score*) OR (pathology NEXT score*) OR (biopsy NEXT score*) OR (chronicity NEXT score*)):ti,ab,kw #4 (procurement OR donor OR preimplant* OR pretransplant* OR prereperfusion* OR (pre NEXT reperfusion*) OR periimplant* OR postexplant* OR "back table" OR implantation):ti,ab,kw #5 (discard* OR accept* OR allocat* OR utilization OR utilisation OR nonuse OR "non-use" OR nonutilization OR non-utilisation OR turndown* OR "turn down" OR declin* OR refus* OR "kidney yield" OR "organ yield" OR (organ NEXT offer*) OR "organ placement" OR "kidney placement" OR (transplant NEXT rate*) OR (acceptance NEXT rate*) OR (discard NEXT rate*) OR "biopsy use" OR (biopsy NEXT practice*) OR (biopsy NEXT rate*) OR decision* OR policy OR "biopsy-guided" OR "biopsy availability" OR (biopsy NEXT service*) OR disclos* OR (biopsy NEXT result*) OR (biopsy NEXT report*) OR (histology NEXT report*) OR (pathology NEXT report*) OR communicat* OR turnaround OR telepatholog* OR centraliz* OR centralis* OR "on call" OR "24 hour" OR "pathology coverage" OR "single kidney" OR "dual kidney" OR implement* OR evaluat* OR impact* OR random* OR trial OR "stepped wedge" OR "stepped-wedge" OR cluster*):ti,ab,kw #6 (#1 AND (#2 OR (#3 AND #4)) AND #5) in Trials
ClinicalTrials.gov	A. Prognostic morphology	AREA[BasicSearch]EXPANSION[Term]("kidney transplant" OR "kidney transplants" OR "kidney transplantation" OR "renal transplant" OR "renal transplants" OR "renal transplantation" OR "kidney graft" OR "kidney grafts" OR "renal graft" OR "renal grafts" OR "kidney allograft" OR "kidney allografts" OR "renal allograft" OR "renal allografts" OR "donor kidney" OR "donor kidneys" OR "kidney donor" OR "kidney donors" OR "recovered kidney" OR "recovered kidneys" OR "donated kidney" OR "donated kidneys") AND AREA[BasicSearch]EXPANSION[Term](biopsy OR biopsies OR histology OR histologic OR histological OR histopathology OR histopathologic OR histopathological OR pathology OR pathologic OR pathological OR morphology OR morphologic OR morphological OR microscopy OR microscopic OR "frozen section" OR "frozen sections" OR "permanent section" OR "permanent sections" OR "digital pathology" OR telepathology) AND AREA[BasicSearch]EXPANSION[Term]("deceased donor" OR "deceased donors" OR procurement OR preimplantation OR "pre-implantation" OR prereperfusion OR "pre-reperfusion" OR implantation OR reperfusion OR postreperfusion OR "post-reperfusion" OR "back table" OR donor OR donors OR "organ offer" OR "organ offers")
ClinicalTrials.gov	B. Incremental prediction	AREA[BasicSearch]EXPANSION[Term]("kidney transplant" OR "kidney transplants" OR "kidney transplantation" OR "renal transplant" OR "renal transplants" OR "renal transplantation" OR "kidney graft" OR "kidney grafts" OR "renal graft" OR "renal grafts" OR "kidney allograft" OR "kidney allografts" OR "renal allograft" OR "renal allografts" OR "donor kidney" OR "donor kidneys" OR "kidney donor" OR "kidney donors" OR "recovered kidney" OR "recovered kidneys" OR "donated kidney" OR "donated kidneys") AND AREA[BasicSearch]EXPANSION[Term](biopsy OR biopsies OR histology OR histologic OR histological OR histopathology OR histopathologic OR histopathological OR pathology OR pathologic OR pathological OR morphology OR morphologic OR morphological OR microscopy OR microscopic OR "frozen section" OR "frozen sections" OR "permanent section" OR "permanent sections" OR "digital pathology" OR telepathology) AND AREA[BasicSearch]EXPANSION[Term](prediction OR prognostic OR model OR validation OR discrimination OR calibration OR ROC OR AUC OR AUROC OR "C statistic" OR "C-statistic" OR "machine learning" OR "deep learning" OR "artificial intelligence" OR "decision curve" OR "net benefit")
ClinicalTrials.gov	C. Availability, utilization or decision impact	AREA[BasicSearch]EXPANSION[Term]("kidney transplant" OR "kidney transplants" OR "kidney transplantation" OR "renal transplant" OR "renal transplants" OR "renal transplantation" OR "kidney graft" OR "kidney grafts" OR "renal graft" OR "renal grafts" OR "kidney allograft" OR "kidney allografts" OR "renal allograft" OR "renal allografts" OR "donor kidney" OR "donor kidneys" OR "kidney donor" OR "kidney donors" OR "recovered kidney" OR "recovered kidneys" OR "donated kidney" OR "donated kidneys") AND AREA[BasicSearch]EXPANSION[Term](biopsy OR biopsies OR histology OR histologic OR histological OR histopathology OR histopathologic OR histopathological OR pathology OR pathologic OR pathological OR morphology OR morphologic OR morphological OR microscopy OR microscopic OR "frozen section" OR "frozen sections" OR "permanent section" OR "permanent sections" OR "digital pathology" OR telepathology) AND AREA[BasicSearch]EXPANSION[Term](allocation OR acceptance OR discard OR utilization OR utilisation OR nonuse OR "non-use" OR "organ offer" OR "organ offers" OR "biopsy service" OR "digital pathology service" OR "kidney yield" OR "organ yield" OR "transplant rate" OR disclosure OR communication OR "biopsy result" OR "biopsy results" OR "biopsy report" OR "biopsy reports" OR "histology report" OR "histology reports" OR "pathology report" OR "pathology reports" OR "single kidney" OR "dual kidney")
ClinicalTrials.gov	Master (registry eligibility source)	AREA[BasicSearch]EXPANSION[Term]("kidney transplant" OR "kidney transplants" OR "kidney transplantation" OR "renal transplant" OR "renal transplants" OR "renal transplantation" OR "kidney graft" OR "kidney grafts" OR "renal graft" OR "renal grafts" OR "kidney allograft" OR "kidney allografts" OR "renal allograft" OR "renal allografts" OR "donor kidney" OR "donor kidneys" OR "kidney donor" OR "kidney donors" OR "recovered kidney" OR "recovered kidneys" OR "donated kidney" OR "donated kidneys") AND AREA[BasicSearch]EXPANSION[Term](biopsy OR biopsies OR histology OR histologic OR histological OR histopathology OR histopathologic OR histopathological OR pathology OR pathologic OR pathological OR morphology OR morphologic OR morphological OR microscopy OR microscopic OR "frozen section" OR "frozen sections" OR "permanent section" OR "permanent sections" OR "digital pathology" OR telepathology)
ISRCTN	A. Prognostic morphology	("kidney transplant" OR "kidney transplants" OR "kidney transplantation" OR "renal transplant" OR "renal transplants" OR "renal transplantation" OR "kidney graft" OR "kidney grafts" OR "renal graft" OR "renal grafts" OR "kidney allograft" OR "kidney allografts" OR "renal allograft" OR "renal allografts" OR "donor kidney" OR "donor kidneys" OR "kidney donor" OR "kidney donors" OR "recovered kidney" OR "recovered kidneys" OR "donated kidney" OR "donated kidneys") AND (biopsy OR biopsies OR histology OR histologic OR histological OR histopathology OR histopathologic OR histopathological OR pathology OR pathologic OR pathological OR morphology OR morphologic OR morphological OR microscopy OR microscopic OR "frozen section" OR "frozen sections" OR "permanent section" OR "permanent sections" OR "digital pathology" OR telepathology) AND ("deceased donor" OR "deceased donors" OR procurement OR preimplantation OR "pre-implantation" OR prereperfusion OR "pre-reperfusion" OR implantation OR reperfusion OR postreperfusion OR "post-reperfusion" OR "back table" OR donor OR donors OR "organ offer" OR "organ offers")
ISRCTN	B. Incremental prediction	("kidney transplant" OR "kidney transplants" OR "kidney transplantation" OR "renal transplant" OR "renal transplants" OR "renal transplantation" OR "kidney graft" OR "kidney grafts" OR "renal graft" OR "renal grafts" OR "kidney allograft" OR "kidney allografts" OR "renal allograft" OR "renal allografts" OR "donor kidney" OR "donor kidneys" OR "kidney donor" OR "kidney donors" OR "recovered kidney" OR "recovered kidneys" OR "donated kidney" OR "donated kidneys") AND (biopsy OR biopsies OR histology OR histologic OR histological OR histopathology OR histopathologic OR histopathological OR pathology OR pathologic OR pathological OR morphology OR morphologic OR morphological OR microscopy OR microscopic OR "frozen section" OR "frozen sections" OR "permanent section" OR "permanent sections" OR "digital pathology" OR telepathology) AND (prediction OR predictions OR prognostic OR model OR models OR validation OR validations OR discrimination OR calibration OR calibrations OR ROC OR AUC OR AUROC OR "C statistic" OR "C statistics" OR "C-statistic" OR "C-statistics" OR "machine learning" OR "deep learning" OR "artificial intelligence" OR "decision curve" OR "decision curves" OR "net benefit" OR "net benefits")
ISRCTN	C. Availability, utilization or decision	("kidney transplant" OR "kidney transplants" OR "kidney transplantation" OR "renal transplant" OR "renal transplants" OR "renal transplantation" OR "kidney graft" OR "kidney grafts" OR "renal graft" OR "renal grafts"

Source	Stream	Query string as executed
	impact	OR "kidney allograft" OR "kidney allografts" OR "renal allograft" OR "renal allografts" OR "donor kidney" OR "donor kidneys" OR "kidney donor" OR "kidney donors" OR "recovered kidney" OR "recovered kidneys" OR "donated kidney" OR "donated kidneys") AND (biopsy OR biopsies OR histology OR histologic OR histological OR histopathology OR histopathologic OR histopathological OR pathology OR pathologic OR pathological OR morphology OR morphologic OR morphological OR microscopy OR microscopic OR "frozen section" OR "frozen sections" OR "permanent section" OR "permanent sections" OR "digital pathology" OR telepathology) AND (allocation OR allocations OR acceptance OR acceptances OR discard OR discards OR utilization OR utilizations OR utilisation OR utilisations OR nonuse OR "non-use" OR "organ offer" OR "organ offers" OR "biopsy service" OR "biopsy services" OR "digital pathology service" OR "digital pathology services" OR "kidney yield" OR "kidney yields" OR "organ yield" OR "organ yields" OR "transplant rate" OR "transplant rates" OR disclosure OR disclosures OR communication OR communications OR "biopsy result" OR "biopsy results" OR "biopsy report" OR "biopsy reports" OR "histology report" OR "histology reports" OR "pathology report" OR "pathology reports" OR "single kidney" OR "single kidneys" OR "dual kidney" OR "dual kidneys")
ISRCTN	Master (registry eligibility source)	("kidney transplant" OR "kidney transplants" OR "kidney transplantation" OR "renal transplant" OR "renal transplants" OR "renal transplantation" OR "kidney graft" OR "kidney grafts" OR "renal graft" OR "renal grafts" OR "kidney allograft" OR "kidney allografts" OR "renal allograft" OR "renal allografts" OR "donor kidney" OR "donor kidneys" OR "kidney donor" OR "kidney donors" OR "recovered kidney" OR "recovered kidneys" OR "donated kidney" OR "donated kidneys") AND (biopsy OR biopsies OR histology OR histologic OR histological OR histopathology OR histopathologic OR histopathological OR pathology OR pathologic OR pathological OR morphology OR morphologic OR morphological OR microscopy OR microscopic OR "frozen section" OR "frozen sections" OR "permanent section" OR "permanent sections" OR "digital pathology" OR telepathology)
WHO ICTRP	A. Prognostic morphology	("kidney transplant" OR "kidney transplants" OR "kidney transplantation" OR "renal transplant" OR "renal transplants" OR "renal transplantation" OR "kidney graft" OR "kidney grafts" OR "renal graft" OR "renal grafts" OR "kidney allograft" OR "kidney allografts" OR "renal allograft" OR "renal allografts" OR "donor kidney" OR "donor kidneys" OR "kidney donor" OR "kidney donors" OR "recovered kidney" OR "recovered kidneys" OR "donated kidney" OR "donated kidneys") AND (biopsy OR biopsies OR histology OR histologic OR histological OR histopathology OR histopathologic OR histopathological OR pathology OR pathologic OR pathological OR morphology OR morphologic OR morphological OR microscopy OR microscopic OR "frozen section" OR "frozen sections" OR "permanent section" OR "permanent sections" OR "digital pathology" OR telepathology) AND ("deceased donor" OR "deceased donors" OR procurement OR preimplantation OR "pre-implantation" OR prereperfusion OR "pre-reperfusion" OR implantation OR reperfusion OR postreperfusion OR "post-reperfusion" OR "back table" OR donor OR donors OR "organ offer" OR "organ offers")
WHO ICTRP	B. Incremental prediction	("kidney transplant" OR "kidney transplants" OR "kidney transplantation" OR "renal transplant" OR "renal transplants" OR "renal transplantation" OR "kidney graft" OR "kidney grafts" OR "renal graft" OR "renal grafts" OR "kidney allograft" OR "kidney allografts" OR "renal allograft" OR "renal allografts" OR "donor kidney" OR "donor kidneys" OR "kidney donor" OR "kidney donors" OR "recovered kidney" OR "recovered kidneys" OR "donated kidney" OR "donated kidneys") AND (biopsy OR biopsies OR histology OR histologic OR histological OR histopathology OR histopathologic OR histopathological OR pathology OR pathologic OR pathological OR morphology OR morphologic OR morphological OR microscopy OR microscopic OR "frozen section" OR "frozen sections" OR "permanent section" OR "permanent sections" OR "digital pathology" OR telepathology) AND (prediction OR prognostic OR model OR validation OR discrimination OR calibration OR ROC OR AUC OR AUROC OR "C statistic" OR "C-statistic" OR "machine learning" OR "deep learning" OR "artificial intelligence" OR "decision curve" OR "net benefit")
WHO ICTRP	C. Availability, utilization or decision impact	("kidney transplant" OR "kidney transplants" OR "kidney transplantation" OR "renal transplant" OR "renal transplants" OR "renal transplantation" OR "kidney graft" OR "kidney grafts" OR "renal graft" OR "renal grafts" OR "kidney allograft" OR "kidney allografts" OR "renal allograft" OR "renal allografts" OR "donor kidney" OR "donor kidneys" OR "kidney donor" OR "kidney donors" OR "recovered kidney" OR "recovered kidneys" OR "donated kidney" OR "donated kidneys") AND (biopsy OR biopsies OR histology OR histologic OR histological OR histopathology OR histopathologic OR histopathological OR pathology OR pathologic OR pathological OR morphology OR morphologic OR morphological OR microscopy OR microscopic OR "frozen section" OR "frozen sections" OR "permanent section" OR "permanent sections" OR "digital pathology" OR telepathology)
WHO ICTRP	C, prioritization only	("kidney transplant" OR "kidney transplants" OR "kidney transplantation" OR "renal transplant" OR "renal transplants" OR "renal transplantation" OR "kidney graft" OR "kidney grafts" OR "renal graft" OR "renal grafts" OR "kidney allograft" OR "kidney allografts" OR "renal allograft" OR "renal allografts" OR "donor kidney" OR "donor kidneys" OR "kidney donor" OR "kidney donors" OR "recovered kidney" OR "recovered kidneys" OR "donated kidney" OR "donated kidneys") AND (biopsy OR biopsies OR histology OR histologic OR histological OR histopathology OR histopathologic OR histopathological OR pathology OR pathologic OR pathological OR morphology OR morphologic OR morphological OR microscopy OR microscopic OR "frozen section" OR "frozen sections" OR "permanent section" OR "permanent sections" OR "digital pathology" OR telepathology) AND (allocation OR acceptance OR discard OR utilization OR utilisation OR nonuse OR "non-use" OR "organ offer" OR "organ offers" OR service OR availability OR implementation OR impact OR trial OR disclosure OR communication OR "biopsy result" OR "biopsy results" OR "biopsy report" OR "biopsy reports" OR "histology report" OR "histology reports" OR "pathology report" OR "pathology reports")
WHO ICTRP	Master (registry eligibility source)	("kidney transplant" OR "kidney transplants" OR "kidney transplantation" OR "renal transplant" OR "renal transplants" OR "renal transplantation" OR "kidney graft" OR "kidney grafts" OR "renal graft" OR "renal grafts" OR "kidney allograft" OR "kidney allografts" OR "renal allograft" OR "renal allografts" OR "donor kidney" OR "donor kidneys" OR "kidney donor" OR "kidney donors" OR "recovered kidney" OR "recovered kidneys" OR "donated kidney" OR "donated kidneys") AND (biopsy OR biopsies OR histology OR histologic OR histological OR histopathology OR histopathologic OR histopathological OR pathology OR pathologic OR pathological OR morphology OR morphologic OR morphological OR microscopy OR microscopic OR "frozen section" OR "frozen sections" OR "permanent section" OR "permanent sections" OR "digital pathology" OR telepathology)

Abbreviations and symbols: AND/OR, Boolean search operators; AREA/EXPANSION, ClinicalTrials.gov query directives; AUC, area under the receiver operating characteristic curve; AUROC, area under the receiver operating characteristic curve; CADI, Chronic Allograft Damage Index; CENTRAL, Cochrane Central Register of Controlled Trials; CNN, convolutional neural network; de/mh, subject-heading search fields; exp, exploded subject heading; IDI, integrated discrimination improvement; IFTA, interstitial fibrosis and tubular atrophy; ISRCTN, International Standard Randomised Controlled Trial Number registry; MAPI, Maryland Aggregate Pathology Index; MEDLINE, Medical Literature Analysis and Retrieval System Online; MeSH, Medical Subject Headings; NEAR/NEXT, database-specific proximity operators; NRI, net reclassification improvement; PRESS, Peer Review of Electronic Search Strategies; ROC, receiver operating characteristic; ti/ab/kw, title, abstract and keyword fields; tiab, title and abstract search field; TITLE-ABS-KEY, title, abstract and keyword search fields; TS, Topic search field; WHO ICTRP, World Health Organization International Clinical Trials Registry Platform; WSI, whole-slide image.

Searches ran from inception to 25 July 2026 with no date or language restriction; CENTRAL was restricted to its Trials source set, Embase.com used the combined Embase and MEDLINE scope, and no sidebar, status, phase, study-type or results filters were applied. The PRESS 2015-guided review and response are detailed in Supplementary Table S1c.

Table S1b. Final source execution

Source execution summary

Source	Source-native records	Stream A	Stream B	Stream C
PubMed/MEDLINE	7,053	5,703	1,766	3,286
Embase.com	15,838	11,799	4,452	8,999
Scopus	12,260	9,678	3,486	6,491
Web of Science Core Collection	7,190	5,120	2,028	4,099
CENTRAL	1,312	906	277	938
ClinicalTrials.gov	1,818 master records	661	269	131
ISRCTN	75 master records	45	14	19
WHO ICTRP	130 master records	Classification memberships retained in the archive	Classification memberships retained in the archive	Classification memberships retained in the archive

Abbreviations and symbols: CENTRAL, Cochrane Central Register of Controlled Trials; ISRCTN, International Standard Randomised Controlled Trial Number registry; MEDLINE, Medical Literature Analysis and Retrieval System Online; WHO ICTRP, World Health Organization International Clinical Trials Registry Platform.

Stream totals exceed source-native totals because a record could satisfy more than one stream. For registries, the master query was the sole eligibility source and A/B/C were classification memberships.

Table S1c. PRESS 2015-guided peer-review assessment, response and final disposition

An external reviewer assessed the primary PubMed strategies against all six PRESS 2015 domains and additionally examined database translations and registry logic. The initial V2.4.1 assessment required revisions; the final column records the disposition after the response and reconfirmation cycle.

PRESS domain and initial A/B/C rating	Main reviewer finding	Response and evidence	Final disposition
Translation of the question into searchable concepts (SUG/OK/OK)	Consider adding standalone donor[tiab] to Stream A.	Not adopted. After the accepted donor biops* repair, the term added 2,950 nonspecific marginal records without closing an additional identified retrieval gap.	Resolved; no query change.
Boolean, proximity, nesting and set logic (SUG/OK/OK)	Consider propagating the Embase proximity rescue across platforms.	Retained as source-specific because syntax and wildcard behavior differed across platforms; the rationale and benchmark recovery were documented.	Resolved; documented.
Subject headings, explosion, focus and subheadings (REQ/SUG/SUG)	Replace four [Majr] restrictions with [Mesh] in PubMed A; consider a broader Emtree rescue.	The four tags were replaced with [Mesh] (836 added, 0 lost). The broad Emtree rescue was not adopted after highly nonspecific retrieval testing.	Required revision implemented and confirmed.
Text words, variants, truncation, acronyms and fields (REQ/SUG/SUG)	Truncate fixed biopsy phrases; add zero-time/"zero time" and preperfusion variants.	Implemented source-specifically in V2.4.2. Isolated PubMed gains were +8/–0 for truncation, +5/–0 for zero-time, and +4/–0 specific to preperfusion; the combined repaired query gained 851 and lost 0 because the sets overlap. The zero-time propagation reruns yielded Scopus +2/–0, Web of Science +9/–0 and CENTRAL +0/–0.	Required revisions implemented and confirmed.
Spelling, syntax, line numbers and set construction (SUG/SUG/SUG)	Preserve translation and phrase-warning evidence.	Raw tests were retained and produced no phrase-index warnings; no additional query change was required.	Resolved; evidence deposited.
Limits and filters (OK/OK/OK)	No concern identified.	No change. Searches retained inception coverage without date or language restrictions.	No revision.

Abbreviations and symbols: A, prognostic morphology search; B, incremental prediction search; C, availability, utilization or decision-impact search; CENTRAL, Cochrane Central Register of Controlled Trials; Majr, major-topic Medical Subject Headings field; MD, Doctor of Medicine; MeSH, Medical Subject Headings; MSc, Master of Science; OK, no revision; ORCID, Open Researcher and Contributor ID; PRESS, Peer Review of Electronic Search Strategies; REQ, required revision; SUG, suggested revision; tiab, title and abstract search field.

A, prognostic morphology; B, incremental prediction; C, availability, utilization or decision impact. OK, no revision; SUG, suggested revision; REQ, required revision. The initial V2.4.1 assessment was completed on 24 July 2026. Required revisions were incorporated into V2.4.2 and their implementation was confirmed by the reviewer on 25 July 2026 before final execution. Reviewer: Pedro Martínez-Ayala, MD, MSc; ORCID 0000-0002-0505-6340; pedro.martinez@cucs.udg.mx; Antiguo Hospital Civil de Guadalajara “Fray Antonio Alcalde”, Guadalajara 44280, Jalisco, Mexico; and División Salud, Centro Universitario de Tlajomulco, Universidad de Guadalajara, Tlajomulco de Zuñiga 45641, Mexico. He was independent of strategy development and execution, was not a manuscript author, declared no conflict, personally verified the strategies, and reported five years of search-review experience or training.

Table S2. Characteristics of included reports and their contribution to the synthesis

All 225 included reports are cited in the main article; Ref. is the number printed in that reference list. Suffixes a, b and c distinguish separate reports sharing the same first author and publication year. The study-unit role distinguishes reports that add an independent study/cohort from linked companion reports. Descriptors reproduce fields available in the retained analytic extractions and are not inferred from titles; an unavailable report-level descriptor does not mean that the original publication omitted it. Analysis areas identify pooled and other retained contributions. P, prognostic association; I, incremental prediction; U, clinical utility; M, measurement.

Ref.	First author, year	Study-unit role	Study descriptors from retained extraction	Domains	Synthesis route	Analysis area(s) contributed
103	Adani, 2019	Eligible report; adds one study investigation and one cohort family	No report-level descriptive fields in the retained analytic extraction	P	Other eligible evidence	Domain evidence without a retained prognostic effect
124	Alechinsky, 2021	Eligible report; adds one study investigation and one cohort family	No report-level descriptive fields in the retained analytic extraction	P	Other eligible evidence	Domain evidence without a retained prognostic effect
118	Aleš Rigler, 2020	Eligible report; adds one study investigation and one cohort family	No report-level descriptive fields in the retained analytic extraction	P	Other eligible evidence	Domain evidence without a retained prognostic effect
42	Alfrey, 2003	Eligible report; adds one study investigation and one cohort family	No report-level descriptive fields in the retained analytic extraction	P	Other eligible evidence	Domain evidence without a retained prognostic effect
89	Amenábar, 2016	Eligible report; adds one study investigation and one cohort family	Enrollment: 2008-03 to 2012-05; Analytic n: 110; Biopsy: technique Wedge; processing Paraffin	P	Selected; displayed, not pooled	eGFR x chronic composite score; low eGFR x chronic composite score
155	Androvitsanea, 2025	Eligible report; adds one study investigation and one cohort family	No report-level descriptive fields in the retained analytic extraction	P	Other eligible evidence	Domain evidence without a retained prognostic effect
165	Anglicheau, 2008	Eligible report; adds one study investigation and one cohort family	No report-level descriptive fields in the retained analytic extraction	P, I	Other eligible evidence	Domain evidence without a retained prognostic effect
195	Arias, 2006	Eligible report; adds one study investigation and one cohort family	No report-level descriptive fields in the retained analytic extraction	P, M	Other eligible evidence	Domain evidence without a retained prognostic effect
52	Arias, 2007	Eligible report; adds one study investigation and one cohort family	No report-level descriptive fields in the retained analytic extraction	P	Other eligible evidence	Domain evidence without a retained prognostic effect
74	Assis-Borba, 2014	Eligible report; adds one study investigation and one cohort family	Analytic n: 372	P	Selected; displayed, not pooled	all-cause graft failure x Remuzzi score; graft failure x Remuzzi score
171	Aubert, 2015	Eligible report; adds one study investigation and one cohort family	No report-level descriptive fields in the retained analytic extraction	P, I	Other eligible evidence	Domain evidence without a retained prognostic effect
90	Auglienė, 2017	Eligible report; adds one study investigation and one cohort family	No report-level descriptive fields in the retained analytic extraction	P	Other eligible evidence	Domain evidence without a retained prognostic effect
247	Ayorinde, 2024	Eligible companion/overlapping report; adds no independent population	Cohort: Metrology reanalysis of repeat biopsies; Context: No eligible clinical prognostic ratio.	M	Other eligible evidence	Measurement: Measurement uncertainty in renal preimplantation biopsy assessment
13	Azancot, 2014	Eligible report; adds one study investigation and one cohort family	Analytic n: 117	P, I	Selected; displayed, not pooled	DGF x chronic composite score; acute rejection x chronic composite score
40	Badid, 2002	Eligible report; adds one study investigation and one cohort family	No report-level descriptive fields in the retained analytic extraction	P	Other eligible evidence	Domain evidence without a retained prognostic effect
51	Bajwa, 2007	Eligible report; adds one study investigation and one cohort family	Country: United States; Setting: OPTN/UNOS national database; analysis by the University of California, Los Angeles and the University of Southern California (Bajwa et al.); Enrollment: 2000-01-01 to 2005-12-31; Analytic n: 12129	P	Selected; displayed, not pooled	all-cause graft failure x glomerulosclerosis
101	Batal, 2018	Eligible companion/overlapping report; adds no independent population	No report-level descriptive fields in the retained analytic extraction	P	Other eligible evidence	Domain evidence without a retained prognostic effect
85	Batra, 2016	Eligible companion/overlapping report; adds no independent population	No report-level descriptive fields in the retained analytic extraction	P	Other eligible evidence	Domain evidence without a retained prognostic effect
256	Bikbov, 2021	Contextual evidence unit; excluded from the primary prognostic meta-analysis	No report-level descriptive fields in the retained analytic extraction	–	Contextual evidence only	Contextual evidence only
99	Bissolati, 2018	Eligible companion/overlapping report; adds no independent population	No report-level descriptive fields in the retained analytic extraction	P	Other eligible evidence	Domain evidence without a retained prognostic effect
104	Bissolati, 2019	Eligible report; adds one study investigation and one cohort family	No report-level descriptive fields in the retained analytic extraction	P	Other eligible evidence	Domain evidence without a retained prognostic effect
122	Bissolati, 2021	Eligible report; adds one study investigation and one cohort family	Design: Nonrandomized decision-utility cohort; Dual-kidney group: Reported in source family; Single/reference group: Reported in source family	P	Other eligible evidence	Clinical utility: DGF and pod30 renal function

Ref.	First author, year	Study-unit role	Study descriptors from retained extraction	Domains	Synthesis route	Analysis area(s) contributed
91	Bontha, 2017	Eligible report; adds one study investigation and one cohort family	No report-level descriptive fields in the retained analytic extraction	P	Other eligible evidence	Domain evidence without a retained prognostic effect
14	Carpenter, 2018	Eligible companion/overlapping report; adds no independent population	No report-level descriptive fields in the retained analytic extraction	P	Other eligible evidence	Domain evidence without a retained prognostic effect
129	Carrara, 2021	Eligible report; adds one study investigation and one cohort family	No report-level descriptive fields in the retained analytic extraction	P	Other eligible evidence	Domain evidence without a retained prognostic effect
53	Carroll, 2008	Eligible report; adds one study investigation and one cohort family	No report-level descriptive fields in the retained analytic extraction	P	Other eligible evidence	Domain evidence without a retained prognostic effect
71	Carta, 2013	Eligible report; adds one study investigation and one cohort family	Analytic n: 100	P	Selected; displayed, not pooled	acute rejection x Karpinski/MAPI; all-cause graft failure x Karpinski/MAPI; graft failure x Karpinski/MAPI
95	Casati, 2018	Eligible companion/overlapping report; adds no independent population	No report-level descriptive fields in the retained analytic extraction	P	Other eligible evidence	Domain evidence without a retained prognostic effect
102	Centellas-Pérez, 2019	Eligible report; adds one study investigation and one cohort family	Enrollment: 2011 to 2015; Analytic n: 183; Biopsy: processing varied	P	Selected; displayed, not pooled	DGF x Karpinski/MAPI; all-cause graft failure x Karpinski/MAPI; all-cause graft failure x chronic composite score; primary nonfunction x chronic composite score
177	Chen, 2019	Eligible companion/overlapping report; adds no independent population	No report-level descriptive fields in the retained analytic extraction	P, I	Other eligible evidence	Domain evidence without a retained prognostic effect
179	Chen, 2021	Eligible report; adds one study investigation and one cohort family	Enrollment: 2012 to 2014; Effect-specific analytic n; see Table S3; Biopsy: technique varied; processing varied	P, I	Pooled meta-analysis	Pooled: GS-GRAFT FAILURE; IFTA-GRAFT FAILURE; REMUZZI-DGF; TUBULAR ATROPHY-GRAFT FAILURE; VASCULAR-GRAFT FAILURE; selected/other: DGF x Karpinski/MAPI; DGF x Leuven score; DGF x Remuzzi score; DGF x chronic composite score; graft failure x IFTA; +3 more (see domain table)
149	Chen, 2024	Eligible report; adds one study investigation and one cohort family	No report-level descriptive fields in the retained analytic extraction	P	Other eligible evidence	Domain evidence without a retained prognostic effect
120	Cheungpasitporn, 2020	Eligible report; adds one study investigation within a linked cohort family	Country: United States; Setting: US OPTN/UNOS national registry; Enrollment: 2005-2014; Analytic n: 1730	P	Pooled meta-analysis	Pooled: GS-DGF; GS-GRAFT FAILURE; selected/other: DGF x glomerulosclerosis; acute rejection x glomerulosclerosis; graft failure x glomerulosclerosis
254	Cima, 2019	Contextual evidence unit; excluded from the primary prognostic meta-analysis	No report-level descriptive fields in the retained analytic extraction	–	Contextual evidence only	Contextual evidence only
203	Collini, 2006	Eligible report; adds one study investigation and one cohort family	No report-level descriptive fields in the retained analytic extraction	P, U	Other eligible evidence	Domain evidence without a retained prognostic effect
94	Colussi, 2018	Eligible report; adds one study investigation and one cohort family	No report-level descriptive fields in the retained analytic extraction	P	Other eligible evidence	Domain evidence without a retained prognostic effect
162	Comai, 2026	Eligible report; adds one study investigation and one cohort family	No report-level descriptive fields in the retained analytic extraction	P	Other eligible evidence	Domain evidence without a retained prognostic effect
168	Cravedi, 2010	Eligible report; adds one study investigation and one cohort family	No report-level descriptive fields in the retained analytic extraction	P, I	Other eligible evidence	Domain evidence without a retained prognostic effect
64	Cruzado, 2011	Eligible report; adds one study investigation and one cohort family	No report-level descriptive fields in the retained analytic extraction	P	Other eligible evidence	Domain evidence without a retained prognostic effect
223	D'Arcy, 2009	Eligible report; adds one study investigation and one cohort family	Design: Single-center comparative cohort; abstract/full-report family; Dual-kidney group: 24; Single/reference group: 44	U	Other eligible evidence	Clinical utility: DGF/atn, graft function and 36-month survival
229	De Paolis, 2016	Eligible report; adds one study investigation and one cohort family	Design: Retrospective single-center cohort; Dual-kidney group: 32; Single/reference group: 67	U	Other eligible evidence	Clinical utility: DGF, graft function, graft and patient survival
164	De Vusser, 2013	Contextual evidence unit; excluded from the primary prognostic meta-analysis	No report-level descriptive fields in the retained analytic extraction	P, I	Contextual evidence only	Contextual evidence only
100	Del Moral Martín, 2018	Eligible report; adds one study investigation and one cohort family	Country: Spain; Setting: University hospital complex of granada; Enrollment: 1998-01-01 to 2010-12-31; Analytic n: 114; Biopsy: technique Wedge	P	Selected; displayed, not pooled	graft failure x chronic composite score
39	Di Paolo, 2002	Eligible report; adds one study investigation and one cohort family	Effect-specific analytic n; see Table S3; Biopsy: technique varied; processing varied	P	Pooled meta-analysis	Pooled: VASCULAR-DGF; selected/other: DGF x IFTA; DGF x chronic composite score; DGF x glomerulosclerosis; DGF x tubular atrophy; DGF x vascular injury

Ref.	First author, year	Study-unit role	Study descriptors from retained extraction	Domains	Synthesis route	Analysis area(s) contributed
249	Dong, 2024	Eligible report; adds one study investigation and one cohort family	Context: No eligible baseline-adjusted histology-outcome ratio.	M	Other eligible evidence	Measurement: CEUS against procurement-biopsy remuzzi/pathology
219	Doshi, 2017	Contextual evidence unit; excluded from the primary prognostic meta-analysis	No report-level descriptive fields in the retained analytic extraction	U	Contextual evidence only	Contextual evidence only
156	Eccher, 2025	Eligible report; adds one study investigation and one cohort family	No report-level descriptive fields in the retained analytic extraction	P	Other eligible evidence	Domain evidence without a retained prognostic effect
45	Edwards, 2004	Eligible companion/overlapping report; adds no independent population	No report-level descriptive fields in the retained analytic extraction	P	Other eligible evidence	Domain evidence without a retained prognostic effect
128	Ellingsen, 2021	Eligible report; adds one study investigation and one cohort family	No report-level descriptive fields in the retained analytic extraction	P	Other eligible evidence	Domain evidence without a retained prognostic effect
214	Ernst, 2024	Eligible report; adds one study investigation and one cohort family	Target population: Adult recipients of single kidneys from adult brain-dead donors in Eurotransplant; no donation after circulatory death (DCD) kidneys; Data sources: multiple (2); Samples: multiple (4)	I	Other eligible evidence	Phases: Apparent performance evaluation; External performance evaluation; Model development; Prediction: DGF dialysis within 7d; Death censored graft failure 1y
41	Eschwège, 2002	Eligible report; adds one study investigation and one cohort family	No report-level descriptive fields in the retained analytic extraction	P	Other eligible evidence	Domain evidence without a retained prognostic effect
68	Fernández-Lorente, 2012	Eligible companion/overlapping report; adds no independent population	No report-level descriptive fields in the retained analytic extraction	P	Selected; displayed, not pooled	all-cause graft failure x Remuzzi score
217	Frutos, 2006	Contextual evidence unit; excluded from the primary prognostic meta-analysis	No report-level descriptive fields in the retained analytic extraction	U	Contextual evidence only	Contextual evidence only
227	Frutos, 2012	Eligible report; adds one study investigation and one cohort family	Design: Single-center nonrandomized case-control; Dual-kidney group: 20; Single/reference group: 40	U	Other eligible evidence	Clinical utility: Creatinine clearance at 6 months; Delayed graft function; Dialysis-free graft survival at 3 years
234	Gaber, 1995	Contextual evidence unit; excluded from the primary prognostic meta-analysis	No report-level descriptive fields in the retained analytic extraction	M	Contextual evidence only	Contextual evidence only
207	Gandolfini, 2014	Eligible report; adds one study investigation and one cohort family	No report-level descriptive fields in the retained analytic extraction	P, U	Other eligible evidence	Domain evidence without a retained prognostic effect
255	García-Covarrubias, 2020	Contextual evidence unit; excluded from the primary prognostic meta-analysis	No report-level descriptive fields in the retained analytic extraction	–	Contextual evidence only	Contextual evidence only
73	García-Rubio, 2013	Eligible report; adds one study investigation and one cohort family	No report-level descriptive fields in the retained analytic extraction	P	Other eligible evidence	Domain evidence without a retained prognostic effect
187	Gaut, 2025	Eligible report; adds one study investigation and one cohort family	No report-level descriptive fields in the retained analytic extraction	P, I	Other eligible evidence	Domain evidence without a retained prognostic effect
137	Gilbert, 2022	Eligible report; adds one study investigation and one cohort family	No report-level descriptive fields in the retained analytic extraction	P	Other eligible evidence	Domain evidence without a retained prognostic effect
182	Gomes Filho, 2021	Eligible report; adds one study investigation and one cohort family	No report-level descriptive fields in the retained analytic extraction	P, I	Other eligible evidence	Domain evidence without a retained prognostic effect
221	Gonzalez Sepulveda, 2023	Contextual evidence unit; excluded from the primary prognostic meta-analysis	No report-level descriptive fields in the retained analytic extraction	U	Contextual evidence only	Contextual evidence only
225	Goplani, 2010	Eligible report; adds one study investigation and one cohort family	No report-level descriptive fields in the retained analytic extraction	U	Other eligible evidence	Domain evidence without a retained prognostic effect
204	Greenstein, 2006	Eligible report; adds one study investigation and one cohort family	No report-level descriptive fields in the retained analytic extraction	P, U	Other eligible evidence	Domain evidence without a retained prognostic effect
173	Günther, 2017	Eligible report; adds one study investigation and one cohort family	No report-level descriptive fields in the retained analytic extraction	P, I	Other eligible evidence	Domain evidence without a retained prognostic effect
77	Hall, 2014	Eligible companion/overlapping report; adds no independent population	No report-level descriptive fields in the retained analytic extraction	P	Other eligible evidence	Domain evidence without a retained prognostic effect
176	Hall, 2018	Eligible report; adds one study investigation and one cohort family	No report-level descriptive fields in the retained analytic extraction	P, I	Other eligible evidence	Domain evidence without a retained prognostic effect
253	Han, 2014	Contextual evidence unit; excluded from the primary prognostic meta-analysis	No report-level descriptive fields in the retained analytic extraction	–	Contextual evidence only	Contextual evidence only
98	Hansen, 2018	Eligible report; adds one study investigation and one cohort family	No report-level descriptive fields in the retained analytic extraction	P	Other eligible evidence	Domain evidence without a retained prognostic effect

Ref.	First author, year	Study-unit role	Study descriptors from retained extraction	Domains	Synthesis route	Analysis area(s) contributed
238	He, 2024	Contextual evidence unit; excluded from the primary prognostic meta-analysis	No report-level descriptive fields in the retained analytic extraction	M	Contextual evidence only	Contextual evidence only
106	Heilman, 2019	Eligible companion/overlapping report; adds no independent population	No report-level descriptive fields in the retained analytic extraction	P	Other eligible evidence	Domain evidence without a retained prognostic effect
175	Heylen, 2018	Eligible report; adds one study investigation and one cohort family	No report-level descriptive fields in the retained analytic extraction	P, I	Other eligible evidence	Domain evidence without a retained prognostic effect
75	Hofer, 2014	Eligible report; adds one study investigation and one cohort family	Country: Austria; Setting: Vienna general hospital (medical university of vienna); Enrollment: 1999-01-01 to 2003-12-31 (transplants); median follow-up 6.8 yr (IQR 6.3); event-free recipients 7.8 yr; Analytic n: 628 (primary CLS analysis; sensitivity analysis restricted to 529 organs with own biopsy >=10 glomeruli); Biopsy: technique Wedge; processing Paraffin	P	Selected; displayed, not pooled	all-cause graft failure x IFTA; all-cause graft failure x chronic composite score; all-cause graft failure x tubular atrophy; all-cause graft failure x vascular injury
218	Hosgood, 2015	Contextual evidence unit; excluded from the primary prognostic meta-analysis	No report-level descriptive fields in the retained analytic extraction	U	Contextual evidence only	Contextual evidence only
44	Howie, 2004	Eligible report; adds one study investigation and one cohort family	No report-level descriptive fields in the retained analytic extraction	P	Other eligible evidence	Domain evidence without a retained prognostic effect
190	Hruba, 2026	Eligible report; adds one study investigation and one cohort family	No report-level descriptive fields in the retained analytic extraction	P, I	Other eligible evidence	Domain evidence without a retained prognostic effect
242	Huang, 2018	Eligible report; adds one study investigation and one cohort family	No report-level descriptive fields in the retained analytic extraction	M	Other eligible evidence	Domain evidence without a retained prognostic effect
116	Husain, 2020a	Eligible report; adds one study investigation and one cohort family	No report-level descriptive fields in the retained analytic extraction	P	Other eligible evidence	Domain evidence without a retained prognostic effect
117	Husain, 2020b	Eligible companion/overlapping report; adds no independent population	No report-level descriptive fields in the retained analytic extraction	P	Other eligible evidence	Domain evidence without a retained prognostic effect
123	Husain, 2021	Eligible companion/overlapping report; adds no independent population	Enrollment: 2011-2020 (needle: transplanted 2011-2016 & 2019-2020; discards 2015-2020); wedge-biopsy cohort transplanted 2019-2021; Analytic n: 2033; Biopsy: technique Both; processing Frozen	P	Selected; displayed, not pooled	DGF x chronic composite score; graft failure x chronic composite score; primary nonfunction x chronic composite score
130	Husain, 2022	Eligible report; adds one study investigation and one cohort family	Enrollment: 2015-2019; Analytic n: 30469; Biopsy: technique Both; processing Frozen	P	Selected; displayed, not pooled	graft failure x chronic composite score
250	Hysi, 2025	Eligible report; adds one study investigation and one cohort family	Cohort: First in human qus fibrosis; Context: No eligible preimplantation lesion-to-outcome effect.	M	Other eligible evidence	Measurement: Quantitative ultrasound assessment of transplant-kidney fibrosis
49	Ibernon, 2007	Eligible report; adds one study investigation and one cohort family	No report-level descriptive fields in the retained analytic extraction	P	Other eligible evidence	Domain evidence without a retained prognostic effect
86	Jacobi, 2016	Eligible companion/overlapping report; adds no independent population	Analytic n: 200; Biopsy: technique Core	P	Selected; displayed, not pooled	DGF x chronic composite score; primary nonfunction x chronic composite score
189	Jiang, 2025	Eligible report; adds one study investigation and one cohort family	Enrollment: 2011-10 to 2019-10; Analytic n: 256; Biopsy: technique Core; processing Paraffin	P, I	Selected; displayed, not pooled	graft failure x Remuzzi score
169	Kahu, 2011	Eligible report; adds one study investigation and one cohort family	No report-level descriptive fields in the retained analytic extraction	P, I	Other eligible evidence	Domain evidence without a retained prognostic effect
111	Kaisar, 2019	Eligible report; adds one study investigation and one cohort family	No report-level descriptive fields in the retained analytic extraction	P	Other eligible evidence	Domain evidence without a retained prognostic effect
108	Kalthoonical, 2019	Eligible report; adds one study investigation and one cohort family	No report-level descriptive fields in the retained analytic extraction	P	Other eligible evidence	Domain evidence without a retained prognostic effect
9	Kasiske, 2014	Eligible companion/overlapping report; adds no independent population	No report-level descriptive fields in the retained analytic extraction	P	Other eligible evidence	Domain evidence without a retained prognostic effect
55	Kayler, 2008	Eligible report; adds one study investigation and one cohort family	Country: United States; Setting: University of pittsburgh medical center (thomas e. starzl transplantation institute), pittsburgh, pa; Enrollment: Transplants performed 1989-2006 (patients screened jan 1987-oct 2006); Analytic n: 597; Biopsy: technique Wedge; processing Paraffin	P	Selected; displayed, not pooled	all-cause graft failure x glomerulosclerosis; all-cause graft failure x vascular injury
57	Kayler, 2009	Eligible report; adds one study investigation and one cohort family	No report-level descriptive fields in the retained analytic extraction	P	Other eligible evidence	Domain evidence without a retained prognostic effect

Ref.	First author, year	Study-unit role	Study descriptors from retained extraction	Domains	Synthesis route	Analysis area(s) contributed
200	Keijbeck, 2020	Eligible report; adds one study investigation within a linked cohort family	No report-level descriptive fields in the retained analytic extraction	P, M	Other eligible evidence	Domain evidence without a retained prognostic effect
212	Kim, 2024	Contextual evidence unit; excluded from the primary prognostic meta-analysis	No report-level descriptive fields in the retained analytic extraction	I	Contextual evidence only	Contextual evidence only
236	Konkel, 2019	Contextual evidence unit; excluded from the primary prognostic meta-analysis	No report-level descriptive fields in the retained analytic extraction	M	Contextual evidence only	Contextual evidence only
240	Koo, 1998	Eligible report; adds one study investigation and one cohort family	Context: Post-reperfusion change is contextual and outside strict pre-reperfusion prognostic pooling.	M	Other eligible evidence	Measurement: Paired pre/post-reperfusion immunohistochemistry
54	Koppelstaetter, 2008	Eligible report; adds one study investigation and one cohort family	No report-level descriptive fields in the retained analytic extraction	P	Other eligible evidence	Domain evidence without a retained prognostic effect
80	Kosmoliaptsis, 2015	Eligible report; adds one study investigation and one cohort family	No report-level descriptive fields in the retained analytic extraction	P	Other eligible evidence	Domain evidence without a retained prognostic effect
237	Kuroyanagi, 2023	Contextual evidence unit; excluded from the primary prognostic meta-analysis	No report-level descriptive fields in the retained analytic extraction	M	Contextual evidence only	Contextual evidence only
226	Kute, 2012	Eligible companion/overlapping report; adds no independent population	No report-level descriptive fields in the retained analytic extraction	U	Other eligible evidence	Domain evidence without a retained prognostic effect
72	La Manna, 2013	Eligible companion/overlapping report; adds no independent population	No report-level descriptive fields in the retained analytic extraction	P	Other eligible evidence	Domain evidence without a retained prognostic effect
152	Langer, 2025	Eligible report; adds one study investigation and one cohort family	No report-level descriptive fields in the retained analytic extraction	P	Other eligible evidence	Domain evidence without a retained prognostic effect
83	Lee, 2016	Eligible report; adds one study investigation and one cohort family	Enrollment: 2006-12 to 2011-07; Analytic n: 135; Biopsy: technique varied; processing varied	P	Pooled meta-analysis	Pooled: VASCULAR-DGF; selected/other: DGF x vascular injury; eGFR x glomerulosclerosis; eGFR x tubular atrophy; eGFR x vascular injury
159	Lee, 2026	Eligible companion/overlapping report; adds no independent population	Country: United States; Setting: US OPTN/STAR national registry; Enrollment: 2014-2022; Analytic n: 13962; Biopsy: technique Both; processing Frozen	P	Pooled meta-analysis	Pooled: GS-DGF; GS-GRAFT FAILURE; IFTA-DGF; IFTA-eGFR BINARY LOW; IFTA-GRAFT FAILURE; VASCULAR-DGF; VASCULAR-eGFR BINARY LOW; VASCULAR-GRAFT FAILURE; selected/other: DGF x GS MPP joint; DGF x IFTA; DGF x glomerulosclerosis; DGF x vascular injury; graft failure x GS MPP joint; +11 more (see domain table)
105	Lentine, 2019	Eligible companion/overlapping report; adds no independent population	No report-level descriptive fields in the retained analytic extraction	P	Other eligible evidence	Domain evidence without a retained prognostic effect
245	Li, 2021	Eligible report; adds one study investigation and one cohort family	No report-level descriptive fields in the retained analytic extraction	M	Other eligible evidence	Domain evidence without a retained prognostic effect
148	Li, 2024	Eligible companion/overlapping report; adds no independent population	Country: China; Setting: First affiliated hospital of xi'an jiaotong university, nephropathy hospital, department of renal transplantation, xi'an, shaanxi; Enrollment: Development: may 2018-december 2019; external validation: january 2020-april 2020; Analytic n: 492; Biopsy: technique varied; processing varied	P	Pooled meta-analysis	Pooled: ACUTE TUBULAR INJURY-DGF; GS-DGF; IFTA-DGF; VASCULAR-DGF; selected/other: DGF x IFTA; DGF x acute tubular injury; DGF x chronic composite score; DGF x glomerulosclerosis; DGF x other acute lesion; +3 more (see domain table)
12	Liapis, 2017	Eligible report; adds one study investigation and one cohort family	No report-level descriptive fields in the retained analytic extraction	P	Other eligible evidence	Domain evidence without a retained prognostic effect
109	Lim, 2019	Eligible companion/overlapping report; adds no independent population	No report-level descriptive fields in the retained analytic extraction	P	Other eligible evidence	Domain evidence without a retained prognostic effect
239	Limas, 1977	Eligible report; adds one study investigation and one cohort family	No report-level descriptive fields in the retained analytic extraction	M	Other eligible evidence	Domain evidence without a retained prognostic effect
62	Lin, 2010	Eligible report; adds one study investigation and one cohort family	Enrollment: 2004-04 to 2008-04; Analytic n: 55; Biopsy: technique Wedge; processing Frozen	P	Pooled meta-analysis	Pooled: GS-GRAFT FAILURE; selected/other: graft failure x glomerulosclerosis
246	Loizeau, 2024	Eligible report; adds one study investigation and one cohort family	Cohort: Uk repeat biopsy metrology 144 cases; Context: No eligible post-transplant prognostic ratio.	M	Other eligible evidence	Measurement: Repeat-biopsy processing and assessment uncertainty
193	Lopes, 2005	Eligible companion/overlapping report; adds no independent population	Country: Spain; Setting: Bellvitge hospital, barcelona; Enrollment: 2000-06 to 2001-12; Analytic n: 77; Biopsy: technique varied	P, M	Selected; displayed, not pooled	DGF x glomerulosclerosis; graft failure x chronic composite score

Ref.	First author, year	Study-unit role	Study descriptors from retained extraction	Domains	Synthesis route	Analysis area(s) contributed
76	Losappio, 2014	Eligible companion/overlapping report; adds no independent population	Country: Italy; Setting: University of bari transplant center (bari, italy); analysis/authorship also university of foggia; Enrollment: 1997-2007; Analytic n: 372; Biopsy: technique varied; processing varied	P	Selected; displayed, not pooled	DGF x Remuzzi score; primary nonfunction x chronic composite score
196	Loverre, 2008	Eligible report; adds one study investigation and one cohort family	No report-level descriptive fields in the retained analytic extraction	P, M	Other eligible evidence	Domain evidence without a retained prognostic effect
154	Loza, 2025	Eligible report; adds one study investigation and one cohort family	No report-level descriptive fields in the retained analytic extraction	P	Other eligible evidence	Domain evidence without a retained prognostic effect
36	Lu, 2000	Eligible report; adds one study investigation and one cohort family	Setting: Stanford; Enrollment: 1995-02 to 1998-11; Analytic n: 89	P	Pooled meta-analysis	Pooled: GS-DGF; selected/other: DGF x glomerulosclerosis
38	Lu, 2001	Eligible companion/overlapping report; adds no independent population	Setting: Stanford; Enrollment: 1995-02 to 1998-11; Analytic n: 89	P	Selected; displayed, not pooled	graft failure x chronic composite score
47	Lubuska, 2006	Eligible report; adds one study investigation and one cohort family	Enrollment: 1994 to 1997	P	Pooled meta-analysis	Pooled: GS-DGF; IFTA-DGF; VASCULAR-DGF; selected/other: DGF x IFTA; DGF x glomerulosclerosis; DGF x other acute lesion; DGF x vascular injury; graft failure x IFTA; +4 more (see domain table)
213	Luo, 2021	Eligible report; adds one study investigation and one cohort family	No report-level descriptive fields in the retained analytic extraction	I	Other eligible evidence	Domain evidence without a retained prognostic effect
121	Luque, 2021	Eligible companion/overlapping report; adds no independent population	No report-level descriptive fields in the retained analytic extraction	P	Other eligible evidence	Domain evidence without a retained prognostic effect
79	Mallon, 2015	Eligible report; adds one study investigation and one cohort family	No report-level descriptive fields in the retained analytic extraction	P	Other eligible evidence	Domain evidence without a retained prognostic effect
143	Marletta, 2023	Eligible report; adds one study investigation and one cohort family	Enrollment: January 2015 - december 2019; Effect-specific analytic n; see Table S3; Biopsy: technique Both; processing Paraffin	P	Selected; displayed, not pooled	all-cause graft failure x IFTA; all-cause graft failure x glomerulosclerosis; all-cause graft failure x tubular atrophy
244	Marsh, 2021	Eligible report; adds one study investigation and one cohort family	Cohort: Deep learning glomerulosclerosis image validation; Context: No recipient outcome effect was estimated.	M	Other eligible evidence	Measurement: Automated glomerulosclerosis quantification
69	Martínez-Vaquera, 2013	Eligible report; adds one study investigation and one cohort family	No report-level descriptive fields in the retained analytic extraction	P	Other eligible evidence	Domain evidence without a retained prognostic effect
65	Mas, 2011	Eligible report; adds one study investigation and one cohort family	No report-level descriptive fields in the retained analytic extraction	P	Other eligible evidence	Domain evidence without a retained prognostic effect
56	Matignon, 2008	Eligible report; adds one study investigation and one cohort family	Country: France; Setting: Henri mondor hospital, creteil (pathology co-read at necker hospital, paris), ap-hp; Enrollment: 2002 to 2006; Analytic n: 172; Biopsy: technique varied; processing varied	P	Pooled meta-analysis	Pooled: ACUTE TUBULAR INJURY-DGF; GS-DGF; IFTA-DGF; VASCULAR-DGF; selected/other: DGF x IFTA; DGF x acute tubular injury; DGF x glomerulosclerosis; DGF x other acute lesion; DGF x vascular injury
88	Matos, 2016	Eligible report; adds one study investigation and one cohort family	No report-level descriptive fields in the retained analytic extraction	P	Other eligible evidence	Domain evidence without a retained prognostic effect
215	Matti, 2024	Eligible report; adds one study investigation and one cohort family	No report-level descriptive fields in the retained analytic extraction	I, M	Other eligible evidence	Domain evidence without a retained prognostic effect
43	McCall, 2003	Eligible report; adds one study investigation and one cohort family	No report-level descriptive fields in the retained analytic extraction	P	Other eligible evidence	Domain evidence without a retained prognostic effect
58	McGlynn, 2009	Eligible report; adds one study investigation and one cohort family	No report-level descriptive fields in the retained analytic extraction	P	Other eligible evidence	Domain evidence without a retained prognostic effect
230	Messina, 2017	Eligible report; adds one study investigation and one cohort family	No report-level descriptive fields in the retained analytic extraction	U	Other eligible evidence	Domain evidence without a retained prognostic effect
141	Miri, 2023	Eligible report; adds one study investigation and one cohort family	No report-level descriptive fields in the retained analytic extraction	P	Other eligible evidence	Domain evidence without a retained prognostic effect
241	Mittal, 2017	Eligible report; adds one study investigation and one cohort family	No report-level descriptive fields in the retained analytic extraction	M	Other eligible evidence	Domain evidence without a retained prognostic effect
92	Mohan, 2017	Eligible report; adds one study investigation and one cohort family	No report-level descriptive fields in the retained analytic extraction	P	Other eligible evidence	Domain evidence without a retained prognostic effect

Ref.	First author, year	Study-unit role	Study descriptors from retained extraction	Domains	Synthesis route	Analysis area(s) contributed
142	Mokos, 2023	Eligible companion/overlapping report; adds no independent population	No report-level descriptive fields in the retained analytic extraction	P	Other eligible evidence	Domain evidence without a retained prognostic effect
199	Moledina, 2017	Eligible companion/overlapping report; adds no independent population	Cohort: Nm093; Context: Adjusted 6-month eGFR 43.2 severe, 46.1 mild, 49.4 no ATI; contrast variance not reported.	P, M	Other eligible evidence	Measurement: Procurement-biopsy acute tubular injury
114	Mota-Zamorano, 2019	Eligible report; adds one study investigation and one cohort family	No report-level descriptive fields in the retained analytic extraction	P	Other eligible evidence	Domain evidence without a retained prognostic effect
87	Mourão, 2016	Eligible report; adds one study investigation and one cohort family	No report-level descriptive fields in the retained analytic extraction	P	Pooled meta-analysis	Pooled: ACUTE TUBULAR INJURY-DGF; selected/other: DGF x acute tubular injury
167	Munivenkatappa, 2008	Eligible report; adds one study investigation and one cohort family	Enrollment: 1999-01 to 2005-12; Analytic n: 259; Biopsy: technique Wedge; processing Both	P, I	Pooled meta-analysis	Pooled: GS-GRAFT FAILURE; IFTA-GRAFT FAILURE; VASCULAR-GRAFT FAILURE; selected/other: graft failure x IFTA; graft failure x acute tubular injury; graft failure x glomerulosclerosis; graft failure x vascular injury
150	Murata, 2024	Eligible report; adds one study investigation and one cohort family	No report-level descriptive fields in the retained analytic extraction	P	Other eligible evidence	Domain evidence without a retained prognostic effect
201	Nachiappa Ganesh, 2023	Eligible report; adds one study investigation and one cohort family	No report-level descriptive fields in the retained analytic extraction	P, M	Other eligible evidence	Domain evidence without a retained prognostic effect
251	Nardo, 2011	Contextual evidence unit; excluded from the primary prognostic meta-analysis	No report-level descriptive fields in the retained analytic extraction	–	Contextual evidence only	Contextual evidence only
66	Navarro, 2011	Eligible report; adds one study investigation and one cohort family	Setting: Hospital reina sofia, cordoba, spain; Enrollment: Not stated beyond long-term follow-up; Analytic n: 136	P	Selected; displayed, not pooled	graft failure x chronic composite score
134	Ninan, 2022	Eligible report; adds one study investigation and one cohort family	No report-level descriptive fields in the retained analytic extraction	P	Other eligible evidence	Domain evidence without a retained prognostic effect
224	O'Connor, 2009	Eligible report; adds one study investigation and one cohort family	No report-level descriptive fields in the retained analytic extraction	U	Other eligible evidence	Domain evidence without a retained prognostic effect
232	Odaldi, 2022	Eligible report; adds one study investigation within a linked cohort family	Design: Retrospective cohort with propensity-score adjustment; Single/reference group: 322 total; 207 in situ and 115 ex situ	U	Other eligible evidence	Clinical utility: Graft loss
153	Oh, 2025	Eligible report; adds one study investigation and one cohort family	No report-level descriptive fields in the retained analytic extraction	P	Other eligible evidence	Domain evidence without a retained prognostic effect
194	Ortiz, 2005	Eligible companion/overlapping report; adds no independent population	No report-level descriptive fields in the retained analytic extraction	P, M	Other eligible evidence	Domain evidence without a retained prognostic effect
248	Pan, 2024	Eligible report; adds one study investigation and one cohort family	Cohort: China ex vivo ste 2022 2023; Context: Authorized route is domain m; not entered into morphology prognostic pools.	M	Other eligible evidence	Measurement: Sound-touch elastography against procurement histology/allograft function
67	Patel, 2012	Eligible report; adds one study investigation and one cohort family	Enrollment: 2006-05 to 2010-12; Analytic n: 47; Biopsy: processing Both	P	Selected; displayed, not pooled	DGF x chronic composite score; eGFR x chronic composite score; graft failure x chronic composite score
145	Paterson, 2024	Eligible report; adds one study investigation and one cohort family	No report-level descriptive fields in the retained analytic extraction	P	Other eligible evidence	Domain evidence without a retained prognostic effect
135	Pavlovic, 2022	Eligible report; adds one study investigation and one cohort family	Analytic n: 73; Biopsy: technique varied; processing varied	P	Pooled meta-analysis	Pooled: REMUZZI-DGF; selected/other: DGF x IFTA; DGF x Remuzzi score
96	Pegas, 2018	Eligible report; adds one study investigation and one cohort family	Enrollment: 2006 to 2013; Analytic n: 430; Biopsy: technique varied; processing varied	P	Pooled meta-analysis	Pooled: GS-GRAFT FAILURE; IFTA-GRAFT FAILURE; TUBULAR ATROPHY-GRAFT FAILURE; VASCULAR-GRAFT FAILURE; selected/other: graft failure x IFTA; graft failure x glomerulosclerosis; graft failure x tubular atrophy; graft failure x vascular injury
197	Perco, 2009	Eligible report; adds one study investigation and one cohort family	No report-level descriptive fields in the retained analytic extraction	P, M	Other eligible evidence	Domain evidence without a retained prognostic effect
125	Perez-Gutierrez, 2021	Eligible report; adds one study investigation and one cohort family	Effect-specific analytic n; see Table S3; Biopsy: technique varied; processing varied	P	Pooled meta-analysis	Pooled: VASCULAR-DGF; selected/other: DGF x acute tubular injury; DGF x glomerulosclerosis; DGF x tubular atrophy; DGF x vascular injury
110	Phillips, 2019	Eligible report; adds one study investigation and one cohort family	No report-level descriptive fields in the retained analytic extraction	P	Other eligible evidence	Domain evidence without a retained prognostic effect
170	Philosophe, 2014	Eligible report; adds one study investigation and one cohort family	Analytic n: 196	P, I	Selected; displayed, not pooled	graft failure x Karpinski/MAPI

Ref.	First author, year	Study-unit role	Study descriptors from retained extraction	Domains	Synthesis route	Analysis area(s) contributed
252	Pierobon, 2013	Contextual evidence unit; excluded from the primary prognostic meta-analysis	No report-level descriptive fields in the retained analytic extraction	–	Contextual evidence only	Contextual evidence only
37	Pokorná, 2001	Eligible report; adds one study investigation and one cohort family	No report-level descriptive fields in the retained analytic extraction	P	Other eligible evidence	Domain evidence without a retained prognostic effect
202	Polyak, 2000	Eligible report; adds one study investigation and one cohort family	No report-level descriptive fields in the retained analytic extraction	P, U	Other eligible evidence	Domain evidence without a retained prognostic effect
188	Proença, 2025	Eligible report; adds one study investigation and one cohort family	Analytic n: 339 recipients; Biopsy: technique Both; processing Paraffin	P, I	Pooled meta-analysis	Pooled: IFTA-eGFR BINARY LOW; VASCULAR-eGFR BINARY LOW; selected/other: low eGFR x IFTA; low eGFR x Karpinski/MAPI; low eGFR x Remuzzi score; low eGFR x acute tubular injury; low eGFR x cortical scarring; +6 more (see domain table)
78	Pêgas, 2014	Eligible report; adds one study investigation and one cohort family	No report-level descriptive fields in the retained analytic extraction	P	Other eligible evidence	Domain evidence without a retained prognostic effect
70	Ramalho, 2013	Eligible report; adds one study investigation and one cohort family	No report-level descriptive fields in the retained analytic extraction	P	Other eligible evidence	Domain evidence without a retained prognostic effect
160	Rampersad, 2026	Eligible companion/overlapping report; adds no independent population	No report-level descriptive fields in the retained analytic extraction	P	Other eligible evidence	Domain evidence without a retained prognostic effect
46	Re, 2006	Eligible report; adds one study investigation and one cohort family	No report-level descriptive fields in the retained analytic extraction	P	Other eligible evidence	Domain evidence without a retained prognostic effect
206	Re, 2010	Eligible report; adds one study investigation and one cohort family	No report-level descriptive fields in the retained analytic extraction	P, U	Other eligible evidence	Domain evidence without a retained prognostic effect
180	Reese, 2021	Eligible report; adds one study investigation and one cohort family	Effect-specific analytic n; see Table S3; Biopsy: technique varied; processing varied	P, I	Pooled meta-analysis	Pooled: GS-GRAFT FAILURE; IFTA-GRAFT FAILURE; VASCULAR-GRAFT FAILURE; selected/other: graft failure x IFTA; graft failure x glomerulosclerosis; graft failure x vascular injury
222	Remuzzi, 2006	Eligible report; adds one study investigation and one cohort family	Design: Prospective nonrandomized comparative cohort; Dual-kidney group: Histology-guided group included single and dual recipients; Single/reference group: 62 histology-evaluated recipients; 124 older-donor negative references	U	Other eligible evidence	Clinical utility: Graft failure/return to dialysis
235	Roake, 1996	Contextual evidence unit; excluded from the primary prognostic meta-analysis	No report-level descriptive fields in the retained analytic extraction	M	Contextual evidence only	Contextual evidence only
192	Rohr, 1983	Eligible report; adds one study investigation and one cohort family	No report-level descriptive fields in the retained analytic extraction	P, M	Pooled meta-analysis	Pooled: ACUTE TUBULAR INJURY-DGF; selected/other: DGF x acute tubular injury
84	Sagasta, 2016	Eligible report; adds one study investigation and one cohort family	No report-level descriptive fields in the retained analytic extraction	P	Other eligible evidence	Domain evidence without a retained prognostic effect
157	Sageshima, 2025	Eligible report; adds one study investigation within a linked cohort family	No report-level descriptive fields in the retained analytic extraction	P	Other eligible evidence	Domain evidence without a retained prognostic effect
178	Saha-Chaudhuri, 2020	Eligible report; adds one study investigation and one cohort family	Analytic n: 163; Biopsy: technique Wedge; processing Frozen	P, I	Selected; displayed, not pooled	eGFR x Karpinski/MAPI; eGFR x Remuzzi score; eGFR x glomerulosclerosis; eGFR x tubular atrophy
243	Salvi, 2020	Eligible report; adds one study investigation and one cohort family	Context: Computational morphology; no clinical prognostic ratio.	M	Other eligible evidence	Measurement: Automated vascular and fibrosis segmentation
220	Schantz, 2022	Contextual evidence unit; excluded from the primary prognostic meta-analysis	No report-level descriptive fields in the retained analytic extraction	U	Contextual evidence only	Contextual evidence only
138	Scurt, 2023a	Eligible companion/overlapping report; adds no independent population	No report-level descriptive fields in the retained analytic extraction	P	Other eligible evidence	Domain evidence without a retained prognostic effect
140	Scurt, 2023b	Eligible companion/overlapping report; adds no independent population	No report-level descriptive fields in the retained analytic extraction	P	Other eligible evidence	Domain evidence without a retained prognostic effect
186	Scurt, 2023c	Eligible report; adds one study investigation and one cohort family	Analytic n: 223; Biopsy: technique Both; processing Paraffin	P, I	Selected; displayed, not pooled	graft failure x chronic composite score
146	Scurt, 2024	Eligible companion/overlapping report; adds no independent population	No report-level descriptive fields in the retained analytic extraction	P	Other eligible evidence	Domain evidence without a retained prognostic effect

Ref.	First author, year	Study-unit role	Study descriptors from retained extraction	Domains	Synthesis route	Analysis area(s) contributed
161	Sibulesky, 2026	Eligible companion/overlapping report; adds no independent population	No report-level descriptive fields in the retained analytic extraction	P	Other eligible evidence	Domain evidence without a retained prognostic effect
61	Snanoudj, 2009	Eligible report; adds one study investigation within a linked cohort family	No report-level descriptive fields in the retained analytic extraction	P	Other eligible evidence	Domain evidence without a retained prognostic effect
48	Snoeijs, 2006	Eligible companion/overlapping report; adds no independent population	No report-level descriptive fields in the retained analytic extraction	P	Other eligible evidence	Domain evidence without a retained prognostic effect
166	Snoeijs, 2008	Eligible report; adds one study investigation and one cohort family	No report-level descriptive fields in the retained analytic extraction	P, I	Other eligible evidence	Domain evidence without a retained prognostic effect
132	So, 2022	Eligible report; adds one study investigation and one cohort family	No report-level descriptive fields in the retained analytic extraction	P	Other eligible evidence	Domain evidence without a retained prognostic effect
81	Sood, 2015	Eligible report; adds one study investigation and one cohort family	No report-level descriptive fields in the retained analytic extraction	P	Other eligible evidence	Domain evidence without a retained prognostic effect
133	Stewart, 2022	Eligible companion/overlapping report; adds no independent population	Country: United States; Setting: US OPTN national registry; Enrollment: 2008-2012; Analytic n: 5997	P	Pooled meta-analysis	Pooled: VASCULAR-GRAFT FAILURE; selected/other: all-cause graft failure x IFTA; all-cause graft failure x glomerulosclerosis; all-cause graft failure x vascular injury; graft failure x vascular injury
50	Sulikowski, 2007	Eligible report; adds one study investigation and one cohort family	No report-level descriptive fields in the retained analytic extraction	P	Other eligible evidence	Domain evidence without a retained prognostic effect
63	Sulikowski, 2010	Eligible report; adds one study investigation and one cohort family	No report-level descriptive fields in the retained analytic extraction	P	Other eligible evidence	Domain evidence without a retained prognostic effect
233	Summers, 2025	Eligible report; adds one study investigation and one cohort family	No report-level descriptive fields in the retained analytic extraction	U	Other eligible evidence	Domain evidence without a retained prognostic effect
205	Sung, 2008	Eligible report; adds one study investigation and one cohort family	No report-level descriptive fields in the retained analytic extraction	P, U	Other eligible evidence	Domain evidence without a retained prognostic effect
93	Sánchez-Escuredo, 2017	Eligible report; adds one study investigation and one cohort family	Country: Spain; Setting: Hospital clínic barcelona (universitat de barcelona); Enrollment: 2000-2010 (donors detected jan 2000-dec 2010; 5-year follow-up); Analytic n: 364; Biopsy: technique varied; processing varied	P	Selected; displayed, not pooled	all-cause graft failure x IFTA; all-cause graft failure x glomerulosclerosis; all-cause graft failure x tubular atrophy; all-cause graft failure x vascular injury
147	Tan, 2024	Eligible companion/overlapping report; adds no independent population	Country: China; Setting: First affiliated hospital of sun yat-sen university, guangzhou; Enrollment: 2014 to 2018; Analytic n: 97; Biopsy: technique Core; processing Both	P	Pooled meta-analysis	Pooled: REMUZZI-DGF; selected/other: DGF x Karpinski/MAPI; DGF x Remuzzi score; DGF x chronic composite score
144	Tanriover, 2023	Eligible companion/overlapping report; adds no independent population	No report-level descriptive fields in the retained analytic extraction	P	Other eligible evidence	Domain evidence without a retained prognostic effect
107	Tavakkoli, 2019	Eligible report; adds one study investigation and one cohort family	No report-level descriptive fields in the retained analytic extraction	P	Other eligible evidence	Domain evidence without a retained prognostic effect
208	Tavares da Silva, 2014	Eligible report; adds one study investigation and one cohort family	No report-level descriptive fields in the retained analytic extraction	P, U	Other eligible evidence	Domain evidence without a retained prognostic effect
181	Teixeira, 2021	Eligible report; adds one study investigation and one cohort family	Enrollment: 2014-07 to 2017-12; Analytic n: 159; Biopsy: technique varied; processing varied	P, I	Pooled meta-analysis	Pooled: GS-DGF; GS-GRAFT FAILURE; IFTA-DGF; IFTA-eGFR BINARY LOW; VASCULAR-DGF; VASCULAR-eGFR BINARY LOW; VASCULAR-GRAFT FAILURE; selected/other: DGF x IFTA; DGF x MAPI component pgf; DGF x glomerulosclerosis; DGF x vascular injury; graft failure x glomerulosclerosis; +5 more (see domain table)
211	Tirasattayapitak, 2024	Contextual evidence unit; excluded from the primary prognostic meta-analysis	No report-level descriptive fields in the retained analytic extraction	P, I, U, M	Contextual evidence only	Contextual evidence only
112	Trailin, 2019a	Eligible report; adds one study investigation and one cohort family	No report-level descriptive fields in the retained analytic extraction	P	Other eligible evidence	Domain evidence without a retained prognostic effect
115	Trailin, 2019b	Eligible companion/overlapping report; adds no independent population	No report-level descriptive fields in the retained analytic extraction	P	Other eligible evidence	Domain evidence without a retained prognostic effect
174	Traynor, 2017	Eligible companion/overlapping report; adds no independent population	No report-level descriptive fields in the retained analytic extraction	P, I	Other eligible evidence	Domain evidence without a retained prognostic effect
191	Truong, 2019	Contextual evidence unit; excluded from the primary prognostic meta-analysis	No report-level descriptive fields in the retained analytic extraction	P, M	Contextual evidence only	Contextual evidence only

Ref.	First author, year	Study-unit role	Study descriptors from retained extraction	Domains	Synthesis route	Analysis area(s) contributed
216	Verran, 2001	Contextual evidence unit; excluded from the primary prognostic meta-analysis	No report-level descriptive fields in the retained analytic extraction	U	Contextual evidence only	Contextual evidence only
131	Villanego, 2022	Eligible report; adds one study investigation and one cohort family	Enrollment: 2010-01 to 2019-06; Analytic n: 266; Biopsy: technique varied; processing varied	P	Pooled meta-analysis	Pooled: GS-GRAFT FAILURE; IFTA-GRAFT FAILURE; TUBULAR ATROPHY-GRAFT FAILURE; VASCULAR-GRAFT FAILURE; selected/other: eGFR x Remuzzi score; graft failure x IFTA; graft failure x Remuzzi score; graft failure x glomerulosclerosis; graft failure x tubular atrophy; +1 more (see domain table)
126	Vonbrunn, 2021	Eligible report; adds one study investigation and one cohort family	No report-level descriptive fields in the retained analytic extraction	P	Other eligible evidence	Domain evidence without a retained prognostic effect
136	Vonbrunn, 2022	Eligible report; adds one study investigation and one cohort family	No report-level descriptive fields in the retained analytic extraction	P	Other eligible evidence	Domain evidence without a retained prognostic effect
183	Wang, 2021	Eligible companion/overlapping report; adds no independent population	No report-level descriptive fields in the retained analytic extraction	P, I	Other eligible evidence	Domain evidence without a retained prognostic effect
184	Wang, 2022	Eligible report; adds one study investigation and one cohort family	Enrollment: 2012-01 to 2017-06; Analytic n: 140; Biopsy: technique Core; processing Both	P, I	Pooled meta-analysis	Pooled: ACUTE TUBULAR INJURY-DGF; GS-DGF; IFTA-DGF; VASCULAR-DGF; selected/other: DGF x IFTA; DGF x acute tubular injury; DGF x glomerulosclerosis; DGF x tubular atrophy; DGF x vascular injury; +5 more (see domain table)
139	Wang, 2023	Eligible companion/overlapping report; adds no independent population	No report-level descriptive fields in the retained analytic extraction	P	Other eligible evidence	Domain evidence without a retained prognostic effect
210	Wang, 2025	Contextual evidence unit; excluded from the primary prognostic meta-analysis	No report-level descriptive fields in the retained analytic extraction	P, U, M	Contextual evidence only	Contextual evidence only
97	Wazna, 2018	Eligible report; adds one study investigation and one cohort family	Enrollment: 2000 to 2008; Analytic n: 300; Biopsy: processing Paraffin	P	Pooled meta-analysis	Pooled: VASCULAR-GRAFT FAILURE; selected/other: graft failure x vascular injury
60	Ważna, 2009	Eligible companion/overlapping report; adds no independent population	No report-level descriptive fields in the retained analytic extraction	P	Other eligible evidence	Domain evidence without a retained prognostic effect
113	Weissenbacher, 2019	Eligible report; adds one study investigation and one cohort family	No report-level descriptive fields in the retained analytic extraction	P	Selected; displayed, not pooled	DGF x Remuzzi score
59	Woestenburg, 2009	Eligible report; adds one study investigation and one cohort family	No report-level descriptive fields in the retained analytic extraction	P	Other eligible evidence	Domain evidence without a retained prognostic effect
198	Wohlfahrtova, 2014	Eligible report; adds one study investigation and one cohort family	No report-level descriptive fields in the retained analytic extraction	P, M	Other eligible evidence	Domain evidence without a retained prognostic effect
228	Woodside, 2012	Eligible report; adds one study investigation and one cohort family	No report-level descriptive fields in the retained analytic extraction	U	Other eligible evidence	Domain evidence without a retained prognostic effect
82	Yamakawa, 2015	Eligible report; adds one study investigation and one cohort family	No report-level descriptive fields in the retained analytic extraction	P	Other eligible evidence	Domain evidence without a retained prognostic effect
163	Yan, 2026	Eligible report; adds one study investigation and one cohort family	No report-level descriptive fields in the retained analytic extraction	P	Other eligible evidence	Domain evidence without a retained prognostic effect
231	Yap, 2021	Eligible report; adds one study investigation and one cohort family	No report-level descriptive fields in the retained analytic extraction	U	Other eligible evidence	Domain evidence without a retained prognostic effect
209	Yi, 2022	Eligible companion/overlapping report; adds no independent population	Cohort: US OPTN ai procurement biopsy 2011 2020 national overlap group; Context: Abstract companion of historical included pmid 37923131; no independent study/effect count.	P, I, M	Other eligible evidence	Measurement: Deep-learning compartment segmentation on frozen procurement biopsies
151	Yi, 2024	Eligible companion/overlapping report; adds no independent population	No report-level descriptive fields in the retained analytic extraction	P	Other eligible evidence	Domain evidence without a retained prognostic effect
172	Yong, 2015	Eligible report; adds one study investigation and one cohort family	No report-level descriptive fields in the retained analytic extraction	P, I	Other eligible evidence	Domain evidence without a retained prognostic effect
185	Zagni, 2022	Eligible report; adds one study investigation and one cohort family	Target population: Kidneys from Italian donation after circulatory death (DCD) donors after regional normothermic perfusion; donation after brain death (DBD) biopsies served as morphology controls; Data source: Retrospective Milan/NITp series, September 2015–July 2019; 47 biopsied DCD kidneys, 40 transplanted; Sample: 40 transplanted kidneys	P, I	Other eligible evidence	Phases: Apparent performance evaluation; Model development; Prediction: Poor early graft function DGF or SGF 7d

Ref.	First author, year	Study-unit role	Study descriptors from retained extraction	Domains	Synthesis route	Analysis area(s) contributed
158	Zanchetta, 2025	Eligible report; adds one study investigation and one cohort family	No report-level descriptive fields in the retained analytic extraction	P	Selected; displayed, not pooled	DGF x Karpinski/MAPI; graft failure x Karpinski/MAPI
119	Zheng, 2020	Eligible report; adds one study investigation and one cohort family	Enrollment: 2018-01 to 2019-09; Analytic n: 333; Biopsy: technique Core; processing Paraffin	P	Pooled meta-analysis	Pooled: VASCULAR-DGF; selected/other: DGF x Remuzzi score; DGF x acute tubular injury; DGF x vascular injury
127	Šenjug, 2021	Eligible report; adds one study investigation and one cohort family	No report-level descriptive fields in the retained analytic extraction	P	Other eligible evidence	Domain evidence without a retained prognostic effect

Abbreviations and symbols: ATI, acute tubular injury; CEUS, contrast-enhanced ultrasonography; CLS, chronic lesion score; DBD, donation after brain death; DCD, donation after circulatory death; DGF, delayed graft function; eGFR, estimated glomerular filtration rate; GS, glomerulosclerosis; I, incremental prediction; IFTA, interstitial fibrosis and tubular atrophy; IQR, interquartile range; M, measurement; MAPI, Maryland Aggregate Pathology Index; MPP, machine perfusion parameters; n, reported sample size; OPTN, Organ Procurement and Transplantation Network; P, prognostic association; Ref., reference number in the main manuscript; SGF, slow graft function; STAR, Standard Transplant Analysis and Research; U, clinical utility; UNOS, United Network for Organ Sharing; US, United States.

Table S3. Figure 2 appraisal crosswalk and prognostic-association evidence selected for synthesis

Panel A. Figure 2 appraisal crosswalk

Review question	Instrument	Unit counted	n	Distribution	Supporting location
Prognostic association	QUIPS	Unique design-applicable prognostic appraisal unit	192	12 moderate; 180 high	Table S3, Panel B
Incremental prediction	CHARMS with PROBAST+AI	Model-phase assessment	16	16 high	Table S4
Clinical utility	RoB 2 cluster or ROBINS-I	Outcome- or comparison-specific strategy appraisal	18	1 low; 7 some concerns; 4 serious; 5 critical; 1 not applicable	Table S5, Panel A
Measurement	QAREL or adapted method-comparison appraisal	Design-applicable measurement assessment	5	1 low; 2 moderate; 1 high; 1 not applicable	Tables S6A–S6B

Abbreviations and symbols: CHARMS, Critical Appraisal and Data Extraction for Systematic Reviews of Prediction Modelling Studies; n, number of assessment units; PROBAST, Prediction model Risk Of Bias ASsessment Tool; PROBAST+AI, Prediction model Risk Of Bias ASsessment Tool plus Artificial Intelligence; QAREL, Quality Appraisal for Reliability Studies; QUIPS, Quality In Prognosis Studies; RoB 2, revised Cochrane risk-of-bias tool for randomized trials; ROBINS-I, Risk Of Bias In Non-randomized Studies of Interventions.

Each design-applicable appraisal unit is counted once. Category labels retain their instrument-specific meanings and should not be interpreted as equivalent across tools. Two context-only units without an applicable appraisal are excluded from the denominator. The historical 27-study PROBAST subset underwent a second independent methodological review, with complete agreement across all 27 studies. The 19-study QUIPS update used an assessor-and-verifier workflow, and five disagreements were reconciled.

Panel B. Prognostic-association evidence selected for synthesis

Study/cohort	Outcome and horizon	Lesion or score	Contrast or unit	Effect (95% CI)	Adjustment	Analytic n	QUIPS	Cell k and route
Cheungpasitporn, 2020 (US OPTN/UNOS national…)	Acute rejection; 1 year	Glomerulosclerosis	GS >20% vs 11-20%	OR 1.10 (0.81 to 1.48)	Baseline-adjusted	1,730	High	k=1; SWiM
Carta, 2013 (distinct cohort)	Acute rejection; 3 years	Karpinski/MAPI score; Karpinski	score 4-5 versus score 1-3	OR 0.75 (0.26 to 2.19)	Crude; recalculated	100	Unknown	k=1; SWiM
Azancot, 2014 (distinct cohort)	Acute rejection; time-to-event, longest eligible	Chronic composite score; Study-specific: BANFF chronic donor score 0-15	score 4-5 or 6-7 versus <=3	OR 0.56 (CI not reported)	Crude; recalculated	117	Unknown	k=1; SWiM
Centellas-Pérez, 2019 (2011 to 2015)	All-cause graft failure; longest eligible follow-up	Chronic composite score; BANFF	4-5 vs 0-3 (0-5 scale)	OR 4.54 (1.43 to 14.50)	Crude; reported	183	High	k=1; SWiM
Hofer, 2014 (Vienna General Hosp.…)	All-cause graft failure; longest eligible follow-up	Chronic composite score; Study-specific: CLS	CLS 4-6 (moderate) vs 0-3 (mild)	HR 1.20 (0.83 to 1.74)	Baseline-adjusted	628 primary; 529 sensitivity	Moderate	k=1; SWiM
Stewart, 2022 (US OPTN national registry)	All-cause graft failure; longest eligible follow-up	Glomerulosclerosis	11%+ versus 0%-5%	HR 1.18 (1.07 to 1.28)	Baseline-adjusted	5,997	Unknown	k=2; forest; no pooled estimate
Bajwa, 2007	All-cause graft failure; longest eligible follow-up	Glomerulosclerosis	GS >5% vs 0-5%	HR 1.15 (1.06 to 1.24)	Baseline-adjusted	12,129	High	k=2; forest; no pooled estimate
Marletta, 2023 (January 2015 - December 2019)	All-cause graft failure; longest eligible follow-up	Glomerulosclerosis	Per 10 percentage points	OR 4.05 (1.79 to 12.76)	Baseline-adjusted	78	High	k=1; SWiM
Kayler, 2008 (University of Pittsburgh…)	All-cause graft failure; longest eligible follow-up	Glomerulosclerosis	category 4 versus 0	OR 3.60 (0.61 to 21.32)	Crude; recalculated	597	High	k=1; SWiM
Sánchez-Escuredo, 2017 (Hosp. Clínic Barcelona…)	All-cause graft failure; longest eligible follow-up	Glomerulosclerosis	per RS grade increment (0/1-20%/21-30%/>30% global sclerosis)	HR 1.39 (0.48 to 4.02)	Baseline-adjusted	364	High	k=1; SWiM
Kayler, 2008 (University of Pittsburgh…)	All-cause graft failure; longest eligible follow-up	Glomerulosclerosis	per category	OR 1.35 (1.02 to 1.78)	Crude; reported	597	High	k=1; SWiM
Marletta, 2023 (distinct cohort)	All-cause graft failure; longest eligible follow-up	Glomerulosclerosis; Computational whole-slide glomerulosclerosis	Per 10 percentage points	OR 4.05 (1.34 to 18.67)	Baseline-adjusted	57	Unknown	k=1; SWiM
Stewart, 2022 (US OPTN national registry)	All-cause graft failure; longest eligible follow-up	Interstitial fibrosis/tubular atrophy	mild-moderate/severe versus absent/minimal	HR 1.14 (0.95 to 1.39)	Baseline-adjusted	5,997	Unknown	k=1; SWiM
Hofer, 2014 (Vienna General Hosp.…)	All-cause graft failure; longest eligible follow-up	Interstitial fibrosis/tubular atrophy	one-grade increase on 0-3 component score	HR 0.97 (0.73 to 1.29)	Crude; reported	628 primary; 529 sensitivity	Moderate	k=2; forest; no pooled estimate
Sánchez-Escuredo, 2017 (Hosp. Clínic Barcelona…)	All-cause graft failure; longest eligible follow-up	Interstitial fibrosis/tubular atrophy	per Remuzzi grade 0-3	HR 3.06 (1.56 to 6.03)	Baseline-adjusted	364	High	k=2; forest; no pooled estimate
Marletta, 2023 (distinct cohort)	All-cause graft failure; longest eligible follow-up	Interstitial fibrosis/tubular atrophy; Computational whole-slide interstitial fibrosis	Per 10 percentage points	OR 5.69 (1.79 to 28.93)	Baseline-adjusted	57	Unknown	k=1; SWiM
Carta, 2013 (distinct cohort)	All-cause graft failure; longest eligible follow-up	Karpinski/MAPI score; Karpinski	score 4-5 versus score 1-3	OR 1.26 (0.53 to 3.01)	Crude; recalculated	100	Unknown	k=1; SWiM

Study/cohort	Outcome and horizon	Lesion or score	Contrast or unit	Effect (95% CI)	Adjustment	Analytic n	QUIPS	Cell k and route
Centellas-Pérez, 2019 (2011 to 2015)	All-cause graft failure; longest eligible follow-up	Karpinski/MAPI score; Study-specific: Karpinski MAPI unresolved	4-5 vs 0-3	OR 1.57 (0.62 to 3.99)	Crude; recalculated	183	High	k=1; SWiM
Fernández-Lorente, 2012	All-cause graft failure; longest eligible follow-up	Remuzzi score	score >=5 versus <=3	HR 0.74 (0.44 to 1.23)	Crude; reported	NR	Unknown	k=1; SWiM
Assis-Borba, 2014 (distinct cohort)	All-cause graft failure; longest eligible follow-up	Remuzzi score; Study-specific: Four compartment semiquantitative preimplant score	moderate 4-6 or severe >=7/3 in one category versus mild 0-3	HR 1.30 (0.70 to 2.30)	Crude; reported	372	Unknown	k=1; SWiM
Hofer, 2014 (Vienna General Hosp.…)	All-cause graft failure; longest eligible follow-up	Tubular atrophy	one-grade increase on 0-3 component score	HR 1.05 (0.80 to 1.40)	Crude; reported	628 primary; 529 sensitivity	Moderate	k=2; forest; no pooled estimate
Sánchez-Escuredo, 2017 (Hosp. Clínic Barcelona…)	All-cause graft failure; longest eligible follow-up	Tubular atrophy	per tubular atrophy Remuzzi grade 0-3	HR 1.72 (0.88 to 3.35)	Baseline-adjusted	364	High	k=2; forest; no pooled estimate
Marletta, 2023 (distinct cohort)	All-cause graft failure; longest eligible follow-up	Tubular atrophy; Computational whole-slide tubular atrophy	Per 10 percentage points	OR 9.31 (1.48 to 226.6)	Baseline-adjusted	57	Unknown	k=1; SWiM
Kayler, 2008 (University of Pittsburgh…)	All-cause graft failure; longest eligible follow-up	Vascular injury	>=25% versus <25%	OR 2.41 (1.36 to 4.30)	Crude; reported	597	High	k=1; SWiM
Stewart, 2022 (US OPTN national registry)	All-cause graft failure; longest eligible follow-up	Vascular injury	mild-moderate/severe versus absent/minimal	HR 1.02 (0.90 to 1.17)	Baseline-adjusted	5,997	Unknown	k=1; SWiM
Hofer, 2014 (Vienna General Hosp.…)	All-cause graft failure; longest eligible follow-up	Vascular injury	one-grade increase on 0-3 component score	HR 0.98 (0.78 to 1.24)	Crude; reported	628 primary; 529 sensitivity	Moderate	k=2; forest; no pooled estimate
Sánchez-Escuredo, 2017 (Hosp. Clínic Barcelona…)	All-cause graft failure; longest eligible follow-up	Vascular injury	per RS grade increment (0-3, wall thickness vs lumen)	HR 1.37 (0.68 to 2.77)	Baseline-adjusted	364	High	k=2; forest; no pooled estimate
Rohr, 1983	DGF; principal DGF definition	Acute tubular injury	ATN apparent vs absent	OR 49.08 (9.89 to 243.6)	Crude; recalculated	NR	High	k=5; principal pool
Perez-Gutierrez, 2021 (distinct cohort)	DGF; principal DGF definition	Acute tubular injury	Banff none/mild/moderate/severe, per category increase	OR 1.10 (0.39 to 2.43)	Baseline-adjusted; covariates incomplete	118/228 total	High	k=2; forest; no pooled estimate
Li, 2024 (First Affiliated Hosp. of…)	DGF; principal DGF definition	Acute tubular injury	moderate/severe versus mild	OR 8.42 (4.22 to 17.22)	Baseline-adjusted	492	High	k=5; principal pool
Mourão, 2016	DGF; principal DGF definition	Acute tubular injury	moderate/severe vs mild/none	OR 2.15 (0.45 to 10.29)	Crude; recalculated	NR	High	k=5; principal pool
Zheng, 2020 (distinct cohort)	DGF; principal DGF definition	Acute tubular injury	per one-point increase	OR 4.72 (2.32 to 9.60)	Baseline-adjusted	NR	High	k=2; forest; no pooled estimate
Matignon, 2008 (Henri Mondor Hosp., Creteil…)	DGF; principal DGF definition	Acute tubular injury	present versus absent	OR 0.96 (0.46 to 1.99)	Crude; recalculated	172	Moderate	k=5; principal pool
Wang, 2022 (distinct cohort)	DGF; principal DGF definition	Acute tubular injury	severe versus mild	OR 5.31 (1.75 to 16.42)	Baseline-adjusted	140	High	k=5; principal pool
Chen, 2021 (2012 to 2014)	DGF; principal DGF definition	Chronic composite score; BANFF	6-12 versus 0-2 (Table 3 prints overlapping 0-3)	OR 0.73 (0.28 to 1.87)	Crude; reported	173	High	k=2; forest; no pooled estimate
Tan, 2024 (First Affiliated Hosp. of…)	DGF; principal DGF definition	Chronic composite score; BANFF	M-S grade vs Mild/No grade	OR 0.99 (0.85 to 1.15)	Baseline-adjusted	97	High	k=2; forest; no pooled estimate
Tan, 2024 (distinct cohort)	DGF; principal DGF definition	Chronic composite score; CADI	moderate-severe versus mild/no grade	OR 1.10 (0.84 to 1.42)	Baseline-adjusted	97	Unknown	k=1; SWiM
Tan, 2024 (distinct cohort)	DGF; principal DGF definition	Chronic composite score; DDS	moderate-severe versus mild/no grade	OR 1.08 (0.86 to 1.37)	Baseline-adjusted	97	Unknown	k=1; SWiM
Tan, 2024 (distinct cohort)	DGF; principal DGF definition	Chronic composite score; Pirani	moderate-severe versus mild/no grade	OR 1.14 (0.93 to 1.39)	Baseline-adjusted	97	Unknown	k=1; SWiM
Azancot, 2014 (distinct cohort)	DGF; principal DGF definition	Chronic composite score; Study-specific: BANFF chronic donor score 0-15	score 4-5 or 6-7 versus <=3	OR 1.71 (CI not reported)	Crude; recalculated	117	Unknown	k=1; SWiM
Di Paolo, 2002 (distinct cohort)	DGF; principal DGF definition	Chronic composite score; Study-specific: Four compartment	Continuous, per 1-point increase in global score	OR 1.10 (0.94 to 1.27)	Baseline-adjusted	100	High	k=1; SWiM
Di Paolo, 2002 (distinct cohort)	DGF; principal DGF definition	Chronic composite score; Study-specific: Four compartment	Per point within score >4	OR 1.20 (0.83 to 1.73)	Baseline-adjusted	100	High	k=1; SWiM

Study/cohort	Outcome and horizon	Lesion or score	Contrast or unit	Effect (95% CI)	Adjustment	Analytic n	QUIPS	Cell k and route
Di Paolo, 2002 (distinct cohort)	DGF; principal DGF definition	Chronic composite score; Study-specific: Four compartment	per one-grade or one-point increase	OR 1.35 (0.86 to 2.12)	Baseline-adjusted	100 (48 DGF; 52 no DGF)	High	k=1; SWiM
Husain, 2021 (distinct cohort)	DGF; principal DGF definition	Chronic composite score; Study-specific: Local three compartment	suboptimal versus optimal	OR 1.25 (0.79 to 1.98)	Crude; recalculated	NR	Unknown	k=1; SWiM
Patel, 2012 (2006-05 to 2010-12)	DGF; principal DGF definition	Chronic composite score; Study-specific: Multi instrument unresolved	GS >=10% of glomeruli or arteriosclerosis	OR 0.63 (0.19 to 2.13)	Crude; recalculated	47	High	k=1; SWiM
Li, 2024 (First Affiliated Hosp. of…)	DGF; principal DGF definition	Chronic composite score; Study-specific: Multi instrument unresolved	continuous per 1 point	OR 1.13 (0.96 to 1.33)	Baseline-adjusted	492	High	k=1; SWiM
Jacobi, 2016 (distinct cohort)	DGF; principal DGF definition	Chronic composite score; Study-specific: Multi instrument unresolved	high score band versus 0-5; text >5, figure legend >6	OR 0.85 (0.21 to 3.46)	Crude; recalculated	200	High	k=1; SWiM
Tan, 2024 (distinct cohort)	DGF; principal DGF definition	Chronic composite score; UGARTE	moderate-severe versus mild/no grade	OR 1.12 (0.96 to 1.30)	Baseline-adjusted	97	Unknown	k=1; SWiM
Teixeira, 2021 (2014-07 to 2017-12)	DGF; principal DGF definition	Glomerulosclerosis	>15% glomerulosclerosis	OR 1.79 (0.36 to 8.81)	Baseline-adjusted	159	High	k=8; principal pool
Matignon, 2008 (Henri Mondor Hosp., Creteil…)	DGF; principal DGF definition	Glomerulosclerosis	>20% versus <=20%	OR 1.59 (0.60 to 4.21)	Crude; recalculated	172	Moderate	k=8; principal pool
Lee, 2026 (US OPTN/STAR national…)	DGF; principal DGF definition	Glomerulosclerosis	GS >20%	OR 0.90 (0.73 to 1.12)	Baseline-adjusted	13,962	High	k=8; principal pool
Cheungpasitporn, 2020 (US OPTN/UNOS national…)	DGF; principal DGF definition	Glomerulosclerosis	GS >20% vs 11-20%	OR 0.82 (0.65 to 1.05)	Baseline-adjusted	1,730	High	k=8; principal pool
Lu, 2000	DGF; principal DGF definition	Glomerulosclerosis	GS >=20% vs <20%	OR 0.37 (0.08 to 1.80)	Crude; recalculated	89	Unknown	k=8; principal pool
Lubuska, 2006 (1994 to 1997)	DGF; principal DGF definition	Glomerulosclerosis	GS >=20% vs <20%	OR 0.30 (0.03 to 2.68)	Crude; recalculated	NR	High	k=8; principal pool
Di Paolo, 2002 (distinct cohort)	DGF; principal DGF definition	Glomerulosclerosis	Per 1-grade increase (0-3 scale)	OR 1.68 (0.88 to 3.19)	Baseline-adjusted	100	High	k=1; SWiM
Lopes, 2005 (Bellvitge Hosp., Barcelona)	DGF; principal DGF definition	Glomerulosclerosis	Per 10 percentage points	RR 1.79 (1.10 to 3.39)	Crude; reported	77	High	k=1; SWiM
Perez-Gutierrez, 2021 (distinct cohort)	DGF; principal DGF definition	Glomerulosclerosis	Per 10 percentage points	OR 1.34 (0.39 to 4.81)	Baseline-adjusted; covariates incomplete	118	High	k=1; SWiM
Wang, 2022 (2012-01 to 2017-06)	DGF; principal DGF definition	Glomerulosclerosis	Present vs absent	OR 1.42 (0.56 to 3.44)	Crude; reported	140	High	k=8; principal pool
Li, 2024 (First Affiliated Hosp. of…)	DGF; principal DGF definition	Glomerulosclerosis	moderate versus none	OR 8.03 (2.07 to 31.18)	Crude; recalculated	492	High	k=8; principal pool
Lee, 2026 (US OPTN/STAR national…)	DGF; principal DGF definition	Glomerulosclerosis and machine perfusion; Machine-perfusion stratified: GS MPP joint	>20% GS with poor MPP versus 0%-10% GS with good MPP	RR 1.33 (1.09 to 1.63)	Crude; reported	13,962	High	k=1; SWiM
Lee, 2026 (US OPTN/STAR national…)	DGF; principal DGF definition	Glomerulosclerosis; Machine-perfusion stratified: GS	>20% GS with good MPP versus 0%-10% GS with good MPP	RR 0.96 (0.81 to 1.13)	Crude; reported	13,962	High	k=1; SWiM
Lee, 2026 (US OPTN/STAR national…)	DGF; principal DGF definition	Interstitial fibrosis/tubular atrophy	IFTA more-than-mild	OR 0.79 (0.62 to 1.00)	Baseline-adjusted	13,962	High	k=6; principal pool
Di Paolo, 2002 (distinct cohort)	DGF; principal DGF definition	Interstitial fibrosis/tubular atrophy	Per 1-grade increase	OR 0.62 (0.24 to 1.59)	Baseline-adjusted	100	High	k=1; SWiM
Pavlovic, 2022 (distinct cohort)	DGF; principal DGF definition	Interstitial fibrosis/tubular atrophy	Per 10 percentage points	OR 2.59 (1.12 to 6.09)	Baseline-adjusted	73	High	k=1; SWiM
Wang, 2022 (2012-01 to 2017-06)	DGF; principal DGF definition	Interstitial fibrosis/tubular atrophy	Present vs absent	OR 1.39 (0.36 to 4.40)	Crude; reported	140	High	k=6; principal pool
Lubuska, 2006 (1994 to 1997)	DGF; principal DGF definition	Interstitial fibrosis/tubular atrophy	any interstitial fibrosis vs none	OR 2.00 (0.91 to 4.40)	Crude; recalculated	NR	High	k=6; principal pool
Li, 2024 (First Affiliated Hosp. of…)	DGF; principal DGF definition	Interstitial fibrosis/tubular atrophy	moderate versus none	OR 8.03 (2.07 to 31.18)	Crude; recalculated	492	High	k=6; principal pool

Study/cohort	Outcome and horizon	Lesion or score	Contrast or unit	Effect (95% CI)	Adjustment	Analytic n	QUIPS	Cell k and route
Matignon, 2008 (Henri Mondor Hosp., Creteil…)	DGF; principal DGF definition	Interstitial fibrosis/tubular atrophy	present versus absent	OR 1.56 (0.74 to 3.32)	Crude; recalculated	172	Moderate	k=6; principal pool
Teixeira, 2021 (2014-07 to 2017-12)	DGF; principal DGF definition	Interstitial fibrosis/tubular atrophy	present versus absent	OR 3.16 (0.82 to 12.13)	Baseline-adjusted	159	High	k=6; principal pool
Pavlovic, 2022 (distinct cohort)	DGF; principal DGF definition	Interstitial fibrosis/tubular atrophy; Morphometric Sirius red area (frozen section) with interstitial-fibrosis score	Per 10 percentage points (numerically unstable)	OR 2.53e-03 (2.59e-10 to 2.06e04)	Baseline-adjusted	73	High	k=1; SWiM
Pavlovic, 2022 (distinct cohort)	DGF; principal DGF definition	Interstitial fibrosis/tubular atrophy; Morphometric Sirius red area (paraffin section) with interstitial-fibrosis score	Per 10 percentage points (numerically unstable)	OR 2.06e04 (1.02e-07 to 2.06e14)	Baseline-adjusted	73	High	k=1; SWiM
Pavlovic, 2022 (distinct cohort)	DGF; principal DGF definition	Interstitial fibrosis/tubular atrophy; Morphometric Sirius red interstitial area (frozen section)	Per 10 percentage points	OR 1.10 (0.54 to 2.59)	Baseline-adjusted	73	High	k=1; SWiM
Pavlovic, 2022 (distinct cohort)	DGF; principal DGF definition	Interstitial fibrosis/tubular atrophy; Morphometric Sirius red interstitial area (paraffin section)	Per 10 percentage points	OR 2.84 (1.24 to 6.73)	Crude; reported	73	High	k=1; SWiM
Zanchetta, 2025 (distinct cohort)	DGF; principal DGF definition	Karpinski/MAPI score; Karpinski	score 5 versus score 4	OR 0.97 (0.42 to 2.24)	Crude; recalculated	NR	High	k=1; SWiM
Chen, 2021 (2012 to 2014)	DGF; principal DGF definition	Karpinski/MAPI score; MAPI	7-9 versus 0-3	OR 1.16 (0.33 to 4.06)	Crude; reported	173	High	k=1; SWiM
Centellas-Pérez, 2019 (2011 to 2015)	DGF; principal DGF definition	Karpinski/MAPI score; Study-specific: Karpinski MAPI unresolved	4-5 vs 0-3	OR 1.30 (0.56 to 3.02)	Crude; recalculated	183	High	k=1; SWiM
Tan, 2024 (First Affiliated Hosp. of…)	DGF; principal DGF definition	Karpinski/MAPI score; Study-specific: Karpinski MAPI unresolved	M-S grade vs Mild/No grade	OR 1.59 (1.24 to 2.04)	Baseline-adjusted	97	High	k=1; SWiM
Chen, 2021 (2012 to 2014)	DGF; principal DGF definition	Leuven score; Leuven	>60 versus <60	OR 1.93 (0.85 to 4.39)	Crude; reported	173	High	k=1; SWiM
Teixeira, 2021 (2014-07 to 2017-12)	DGF; principal DGF definition	MAPI periglomerular-fibrosis component; Routine: MAPI component PGF	periglomerular fibrosis present vs absent	OR 1.37 (0.15 to 12.76)	Baseline-adjusted	159	High	k=1; SWiM
Li, 2024 (First Affiliated Hosp. of…)	DGF; principal DGF definition	Other acute lesion; Arteriolar smooth-muscle vacuolar degeneration	mild versus none	OR 2.52 (1.07 to 5.92)	Crude; recalculated	492	High	k=1; SWiM
Lubuska, 2006 (1994 to 1997)	DGF; principal DGF definition	Other acute lesion; Routine: Acute other brush border loss	loss vs preserved	OR 3.08 (1.26 to 7.67)	Crude; reported	NR	High	k=1; SWiM
Lubuska, 2006 (1994 to 1997)	DGF; principal DGF definition	Other acute lesion; Routine: Acute other hemoglobin cylinders	present vs absent	OR 4.78 (1.92 to 12.02)	Crude; reported	NR	High	k=1; SWiM
Matignon, 2008 (Henri Mondor Hosp., Creteil…)	DGF; principal DGF definition	Other acute lesion; Routine: Acute other osmotic nephrosis present	present versus absent	OR 1.23 (0.64 to 2.35)	Crude; recalculated	172	Moderate	k=1; SWiM
Lubuska, 2006 (1994 to 1997)	DGF; principal DGF definition	Other acute lesion; Routine: Acute other tubular dilatation	present vs absent	OR 0.53 (0.20 to 1.38)	Crude; reported	NR	High	k=1; SWiM
Lubuska, 2006 (1994 to 1997)	DGF; principal DGF definition	Other acute lesion; Routine: Acute other vacuolar dystrophy	any vacuolar dystrophy vs none	OR 1.46 (0.56 to 3.86)	Crude; reported	NR	High	k=1; SWiM
Li, 2024 (First Affiliated Hosp. of…)	DGF; principal DGF definition	Other chronic lesion; Routine: Chronic other mesangial matrix hyperplasia grade	moderate versus none	OR 3.15 (1.04 to 9.52)	Crude; recalculated	492	High	k=1; SWiM
Tan, 2024 (First Affiliated Hosp. of…)	DGF; principal DGF definition	Remuzzi score	Moderate/severe grade versus mild grade	OR 1.20 (0.96 to 1.50)	Baseline-adjusted	97	High	k=3; exploratory pool
Chen, 2021 (2012 to 2014)	DGF; principal DGF definition	Remuzzi score	Remuzzi 4-6 vs 0-3	OR 0.83 (0.38 to 1.82)	Crude; reported	173	High	k=3; exploratory pool
Pavlovic, 2022 (distinct cohort)	DGF; principal DGF definition	Remuzzi score	Remuzzi interstitial-fibrosis score (median 2; range 1-2)	OR 0.72 (0.28 to 1.82)	Crude; reported	73	High	k=3; exploratory pool
Losappio, 2014 (University of Bari…)	DGF; principal DGF definition	Remuzzi score	Score ordinal 0 vs 1-4 vs 5 (per category step)	OR 1.92 (1.02 to 3.60)	Baseline-adjusted	372	High	k=2; forest; no pooled estimate
Zheng, 2020 (2018-01 to 2019-09)	DGF; principal DGF definition	Remuzzi score	continuous, per 1-point increase	OR 1.21 (1.02 to 1.43)	Baseline-adjusted	333	High	k=2; forest; no pooled estimate

Study/cohort	Outcome and horizon	Lesion or score	Contrast or unit	Effect (95% CI)	Adjustment	Analytic n	QUIPS	Cell k and route
Weissenbacher, 2019	DGF; principal DGF definition	Remuzzi score	per 1 point	OR 1.04 (0.71 to 1.52)	Baseline-adjusted	NR	High	k=2; forest; no pooled estimate
Pavlovic, 2022 (distinct cohort)	DGF; principal DGF definition	Remuzzi score	per one grade	OR 1.50 (0.54 to 4.15)	Crude; reported	73	High	k=2; forest; no pooled estimate
Perez-Gutierrez, 2021 (distinct cohort)	DGF; principal DGF definition	Tubular atrophy	Banff none/mild/moderate/severe, per category increase	OR 0.40 (0.05 to 3.01)	Baseline-adjusted; covariates incomplete	118/228 total	High	k=2; forest; no pooled estimate
Li, 2024 (First Affiliated Hosp. of…)	DGF; principal DGF definition	Tubular atrophy	mild versus none	OR 1.28 (0.75 to 2.18)	Crude; recalculated	492	High	k=2; forest; no pooled estimate
Di Paolo, 2002 (distinct cohort)	DGF; principal DGF definition	Tubular atrophy	per one-grade or one-point increase	OR 1.27 (0.48 to 3.39)	Baseline-adjusted	100 (48 DGF; 52 no DGF)	High	k=2; forest; no pooled estimate
Wang, 2022 (distinct cohort)	DGF; principal DGF definition	Tubular atrophy	present versus absent	OR 2.05 (0.79 to 5.10)	Crude; reported	140	High	k=2; forest; no pooled estimate
Lee, 2016 (2006-12 to 2011-07)	DGF; principal DGF definition	Vascular injury	Banff AH >=1	OR 2.80 (1.19 to 6.37)	Baseline-adjusted	135	Moderate	k=7; principal pool
Perez-Gutierrez, 2021 (distinct cohort)	DGF; principal DGF definition	Vascular injury	Banff none/mild/mod/severe	OR 6.31 (1.66 to 24.01)	Baseline-adjusted; covariates incomplete	118	High	k=3; exploratory pool
Di Paolo, 2002 (distinct cohort)	DGF; principal DGF definition	Vascular injury	Per 1-grade increase	OR 1.81 (0.93 to 3.52)	Baseline-adjusted	100	High	k=3; exploratory pool
Wang, 2022 (2012-01 to 2017-06)	DGF; principal DGF definition	Vascular injury	Present vs absent	OR 1.48 (0.62 to 3.48)	Crude; reported	140	High	k=7; principal pool
Matignon, 2008 (Henri Mondor Hosp., Creteil…)	DGF; principal DGF definition	Vascular injury	ah+ (grade>=1) vs ah- (grade 0)	OR 2.20 (1.10 to 4.50)	Baseline-adjusted	172	Moderate	k=7; principal pool
Lubuska, 2006 (1994 to 1997)	DGF; principal DGF definition	Vascular injury	any arterial damage vs none	OR 1.55 (0.60 to 4.05)	Crude; reported	NR	High	k=7; principal pool
Zheng, 2020 (2018-01 to 2019-09)	DGF; principal DGF definition	Vascular injury	continuous, per 1-point increase (0-3)	OR 1.82 (1.18 to 2.81)	Crude; reported	333	High	k=3; exploratory pool
Li, 2024 (First Affiliated Hosp. of…)	DGF; principal DGF definition	Vascular injury	moderate versus none	OR 4.42 (2.38 to 8.20)	Crude; recalculated	492	High	k=7; principal pool
Teixeira, 2021 (2014-07 to 2017-12)	DGF; principal DGF definition	Vascular injury	present versus absent	OR 1.13 (0.36 to 3.54)	Baseline-adjusted	159	High	k=7; principal pool
Lee, 2026 (US OPTN/STAR national…)	DGF; principal DGF definition	Vascular injury	vascular more-than-mild vs none-to-mild	OR 1.05 (0.86 to 1.27)	Baseline-adjusted	13,962	High	k=7; principal pool
Munivenkatappa, 2008 (distinct cohort)	Graft failure (death-censored/unclear); longest eligible follow-up	Acute tubular injury	any versus absent	HR 1.03 (0.53 to 2.02)	Crude; reported	259	High	k=1; SWiM
Scurt, 2023	Graft failure (death-censored/unclear); longest eligible follow-up	Chronic composite score; Navarro	Navarro Full Score; continuous per 1-point increase	HR 1.50 (1.14 to 1.97)	Crude; reported	223	High	k=1; SWiM
Martín, 2018	Graft failure (death-censored/unclear); longest eligible follow-up	Chronic composite score; Study-specific: Andalusian histological score	per point	HR 1.01 (0.72 to 1.39)	Crude; reported	114	High	k=1; SWiM
Lopes, 2005 (Bellvitge Hosp., Barcelona)	Graft failure (death-censored/unclear); longest eligible follow-up	Chronic composite score; Study-specific: Chronic damage score	per score point	RR 2.20 (1.50 to 3.30)	Crude; reported	77	High	k=1; SWiM
Lopes, 2005 (Bellvitge Hosp., Barcelona)	Graft failure (death-censored/unclear); longest eligible follow-up	Chronic composite score; Study-specific: Donor chronic damage score GS cv ci	per score point; GS >10% + cv + ci	RR 3.90 (1.70 to 8.90)	Baseline-adjusted; covariates incomplete	77	High	k=1; SWiM
Lu, 2001	Graft failure (death-censored/unclear); longest eligible follow-up	Chronic composite score; Study-specific: Generic score	high-risk vs low-risk histology	OR 1.65 (0.29 to 9.29)	Crude; recalculated	89	Unknown	k=1; SWiM

Study/cohort	Outcome and horizon	Lesion or score	Contrast or unit	Effect (95% CI)	Adjustment	Analytic n	QUIPS	Cell k and route
Navarro, 2011	Graft failure (death-censored/unclear); longest eligible follow-up	Chronic composite score; Study-specific: Generic score	total histologic score >5 vs <=5	HR 6.95 (1.57 to 30.73)	Baseline-adjusted	136	Unknown	k=1; SWiM
Husain, 2021 (distinct cohort)	Graft failure (death-censored/unclear); longest eligible follow-up	Chronic composite score; Study-specific: Local three compartment	optimal versus suboptimal	HR 1.14 (0.69 to 1.85)	Baseline-adjusted	NR	Unknown	k=1; SWiM
Patel, 2012 (2006-05 to 2010-12)	Graft failure (death-censored/unclear); longest eligible follow-up	Chronic composite score; Study-specific: Multi instrument unresolved	GS >=10% or arteriosclerosis >=10%	OR 6.50 (0.67 to 63.43)	Crude; recalculated	47	High	k=1; SWiM
Husain, 2022	Graft failure (death-censored/unclear); longest eligible follow-up	Chronic composite score; Study-specific: Optimal suboptimal	Suboptimal: GS >=11%, IFTA >=mild, or vascular injury	HR 1.17 (1.07 to 1.29)	Baseline-adjusted	30,469	High	k=1; SWiM
Husain, 2021 (distinct cohort)	Graft failure (death-censored/unclear); longest eligible follow-up	Chronic composite score; Study-specific: Star three compartment	optimal versus suboptimal	HR 1.11 (0.81 to 1.52)	Baseline-adjusted	NR	Unknown	k=1; SWiM
Teixeira, 2021 (2014-07 to 2017-12)	Graft failure (death-censored/unclear); longest eligible follow-up	Glomerulosclerosis	>15% glomerulosclerosis	HR 1.93 (0.20 to 17.10)	Baseline-adjusted	159	High	k=9; principal pool
Lee, 2026 (US OPTN/STAR national…)	Graft failure (death-censored/unclear); longest eligible follow-up	Glomerulosclerosis	>20% versus 0%-10%	HR 1.54 (1.17 to 2.03)	Baseline-adjusted	13,962	High	k=9; principal pool
Reese, 2021	Graft failure (death-censored/unclear); longest eligible follow-up	Glomerulosclerosis	>20% versus 0-5%	HR 1.83 (1.35 to 2.47)	Crude; reported	1,629 development; 1,107 validation	Moderate	k=9; principal pool
Pegas, 2018 (2006 to 2013)	Graft failure (death-censored/unclear); longest eligible follow-up	Glomerulosclerosis	>25% versus 0%	HR 10.68 (4.09 to 27.90)	Crude; reported	430	High	k=9; principal pool
Lin, 2010	Graft failure (death-censored/unclear); longest eligible follow-up	Glomerulosclerosis	Advanced glomerulosclerosis (not quantified)	HR 2.89 (1.02 to 8.17)	Crude; reported	55	High	k=9; principal pool
Villanego, 2022 (2010-01 to 2019-06)	Graft failure (death-censored/unclear); longest eligible follow-up	Glomerulosclerosis	Component score >=1 vs 0	HR 2.86 (1.33 to 6.15)	Baseline-adjusted	266	High	k=9; principal pool
Chen, 2021 (2012 to 2014)	Graft failure (death-censored/unclear); longest eligible follow-up	Glomerulosclerosis	GS >10% (vs <=10%)	HR 1.49 (0.47 to 4.70)	Crude; reported	173	High	k=9; principal pool
Cheungpasitporn, 2020 (US OPTN/UNOS national…)	Graft failure (death-censored/unclear); longest eligible follow-up	Glomerulosclerosis	GS >20% vs 11-20%	HR 0.95 (0.78 to 1.15)	Baseline-adjusted	1,730	High	k=9; principal pool
Munivenkatappa, 2008 (1999-01 to 2005-12)	Graft failure (death-censored/unclear); longest eligible follow-up	Glomerulosclerosis	GS >=15%	HR 1.87 (1.17 to 2.99)	Crude; reported	259	High	k=9; principal pool
Munivenkatappa, 2008 (distinct cohort)	Graft failure (death-censored/unclear); longest eligible follow-up	Glomerulosclerosis	Per 10 percentage points	HR 1.48 (1.10 to 1.97)	Crude; reported	259	High	k=2; forest; no pooled estimate
Pegas, 2018 (2006 to 2013)	Graft failure (death-censored/unclear); longest eligible follow-up	Glomerulosclerosis	Per 10 percentage points	HR 1.63 (1.34 to 1.97)	Crude; reported	430	High	k=2; forest; no pooled estimate
Lee, 2026 (US OPTN/STAR national…)	Graft failure (death-censored/unclear); longest eligible follow-up	Glomerulosclerosis and machine perfusion; Machine-perfusion stratified: GS MPP joint	>20% GS with poor MPP versus 0%-10% GS with good MPP	HR 1.75 (1.07 to 2.87)	Crude; reported	13,962	High	k=1; SWiM

Study/cohort	Outcome and horizon	Lesion or score	Contrast or unit	Effect (95% CI)	Adjustment	Analytic n	QUIPS	Cell k and route
Lee, 2026 (US OPTN/STAR national…)	Graft failure (death-censored/unclear); longest eligible follow-up	Glomerulosclerosis; Machine-perfusion stratified: GS	>20% GS with good MPP versus 0%-10% GS with good MPP	HR 1.98 (1.47 to 2.67)	Crude; reported	13,962	High	k=1; SWiM
Reese, 2021	Graft failure (death-censored/unclear); longest eligible follow-up	Interstitial fibrosis/tubular atrophy	IFTA Banff >=2	HR 1.51 (1.00 to 2.26)	Baseline-adjusted	1,629	Moderate	k=6; principal pool
Pegas, 2018 (2006 to 2013)	Graft failure (death-censored/unclear); longest eligible follow-up	Interstitial fibrosis/tubular atrophy	Moderate (26-50%)	HR 4.15 (1.81 to 9.54)	Crude; reported	430	High	k=6; principal pool
Munivenkatappa, 2008 (1999-01 to 2005-12)	Graft failure (death-censored/unclear); longest eligible follow-up	Interstitial fibrosis/tubular atrophy	Sclerotic focus with fibrosis present vs absent	HR 2.58 (1.24 to 5.38)	Crude; reported	259	High	k=6; principal pool
Lubuska, 2006 (1994 to 1997)	Graft failure (death-censored/unclear); longest eligible follow-up	Interstitial fibrosis/tubular atrophy	any interstitial fibrosis vs none	OR 0.44 (0.13 to 1.47)	Crude; reported	NR	High	k=1; SWiM
Lee, 2026 (US OPTN/STAR national…)	Graft failure (death-censored/unclear); longest eligible follow-up	Interstitial fibrosis/tubular atrophy	more than mild versus none to mild	HR 1.38 (1.01 to 1.89)	Baseline-adjusted	13,962	High	k=6; principal pool
Chen, 2021 (2012 to 2014)	Graft failure (death-censored/unclear); longest eligible follow-up	Interstitial fibrosis/tubular atrophy	presence versus absence	HR 0.64 (0.21 to 1.92)	Crude; reported	173	High	k=6; principal pool
Villanego, 2022 (2010-01 to 2019-06)	Graft failure (death-censored/unclear); longest eligible follow-up	Interstitial fibrosis/tubular atrophy	score >=1 versus 0	HR 1.30 (0.59 to 2.89)	Crude; reported	266	High	k=6; principal pool
Munivenkatappa, 2008 (distinct cohort)	Graft failure (death-censored/unclear); longest eligible follow-up	Interstitial fibrosis/tubular atrophy; Computer-colourimetric cumulative fibrosis	Per 10 percentage points	HR 1.22 (0.90 to 1.63)	Crude; reported	259	High	k=1; SWiM
Carta, 2013 (distinct cohort)	Graft failure (death-censored/unclear); longest eligible follow-up	Karpinski/MAPI score; Karpinski	score 4-5 versus score 1-3	OR 1.67 (0.47 to 5.94)	Crude; recalculated	100	Unknown	k=2; forest; no pooled estimate
Zanchetta, 2025 (distinct cohort)	Graft failure (death-censored/unclear); longest eligible follow-up	Karpinski/MAPI score; Karpinski	score 5 versus score 4	OR 1.02 (0.28 to 3.66)	Crude; recalculated	NR	High	k=2; forest; no pooled estimate
Philosophe, 2014	Graft failure (death-censored/unclear); longest eligible follow-up	Karpinski/MAPI score; MAPI	MAPI 12-15 versus 0-7	HR 6.58 (3.12 to 13.88)	Crude; reported	196	Unknown	k=1; SWiM
Lubuska, 2006 (1994 to 1997)	Graft failure (death-censored/unclear); longest eligible follow-up	Other acute lesion; Routine: Acute other brush border loss	loss vs preserved	OR 0.55 (0.16 to 1.83)	Crude; reported	NR	High	k=1; SWiM
Lubuska, 2006 (1994 to 1997)	Graft failure (death-censored/unclear); longest eligible follow-up	Other acute lesion; Routine: Acute other hemoglobin cylinders	present vs absent	OR 0.38 (0.09 to 1.44)	Crude; reported	NR	High	k=1; SWiM
Lubuska, 2006 (1994 to 1997)	Graft failure (death-censored/unclear); longest eligible follow-up	Other acute lesion; Routine: Acute other tubular dilatation	present vs absent	OR 0.64 (0.17 to 2.49)	Crude; reported	NR	High	k=1; SWiM
Lubuska, 2006 (1994 to 1997)	Graft failure (death-censored/unclear); longest eligible follow-up	Other acute lesion; Routine: Acute other vacuolar dystrophy	any vacuolar dystrophy vs none	OR 0.74 (0.21 to 2.62)	Crude; reported	NR	High	k=1; SWiM
Jiang, 2025	Graft failure (death-censored/unclear); longest eligible follow-up	Remuzzi score	per one-point increase in Remuzzi score	HR 1.70 (1.19 to 2.30)	Baseline-adjusted	256	High	k=1; SWiM
Assis-Borba, 2014 (distinct cohort)	Graft failure (death-censored/unclear); longest eligible follow-up	Remuzzi score; Study-specific: Four compartment semiquantitative preimplant score	moderate 4-6 or severe >=7/3 in one category versus mild 0-3	HR 1.90 (0.90 to 3.90)	Crude; reported	372	Unknown	k=1; SWiM

Study/cohort	Outcome and horizon	Lesion or score	Contrast or unit	Effect (95% CI)	Adjustment	Analytic n	QUIPS	Cell k and route
Villanego, 2022 (2010-01 to 2019-06)	Graft failure (death-censored/unclear); longest eligible follow-up	Remuzzi score; Study-specific: Spanish five compartment preimplant score	>3 versus <=3	HR 1.41 (0.68 to 2.94)	Baseline-adjusted	266	High	k=1; SWiM
Chen, 2021 (2012 to 2014)	Graft failure (death-censored/unclear); longest eligible follow-up	Tubular atrophy	Present vs absent	HR 0.65 (0.22 to 1.94)	Crude; reported	173	High	k=3; exploratory pool
Pegas, 2018 (2006 to 2013)	Graft failure (death-censored/unclear); longest eligible follow-up	Tubular atrophy	moderate 26-50% versus absent	HR 4.74 (1.88 to 11.92)	Crude; reported	430	High	k=3; exploratory pool
Villanego, 2022 (2010-01 to 2019-06)	Graft failure (death-censored/unclear); longest eligible follow-up	Tubular atrophy	score >=1 versus 0	HR 0.66 (0.31 to 1.42)	Crude; reported	266	High	k=3; exploratory pool
Wazna, 2018	Graft failure (death-censored/unclear); longest eligible follow-up	Vascular injury	Arteriolar hyalinosis grade 1-3 vs 0	HR 1.94 (1.04 to 3.60)	Crude; reported	300	High	k=9; principal pool
Villanego, 2022 (2010-01 to 2019-06)	Graft failure (death-censored/unclear); longest eligible follow-up	Vascular injury	Component score >=1 vs 0	HR 2.32 (1.12 to 4.79)	Baseline-adjusted	266	High	k=9; principal pool
Chen, 2021 (2012 to 2014)	Graft failure (death-censored/unclear); longest eligible follow-up	Vascular injury	Present vs absent	HR 0.39 (0.13 to 1.12)	Crude; reported	173	High	k=9; principal pool
Munivenkatappa, 2008 (1999-01 to 2005-12)	Graft failure (death-censored/unclear); longest eligible follow-up	Vascular injury	WLR interlobular >=0.5	HR 2.05 (1.21 to 3.47)	Crude; reported	259	High	k=9; principal pool
Lubuska, 2006 (1994 to 1997)	Graft failure (death-censored/unclear); longest eligible follow-up	Vascular injury	any arterial damage vs none	OR 1.05 (0.35 to 4.22)	Crude; reported	NR	High	k=1; SWiM
Reese, 2021	Graft failure (death-censored/unclear); longest eligible follow-up	Vascular injury	high score >=2 versus low score 0 or 1	HR 1.49 (1.20 to 1.86)	Crude; reported	1,629 development; 1,107 validation	Moderate	k=9; principal pool
Stewart, 2022 (US OPTN national registry)	Graft failure (death-censored/unclear); longest eligible follow-up	Vascular injury	mild-moderate/severe versus absent/minimal	HR 1.30 (1.02 to 1.62)	Baseline-adjusted	5,997	Unknown	k=9; principal pool
Lee, 2026 (US OPTN/STAR national…)	Graft failure (death-censored/unclear); longest eligible follow-up	Vascular injury	more than mild versus none to mild	HR 1.06 (0.80 to 1.41)	Baseline-adjusted	13,962	High	k=9; principal pool
Teixeira, 2021 (2014-07 to 2017-12)	Graft failure (death-censored/unclear); longest eligible follow-up	Vascular injury	present versus absent	HR 3.14 (0.98 to 10.06)	Baseline-adjusted	159	High	k=9; principal pool
Pegas, 2018 (2006 to 2013)	Graft failure (death-censored/unclear); longest eligible follow-up	Vascular injury	severe >50% versus absent	HR 2.56 (0.84 to 7.74)	Crude; reported	430	High	k=9; principal pool
Proença, 2025 (distinct cohort)	Low eGFR; 9-15 months	Acute tubular injury	severe versus mild	OR 1.35 (0.41 to 4.50)	Crude; recalculated	339	Moderate	k=1; SWiM
Amenábar, 2016 (2008-03 to 2012-05)	Low eGFR; 9-15 months	Chronic composite score; Ovalle	score 6-8 vs 0-5	OR 1.99 (0.66 to 6.01)	Crude; recalculated	110	High	k=1; SWiM
Proença, 2025 (distinct cohort)	Low eGFR; 9-15 months	Cortical scarring; Routine: Cortical scarring	Per 10 percentage points (numerically unstable)	OR 2.86e04 (21.65 to 3.68e07)	Baseline-adjusted	339	Moderate	k=1; SWiM
Proença, 2025 (distinct cohort)	Low eGFR; 9-15 months	Glomerular thrombi; Routine: Glomerular thrombi	present versus absent	OR 0.37 (0.05 to 2.88)	Crude; recalculated	339	Moderate	k=1; SWiM
Teixeira, 2021 (2014-07 to 2017-12)	Low eGFR; 9-15 months	Glomerulosclerosis	>15% glomerulosclerosis	OR 0.71 (0.15 to 3.39)	Baseline-adjusted	159	High	k=2; forest; no pooled estimate

Study/cohort	Outcome and horizon	Lesion or score	Contrast or unit	Effect (95% CI)	Adjustment	Analytic n	QUIPS	Cell k and route
Lee, 2026 (US OPTN/STAR national…)	Low eGFR; 9-15 months	Glomerulosclerosis	>20% versus 0%-10%	OR 2.05 (1.39 to 3.02)	Baseline-adjusted	13,962	High	k=2; forest; no pooled estimate
Proença, 2025 (distinct cohort)	Low eGFR; 9-15 months	Glomerulosclerosis	Per 10 percentage points	OR 1.48 (1.10 to 2.16)	Crude; reported	339	Moderate	k=1; SWiM
Lee, 2026 (US OPTN/STAR national…)	Low eGFR; 9-15 months	Glomerulosclerosis and machine perfusion; Machine-perfusion stratified: GS MPP joint	>20% GS with poor MPP versus 0%-10% GS with good MPP	RR 3.67 (2.25 to 5.97)	Crude; reported	13,962	High	k=1; SWiM
Lee, 2026 (US OPTN/STAR national…)	Low eGFR; 9-15 months	Glomerulosclerosis; Machine-perfusion stratified: GS	>20% GS with good MPP versus 0%-10% GS with good MPP	RR 2.37 (1.60 to 3.51)	Crude; reported	13,962	High	k=1; SWiM
Proença, 2025 (distinct cohort)	Low eGFR; 9-15 months	Interstitial fibrosis/tubular atrophy	ci1 versus ci0	OR 3.43 (1.89 to 6.28)	Crude; reported	339	Moderate	k=3; exploratory pool
Lee, 2026 (US OPTN/STAR national…)	Low eGFR; 9-15 months	Interstitial fibrosis/tubular atrophy	more than mild versus none to mild	OR 1.16 (0.73 to 1.83)	Baseline-adjusted	13,962	High	k=3; exploratory pool
Teixeira, 2021 (2014-07 to 2017-12)	Low eGFR; 9-15 months	Interstitial fibrosis/tubular atrophy	present versus absent	OR 0.45 (0.13 to 1.54)	Baseline-adjusted	159	High	k=3; exploratory pool
Proença, 2025 (distinct cohort)	Low eGFR; 9-15 months	Karpinski/MAPI score; MAPI	8-11 versus 0-7	OR 4.08 (1.84 to 9.06)	Crude; recalculated	339	Moderate	k=1; SWiM
Teixeira, 2021 (2014-07 to 2017-12)	Low eGFR; 9-15 months	MAPI periglomerular-fibrosis component; Routine: MAPI component PGF	present versus absent	OR 1.39 (0.18 to 11.03)	Baseline-adjusted	159	High	k=1; SWiM
Proença, 2025 (distinct cohort)	Low eGFR; 9-15 months	Mesangial lesion; Routine: Mesangial	mm >1 versus <=1	OR 0.61 (0.07 to 4.99)	Crude; recalculated	339	Moderate	k=1; SWiM
Proença, 2025 (distinct cohort)	Low eGFR; 9-15 months	Other glomerular lesion; Routine: Glomerular other periglomerular fibrosis presence	present versus absent	OR 2.60 (1.11 to 6.08)	Crude; recalculated	339	Moderate	k=1; SWiM
Proença, 2025 (distinct cohort)	Low eGFR; 9-15 months	Remuzzi score	7-12 versus 0-3	OR 5.20 (1.02 to 26.61)	Baseline-adjusted	339	Moderate	k=1; SWiM
Proença, 2025 (distinct cohort)	Low eGFR; 9-15 months	Tubular atrophy	ct1 versus ct0	OR 3.44 (1.88 to 6.28)	Crude; recalculated	339	Moderate	k=1; SWiM
Proença, 2025 (distinct cohort)	Low eGFR; 9-15 months	Vascular injury	ah1 versus ah0	OR 2.18 (1.15 to 4.15)	Crude; reported	339	Moderate	k=3; exploratory pool
Lee, 2026 (US OPTN/STAR national…)	Low eGFR; 9-15 months	Vascular injury	more than mild versus none to mild	OR 1.75 (1.23 to 2.49)	Baseline-adjusted	13,962	High	k=3; exploratory pool
Teixeira, 2021 (2014-07 to 2017-12)	Low eGFR; 9-15 months	Vascular injury	present versus absent	OR 0.54 (0.18 to 1.63)	Baseline-adjusted	159	High	k=3; exploratory pool
Centellas-Pérez, 2019 (2011 to 2015)	Primary nonfunction; 1 year	Chronic composite score; Study-specific: BANFF biopsy score	score 4-5 versus 0-3	OR 4.70 (CI not reported)	Crude; recalculated	183	High	k=1; SWiM
Losappio, 2014 (University of Bari…)	Primary nonfunction; early, study-defined	Chronic composite score; Karpinski remuzzi hybrid	score 5 versus 0	OR 6.22 (0.83 to 46.35)	Crude; recalculated	372	High	k=1; SWiM
Jacobi, 2016 (distinct cohort)	Primary nonfunction; early, study-defined	Chronic composite score; Study-specific: Multi instrument unresolved	high score band versus 0-5; text >5, figure legend >6	OR 5.58 (1.07 to 29.09)	Crude; recalculated	NR	High	k=1; SWiM
Lee, 2026 (US OPTN/STAR national…)	Primary nonfunction; early, study-defined	Vascular injury	vascular more-than-mild	OR 1.40 (0.66 to 2.97)	Baseline-adjusted	13,962	High	k=1; SWiM
Husain, 2021 (2011-2020 (needle:…)	Primary nonfunction; up to 3 months	Chronic composite score; Study-specific: Local three compartment	Optimal vs suboptimal	OR 1.41 (0.84 to 2.37)	Crude; recalculated	2,033	High	k=1; SWiM
Lee, 2026 (US OPTN/STAR national…)	Primary nonfunction; up to 3 months	Glomerulosclerosis	>20% versus 0%-10%	OR 2.70 (1.43 to 5.11)	Baseline-adjusted	13,962	High	k=1; SWiM
Lee, 2026 (US OPTN/STAR national…)	Primary nonfunction; up to 3 months	Glomerulosclerosis and machine perfusion; Machine-perfusion stratified: GS MPP joint	>20% GS with poor MPP versus 0%-10% GS with good MPP	RR 6.43 (2.64 to 15.70)	Crude; reported	13,962	High	k=1; SWiM
Lee, 2026 (US OPTN/STAR national…)	Primary nonfunction; up to 3 months	Glomerulosclerosis; Machine-perfusion stratified: GS	>20% GS with good MPP versus 0%-10% GS with good MPP	RR 3.46 (1.60 to 7.48)	Crude; reported	13,962	High	k=1; SWiM

Study/cohort	Outcome and horizon	Lesion or score	Contrast or unit	Effect (95% CI)	Adjustment	Analytic n	QUIPS	Cell k and route
Lee, 2026 (US OPTN/STAR national…)	Primary nonfunction; up to 3 months	Interstitial fibrosis/tubular atrophy	more than mild versus none to mild	OR 0.83 (0.29 to 2.37)	Baseline-adjusted	13,962	High	k=1; SWiM
Lubuska, 2006 (1994 to 1997)	Primary nonfunction; week 1	Other acute lesion; Routine: Acute other brush border loss	loss vs preserved	OR 0.82 (0.16 to 4.15)	Crude; reported	NR	High	k=1; SWiM
Lubuska, 2006 (1994 to 1997)	Primary nonfunction; week 1	Other acute lesion; Routine: Acute other hemoglobin cylinders	present vs absent	OR 0.92 (0.16 to 4.75)	Crude; reported	NR	High	k=1; SWiM
Lubuska, 2006 (1994 to 1997)	Primary nonfunction; week 1	Other acute lesion; Routine: Acute other vacuolar dystrophy	any vacuolar dystrophy vs none	OR 0.40 (0.08 to 2.05)	Crude; reported	NR	High	k=1; SWiM
Lubuska, 2006 (1994 to 1997)	Primary nonfunction; week 1	Vascular injury	any arteriolar damage vs none	OR 1.58 (0.31 to 8.84)	Crude; reported	NR	High	k=1; SWiM
Wang, 2022 (distinct cohort)	eGFR; 9-15 months	Acute tubular injury	severe versus mild	BETA -6.43 (-16.80 to 3.94)	Baseline-adjusted	140	High	k=1; SWiM
Amenábar, 2016 (distinct cohort)	eGFR; 9-15 months	Chronic composite score; Ovalle	score 6-8 vs pooled 0-5	MD -8.60 (-14.33 to -2.87)	Crude; recalculated	NR	High	k=1; SWiM
Patel, 2012 (2006-05 to 2010-12)	eGFR; 9-15 months	Chronic composite score; Study-specific: Multi instrument unresolved	GS >=10% or arteriosclerosis >=10%	MD -10.00 (-21.49 to 1.49)	Crude; recalculated	47	High	k=1; SWiM
Lee, 2016 (2006-12 to 2011-07)	eGFR; 9-15 months	Glomerulosclerosis	>0% versus 0%	MD -7.30 (CI not reported)	Crude; reported	135	Moderate	k=1; SWiM
Saha-Chaudhuri, 2020 (distinct cohort)	eGFR; 9-15 months	Glomerulosclerosis	grade 3 versus grade 0	BETA -55.81 (-112.8 to 1.15)	Baseline-adjusted	163	High	k=2; forest; no pooled estimate
Wang, 2022 (distinct cohort)	eGFR; 9-15 months	Glomerulosclerosis	present versus absent	BETA -10.91 (-19.16 to -2.66)	Crude; reported	140	High	k=2; forest; no pooled estimate
Wang, 2022 (distinct cohort)	eGFR; 9-15 months	Interstitial fibrosis/tubular atrophy	present versus absent	BETA -16.95 (-28.41 to -5.49)	Baseline-adjusted	140	High	k=1; SWiM
Saha-Chaudhuri, 2020 (distinct cohort)	eGFR; 9-15 months	Karpinski/MAPI score; Karpinski	7-12 versus 0-3	BETA 29.09 (-5.33 to 63.52)	Baseline-adjusted	163	High	k=1; SWiM
Saha-Chaudhuri, 2020 (distinct cohort)	eGFR; 9-15 months	Remuzzi score	7-12 versus 0-3	BETA -4.39 (-47.37 to 38.58)	Baseline-adjusted	163	High	k=1; SWiM
Villanego, 2022 (2010-01 to 2019-06)	eGFR; 9-15 months	Remuzzi score; Study-specific: Spanish five compartment preimplant score	>3 versus <=3	MD -8.40 (CI not reported)	Crude; reported	266	High	k=1; SWiM
Lee, 2016 (2006-12 to 2011-07)	eGFR; 9-15 months	Tubular atrophy	>=1 versus 0	MD -9.00 (CI not reported)	Crude; reported	135	Moderate	k=1; SWiM
Saha-Chaudhuri, 2020 (distinct cohort)	eGFR; 9-15 months	Tubular atrophy	grade 2 versus grade 0	BETA -19.45 (-46.44 to 7.54)	Baseline-adjusted	163	High	k=2; forest; no pooled estimate
Wang, 2022 (distinct cohort)	eGFR; 9-15 months	Tubular atrophy	present versus absent	BETA -4.62 (-13.70 to 4.45)	Crude; reported	140	High	k=2; forest; no pooled estimate
Lee, 2016 (2006-12 to 2011-07)	eGFR; 9-15 months	Vascular injury	>=1 versus 0	MD -12.80 (CI not reported)	Crude; reported	135	Moderate	k=1; SWiM
Wang, 2022 (distinct cohort)	eGFR; 9-15 months	Vascular injury	present versus absent	BETA -9.68 (-17.53 to -1.84)	Baseline-adjusted	140	High	k=1; SWiM

Abbreviations and symbols: AH, arteriolar hyalinosis; ATN, acute tubular necrosis; BETA, regression coefficient; CADI, Chronic Allograft Damage Index; CI, confidence interval; ci, Banff interstitial fibrosis score; the numeric suffix denotes the grade; CLS, chronic lesion score; ct, Banff tubular atrophy score; the numeric suffix denotes the grade; DDS, deceased donor score; DGF, delayed graft function; eGFR, estimated glomerular filtration rate; GS, glomerulosclerosis; HR, hazard ratio; IFTA, interstitial fibrosis and tubular atrophy; k, number of cohorts in the analytic cell; M-S, moderate to severe; MAPI, Maryland Aggregate Pathology Index; MD, mean difference; MPP, machine perfusion parameters; n, reported sample size; NR, not reported; OPTN, Organ Procurement and Transplantation Network; OR, odds ratio; PGF, periglomerular fibrosis; PNF, primary nonfunction; PROBAST, Prediction model Risk Of Bias ASsessment Tool; QUIPS, Quality In Prognosis Studies; RR, risk ratio; RS, Remuzzi score; STAR, Standard Transplant Analysis and Research; SWiM, synthesis without meta-analysis; UNOS, United Network for Organ Sharing; US, United States; WLR, wall-to-lumen ratio.

Rows are the 211 effects selected across 143 prognostic analytic cells, not the complete 2,063-row extraction universe. Each row is one estimate retained after applying the one-estimate-per-nonoverlapping-cohort-per-cell rule. The cell column gives the number of independent cohorts (k) and the corresponding synthesis route. Forty-eight source effect rows did not state direction explicitly and remain as reported; after synthesis routing, these contributed to 21 single-cohort cells without a securely assignable direction. Two explicitly favorable-versus-adverse contrasts were inverted. Dose-per-10-percentage-point rows were standardized from source coefficients reported per percentage point. Rows marked numerically unstable were retained without repair and did not contribute to a pooled model. The complete machine-readable extraction, identifiers, source locations and row hashes are archived in the data repository.

Table S4. Incremental-prediction model phase, performance, risk of bias and applicability

Study	Model and outcome	Evaluation phase	Tool	RoB	Applicability	Discrimination	Calibration	Clinical utility
Ernst, 2024	DGF: clinical score	Model development	CHARMS + PROBAST+AI	High	High	Not reported for this phase	Not reported for this phase	No decision-curve, net-benefit, prospective-impact, or randomized-utility evaluation
Ernst, 2024	DGF: clinical score	Apparent performance	CHARMS + PROBAST+AI	High	High	AUC 0.672	intercept 0.007; slope 0.989	No decision-curve, net-benefit, prospective-impact, or randomized-utility evaluation
Ernst, 2024	DGF: clinical score	External performance	CHARMS + PROBAST+AI	High	High	AUC 0.702	intercept -0.578; slope 0.628	No decision-curve, net-benefit, prospective-impact, or randomized-utility evaluation
Ernst, 2024	DGF: clinical + histology update	Model development	CHARMS + PROBAST+AI	High	High	Not reported for this phase	Not reported for this phase	No decision-curve, net-benefit, prospective-impact, or randomized-utility evaluation
Ernst, 2024	DGF: clinical + histology update	Apparent performance	CHARMS + PROBAST+AI	High	High	AUC 0.696	approximately intercept 0.007; slope 0.989	No decision-curve, net-benefit, prospective-impact, or randomized-utility evaluation
Ernst, 2024	DGF: clinical + histology update	External performance	CHARMS + PROBAST+AI	High	High	AUC 0.701	intercept -0.584; slope 0.388	No decision-curve, net-benefit, prospective-impact, or randomized-utility evaluation
Ernst, 2024	1-y graft loss: clinical score	Model development	CHARMS + PROBAST+AI	High	High	Not reported for this phase	Not reported for this phase	No decision-curve, net-benefit, prospective-impact, or randomized-utility evaluation
Ernst, 2024	1-y graft loss: clinical score	Apparent performance	CHARMS + PROBAST+AI	High	High	AUC 0.700	intercept -0.025; slope 1.034	No decision-curve, net-benefit, prospective-impact, or randomized-utility evaluation
Ernst, 2024	1-y graft loss: clinical score	External performance	CHARMS + PROBAST+AI	High	High	AUC 0.765	intercept -0.422; slope 1.638	No decision-curve, net-benefit, prospective-impact, or randomized-utility evaluation
Ernst, 2024	1-y graft loss: clinical + histology update	Model development	CHARMS + PROBAST+AI	High	Unclear	Not reported for this phase	Not reported for this phase	No decision-curve, net-benefit, prospective-impact, or randomized-utility evaluation
Ernst, 2024	1-y graft loss: clinical + histology update	Apparent performance	CHARMS + PROBAST+AI	High	Unclear	AUC 0.707	intercept 0.005; slope 1.004	No decision-curve, net-benefit, prospective-impact, or randomized-utility evaluation
Ernst, 2024	1-y graft loss: clinical + histology update	External performance	CHARMS + PROBAST+AI	High	Unclear	AUC 0.701	intercept -0.378; slope 1.454	No decision-curve, net-benefit, prospective-impact, or randomized-utility evaluation
Zagni, 2022	Poor early graft function: brush-border score	Model development	CHARMS + PROBAST+AI	High	High	Not reported for this phase	Not reported for this phase	No decision-curve, net-benefit, prospective-impact, or randomized-utility evaluation
Zagni, 2022	Poor early graft function: brush-border score	Apparent performance	CHARMS + PROBAST+AI	High	High	AUC 0.68 (95% CI 0.51-0.82)	not reported	No decision-curve, net-benefit, prospective-impact, or randomized-utility evaluation
Traynor, 2017	5-y eGFR <=35: clinical + Karpinski	Development/apparent performance	CHARMS + PROBAST+AI	High	High	Clinical model AUC/C-statistic 0.64; plus Karpinski 0.67 (body; abstract reports 0.66), p=0.65	Not reported	No decision-curve, net-benefit, prospective-impact, or randomized-utility evaluation
Hall, 2018	Leuven score: discard/DGF/graft failure/eGFR	Model evaluation	CHARMS + PROBAST+AI	High	High	AUC values reported for discard, three-year allograft failure, and DGF; R^2 reported for eGFR	Not reported	No decision-curve, net-benefit, prospective-impact, or randomized-utility evaluation

Abbreviations and symbols: AUC, area under the receiver operating characteristic curve; CHARMS, Critical Appraisal and Data Extraction for Systematic Reviews of Prediction Modelling Studies; CI, confidence interval; DGF, delayed graft function; eGFR, estimated glomerular filtration rate; PROBAST+AI, Prediction model Risk Of Bias ASsessment Tool plus Artificial Intelligence; R^2, coefficient of determination; RoB, risk of bias; y, years.

Rows are model phases, not independent studies. Blank performance fields in the source appraisal are displayed as “Not reported for this phase.” AUC, area under the receiver-operating-characteristic curve; DGF, delayed graft function; eGFR, estimated glomerular filtration rate; PROBAST+AI, Prediction model Risk Of Bias ASsessment Tool plus artificial intelligence extension; RoB, risk of bias.

Table S5. Clinical-utility risk-of-bias assessments, effects and outcome-level certainty

Panel A. Design-specific risk-of-bias assessments underlying Figure 2

Study/comparison	Outcome	Instrument	Overall judgment
PITHIA trial	Biopsy-related adverse events	RoB 2 cluster	Not applicable
PITHIA trial	Cold ischemia time	RoB 2 cluster	Some concerns
PITHIA trial	DGF (dialysis within 7 d)	RoB 2 cluster	Some concerns
PITHIA trial	eGFR at 12 months	RoB 2 cluster	Some concerns
PITHIA trial	Graft survival at 12 months	RoB 2 cluster	Some concerns
PITHIA trial	Patient survival at 12 months	RoB 2 cluster	Some concerns
PITHIA trial	Primary nonfunction	RoB 2 cluster	Some concerns
PITHIA trial	Transplantation at first offer	RoB 2 cluster	Low
PITHIA trial	Overall transplantation at last offer	RoB 2 cluster	Some concerns
Mallon et al. 2015	DGF (dialysis within 7 d)	ROBINS-I	Critical
Gandolfini et al. 2014	Death-censored graft failure	ROBINS-I	Serious
Sung et al. 2008	Kidney discard	ROBINS-I	Serious
Woodside et al. 2012	Kidney discard	ROBINS-I	Serious
Comai et al. 2026	Graft failure; source definition unclear	ROBINS-I	Critical
Remuzzi et al. 2006	Graft failure	ROBINS-I	Critical
Odaldi et al. 2022	Graft loss	ROBINS-I	Serious
Bissolati et al. 2021	DGF (dialysis within 7 d)	ROBINS-I	Critical
Bissolati et al. 2021	Renal function at postoperative day 30	ROBINS-I	Critical

Abbreviations and symbols: d, days; DGF, delayed graft function; eGFR, estimated glomerular filtration rate; PITHIA, PreImplantation Trial of Histopathology In renal Allografts; RoB 2, revised Cochrane risk-of-bias tool for randomized trials; ROBINS-I, Risk Of Bias In Non-randomized Studies of Interventions.

Panel A contains the 18 design-applicable clinical-utility risk-of-bias units counted in Figure 2: nine outcome-specific RoB 2 cluster assessments from PITHIA and nine ROBINS-I strategy–outcome assessments. Provisional labels attached to two abstract-only source assessments in the appraisal ledger are displayed here using their final normalized categories.

Panel B. Extractable clinical-utility effects and outcome-level certainty

Study	Comparison	Design	Outcome	Effect	CI or precision	Reason not pooled	Final certainty
PITHIA trial	Access to national digital preimplantation biopsy service versus no access	Stepped wedge cluster randomized registry trial	Biopsy-related adverse events	No biopsy-related complications were reported among 287 biopsied kidneys; no randomized-arm comparative effect.	Not estimable	Single cluster-randomized policy evaluation; no compatible second study	Not estimable
PITHIA trial	Access to national digital preimplantation biopsy service versus no access	Stepped wedge cluster randomized registry trial	Cold ischemia time	Adjusted MD 0.73 h	95% CI -0.08 to 1.53	Single cluster-randomized policy evaluation; no compatible second study	Low
PITHIA trial	Access to national digital preimplantation biopsy service versus no access	Stepped wedge cluster randomized registry trial	DGF (dialysis within 7 d)	Adjusted OR 1.12; RD -0.01	95% CI 0.67 to 1.86; 95% CI -0.10 to 0.08	Single cluster-randomized policy evaluation; no compatible second study	Very low
PITHIA trial	Access to national digital preimplantation biopsy service versus no access	Stepped wedge cluster randomized registry trial	eGFR at 12 months	Adjusted MD 1.53 mL/min/1.73 m^2	97.5% CI -2.33 to 5.40	Single cluster-randomized policy evaluation; no compatible second study	Low
PITHIA trial	Access to national digital preimplantation biopsy service versus no access	Stepped wedge cluster randomized registry trial	Graft survival at 12 months	Adjusted HR 1.02	95% CI 0.56 to 1.85	Single cluster-randomized policy evaluation; no compatible second study	Very low
PITHIA trial	Access to national digital preimplantation biopsy service versus no access	Stepped wedge cluster randomized registry trial	Patient survival at 12 months	Adjusted HR 0.67	95% CI 0.35 to 1.28	Single cluster-randomized policy evaluation; no compatible second study	Very low
PITHIA trial	Access to national digital preimplantation biopsy service versus no access	Stepped wedge cluster randomized registry trial	Primary nonfunction	Adjusted OR 1.60; RD 0.02	95% CI 0.51 to 5.02; 95% CI -0.04 to 0.08	Single cluster-randomized policy evaluation; no compatible second study	Very low
PITHIA trial	Access to national digital preimplantation biopsy service versus no access	Stepped wedge cluster randomized registry trial	Transplantation at first offer	Adjusted OR 0.91; RD -0.01	97.5% CI 0.60 to 1.39; 97.5% CI -0.08 to 0.06	Single cluster-randomized policy evaluation; no compatible second study	Moderate
PITHIA trial	Access to national digital preimplantation biopsy service versus no access	Stepped wedge cluster randomized registry trial	Overall transplantation at last offer	Adjusted OR 0.99	95% CI 0.70 to 1.42	Single cluster-randomized policy evaluation; no compatible second study	Low

Study	Comparison	Design	Outcome	Effect	CI or precision	Reason not pooled	Final certainty
Remuzzi et al. 2006	histology-guided allocation of older-donor kidneys versus matched older-donor negative-reference recipients	prospective nonrandomized comparative cohort	graft failure/return to dialysis	HR 3.68, negative reference relative to histology-guided recipients	95% CI 1.29 to 10.52	Comparative allocation strategy is nonrandomized and does not estimate an isolated prognostic lesion effect.	Very low
Italian biopsy-timing cohort 2022	in-situ pre-cross-clamp versus ex-situ back-table biopsy	retrospective cohort with propensity-score adjustment	graft loss	adjusted OR 0.37	95% CI 0.14 to 0.94	Process/timing intervention is clinically distinct from lesion-based prognostic effects.	Very low
Italian ECD single-versus-dual cohort 2016	biopsy-informed single versus dual kidney transplantation	retrospective single-center cohort	DGF, graft function, graft and patient survival	descriptive comparative results; no compatible adjusted ratio	not variance-ready for a biopsy-use effect	Biopsy informed both treatment arms and assignment was nonrandomized.	Not estimable
Bissolati et al. 2021	biopsy-informed Karpinski/MAPI allocation strata	nonrandomized decision-utility cohort	DGF and POD30 renal function	Contextual results only; no isolated variance-ready strategy effect	Not estimable	Allocation contrast does not estimate an isolated prognostic lesion effect; ROBINS-I overall critical.	Not estimable
D’Arcy et al. 2009	biopsy-informed dual versus single transplantation	single-center comparative cohort; abstract/full-report family	DGF/ATN, graft function and 36-month survival	descriptive; no variance-ready strategy effect in the abstract record	not reported	Abstract and full report are one study; nonrandomized allocation does not isolate biopsy utility.	Not estimable
Frutos et al. 2012	biopsy-informed dual kidney transplantation vs biopsy-informed single kidney transplantation	single-center nonrandomized case-control	creatinine clearance at 6 months	MD 14.6 mL/min	Wald 95% CI 5.13 to 24.07 (recalculated from reported SDs)	Both groups used a multivariable biopsy-informed allocation protocol; contrast does not isolate the incremental effect of biopsy information and is confounded by indication.	Not estimable
Frutos et al. 2012	biopsy-informed dual kidney transplantation vs biopsy-informed single kidney transplantation	single-center nonrandomized case-control	delayed graft function	RR 0.86	descriptive; 6/20 vs 14/40	Confounding by indication; no comparison of biopsy use versus non-use.	Not estimable
Frutos et al. 2012	biopsy-informed dual kidney transplantation vs biopsy-informed single kidney transplantation	single-center nonrandomized case-control	dialysis-free graft survival at 3 years	90% vs 90%	log-rank P not significant; no HR	No variance-ready comparative time-to-event estimate.	Not estimable

Abbreviations and symbols: ATN, acute tubular necrosis; CI, confidence interval; d, days; DGF, delayed graft function; ECD, expanded-criteria donor; eGFR, estimated glomerular filtration rate; h, hours; HR, hazard ratio; m^2, square meters; MAPI, Maryland Aggregate Pathology Index; MD, mean difference; min, minutes; mL, milliliters; OR, odds ratio; PITHIA, PreImplantation Trial of Histopathology In renal Allografts; POD30, postoperative day 30; RD, risk difference; RoB 2, revised Cochrane risk-of-bias tool for randomized trials; ROBINS-I, Risk Of Bias In Non-randomized Studies of Interventions; RR, risk ratio; SD, standard deviation.

Panel B contains 17 result/certainty rows selected for reader-facing clinical-utility synthesis. Panels A and B are not one-to-one ledgers: a design-specific appraisal can support more than one reported result, whereas some contextual result rows did not admit an exact outcome-level design-specific risk-of-bias judgment. The nine PITHIA rows come from one cluster-randomized policy evaluation and are not nine independent studies. CI, confidence interval; DGF, delayed graft function; eGFR, estimated glomerular filtration rate; HR, hazard ratio; MD, mean difference; OR, odds ratio; RD, risk difference; RR, risk ratio. “Not estimable” is not a certainty rating.

Table S6A. Measurement evidence

Study	Measurement target	Result	Clinical context	Synthesis route
Ayorinde, 2024	measurement uncertainty in renal preimplantation biopsy assessment	metrological uncertainty and robustness evidence	No eligible clinical prognostic ratio.	Measurement narrative
Yi, 2022	deep-learning compartment segmentation on frozen procurement biopsies	tissue-compartment classification and slide-wide correlation metrics	Abstract companion of historical included PMID 37923131; no independent study/effect count.	Family merge; measurement narrative
Moledina, 2017	procurement-biopsy acute tubular injury	AUROC severe ATI: delta creatinine 0.58 (0.49-0.67); NGAL 0.67 (0.60-0.74)	Adjusted 6-month eGFR 43.2 severe, 46.1 mild, 49.4 no ATI; contrast variance not reported.	Measurement narrative + prognostic SWiM
Koo, 1998	paired pre/post-reperfusion immunohistochemistry	Post-reperfusion neutrophils 29/55 and P-selectin 24/55 in deceased-donor allografts	Post-reperfusion change is contextual and outside strict pre-reperfusion prognostic pooling.	Measurement SWiM
Dong, 2024	CEUS against procurement-biopsy Remuzzi/pathology	Quantitative measurement/correlation and AUROC evidence	No eligible baseline-adjusted histology-outcome ratio.	Measurement narrative
Salvi, 2020	automated vascular and fibrosis segmentation	Accuracy, precision, recall, F1, Dice and distance metrics	Computational morphology; no clinical prognostic ratio.	Measurement narrative
Marsh, 2021	automated glomerulosclerosis quantification	algorithm development and validation against pathologist assessment	No recipient outcome effect was estimated.	Measurement narrative
Pan, 2024	sound-touch elastography against procurement histology/allograft function	measurement/prediction metrics	Authorized route is Domain M; not entered into morphology prognostic pools.	Measurement narrative
Loizeau, 2024	repeat-biopsy processing and assessment uncertainty	measurement uncertainty decomposition	No eligible post-transplant prognostic ratio.	Measurement narrative
Hysi, 2025	quantitative ultrasound assessment of transplant-kidney fibrosis	technology validation against fibrosis	No eligible preimplantation lesion-to-outcome effect.	Measurement narrative

Abbreviations and symbols: ATI, acute tubular injury; AUROC, area under the receiver operating characteristic curve; CEUS, contrast-enhanced ultrasonography; eGFR, estimated glomerular filtration rate; F1, harmonic mean of precision and recall; M, measurement review question; NGAL, neutrophil gelatinase-associated lipocalin; PMID, PubMed identifier; SWiM, synthesis without meta-analysis.

AUROC, area under the receiver-operating-characteristic curve; ATI, acute tubular injury; CEUS, contrast-enhanced ultrasonography; eGFR, estimated glomerular filtration rate; NGAL, neutrophil gelatinase-associated lipocalin. Technical measurement performance was not treated as clinical prediction or decision benefit.

Table S6B. Measurement applicability and quality

Study	Tool	Quality judgment	Applicability	Final certainty
Li, 2021	QAREL	Moderate	Direct: time-zero frozen-section measurement	Not applicable
Limas, 1977	QAREL	Not applicable primary change study	Indirect: mixed timing; contextual only	Not applicable
Huang, 2018	QAREL	Moderate	Partial: pretransplant paraffin-method comparison	Not applicable
Mittal, 2017	QAREL	Low	Partial: procurement context in discarded organs	Not applicable
Keijbeck, 2020	QAREL-adapted method comparison	Low quality	Partial: nonbiopsy macroscopic comparator	Not applicable
Gaber, 1995	QAREL not applicable	Not applicable	Postreperfusion, mixed-donor context only	Not applicable

Abbreviations and symbols: GRADE, Grading of Recommendations Assessment, Development and Evaluation; QAREL, Quality Appraisal for Reliability Studies.

QAREL, Quality Appraisal for Reliability Studies. “Not applicable” records that the measurement evidence was not forced into an intervention or prognostic GRADE rating.

Table S7. Effect selection, dependency decisions and final analysis routing

The first panel reconciles every selection denominator used in the main article. The second panel prints all 21 cohort-dependency decisions that could affect the independent-cohort count. The complete 211-row selected-effect table is Table S3; it is not duplicated here.

Selection stage or final route	n	Meaning
Extracted effect universe	2063	Complete source-level extraction; not printed as a 149-field table
Candidates entering deterministic selection	551	One candidate ledger; alternatives and exclusions retained internally
Quantitatively eligible candidates	440	Effect and variance suitable for quantitative routing
Selected independent effects	211	One estimate per nonoverlapping cohort and analytic cell
Analytic cells	143	Clinically compatible outcome–lesion–measure families
Principal pools	7	At least five independent cohorts
Exploratory pools	5	Three or four independent cohorts
Two-cohort displays	15	No pooled summary; all 30 effects shown in Supplementary Figure S1
Single-cohort SWiM cells	116	All 116 cells shown in Main Figure 6 and fully identified in Table S3

Abbreviations and symbols: n, reported sample size; SWiM, synthesis without meta-analysis.

Table S7 (continued). Cohort-dependency decisions

Demonstrated or unresolved overlap could not increase k within a shared analytic cell; distinct periods or mutually exclusive strata could contribute separately when supported.

Cohort A	Cohort B	Relation	Decision	Reason	Blocked candidates
Husain, 2022 (national OPTN cohort)	Husain, 2022 (creatinine 1–2 mg/dL stratum)	Nested subcohort	Block joint k if shared cell	Table-3 stratum is contained in the same national cohort.	1
Husain, 2022 (national OPTN cohort)	Husain, 2022 (creatinine >2 mg/dL stratum)	Nested subcohort	Block joint k if shared cell	Table-3 stratum is contained in the same national cohort.	1
Husain, 2022 (national OPTN cohort)	Husain, 2022 (creatinine <1 mg/dL stratum)	Nested subcohort	Block joint k if shared cell	Table-3 stratum is contained in the same national cohort.	1
Husain, 2022 (national OPTN cohort)	Husain, 2022 (KDPI 36–84 stratum)	Nested subcohort	Block joint k if shared cell	Table-3 stratum is contained in the same national cohort.	1
Husain, 2022 (national OPTN cohort)	Husain, 2022 (KDPI >=85 stratum)	Nested subcohort	Block joint k if shared cell	Table-3 stratum is contained in the same national cohort.	1
Husain, 2022 (national OPTN cohort)	Husain, 2022 (KDPI <=35 stratum)	Nested subcohort	Block joint k if shared cell	Table-3 stratum is contained in the same national cohort.	1
Husain, 2022 (national OPTN cohort)	Lee, 2026 (national OPTN/STAR cohort)	Possible partial overlap	Block joint k if shared cell	Both are national OPTN/STAR biopsy cohorts; the 2015–2019 window lies within the 2014–2022 window.	0
Husain, 2022 (national OPTN cohort)	Husain, 2021 (national STAR cohort)	Possible partial overlap	Block joint k if shared cell	The 2015–2019 national OPTN cohort may contain participants from the 2015–2020 national STAR paired-kidney cohort.	0
Azancot, 2014 (central reread)	Azancot, 2014 (on-call reading)	Nested subcohort	Block joint k if shared cell	Central reread was available for 112 of 117 ECD transplants.	2
Stewart, 2022	Husain, 2021 (national STAR cohort)	Nonoverlapping periods	Allow separate	Registry source is related but calendar windows do not overlap.	0
Stewart, 2022	Cheungpasitporn, 2020	Possible partial overlap	Block joint k if shared cell	Stewart, 2022 uses national OPTN ECD transplants from 2008-2012, within the Cheungpasitporn, 2020 OPTN 2005-2014 window.	1
Wang, 2022	Tan, 2024 (full report cohort)	Possible partial overlap	Block joint k if shared cell	Same hospital and overlapping 2014-Jun 2017 window, but different eligibility counts and no participant identifiers.	0
Lee, 2026 (national OPTN/STAR cohort)	Husain, 2021 (Columbia local cohort)	Possible partial overlap	Block joint k if shared cell	The Columbia local cohort overlaps 2014–2016 and may contribute participants to the national 2014–2022 OPTN/STAR cohort.	0
Lee, 2026 (national OPTN/STAR cohort)	Husain, 2021 (national STAR cohort)	Possible partial overlap	Block joint k if shared cell	The national STAR paired-kidney cohort from 2015–2020 overlaps the national OPTN/STAR 2014–2022 cohort.	0
Lee, 2026 (national OPTN/STAR cohort)	Cheungpasitporn, 2020	Disjoint eligibility strata	Allow separate	The KDPI eligibility strata are mutually exclusive: 35–85 versus >85, despite the shared boundary year.	0

Cohort A	Cohort B	Relation	Decision	Reason	Blocked candidates
Husain, 2021 (Columbia local cohort)	Husain, 2021 (national STAR cohort)	Possible partial overlap	Block joint k if shared cell	Columbia recipients in 2015-2016 may occur in STAR, but final paired-sample identity is unavailable.	1
Pavlovic, 2022	Hofer, 2014	Distinct cohorts	Allow separate	The reports share a center, but their transplant windows do not overlap.	0
Mourão, 2016 (PBMC analysis)	Mourão, 2016 (preimplantation-biopsy analysis)	Possible partial overlap	Block joint k if shared cell	The two analytic samples are reported in distinct tables, but participant-level overlap is not reported.	1
Kayler, 2008	Kayler, 2008 (ECD subgroup)	Nested subcohort	Block joint k if shared cell	The Kayler, 2008 source labels the n=167 ECD analysis as a subgroup of the full n=597 UPMC deceased-donor cohort.	2
Zheng, 2020	Li, 2024	Partial overlap subset; possible partial overlap	Block joint k if shared cell	The same Xi'an center and 17-month window overlap are documented, but the windows, recipient counts and donor selection differ and participant identifiers are unavailable.	0
Tan, 2024 (full report cohort)	Tan, 2024 (adequate-biopsy subgroup)	Nested subcohort	Block joint k if shared cell	Adequate-biopsy n=97 is nested in the n=185 report cohort.	0

Abbreviations and symbols: ECD, expanded-criteria donor; k, number of cohorts in the analytic cell; KDPI, Kidney Donor Profile Index; n, reported sample size; OPTN, Organ Procurement and Transplantation Network; PBMC, peripheral blood mononuclear cells; STAR, Standard Transplant Analysis and Research; UPMC, University of Pittsburgh Medical Center.

Reader-facing labels use author–year plus a stratum qualifier where needed; internal cohort identifiers remain in the deposited provenance. Each row is an adjudicated dependency edge, not an additional effect.

Table S8. All REML–Hartung–Knapp pooled results

All 12 pooled prognostic bodies meeting the prespecified cohort-count thresholds are printed below. Principal bodies had at least five independent cohorts; exploratory bodies had three or four. Prediction intervals were calculated only for principal bodies.

Role	Outcome and lesion	k	Effect (95% CI)	Prediction interval	I²	P
Principal	Graft failure x glomerulosclerosis	9	HR 2.03 (1.22–3.39)	0.52–7.91	86.0%	0.012
Principal	Graft failure x vascular injury	9	HR 1.48 (1.07–2.04)	0.80–2.71	53.0%	0.023
Principal	Delayed graft function x glomerulosclerosis	8	OR 1.13 (0.59–2.17)	0.32–3.99	74.0%	0.670
Principal	Delayed graft function x vascular injury	7	OR 1.85 (1.11–3.06)	0.53–6.41	68.4%	0.025
Principal	Delayed graft function x IFTA	6	OR 1.78 (0.80–3.98)	0.31–10.41	71.6%	0.124
Principal	Graft failure x IFTA	6	HR 1.64 (0.96–2.82)	0.64–4.20	50.9%	0.064
Principal	Delayed graft function x acute tubular injury	5	OR 5.04 (0.82–30.86)	0.09–292.15	86.6%	0.068
Exploratory	Delayed graft function x Remuzzi score	3	OR 1.09 (0.63–1.89)	Not eligible (k<5)	12.8%	0.554
Exploratory	Delayed graft function x vascular injury (per grade)	3	OR 1.98 (0.76–5.17)	Not eligible (k<5)	0.0%	0.092
Exploratory	Low eGFR at 9–15 months x IFTA	3	OR 1.33 (0.12–15.20)	Not eligible (k<5)	86.5%	0.662
Exploratory	Low eGFR at 9–15 months x vascular injury	3	OR 1.49 (0.31–7.18)	Not eligible (k<5)	63.9%	0.387
Exploratory	Graft failure x tubular atrophy	3	HR 1.27 (0.07–21.69)	Not eligible (k<5)	83.2%	0.752

Abbreviations and symbols: CI, confidence interval; eGFR, estimated glomerular filtration rate; HR, hazard ratio; I^2, percentage of total variability attributable to between-study heterogeneity; IFTA, interstitial fibrosis and tubular atrophy; k, number of cohorts in the analytic cell; OR, odds ratio; REML, restricted maximum likelihood.

REML, restricted maximum likelihood. P values are two-sided tests of the pooled association, not tests of heterogeneity. Every model was independently recomputed; the largest absolute discrepancy on the analysis scale was 0.0000000000000426.

Table S9. Illustrative absolute effects for delayed graft function

All 15 prespecified scenarios are shown: five DGF odds-ratio bodies at hypothetical baseline risks of 20%, 30% and 40%. These baselines are locked scenarios, not pooled observed control risks.

Outcome and lesion	k	Pooled OR (95% CI)	Baseline risk	Exposed risk (95% interval)	Difference per 1000 (95% interval)	MID	Point reaches MID
Delayed graft function x IFTA	6	OR 1.78 (0.80–3.98)	20%	30.8% (16.6%–49.9%)	+108 (-34 to +299)	50 per 1000	Yes
Delayed graft function x IFTA	6	OR 1.78 (0.80–3.98)	30%	43.3% (25.5%–63.0%)	+133 (-45 to +330)	50 per 1000	Yes
Delayed graft function x IFTA	6	OR 1.78 (0.80–3.98)	40%	54.3% (34.7%–72.6%)	+143 (-53 to +326)	50 per 1000	Yes
Delayed graft function x Remuzzi score	3	OR 1.09 (0.63–1.89)	20%	21.5% (13.7%–32.1%)	+15 (-63 to +121)	50 per 1000	No
Delayed graft function x Remuzzi score	3	OR 1.09 (0.63–1.89)	30%	31.9% (21.3%–44.7%)	+19 (-87 to +147)	50 per 1000	No
Delayed graft function x Remuzzi score	3	OR 1.09 (0.63–1.89)	40%	42.2% (29.7%–55.7%)	+22 (-103 to +157)	50 per 1000	No
Delayed graft function x acute tubular injury	5	OR 5.04 (0.82–30.86)	20%	55.8% (17.1%–88.5%)	+358 (-29 to +685)	50 per 1000	Yes
Delayed graft function x acute tubular injury	5	OR 5.04 (0.82–30.86)	30%	68.4% (26.1%–93.0%)	+384 (-39 to +630)	50 per 1000	Yes
Delayed graft function x acute tubular injury	5	OR 5.04 (0.82–30.86)	40%	77.1% (35.4%–95.4%)	+371 (-46 to +554)	50 per 1000	Yes
Delayed graft function x glomerulosclerosis	8	OR 1.13 (0.59–2.17)	20%	22.0% (12.8%–35.1%)	+20 (-72 to +151)	50 per 1000	No
Delayed graft function x glomerulosclerosis	8	OR 1.13 (0.59–2.17)	30%	32.6% (20.2%–48.1%)	+26 (-98 to +181)	50 per 1000	No
Delayed graft function x glomerulosclerosis	8	OR 1.13 (0.59–2.17)	40%	43.0% (28.2%–59.1%)	+30 (-118 to +191)	50 per 1000	No
Delayed graft function x vascular injury	7	OR 1.85 (1.11–3.06)	20%	31.6% (21.8%–43.3%)	+116 (+18 to +233)	50 per 1000	Yes
Delayed graft function x vascular injury	7	OR 1.85 (1.11–3.06)	30%	44.2% (32.3%–56.7%)	+142 (+23 to +267)	50 per 1000	Yes
Delayed graft function x vascular injury	7	OR 1.85 (1.11–3.06)	40%	55.2% (42.6%–67.1%)	+152 (+26 to +271)	50 per 1000	Yes

Abbreviations and symbols: CI, confidence interval; DGF, delayed graft function; IFTA, interstitial fibrosis and tubular atrophy; k, number of cohorts in the analytic cell; MID, minimal important difference; OR, odds ratio.

MID, minimal important difference; 50 events per 1000 was a prespecified author-judgment threshold. Remuzzi score is represented by a dichotomous contrast with a defined reference risk; vascular injury (per grade) is a one-unit dose contrast without a common exposed-versus-unexposed binary risk translation. A point estimate reaching the MID does not imply that the confidence interval excludes smaller effects or the null.

Table S10. Why graft-failure absolute effects were not estimable

All four graft-failure hazard-ratio bodies were assessed for absolute translation. None had both a compatible common horizon and an eligible baseline survival function.

Outcome and lesion	k	Pooled HR (95% CI)	Requested baseline risks	MID	Reason not estimable
Graft failure x glomerulosclerosis	9	HR 2.03 (1.22–3.39)	5%, 10%, 20%	30 per 1000	Contributing HRs use heterogeneous or longest-eligible horizons; no eligible common 5-year baseline survival was supplied. A hazard ratio is not treated as a risk ratio.
Graft failure x IFTA	6	HR 1.64 (0.96–2.82)	5%, 10%, 20%	30 per 1000	Contributing HRs use heterogeneous or longest-eligible horizons; no eligible common 5-year baseline survival was supplied. A hazard ratio is not treated as a risk ratio.
Graft failure x tubular atrophy	3	HR 1.27 (0.07–21.69)	5%, 10%, 20%	30 per 1000	Contributing HRs use heterogeneous or longest-eligible horizons; no eligible common 5-year baseline survival was supplied. A hazard ratio is not treated as a risk ratio.
Graft failure x vascular injury	9	HR 1.48 (1.07–2.04)	5%, 10%, 20%	30 per 1000	Contributing HRs use heterogeneous or longest-eligible horizons; no eligible common 5-year baseline survival was supplied. A hazard ratio is not treated as a risk ratio.

Abbreviations and symbols: CI, confidence interval; HR, hazard ratio; IFTA, interstitial fibrosis and tubular atrophy; k, number of cohorts in the analytic cell; MID, minimal important difference.

The requested baseline risks and 30-per-1000 MID were prespecified author-judgment scenarios. A hazard ratio was not treated as a risk ratio, and no absolute graft-failure estimate was imputed.

Table S11. Prespecified sensitivity-analysis completion

Every one of the 216 prespecified routes is accounted for below by parent body. Numerical results for all 56 executed routes follow in the continuation panel; routes with fewer than three cohorts were not pooled.

Parent outcome and lesion	Prespecified	Executed	k<3; not pooled	Outcome-inapplicable
Delayed graft function x IFTA	18	4	14	0
Delayed graft function x Remuzzi score	18	2	16	0
Delayed graft function x acute tubular injury	18	3	15	0
Delayed graft function x glomerulosclerosis	18	7	11	0
Delayed graft function x vascular injury	18	9	9	0
Delayed graft function x vascular injury (per grade)	18	1	17	0
Graft failure x IFTA	18	7	10	1
Graft failure x glomerulosclerosis	18	8	9	1
Graft failure x tubular atrophy	18	4	13	1
Graft failure x vascular injury	18	9	8	1
Low eGFR at 9–15 months x IFTA	18	1	16	1
Low eGFR at 9–15 months x vascular injury	18	1	16	1

Abbreviations and symbols: eGFR, estimated glomerular filtration rate; IFTA, interstitial fibrosis and tubular atrophy; k, number of cohorts in the analytic cell.

Table S11 (continued). All executed sensitivity models

The table prints every executed sensitivity model, including routes with zero estimated heterogeneity; no route was selected post hoc as a preferred result.

Parent outcome and lesion	Prespecified restriction	k	Effect (95% CI)	I^2	P	Threshold consequence
Delayed graft function x IFTA	Exclude national registries	5	OR 2.14 (1.05–4.35)	0.0%	0.041	Retains primary threshold
Delayed graft function x IFTA	Exclude uncorrected or unknown clustering	6	OR 1.78 (0.80–3.98)	71.6%	0.124	Retains primary threshold
Delayed graft function x IFTA	Exclude uncorrected paired-kidney effects	6	OR 1.78 (0.80–3.98)	71.6%	0.124	Retains primary threshold
Delayed graft function x IFTA	Strict dialysis-within-7-days DGF	5	OR 1.81 (0.61–5.34)	75.6%	0.203	Retains primary threshold
Delayed graft function x Remuzzi score	Exclude national registries	3	OR 1.09 (0.63–1.89)	12.8%	0.554	Below primary retains exploratory pool
Delayed graft function x Remuzzi score	Exclude uncorrected paired-kidney effects	3	OR 1.09 (0.63–1.89)	12.8%	0.554	Below primary retains exploratory pool
Delayed graft function x acute tubular injury	Exclude national registries	5	OR 5.04 (0.82–30.86)	86.6%	0.068	Retains primary threshold
Delayed graft function x acute tubular injury	Exclude uncorrected paired-kidney effects	5	OR 5.04 (0.82–30.86)	86.6%	0.068	Retains primary threshold
Delayed graft function x acute tubular injury	Strict dialysis-within-7-days DGF	5	OR 5.04 (0.82–30.86)	86.6%	0.068	Retains primary threshold
Delayed graft function x glomerulosclerosis	Analytic n >=200	3	OR 1.55 (0.08–30.97)	97.8%	0.595	Below primary retains exploratory pool
Delayed graft function x glomerulosclerosis	Baseline-adjusted only	3	OR 0.87 (0.67–1.13)	0.0%	0.148	Below primary retains exploratory pool
Delayed graft function x glomerulosclerosis	Exclude national registries	6	OR 1.42 (0.45–4.54)	57.6%	0.470	Retains primary threshold
Delayed graft function x glomerulosclerosis	Exclude uncorrected or unknown clustering	7	OR 1.25 (0.51–3.05)	65.1%	0.560	Retains primary threshold
Delayed graft function x glomerulosclerosis	Exclude uncorrected paired-kidney effects	8	OR 1.13 (0.59–2.17)	74.0%	0.670	Retains primary threshold
Delayed graft function x glomerulosclerosis	Pre-Banff-2017 era only	4	OR 0.83 (0.55–1.24)	0.0%	0.233	Below primary retains exploratory pool
Delayed graft function x glomerulosclerosis	Strict dialysis-within-7-days DGF	5	OR 1.65 (0.63–4.34)	66.5%	0.225	Retains primary threshold
Delayed graft function x vascular injury	Baseline-adjusted only	4	OR 1.55 (0.71–3.37)	60.9%	0.169	Below primary retains exploratory pool
Delayed graft function x vascular injury	Core biopsy only	3	OR 2.79 (0.71–10.98)	51.2%	0.085	Below primary retains exploratory pool
Delayed graft function x vascular injury	Exclude national registries	6	OR 2.25 (1.32–3.80)	36.5%	0.011	Retains primary threshold
Delayed graft function x vascular injury	Exclude uncorrected or unknown clustering	7	OR 1.85 (1.11–3.06)	68.4%	0.025	Retains primary threshold
Delayed graft function x vascular injury	Exclude uncorrected paired-kidney effects	7	OR 1.85 (1.11–3.06)	68.4%	0.025	Retains primary threshold
Delayed graft function x vascular injury	Expert or central reading only	3	OR 1.80 (0.58–5.64)	0.0%	0.156	Below primary retains exploratory pool
Delayed graft function x vascular injury	Paraffin processing only	3	OR 3.12 (1.23–7.91)	18.7%	0.034	Below primary retains exploratory pool
Delayed graft function x vascular injury	Pre-Banff-2017 era only	3	OR 2.18 (1.12–4.24)	0.0%	0.037	Below primary retains exploratory pool
Delayed graft function x vascular injury	Strict dialysis-within-7-days DGF	5	OR 2.37 (1.24–4.54)	40.8%	0.021	Retains primary threshold

Parent outcome and lesion	Prespecified restriction	k	Effect (95% CI)	I^2	P	Threshold consequence
Delayed graft function x vascular injury (per grade)	Exclude national registries	3	OR 1.98 (0.76–5.17)	0.0%	0.092	Below primary retains exploratory pool
Graft failure x IFTA	Analytic n >=200	5	HR 1.77 (1.03–3.04)	49.9%	0.043	Retains primary threshold
Graft failure x IFTA	Exclude national registries	5	HR 1.76 (0.80–3.88)	59.9%	0.119	Retains primary threshold
Graft failure x IFTA	Exclude uncorrected or unknown clustering	4	HR 1.83 (0.53–6.37)	67.1%	0.221	Below primary retains exploratory pool
Graft failure x IFTA	Exclude uncorrected paired-kidney effects	6	HR 1.64 (0.96–2.82)	50.9%	0.064	Retains primary threshold
Graft failure x IFTA	Expert or central reading only	4	HR 1.52 (0.82–2.82)	0.0%	0.121	Below primary retains exploratory pool
Graft failure x IFTA	Pre-Banff-2017 era only	3	HR 2.02 (0.20–20.80)	75.7%	0.324	Below primary retains exploratory pool
Graft failure x IFTA	Wedge biopsy only	4	HR 1.83 (0.53–6.37)	67.1%	0.221	Below primary retains exploratory pool
Graft failure x glomerulosclerosis	Analytic n >=200	6	HR 2.09 (0.94–4.66)	93.1%	0.065	Retains primary threshold
Graft failure x glomerulosclerosis	Baseline-adjusted only	4	HR 1.48 (0.69–3.17)	82.4%	0.198	Below primary retains exploratory pool
Graft failure x glomerulosclerosis	Exclude national registries	7	HR 2.55 (1.42–4.60)	64.0%	0.008	Retains primary threshold
Graft failure x glomerulosclerosis	Exclude uncorrected or unknown clustering	7	HR 2.29 (1.10–4.76)	82.6%	0.032	Retains primary threshold
Graft failure x glomerulosclerosis	Exclude uncorrected paired-kidney effects	9	HR 2.03 (1.22–3.39)	86.0%	0.012	Retains primary threshold
Graft failure x glomerulosclerosis	Expert or central reading only	5	HR 1.90 (1.57–2.30)	0.0%	0.001	Retains primary threshold
Graft failure x glomerulosclerosis	Pre-Banff-2017 era only	5	HR 2.27 (0.73–7.10)	89.0%	0.117	Retains primary threshold
Graft failure x glomerulosclerosis	Wedge biopsy only	5	HR 2.94 (1.18–7.34)	65.8%	0.031	Retains primary threshold
Graft failure x tubular atrophy	Exclude national registries	3	HR 1.27 (0.07–21.69)	83.2%	0.752	Below primary retains exploratory pool
Graft failure x tubular atrophy	Exclude uncorrected or unknown clustering	3	HR 1.27 (0.07–21.69)	83.2%	0.752	Below primary retains exploratory pool
Graft failure x tubular atrophy	Exclude uncorrected paired-kidney effects	3	HR 1.27 (0.07–21.69)	83.2%	0.752	Below primary retains exploratory pool
Graft failure x tubular atrophy	Wedge biopsy only	3	HR 1.27 (0.07–21.69)	83.2%	0.752	Below primary retains exploratory pool
Graft failure x vascular injury	Analytic n >=200	7	HR 1.49 (1.15–1.94)	48.6%	0.010	Retains primary threshold
Graft failure x vascular injury	Baseline-adjusted only	4	HR 1.42 (0.75–2.71)	63.4%	0.181	Below primary retains exploratory pool
Graft failure x vascular injury	Exclude national registries	7	HR 1.70 (1.13–2.54)	22.0%	0.019	Retains primary threshold
Graft failure x vascular injury	Exclude uncorrected or unknown clustering	6	HR 1.83 (0.94–3.57)	43.0%	0.067	Retains primary threshold
Graft failure x vascular injury	Exclude uncorrected paired-kidney effects	9	HR 1.48 (1.07–2.04)	53.0%	0.023	Retains primary threshold
Graft failure x vascular injury	Expert or central reading only	5	HR 1.61 (0.71–3.68)	67.7%	0.183	Retains primary threshold
Graft failure x vascular injury	Paraffin processing only	3	HR 1.56 (1.08–2.27)	0.0%	0.035	Below primary retains exploratory pool
Graft failure x vascular injury	Pre-Banff-2017 era only	5	HR 1.48 (0.72–3.07)	60.7%	0.209	Retains primary threshold
Graft failure x vascular injury	Wedge biopsy only	4	HR 1.56 (0.41–5.91)	72.1%	0.364	Below primary retains exploratory pool
Low eGFR at 9–15 months x IFTA	Exclude uncorrected paired-kidney effects	3	OR 1.33 (0.12–15.20)	86.5%	0.662	Below primary retains exploratory pool
Low eGFR at 9–15 months x vascular injury	Exclude uncorrected paired-kidney effects	3	OR 1.49 (0.31–7.18)	63.9%	0.387	Below primary retains exploratory pool

Abbreviations and symbols: CI, confidence interval; DGF, delayed graft function; eGFR, estimated glomerular filtration rate; HR, hazard ratio; I^2, percentage of total variability attributable to between-study heterogeneity; IFTA, interstitial fibrosis and tubular atrophy; k, number of cohorts in the analytic cell; n, reported sample size; OR, odds ratio.

Table S11 (continued). Adjustment-status moderator audit

Adjustment status was prespecified, but no parent body met the locked requirements for meta-regression. Descriptive adjusted-versus-crude subgroup estimates are shown without a moderation claim in Supplementary Figure S5.

Parent outcome and lesion	Parent k	Adjusted k	Crude k	Status	Locked requirement
Delayed graft function x IFTA	6	2	4	Not executable	Parent k>=10 and >=4 per category
Delayed graft function x Remuzzi score	3	1	2	Not executable	Parent k>=10 and >=4 per category
Delayed graft function x acute tubular injury	5	2	3	Not executable	Parent k>=10 and >=4 per category
Delayed graft function x glomerulosclerosis	8	3	5	Not executable	Parent k>=10 and >=4 per category
Delayed graft function x vascular injury	7	4	3	Not executable	Parent k>=10 and >=4 per category
Delayed graft function x vascular injury (per grade)	3	2	1	Not executable	Parent k>=10 and >=4 per category

Parent outcome and lesion	Parent k	Adjusted k	Crude k	Status	Locked requirement
Graft failure x IFTA	6	2	4	Not executable	Parent k>=10 and >=4 per category
Graft failure x glomerulosclerosis	9	4	5	Not executable	Parent k>=10 and >=4 per category
Graft failure x tubular atrophy	3	0	3	Not executable	Parent k>=10 and >=4 per category
Graft failure x vascular injury	9	4	5	Not executable	Parent k>=10 and >=4 per category
Low eGFR at 9–15 months x IFTA	3	2	1	Not executable	Parent k>=10 and >=4 per category
Low eGFR at 9–15 months x vascular injury	3	2	1	Not executable	Parent k>=10 and >=4 per category

Abbreviations and symbols: eGFR, estimated glomerular filtration rate; IFTA, interstitial fibrosis and tubular atrophy; k, number of cohorts in the analytic cell.

Table S12. Complete leave-one-out and influence summary

All 65 cohort omissions are accounted for by parent body. Fifty omissions retained at least three cohorts and were modeled; 15 left two cohorts and therefore received no pooled summary. Supplementary Figure S6 prints every omission-level estimate.

Parent outcome and lesion	Parent k	Omissions	Executed	CI excludes null	Range of omission estimates	Influential cohorts
Delayed graft function x IFTA	6	6	6	1	1.37–2.14	2
Delayed graft function x Remuzzi score	3	3	0	0	Not pooled after omission	1
Delayed graft function x acute tubular injury	5	5	5	1	3.14–8.03	1
Delayed graft function x glomerulosclerosis	8	8	8	0	0.88–1.28	1
Delayed graft function x vascular injury	7	7	7	4	1.49–2.25	2
Delayed graft function x vascular injury (per grade)	3	3	0	0	Not pooled after omission	3
Graft failure x IFTA	6	6	6	2	1.45–1.77	1
Graft failure x glomerulosclerosis	9	9	9	9	1.63–2.29	1
Graft failure x tubular atrophy	3	3	0	0	Not pooled after omission	1
Graft failure x vascular injury	9	9	9	7	1.40–1.56	1
Low eGFR at 9–15 months x IFTA	3	3	0	0	Not pooled after omission	1
Low eGFR at 9–15 months x vascular injury	3	3	0	0	Not pooled after omission	1

Abbreviations and symbols: CI, confidence interval; eGFR, estimated glomerular filtration rate; IFTA, interstitial fibrosis and tubular atrophy; k, number of cohorts in the analytic cell.

Influence flags are diagnostics, not automatic exclusion rules. CI, confidence interval.

Table S13. All exactly matched clinical strata

The 65 effects entering pooled models formed 63 exact population–timing–outcome–horizon–construct–threshold strata. Every stratum is printed below; none retained three cohorts.

Parent outcome and lesion	Parent k	Exact-stratum k	Exact clinical signature	Member cohort(s)
Delayed graft function x IFTA	6	1	ALL DECEASED DONOR; PRE REPERFUSION; DGF; 0 7 DAYS; IFTA; BANFF CI GRADE; EXACT CATEGORICAL CONTRAST; OR	Li, 2024
Delayed graft function x IFTA	6	1	ALL DECEASED DONOR; PRE REPERFUSION; DGF; 0 7 DAYS; IFTA; INTERSTITIAL FIBROSIS PRESENT; EXACT CATEGORICAL CONTRAST; OR	Matignon, 2008
Delayed graft function x IFTA	6	1	ALL DECEASED DONOR; PRE REPERFUSION; DGF; WITHIN 7 DAYS; IFTA; MAPI COMPONENT INTERSTITIAL SCAR; EXACT CATEGORICAL CONTRAST; OR	Teixeira, 2021
Delayed graft function x IFTA	6	1	ALL DECEASED DONOR; PRE REPERFUSION; DGF DIALYSIS WITHIN 7D; WITHIN 7 DAYS; IFTA; BANFF OR STUDY SPECIFIC IFTA; IFTA MODERATE OR WORSE FAMILY; OR	Lee, 2026 (national OPTN/STAR cohort)
Delayed graft function x IFTA	6	1	ALL DECEASED DONOR; PRE REPERFUSION; DGF DIALYSIS WITHIN 7D; WITHIN 7 DAYS; INTERSTITIAL FIBROSIS; INTERSTITIAL FIBROSIS PRESENCE; BINARY PRESENCE VS ABSENCE; OR	Wang, 2022
Delayed graft function x IFTA	6	1	ALL DECEASED DONOR; PRE REPERFUSION; delayed vs primary function among grafts functioning at week 1; WEEK 1; INTERSTITIAL FIBROSIS; INTERSTITIAL FIBROSIS; any interstitial fibrosis vs none; OR	Lubuska, 2006
Delayed graft function x Remuzzi score	3	1	ALL DECEASED DONOR; PRE REPERFUSION; DGF DIALYSIS WITHIN 7D; 0 7 DAYS; SCORE REMUZZI; REMUZZI; Exact Remuzzi score; median 2, range 1–2; OR	Pavlovic, 2022
Delayed graft function x Remuzzi score	3	1	ALL DECEASED DONOR; PRE REPERFUSION; DGF DIALYSIS WITHIN 7D; WITHIN 7 DAYS; SCORE REMUZZI; REMUZZI; Exact comparator: moderate/severe grade versus mild grade; OR	Tan, 2024 (full report cohort)
Delayed graft function x Remuzzi score	3	1	ALL DECEASED DONOR; PRE REPERFUSION; DGF STUDY DEFINED OR UNCLEAR; EARLY STUDY DEFINED; SCORE REMUZZI; REMUZZI; Remuzzi band 4–6 versus 0–3; OR	Chen, 2021
Delayed graft function x acute tubular injury	5	1	ALL DECEASED DONOR; PRE REPERFUSION; DGF; 0 7 DAYS; ACUTE TUBULAR INJURY; ACUTE TUBULAR NECROSIS PRESENT; EXACT CATEGORICAL CONTRAST; OR	Matignon, 2008
Delayed graft function x acute tubular injury	5	1	ALL DECEASED DONOR; PRE REPERFUSION; DGF; 0 7 DAYS; ACUTE TUBULAR INJURY; ATI GRADE; EXACT CATEGORICAL CONTRAST; OR	Li, 2024
Delayed graft function x acute tubular injury	5	1	ALL DECEASED DONOR; PRE REPERFUSION; DGF; DGF INDEX HOSPITALIZATION; ACUTE TUBULAR INJURY; ATI SEVERITY GRADE; EXACT CATEGORICAL CONTRAST; OR	Wang, 2022
Delayed graft function x acute tubular injury	5	1	ALL DECEASED DONOR; PRE REPERFUSION; DGF DIALYSIS WITHIN 7D; 0 7 DAYS; ACUTE TUBULAR NECROSIS; STUDY SPECIFIC ROUTINE HISTOLOGY; MODERATE SEVERE VS MILD NONE; OR	Mourão, 2016 (preimplantation-biopsy analysis)
Delayed graft function x acute tubular injury	5	1	ALL DECEASED DONOR; PRE REPERFUSION; DGF DIALYSIS WITHIN 7D; 0 7 DAYS; ACUTE TUBULAR NECROSIS; procurement morphology; EXACT COMPARATOR ATN APPARENT VS ABSENT; OR	Rohr, 1983
Delayed graft function x glomerulosclerosis	8	1	ALL DECEASED DONOR; PRE REPERFUSION; DGF; 0 7 DAYS; GS; GLOMERULOSCLEROSIS GT20; EXACT CATEGORICAL CONTRAST; OR	Matignon, 2008
Delayed graft function x glomerulosclerosis	8	1	ALL DECEASED DONOR; PRE REPERFUSION; DGF; 0 7 DAYS; GS; PERCENT GS GRADE; EXACT CATEGORICAL CONTRAST; OR	Li, 2024
Delayed graft function x glomerulosclerosis	8	1	ALL DECEASED DONOR; PRE REPERFUSION; DGF; UNKNOWN HORIZON; GS; UNKNOWN INSTRUMENT; GS >=20% vs <20%; OR	Lu, 2000
Delayed graft function x glomerulosclerosis	8	1	ALL DECEASED DONOR; PRE REPERFUSION; DGF DIALYSIS WITHIN 7D; WITHIN 7 DAYS; GLOMERULOSCLEROSIS; PERCENT GS; BINARY PRESENCE VS ABSENCE; OR	Wang, 2022
Delayed graft function x glomerulosclerosis	8	1	ALL DECEASED DONOR; PRE REPERFUSION; DGF DIALYSIS WITHIN 7D; WITHIN 7 DAYS; GLOMERULOSCLEROSIS; PERCENT GS; GS GT15 THRESHOLD FAMILY; OR	Teixeira, 2021
Delayed graft function x glomerulosclerosis	8	1	ALL DECEASED DONOR; PRE REPERFUSION; DGF DIALYSIS WITHIN 7D; WITHIN 7 DAYS; GLOMERULOSCLEROSIS; PERCENT GS; GS GT20 THRESHOLD FAMILY; OR	Lee, 2026 (national OPTN/STAR cohort)
Delayed graft function x glomerulosclerosis	8	1	ALL DECEASED DONOR; PRE REPERFUSION; delayed graft function; MAIN DGF (dialysis within 7 days or study-defined); GLOMERULOSCLEROSIS THRESHOLD; GLOMERULOSCLEROSIS THRESHOLD; GS >20% vs 11-20%; OR	Cheungpasitporn, 2020
Delayed graft function x glomerulosclerosis	8	1	ALL DECEASED DONOR; PRE REPERFUSION; delayed vs primary function among grafts functioning at week 1; WEEK 1; GLOMERULOSCLEROSIS GE20; GLOMERULOSCLEROSIS GE20; GS >=20% vs <20%; OR	Lubuska, 2006
Delayed graft function x vascular injury	7	1	ALL DECEASED DONOR; PRE REPERFUSION; DGF; 0 7 DAYS; VASCULAR; ARTERIAL INTIMAL HYPERPLASIA GRADE; EXACT CATEGORICAL CONTRAST; OR	Li, 2024
Delayed graft function x vascular injury	7	1	ALL DECEASED DONOR; PRE REPERFUSION; DGF; UNKNOWN HORIZON; VASCULAR; UNKNOWN INSTRUMENT; vascular more-than-mild vs none-to-mild; OR	Lee, 2026 (national OPTN/STAR cohort)

Parent outcome and lesion	Parent k	Exact-stratum k	Exact clinical signature	Member cohort(s)
Delayed graft function x vascular injury	7	1	ALL DECEASED DONOR; PRE REPERFUSION; DGF; WITHIN 7 DAYS; VASCULAR; MAPI COMPONENT ARTERIOLAR HYALINOSIS; EXACT CATEGORICAL CONTRAST; OR	Teixeira, 2021
Delayed graft function x vascular injury	7	2	ALL DECEASED DONOR; PRE REPERFUSION; DGF DIALYSIS WITHIN 7D; 0 7 DAYS; ARTERIOLAR HYALINOSIS; BANFF AH OR STUDY SPECIFIC; AH PRESENT VS ABSENT FAMILY; OR	Lee, 2016; Matignon, 2008
Delayed graft function x vascular injury	7	1	ALL DECEASED DONOR; PRE REPERFUSION; DGF DIALYSIS WITHIN 7D; WITHIN 7 DAYS; ARTERIAL INTIMAL FIBROSIS; ARTERIAL INTIMAL FIBROSIS PRESENCE; BINARY PRESENCE VS ABSENCE; OR	Wang, 2022
Delayed graft function x vascular injury	7	1	ALL DECEASED DONOR; PRE REPERFUSION; delayed vs primary function among grafts functioning at week 1; WEEK 1; ARTERIAL DAMAGE; ARTERIAL DAMAGE; any arterial damage vs none; OR	Lubuska, 2006
Delayed graft function x vascular injury (per grade)	3	1	ALL DECEASED DONOR; PRE REPERFUSION; DGF DIALYSIS WITHIN 7D; 0 7 DAYS; ARTERIAL INTIMAL FIBROSIS; ARTERIAL INTIMAL FIBROSIS GRADE; ORDINAL VASCULAR SEVERITY PER GRADE; OR	Perez-Gutierrez, 2021
Delayed graft function x vascular injury (per grade)	3	1	ALL DECEASED DONOR; PRE REPERFUSION; DGF DIALYSIS WITHIN 7D; 0 7 DAYS; ARTERIOLE NARROWING; STUDY SPECIFIC ARTERIOLE NARROWING GRADE 0 3; CONTINUOUS OTHER UNIT; OR	Zheng, 2020
Delayed graft function x vascular injury (per grade)	3	1	ALL DECEASED DONOR; PRE REPERFUSION; DGF STUDY DEFINED OR UNCLEAR; EARLY STUDY DEFINED; VASCULAR MAX ARTERY OR ARTERIOLE; STUDY SPECIFIC MAX ARTERY OR ARTERIOLE GRADE 0 3; ORDINAL PER GRADE; OR	Di Paolo, 2002
Graft failure x IFTA	6	1	ALL DECEASED DONOR; PRE REPERFUSION; DEATH CENSORED GRAFT FAILURE; TTE LONGEST ELIGIBLE; IFTA; BANFF OR STUDY SPECIFIC IFTA; IFTA MODERATE OR WORSE FAMILY; HR	Reese, 2021
Graft failure x IFTA	6	1	ALL DECEASED DONOR; PRE REPERFUSION; DEATH CENSORED GRAFT FAILURE; TTE LONGEST ELIGIBLE; IFTA; BANFF OR STUDY SPECIFIC IFTA; IFTA PRESENT OR MILD PLUS FAMILY; HR	Munivenkatappa, 2008
Graft failure x IFTA	6	1	ALL DECEASED DONOR; PRE REPERFUSION; GRAFT FAILURE; TTE LONGEST ELIGIBLE; IFTA; IFTA MORE THAN MILD; EXACT CATEGORICAL CONTRAST; HR	Lee, 2026 (national OPTN/STAR cohort)
Graft failure x IFTA	6	1	ALL DECEASED DONOR; PRE REPERFUSION; GRAFT FAILURE; TTE LONGEST ELIGIBLE; IFTA; INTERSTITIAL FIBROSIS; EXACT CATEGORICAL CONTRAST; HR	Chen, 2021
Graft failure x IFTA	6	1	ALL DECEASED DONOR; PRE REPERFUSION; GRAFT FAILURE; TTE LONGEST ELIGIBLE; IFTA; SPANISH SCORE INTERSTITIAL FIBROSIS COMPONENT; EXACT CATEGORICAL CONTRAST; HR	Villanego, 2022
Graft failure x IFTA	6	1	ALL DECEASED DONOR; PRE REPERFUSION; GRAFT FAILURE DEFINITION UNCLEAR; TTE LONGEST ELIGIBLE; INTERSTITIAL FIBROSIS; BANFF INTERSTITIAL FIBROSIS PERCENT CATEGORY; IFTA MODERATE OR WORSE FAMILY; HR	Pegas, 2018
Graft failure x glomerulosclerosis	9	1	ALL DECEASED DONOR; PRE REPERFUSION; DEATH CENSORED GRAFT FAILURE; TTE LONGEST ELIGIBLE; GLOMERULOSCLEROSIS; PERCENT GS; BINARY PRESENCE VS ABSENCE; HR	Villanego, 2022
Graft failure x glomerulosclerosis	9	1	ALL DECEASED DONOR; PRE REPERFUSION; DEATH CENSORED GRAFT FAILURE; TTE LONGEST ELIGIBLE; GLOMERULOSCLEROSIS; PERCENT GS; GS GT10 THRESHOLD FAMILY; HR	Chen, 2021
Graft failure x glomerulosclerosis	9	1	ALL DECEASED DONOR; PRE REPERFUSION; DEATH CENSORED GRAFT FAILURE; TTE LONGEST ELIGIBLE; GLOMERULOSCLEROSIS; PERCENT GS; GS GT15 THRESHOLD FAMILY; HR	Munivenkatappa, 2008
Graft failure x glomerulosclerosis	9	1	ALL DECEASED DONOR; PRE REPERFUSION; GRAFT FAILURE; TTE LONGEST ELIGIBLE; GS; BANFF GS PERCENT; EXACT CATEGORICAL CONTRAST; HR	Reese, 2021
Graft failure x glomerulosclerosis	9	1	ALL DECEASED DONOR; PRE REPERFUSION; GRAFT FAILURE; TTE LONGEST ELIGIBLE; GS; PERCENT GS; EXACT CATEGORICAL CONTRAST; HR	Lee, 2026 (national OPTN/STAR cohort)
Graft failure x glomerulosclerosis	9	1	ALL DECEASED DONOR; PRE REPERFUSION; GRAFT FAILURE; TTE LONGEST ELIGIBLE; GS; PERCENT GS CATEGORY; EXACT CATEGORICAL CONTRAST; HR	Pegas, 2018
Graft failure x glomerulosclerosis	9	1	ALL DECEASED DONOR; PRE REPERFUSION; GRAFT FAILURE DEFINITION UNCLEAR; TTE LONGEST ELIGIBLE; GLOMERULOSCLEROSIS; PERCENT GS; EXACT ADVANCED GLOMERULOSCLEROSIS NOT QUANTIFIED; HR	Lin, 2010
Graft failure x glomerulosclerosis	9	1	ALL DECEASED DONOR; PRE REPERFUSION; GRAFT FAILURE DEFINITION UNCLEAR; TTE LONGEST ELIGIBLE; GLOMERULOSCLEROSIS; PERCENT GS; GS GT15 THRESHOLD FAMILY; HR	Teixeira, 2021
Graft failure x glomerulosclerosis	9	1	ALL DECEASED DONOR; PRE REPERFUSION; death-censored graft failure; LONGEST ELIGIBLE FOLLOW-UP; GLOMERULOSCLEROSIS THRESHOLD; GLOMERULOSCLEROSIS THRESHOLD; GS >20% vs 11-20%; HR	Cheungpasitporn, 2020
Graft failure x tubular atrophy	3	1	ALL DECEASED DONOR; PRE REPERFUSION; DEATH CENSORED GRAFT FAILURE; TTE LONGEST ELIGIBLE; TUBULAR ATROPHY; TUBULAR ATROPHY PRESENT ABSENT; IFTA PRESENT OR MILD PLUS FAMILY; HR	Chen, 2021
Graft failure x tubular atrophy	3	1	ALL DECEASED DONOR; PRE REPERFUSION; GRAFT FAILURE; TTE LONGEST ELIGIBLE; TUBULAR ATROPHY; BANFF TUBULAR ATROPHY PERCENT CATEGORY; EXACT CATEGORICAL CONTRAST; HR	Pegas, 2018
Graft failure x tubular atrophy	3	1	ALL DECEASED DONOR; PRE REPERFUSION; GRAFT FAILURE; TTE LONGEST ELIGIBLE; TUBULAR ATROPHY; SPANISH SCORE TUBULAR ATROPHY COMPONENT; EXACT CATEGORICAL CONTRAST; HR	Villanego, 2022

Parent outcome and lesion	Parent k	Exact-stratum k	Exact clinical signature	Member cohort(s)
Graft failure x vascular injury	9	1	ALL DECEASED DONOR; PRE REPERFUSION; DEATH CENSORED GRAFT FAILURE; TTE LONGEST ELIGIBLE; VASCULAR; STUDY SPECIFIC CHRONIC VASCULAR CHANGE CATEGORY; EXACT CATEGORICAL CONTRAST; HR	Stewart, 2022
Graft failure x vascular injury	9	2	ALL DECEASED DONOR; PRE REPERFUSION; DEATH CENSORED GRAFT FAILURE; TTE LONGEST ELIGIBLE; VASCULAR UNSPECIFIED; VASCULAR UNSPECIFIED; BINARY PRESENCE VS ABSENCE; HR	Villanego, 2022; Chen, 2021
Graft failure x vascular injury	9	1	ALL DECEASED DONOR; PRE REPERFUSION; DEATH CENSORED GRAFT FAILURE; TTE LONGEST ELIGIBLE; VASCULAR UNSPECIFIED; VASCULAR UNSPECIFIED; EXACT COMPARATOR WLR INTERLOBULAR 0 5; HR	Munivenkatappa, 2008
Graft failure x vascular injury	9	1	ALL DECEASED DONOR; PRE REPERFUSION; GRAFT FAILURE; ONE YEAR; VASCULAR; MAPI COMPONENT ARTERIOLAR HYALINOSIS; EXACT CATEGORICAL CONTRAST; HR	Teixeira, 2021
Graft failure x vascular injury	9	1	ALL DECEASED DONOR; PRE REPERFUSION; GRAFT FAILURE; TTE LONGEST ELIGIBLE; VASCULAR; BANFF ARTERIOSCLEROSIS PERCENT CATEGORY; EXACT CATEGORICAL CONTRAST; HR	Pegas, 2018
Graft failure x vascular injury	9	1	ALL DECEASED DONOR; PRE REPERFUSION; GRAFT FAILURE; TTE LONGEST ELIGIBLE; VASCULAR; BANFF CV; EXACT CATEGORICAL CONTRAST; HR	Reese, 2021
Graft failure x vascular injury	9	1	ALL DECEASED DONOR; PRE REPERFUSION; GRAFT FAILURE; TTE LONGEST ELIGIBLE; VASCULAR; VASCULAR CHANGES MORE THAN MILD; EXACT CATEGORICAL CONTRAST; HR	Lee, 2026 (national OPTN/STAR cohort)
Graft failure x vascular injury	9	1	ALL DECEASED DONOR; PRE REPERFUSION; GRAFT FAILURE DEFINITION UNCLEAR; TTE LONGEST ELIGIBLE; ARTERIOLAR HYALINOSIS; BANFF AH OR STUDY SPECIFIC; AH PRESENT VS ABSENT FAMILY; HR	Wazna, 2018
Low eGFR at 9–15 months x IFTA	3	1	ALL DECEASED DONOR; PRE REPERFUSION; EGFR 12M BINARY; 9 15 MONTHS; IFTA; BANFF CI GRADE; EXACT CATEGORICAL CONTRAST; OR	Proença, 2025
Low eGFR at 9–15 months x IFTA	3	1	ALL DECEASED DONOR; PRE REPERFUSION; EGFR 12M BINARY; 9 15 MONTHS; IFTA; IFTA MORE THAN MILD; EXACT CATEGORICAL CONTRAST; OR	Lee, 2026 (national OPTN/STAR cohort)
Low eGFR at 9–15 months x IFTA	3	1	ALL DECEASED DONOR; PRE REPERFUSION; EGFR 12M BINARY; 9 15 MONTHS; IFTA; MAPI COMPONENT INTERSTITIAL SCAR; EXACT CATEGORICAL CONTRAST; OR	Teixeira, 2021
Low eGFR at 9–15 months x vascular injury	3	1	ALL DECEASED DONOR; PRE REPERFUSION; EGFR 12M BINARY; 9 15 MONTHS; VASCULAR; BANFF AH GRADE; EXACT CATEGORICAL CONTRAST; OR	Proença, 2025
Low eGFR at 9–15 months x vascular injury	3	1	ALL DECEASED DONOR; PRE REPERFUSION; EGFR 12M BINARY; 9 15 MONTHS; VASCULAR; MAPI COMPONENT ARTERIOLAR HYALINOSIS; EXACT CATEGORICAL CONTRAST; OR	Teixeira, 2021
Low eGFR at 9–15 months x vascular injury	3	1	ALL DECEASED DONOR; PRE REPERFUSION; EGFR 12M BINARY; 9 15 MONTHS; VASCULAR; VASCULAR CHANGES MORE THAN MILD; EXACT CATEGORICAL CONTRAST; OR	Lee, 2026 (national OPTN/STAR cohort)

Abbreviations and symbols: 12M, 12 months; AH, arteriolar hyalinosis; ATI, acute tubular injury; ATN, acute tubular necrosis; CI, confidence interval; CV, Banff vascular fibrous intimal thickening score; DGF, delayed graft function; eGFR, estimated glomerular filtration rate; GE, greater than or equal to; GS, glomerulosclerosis; GT, greater than; HR, hazard ratio; IFTA, interstitial fibrosis and tubular atrophy; k, number of cohorts in the analytic cell; MAPI, Maryland Aggregate Pathology Index; OPTN, Organ Procurement and Transplantation Network; OR, odds ratio; STAR, Standard Transplant Analysis and Research; TTE, time to event; VS, versus; WLR, wall-to-lumen ratio. Capitalized stratum descriptors are retained analysis codes; numeric suffixes on GE/GT specify the original percentage thresholds.

Sixty-one strata contained one cohort and two contained two cohorts. Exact matching was a prespecified sensitivity, not the primary synthesis.

Table S13 (continued). Small-study and publication-bias assessment

All 12 pooled bodies were assessed against the locked minimum of ten cohorts for formal asymmetry testing. No body reached that threshold.

Outcome and lesion	k	Minimum k	Status	Interpretation
Delayed graft function x IFTA	6	10	Not executable	No formal asymmetry test or funnel-plot inference under the locked threshold.
Delayed graft function x Remuzzi score	3	10	Not executable	No formal asymmetry test or funnel-plot inference under the locked threshold.
Delayed graft function x acute tubular injury	5	10	Not executable	No formal asymmetry test or funnel-plot inference under the locked threshold.
Delayed graft function x glomerulosclerosis	8	10	Not executable	No formal asymmetry test or funnel-plot inference under the locked threshold.
Delayed graft function x vascular injury	7	10	Not executable	No formal asymmetry test or funnel-plot inference under the locked threshold.
Delayed graft function x vascular injury (per grade)	3	10	Not executable	No formal asymmetry test or funnel-plot inference under the locked threshold.
Graft failure x IFTA	6	10	Not executable	No formal asymmetry test or funnel-plot inference under the locked threshold.
Graft failure x glomerulosclerosis	9	10	Not executable	No formal asymmetry test or funnel-plot inference under the locked threshold.
Graft failure x tubular atrophy	3	10	Not executable	No formal asymmetry test or funnel-plot inference under the locked threshold.
Graft failure x vascular injury	9	10	Not executable	No formal asymmetry test or funnel-plot inference under the locked threshold.
Low eGFR at 9–15 months x IFTA	3	10	Not executable	No formal asymmetry test or funnel-plot inference under the locked threshold.
Low eGFR at 9–15 months x vascular injury	3	10	Not executable	No formal asymmetry test or funnel-plot inference under the locked threshold.

Abbreviations and symbols: eGFR, estimated glomerular filtration rate; IFTA, interstitial fibrosis and tubular atrophy; k, number of cohorts in the analytic cell.

Publication bias was not statistically assessable; this is not evidence that publication bias was absent.

Table S14A. Complete prognostic Summary of Findings

All 12 pooled prognostic bodies are reported, rather than only the selected outcomes shown in Main Table 3. Absolute effects are presented only for DGF odds ratios; graft-failure hazard ratios were not converted without an aligned baseline survival function and horizon.

Role	Outcome and lesion	k	Participants	Relative effect (95% CI)	Prediction interval	Illustrative absolute effect	Certainty	Interpretation
Principal	Delayed graft function x acute tubular injury	5	>=804 known; 2 unknown and 0 complex cohort denominators	OR 5.04 (0.82 to 30.86)	0.09 to 292.15	baseline 20%: +358 per 1000; baseline 30%: +384 per 1000; baseline 40%: +371 per 1000	Very low	The pooled best-available association was inconclusive (OR 5.04, 95% CI 0.82 to 30.86). At a hypothetical 30% baseline DGF risk, the point estimate corresponds to +384 events per 1000, with an interval from -39 to +630. Certainty is very low; this evidence does not validate a stand-alone acceptance or discard rule.
Principal	Delayed graft function x glomerulosclerosis	8	>=16744 known; 1 unknown and 0 complex cohort denominators	OR 1.13 (0.59 to 2.17)	0.32 to 3.99	baseline 20%: +20 per 1000; baseline 30%: +26 per 1000; baseline 40%: +30 per 1000	Very low	The pooled best-available association was inconclusive (OR 1.13, 95% CI 0.59 to 2.17). At a hypothetical 30% baseline DGF risk, the point estimate corresponds to +26 events per 1000, with an interval from -98 to +181. Certainty is very low; this evidence does not validate a stand-alone acceptance or discard rule.
Principal	Delayed graft function x IFTA	6	>=14925 known; 1 unknown and 0 complex cohort denominators	OR 1.78 (0.80 to 3.98)	0.31 to 10.41	baseline 20%: +108 per 1000; baseline 30%: +133 per 1000; baseline 40%: +143 per 1000	Very low	The pooled best-available association was inconclusive (OR 1.78, 95% CI 0.80 to 3.98). At a hypothetical 30% baseline DGF risk, the point estimate corresponds to +133 events per 1000, with an interval from -45 to +330. Certainty is very low; this evidence does not validate a stand-alone acceptance or discard rule.
Principal	Delayed graft function x vascular injury	7	>=15060 known; 1 unknown and 0 complex cohort denominators	OR 1.85 (1.11 to 3.06)	0.53 to 6.41	baseline 20%: +116 per 1000; baseline 30%: +142 per 1000; baseline 40%: +152 per 1000	Very low	The pooled best-available association excluded the null (OR 1.85, 95% CI 1.11 to 3.06). At a hypothetical 30% baseline DGF risk, the point estimate corresponds to +142 events per 1000, with an interval from +23 to +267. Certainty is very low; this evidence does not validate a stand-alone acceptance or discard rule.
Principal	Graft failure x glomerulosclerosis	9	>=17034 known; 0 unknown and 1 complex cohort denominators	HR 2.03 (1.22 to 3.39)	0.52 to 7.91	Not estimable on an absolute-risk scale from the available inputs	Very low	The pooled best-available association excluded the null (HR 2.03, 95% CI 1.22 to 3.39). Certainty is very low; this evidence does not validate a stand-alone acceptance or discard rule.
Principal	Graft failure x IFTA	6	16,719	HR 1.64 (0.96 to 2.82)	0.64 to 4.20	Not estimable on an absolute-risk scale from the available inputs	Very low	The pooled best-available association was inconclusive (HR 1.64, 95% CI 0.96 to 2.82). Certainty is very low; this evidence does not validate a stand-alone acceptance or discard rule.
Principal	Graft failure x vascular injury	9	>=21546 known; 0 unknown and 1 complex cohort denominators	HR 1.48 (1.07 to 2.04)	0.80 to 2.71	Not estimable on an absolute-risk scale from the available inputs	Very low	The pooled best-available association excluded the null (HR 1.48, 95% CI 1.07 to 2.04). Certainty is very low; this evidence does not validate a stand-alone acceptance or discard rule.
Exploratory	Delayed graft function x Remuzzi score	3	343	OR 1.09 (0.63 to 1.89)	Not estimable (k<5)	baseline 20%: +15 per 1000; baseline 30%: +19 per 1000; baseline 40%: +22 per 1000	Very low	The pooled best-available association was inconclusive (OR 1.09, 95% CI 0.63 to 1.89). At a hypothetical 30% baseline DGF risk, the point estimate corresponds to +19 events per 1000, with an interval from -87 to +147. Certainty is very low; this evidence does not validate a stand-alone acceptance or discard rule.
Exploratory	Delayed graft function x vascular injury (per grade)	3	551	OR 1.98 (0.76 to 5.17)	Not estimable (k<5)	Not estimable on an absolute-risk scale from the available inputs	Very low	The pooled best-available association was inconclusive (OR 1.98, 95% CI 0.76 to 5.17). Certainty is very low; this evidence does not validate a stand-alone acceptance or discard rule.

Role	Outcome and lesion	k	Participants	Relative effect (95% CI)	Prediction interval	Illustrative absolute effect	Certainty	Interpretation
Exploratory	Low eGFR at 9–15 months x IFTA	3	14,460	OR 1.33 (0.12 to 15.20)	Not estimable (k<5)	Not estimable on an absolute-risk scale from the available inputs	Very low	The pooled best-available association was inconclusive (OR 1.33, 95% CI 0.12 to 15.20). Certainty is very low; this evidence does not validate a stand-alone acceptance or discard rule.
Exploratory	Low eGFR at 9–15 months x vascular injury	3	14,460	OR 1.49 (0.31 to 7.18)	Not estimable (k<5)	Not estimable on an absolute-risk scale from the available inputs	Very low	The pooled best-available association was inconclusive (OR 1.49, 95% CI 0.31 to 7.18). Certainty is very low; this evidence does not validate a stand-alone acceptance or discard rule.
Exploratory	Graft failure x tubular atrophy	3	869	HR 1.27 (0.07 to 21.69)	Not estimable (k<5)	Not estimable on an absolute-risk scale from the available inputs	Very low	The pooled best-available association was inconclusive (HR 1.27, 95% CI 0.07 to 21.69). Certainty is very low; this evidence does not validate a stand-alone acceptance or discard rule.

Abbreviations and symbols: CI, confidence interval; DGF, delayed graft function; eGFR, estimated glomerular filtration rate; HR, hazard ratio; IFTA, interstitial fibrosis and tubular atrophy; k, number of cohorts in the analytic cell; OR, odds ratio; PI, prediction interval.

Participant totals are effect-specific analytic denominators and are not deduplicated across bodies; unknown or complex denominators are stated explicitly. PI, prediction interval.

Table S14B. Complete prognostic certainty-domain profile

The cell-specific GRADE domain judgments and downgrade totals corresponding to Table S14A are shown below. Applied downgrading was capped at three levels, the categorical floor of very low certainty.

Outcome and lesion	Risk of bias	Inconsistency	Indirectness	Imprecision	Publication bias	Raw levels	Applied	Final certainty
Delayed graft function x acute tubular injury	Very serious	Very serious	Serious	Very serious	Not assessable (k<10)	7	3	Very low
Delayed graft function x glomerulosclerosis	Very serious	Serious	Serious	Very serious	Not assessable (k<10)	6	3	Very low
Delayed graft function x IFTA	Very serious	Serious	Serious	Very serious	Not assessable (k<10)	6	3	Very low
Delayed graft function x vascular injury	Serious	Serious	Serious	Serious	Not assessable (k<10)	4	3	Very low
Graft failure x glomerulosclerosis	Very serious	Very serious	Serious	Not serious	Not assessable (k<10)	5	3	Very low
Graft failure x IFTA	Very serious	Serious	Serious	Serious	Not assessable (k<10)	5	3	Very low
Graft failure x vascular injury	Very serious	Serious	Serious	Not serious	Not assessable (k<10)	4	3	Very low
Delayed graft function x Remuzzi score	Very serious	Not serious	Not serious	Very serious	Not assessable (k<10)	4	3	Very low
Delayed graft function x vascular injury (per grade)	Very serious	Not serious	Serious	Very serious	Not assessable (k<10)	5	3	Very low
Low eGFR at 9–15 months x IFTA	Very serious	Very serious	Serious	Very serious	Not assessable (k<10)	7	3	Very low
Low eGFR at 9–15 months x vascular injury	Very serious	Serious	Serious	Very serious	Not assessable (k<10)	6	3	Very low
Graft failure x tubular atrophy	Very serious	Very serious	Serious	Very serious	Not assessable (k<10)	7	3	Very low

Abbreviations and symbols: eGFR, estimated glomerular filtration rate; GRADE, Grading of Recommendations Assessment, Development and Evaluation; IFTA, interstitial fibrosis and tubular atrophy; k, number of cohorts in the analytic cell.

Prognostic-factor evidence began at high certainty. Confounding-related components were combined by their maximum supported downgrade rather than added twice; inconsistency, indirectness and imprecision remained separate.

Supplementary Figure S1. Two-cohort evidence

Two studies per cell; no pooled summary or meta-analytic weight. Symbols: † exact contingency total; ‡ analytic sample stated by source; unmarked cohort size.

S1.1. Acute tubular injury and delayed graft function (OR; dose per grade)

Study	n	Model	OR	95% CI	Weight
Zheng, 2020	—	Adj.	4.72	[2.32; 9.60]	—
Perez-Gutierrez, 2021	118‡	Adj.	1.10	[0.39; 2.43]	—

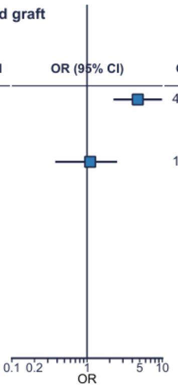

k=2: no pooled estimate or weight.

S1.2. Chronic composite and delayed graft function (OR; dichotomous)

Study	n	Model	OR	95% CI	Weight
Chen, 2021	173	Crude	0.73	[0.28; 1.87]	—
Tan, 2024	97	Adj.	0.99	[0.85; 1.15]	—

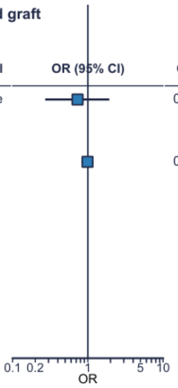

k=2: no pooled estimate or weight.

S1.3. Remuzzi score and delayed graft function (OR; dose per grade)

Study	n	Model	OR	95% CI	Weight
Losappio, 2014	372	Adj.	1.92	[1.02; 3.60]	—
Pavlovic, 2022	73	Crude	1.50	[0.54; 4.15]	—

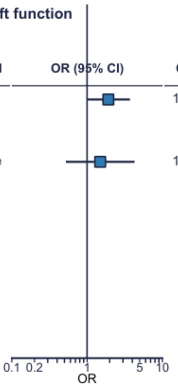

k=2: no pooled estimate or weight.

S1.4. Remuzzi score and delayed graft function (OR; dose per score point)

Study	n	Model	OR	95% CI	Weight
Weissenbacher, 2019	—	Adj.	1.04	[0.71; 1.52]	—
Zheng, 2020	333	Adj.	1.21	[1.02; 1.43]	—

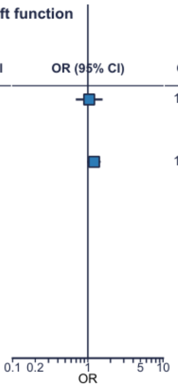

k=2: no pooled estimate or weight.

S1.5. Tubular atrophy and delayed graft function (OR; dichotomous)

Study	n	Model	OR	95% CI	Weight
Wang, 2022	140	Crude	2.05	[0.79; 5.10]	—
Li, 2024	458†	Recalc.	1.28	[0.75; 2.18]	—

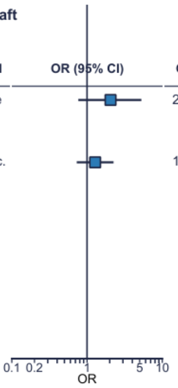

k=2: no pooled estimate or weight.

S1.6. Tubular atrophy and delayed graft function (OR; dose per grade)

Study	n	Model	OR	95% CI	Weight
Di Paolo, 2002	100‡	Adj.	1.27	[0.48; 3.39]	—
Perez-Gutierrez, 2021	118‡	Adj.	0.40	[0.05; 3.01]	—

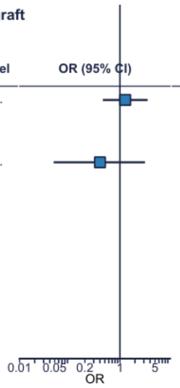

k=2: no pooled estimate or weight.

S1.7. Glomerulosclerosis and low eGFR at 9–15

Study	n	Model	OR	95% CI	Weight
Teixeira, 2021	159	Adj.	0.71	[0.15; 3.39]	—
Lee, 2026	13,962	Adj.	2.05	[1.39; 3.02]	—

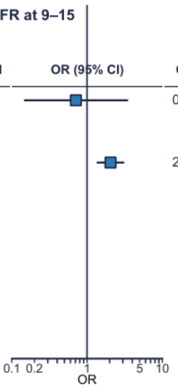

k=2: no pooled estimate or weight.

S1.8. Glomerulosclerosis and eGFR at 9–15

Study	n	Model	BETA	95% CI	Weight
Saha-Chaudhuri, 2020	145‡	Adj.	-55.81	[-112.77; 1.15]	—
Wang, 2022	140	Crude	-10.91	[-19.16; -2.66]	—

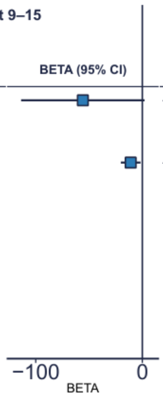

k=2: no pooled estimate or weight.

Abbreviations and symbols: Adj., adjusted estimate; BETA, regression coefficient; CI, confidence interval; eGFR, estimated glomerular filtration rate; k, number of cohorts in the analytic cell; n, reported sample size; OR, odds ratio; Recalc., estimate recalculated from source data. Squares mark study estimates and horizontal lines their confidence intervals.

Supplementary Figure S1. Two-cohort evidence (continued)

Two studies per cell; no pooled summary or meta-analytic weight. Symbols: † exact contingency total; ‡ analytic sample stated by source; unmarked cohort size.

S1.9. Tubular atrophy and eGFR at 9–15 months

Study	n	Model	BETA (95% CI)	BETA	95% CI	Weight
Saha-Chaudhuri, 2020	145‡	Adj.		-19.45	[-46.44; 7.54]	—
Wang, 2022	140	Crude		-4.62	[-13.70; 4.45]	—

k=2: no pooled estimate or weight.

S1.10. Glomerulosclerosis and all-cause graft failure (HR; dichotomous)

Study	n	Model	HR (95% CI)	HR	95% CI	Weight
Bajwa, 2007	12,129	Adj.		1.15	[1.06; 1.24]	—
Stewart, 2022	5,997	Adj.		1.18	[1.07; 1.28]	—

k=2: no pooled estimate or weight.

S1.11. IFTA and all-cause graft failure (HR;

Study	n	Model	HR (95% CI)	HR	95% CI	Weight
Hofer, 2014	628‡	Crude		0.97	[0.73; 1.29]	—
Sánchez-Escuredo, 2017	364	Adj.		3.06	[1.56; 6.03]	—

k=2: no pooled estimate or weight.

S1.12. Tubular atrophy and all-cause graft failure (HR; dose per grade)

Study	n	Model	HR (95% CI)	HR	95% CI	Weight
Hofer, 2014	628‡	Crude		1.05	[0.80; 1.40]	—
Sánchez-Escuredo, 2017	364	Adj.		1.72	[0.88; 3.35]	—

k=2: no pooled estimate or weight.

S1.13. Vascular injury and all-cause graft failure (HR; dose per grade)

Study	n	Model	HR (95% CI)	HR	95% CI	Weight
Hofer, 2014	628‡	Crude		0.98	[0.78; 1.24]	—
Sánchez-Escuredo, 2017	364	Adj.		1.37	[0.68; 2.77]	—

k=2: no pooled estimate or weight.

S1.14. Glomerulosclerosis and graft failure (HR; dose per 10pp)

Study	n	Model	HR (95% CI)	HR	95% CI	Weight
Munivenkatappa, 2008	259	Crude		1.48	[1.10; 1.97]	—
Pegas, 2018	430	Crude		1.63	[1.34; 1.97]	—

k=2: no pooled estimate or weight.

S1.15. Karpinski/MAPI score and graft failure

Study	n	Model	OR (95% CI)	OR	95% CI	Weight
Carta, 2013	100†	Recalc.		1.67	[0.47; 5.94]	—
Zanchetta, 2025	100†	Recalc.		1.02	[0.28; 3.66]	—

k=2: no pooled estimate or weight.

Abbreviations and symbols: Adj., adjusted estimate; BETA, regression coefficient; CI, confidence interval; eGFR, estimated glomerular filtration rate; HR, hazard ratio; IFTA, interstitial fibrosis and tubular atrophy; k, number of cohorts in the analytic cell; MAPI, Maryland Aggregate Pathology Index; n, reported sample size; OR, odds ratio; pp, percentage points; Recalc., estimate recalculated from source data. Squares mark study estimates and horizontal lines their confidence intervals.

Supplementary Figure S2. Principal pooled analyses

Squares show study estimates and confidence intervals; diamonds show pooled estimates. Separate lines show prediction intervals.

S2.1. Acute tubular injury and delayed graft function

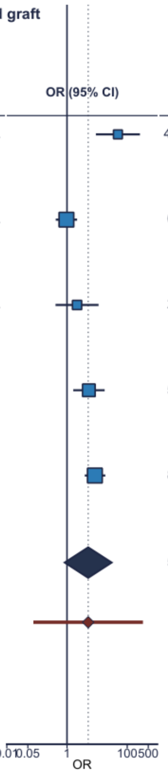

Study	n	Model	OR (95% CI)	OR	95% CI	Weight
Rohr, 1983	—	Recalc.		49.08	[9.89; 243.65]	17.4%
Matignon, 2008	172†	Recalc.		0.96	[0.46; 1.99]	22.3%
Mourão, 2016	—	Recalc.		2.15	[0.45; 10.29]	17.6%
Wang, 2022	140	Adj.		5.31	[1.75; 16.42]	20.3%
Li, 2024	492	Adj.		8.42	[4.22; 17.22]	22.5%
**Random-effects model**	**k=5**	**REML**		**5.04**	**[0.82; 30.86]**	**100%**
Prediction interval					[0.09; 292.15]	

I²=86.6%; τ²=1.712; P=0.068.

S2.2. Glomerulosclerosis and delayed graft function

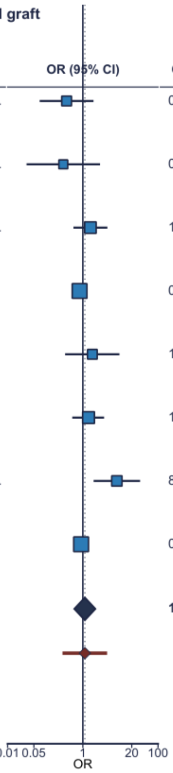

Study	n	Model	OR (95% CI)	OR	95% CI	Weight
Lu, 2000	89†	Recalc.		0.37	[0.08; 1.80]	6.4%
Lubuska, 2006	109†	Recalc.		0.30	[0.03; 2.68]	3.8%
Matignon, 2008	172†	Recalc.		1.59	[0.60; 4.21]	12.2%
Cheungpasitporn, 2020	1,730	Adj.		0.82	[0.65; 1.05]	24.8%
Teixeira, 2021	159	Adj.		1.79	[0.36; 8.81]	6.3%
Wang, 2022	140	Crude		1.42	[0.56; 3.44]	13.1%
Li, 2024	306†	Recalc.		8.03	[2.07; 31.18]	8.1%
Lee, 2026	13,962	Adj.		0.90	[0.73; 1.12]	25.2%
**Random-effects model**	**k=8**	**REML**		**1.13**	**[0.59; 2.17]**	**100%**
Prediction interval					[0.32; 3.99]	

I²=74.0%; τ²=0.208; P=0.670.

S2.3. IFTA and delayed graft function

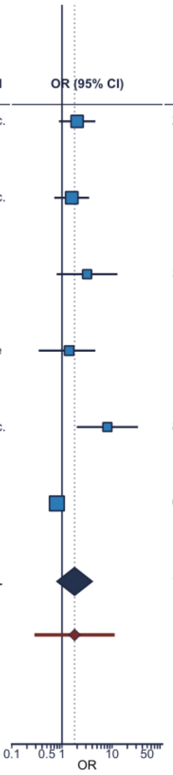

Study	n	Model	OR (95% CI)	OR	95% CI	Weight
Lubuska, 2006	109†	Recalc.		2.00	[0.91; 4.40]	18.7%
Matignon, 2008	172†	Recalc.		1.56	[0.74; 3.32]	19.2%
Teixeira, 2021	159	Adj.		3.16	[0.82; 12.13]	11.8%
Wang, 2022	140	Crude		1.39	[0.36; 4.40]	12.8%
Li, 2024	306†	Recalc.		8.03	[2.07; 31.18]	11.7%
Lee, 2026	13,962	Adj.		0.79	[0.62; 1.00]	25.7%
**Random-effects model**	**k=6**	**REML**		**1.78**	**[0.80; 3.98]**	**100%**
Prediction interval					[0.31; 10.41]	

I²=71.6%; τ²=0.373; P=0.124.

S2.4. Vascular injury and delayed graft function

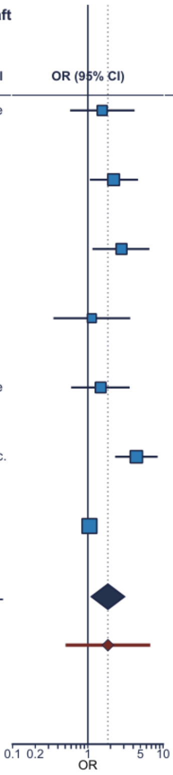

Study	n	Model	OR (95% CI)	OR	95% CI	Weight
Lubuska, 2006	109†	Crude		1.55	[0.60; 4.05]	11.3%
Matignon, 2008	172	Adj.		2.20	[1.10; 4.50]	14.9%
Lee, 2016	135	Adj.		2.80	[1.19; 6.37]	12.9%
Teixeira, 2021	159	Adj.		1.13	[0.36; 3.54]	9.3%
Wang, 2022	140	Crude		1.48	[0.62; 3.48]	12.6%
Li, 2024	303†	Recalc.		4.42	[2.38; 8.20]	16.3%
Lee, 2026	13,962	Adj.		1.05	[0.86; 1.27]	22.8%
**Random-effects model**	**k=7**	**REML**		**1.85**	**[1.11; 3.06]**	**100%**
Prediction interval					[0.53; 6.41]	

I²=68.4%; τ²=0.216; P=0.025.

Abbreviations and symbols: τ², between-study variance on the analysis scale; Adj., adjusted estimate; CI, confidence interval; I², percentage of total variability attributable to between-study heterogeneity; IFTA, interstitial fibrosis and tubular atrophy; k, number of cohorts in the analytic cell; n, reported sample size; OR, odds ratio; Recalc., estimate recalculated from source data; REML, restricted maximum likelihood. P values test the pooled association. †, exact contingency-table total; ‡, source-reported analytic sample; unmarked n, cohort size.

Supplementary Figure S2. Principal pooled analyses (continued)

Squares show study estimates and confidence intervals; diamonds show pooled estimates. Separate lines show prediction intervals.

S2.5. Glomerulosclerosis and graft failure

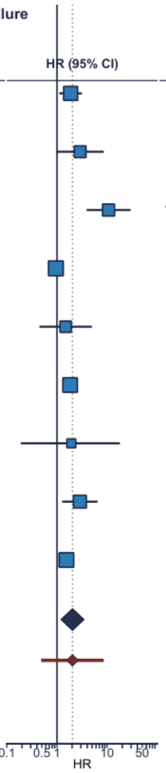

Study	n	Model	HR	95% CI	Weight
Munivenkatappa, 2008	259	Crude	1.87	[1.17; 2.99]	13.9%
Lin, 2010	55	Crude	2.89	[1.02; 8.17]	8.5%
Pegas, 2018	430	Crude	10.68	[4.09; 27.90]	9.1%
Cheungpasitporn, 2020	1,730	Adj.	0.95	[0.78; 1.15]	16.0%
Chen, 2021	173	Crude	1.49	[0.47; 4.70]	7.7%
Reese, 2021	1,629/1,107	Crude	1.83	[1.35; 2.47]	15.3%
Teixeira, 2021	159	Adj.	1.93	[0.20; 17.10]	3.1%
Villanego, 2022	266	Adj.	2.86	[1.33; 6.15]	10.9%
Lee, 2026	13,962	Adj.	1.54	[1.17; 2.03]	15.5%
**Random-effects model**	**k=9**	**REML**	**2.03**	**[1.22; 3.39]**	**100%**
Prediction interval				[0.52; 7.91]	

I^2=86.0%; τ^2=0.298; P=0.012.

S2.6. IFTA and graft failure

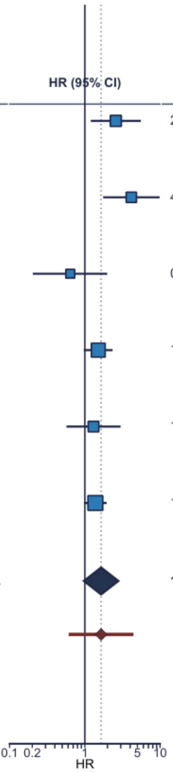

Study	n	Model	HR	95% CI	Weight
Munivenkatappa, 2008	259	Crude	2.58	[1.24; 5.38]	14.2%
Pegas, 2018	430	Crude	4.15	[1.81; 9.54]	12.1%
Chen, 2021	173	Crude	0.64	[0.21; 1.92]	8.0%
Reese, 2021	1,629	Adj.	1.51	[1.00; 2.26]	24.6%
Villanego, 2022	266	Crude	1.30	[0.59; 2.89]	12.8%
Lee, 2026	13,962	Adj.	1.38	[1.01; 1.89]	28.4%
**Random-effects model**	**k=6**	**REML**	**1.64**	**[0.96; 2.82]**	**100%**
Prediction interval				[0.64; 4.20]	

I^2=50.9%; τ^2=0.089; P=0.064.

S2.7. Vascular injury and graft failure

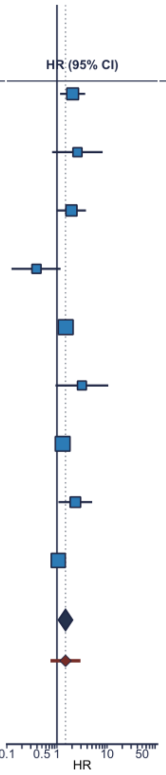

Study	n	Model	HR	95% CI	Weight
Munivenkatappa, 2008	259	Crude	2.05	[1.21; 3.47]	11.1%
Pegas, 2018	430	Crude	2.56	[0.84; 7.74]	3.6%
Wazna, 2018	300	Crude	1.94	[1.04; 3.60]	9.0%
Chen, 2021	173	Crude	0.39	[0.13; 1.12]	3.8%
Reese, 2021	1,629/1,107	Crude	1.49	[1.20; 1.86]	21.7%
Teixeira, 2021	159	Adj.	3.14	[0.98; 10.06]	3.4%
Stewart, 2022	5,997	Adj.	1.30	[1.02; 1.62]	21.2%
Villanego, 2022	266	Adj.	2.32	[1.12; 4.79]	7.2%
Lee, 2026	13,962	Adj.	1.06	[0.80; 1.41]	19.1%
**Random-effects model**	**k=9**	**REML**	**1.48**	**[1.07; 2.04]**	**100%**
Prediction interval				[0.80; 2.71]	

I^2=53.0%; τ^2=0.050; P=0.023.

Abbreviations and symbols: τ^2, between-study variance on the analysis scale; Adj., adjusted estimate; CI, confidence interval; HR, hazard ratio; I^2, percentage of total variability attributable to between-study heterogeneity; IFTA, interstitial fibrosis and tubular atrophy; k, number of cohorts in the analytic cell; n, reported sample size; REML, restricted maximum likelihood. P values test the pooled association. †, exact contingency-table total; ‡, source-reported analytic sample; unmarked n, cohort size.

Supplementary Figure S3. Focused DGF leave-one-cohort-out analyses

S3.A. Glomerulosclerosis and delayed graft function

Cohort omitted	k	OR (95% CI)	OR	95% CI	I²	Status
**Parent model**	**8**		**1.13**	**[0.59; 2.17]**	**74.0%**	**Parent**
Omit Cheungpasitporn, 2020	7		1.28	[0.54; 3.04]	62.4%	Executed
Omit Lee, 2026	7		1.25	[0.51; 3.05]	65.1%	Executed
**Omit Li, 2024**	**7**		**0.88**	**[0.73; 1.06]**	**0.0%**	**Executed**
Omit Lu, 2000	7		1.24	[0.61; 2.49]	78.6%	Executed
Omit Lubuska, 2006	7		1.21	[0.60; 2.45]	79.3%	Executed
Omit Matignon, 2008	7		1.11	[0.48; 2.53]	83.3%	Executed
Omit Teixeira, 2021	7		1.11	[0.51; 2.41]	80.7%	Executed
Omit Wang, 2022	7		1.13	[0.48; 2.64]	84.8%	Executed

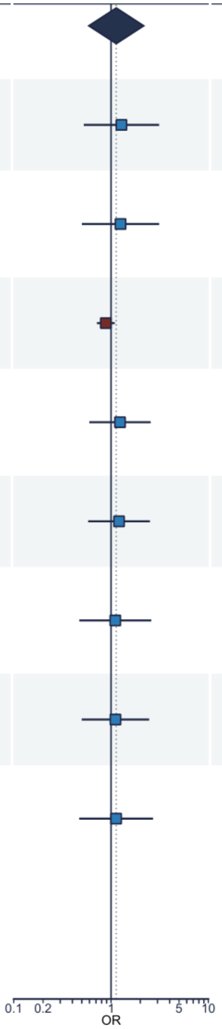

Focused LOO; Li 2024 highlighted.

S3.B. IFTA and delayed graft function

Cohort omitted	k	OR (95% CI)	OR	95% CI	I²	Status
**Parent model**	**6**		**1.78**	**[0.80; 3.98]**	**71.6%**	**Parent**
Omit Lee, 2026	5		2.14	[1.05; 4.35]	0.0%	Executed
**Omit Li, 2024**	**5**		**1.37**	**[0.72; 2.62]**	**58.1%**	**Executed**
Omit Lubuska, 2006	5		1.81	[0.61; 5.34]	75.6%	Executed
Omit Matignon, 2008	5		1.92	[0.65; 5.67]	75.5%	Executed
Omit Teixeira, 2021	5		1.66	[0.62; 4.47]	75.2%	Executed
Omit Wang, 2022	5		1.91	[0.68; 5.41]	78.5%	Executed

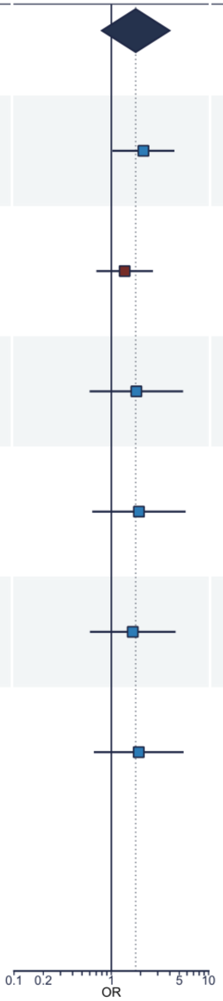

Focused LOO; Li 2024 highlighted.

Abbreviations and symbols: CI, confidence interval; DGF, delayed graft function; I², percentage of total variability attributable to between-study heterogeneity; IFTA, interstitial fibrosis and tubular atrophy; k, number of cohorts in the analytic cell; LOO, leave-one-cohort-out analysis; OR, odds ratio. Symbols mark estimates and horizontal lines their confidence intervals; Parent denotes the full pooled analysis.

Supplementary Figure S4. Prespecified sensitivity analyses

S4.1. Acute tubular injury and delayed graft function

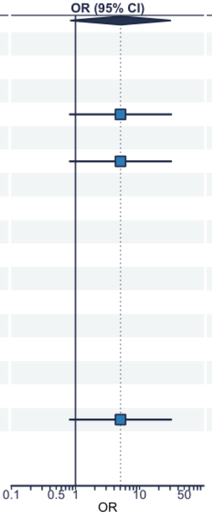

Prespecified route	k	OR	95% CI	I²	Status
Parent model	5	5.04	[0.82; 30.86]	86.6%	Parent
Analytic n ≥200	1	—	—	—	Not pooled
Baseline-adjusted only	2	—	—	—	Not pooled
Core biopsy only	1	—	—	—	Not pooled
Exclude national registries	5	5.04	[0.82; 30.86]	86.6%	Executed
Exclude uncorrected clustering	2	—	—	—	Not pooled
Exclude uncorrected paired kidneys	5	5.04	[0.82; 30.86]	86.6%	Executed
Expert/central reading only	0	—	—	—	Not pooled
Explicit DCD layer	0	—	—	—	Not pooled
Explicit ECD layer	0	—	—	—	Not pooled
Explicit KDPI >=85% layer	0	—	—	—	Not pooled
Frozen processing only	0	—	—	—	Not pooled
Local/on-call reading only	1	—	—	—	Not pooled
Paraffin processing only	2	—	—	—	Not pooled
Post-Banff-2017 era only	1	—	—	—	Not pooled
Pre-Banff-2017 era only	1	—	—	—	Not pooled
QUIPS low or moderate only	1	—	—	—	Not pooled
Strict 7-day dialysis DGF	5	5.04	[0.82; 30.86]	86.6%	Executed
Wedge biopsy only	1	—	—	—	Not pooled

3/18 executed; REML/H-K for k≥3.

S4.2. Glomerulosclerosis and delayed graft function

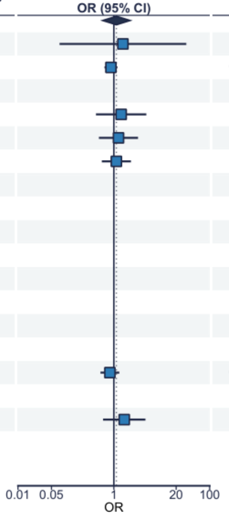

Prespecified route	k	OR	95% CI	I²	Status
Parent model	8	1.13	[0.59; 2.17]	74.0%	Parent
Analytic n ≥200	3	1.55	[0.08; 30.97]	97.8%	Executed
Baseline-adjusted only	3	0.87	[0.67; 1.13]	0.0%	Executed
Core biopsy only	2	—	—	—	Not pooled
Exclude national registries	6	1.42	[0.45; 4.54]	57.6%	Executed
Exclude uncorrected clustering	7	1.25	[0.51; 3.05]	65.1%	Executed
Exclude uncorrected paired kidneys	8	1.13	[0.59; 2.17]	74.0%	Executed
Expert/central reading only	2	—	—	—	Not pooled
Explicit DCD layer	0	—	—	—	Not pooled
Explicit ECD layer	0	—	—	—	Not pooled
Explicit KDPI >=85% layer	1	—	—	—	Not pooled
Frozen processing only	0	—	—	—	Not pooled
Local/on-call reading only	1	—	—	—	Not pooled
Paraffin processing only	2	—	—	—	Not pooled
Post-Banff-2017 era only	1	—	—	—	Not pooled
Pre-Banff-2017 era only	4	0.83	[0.55; 1.24]	0.0%	Executed
QUIPS low or moderate only	1	—	—	—	Not pooled
Strict 7-day dialysis DGF	5	1.65	[0.63; 4.34]	66.5%	Executed
Wedge biopsy only	1	—	—	—	Not pooled

7/18 executed; REML/H-K for k≥3.

S4.3. IFTA and delayed graft function

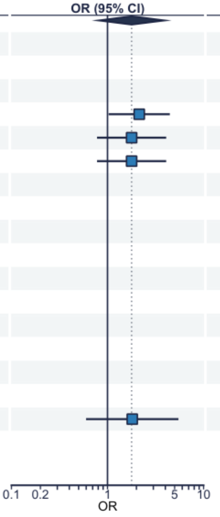

Prespecified route	k	OR	95% CI	I²	Status
Parent model	6	1.78	[0.80; 3.98]	71.6%	Parent
Analytic n ≥200	2	—	—	—	Not pooled
Baseline-adjusted only	2	—	—	—	Not pooled
Core biopsy only	2	—	—	—	Not pooled
Exclude national registries	5	2.14	[1.05; 4.35]	0.0%	Executed
Exclude uncorrected clustering	6	1.78	[0.80; 3.98]	71.6%	Executed
Exclude uncorrected paired kidneys	6	1.78	[0.80; 3.98]	71.6%	Executed
Expert/central reading only	2	—	—	—	Not pooled
Explicit DCD layer	0	—	—	—	Not pooled
Explicit ECD layer	0	—	—	—	Not pooled
Explicit KDPI >=85% layer	0	—	—	—	Not pooled
Frozen processing only	1	—	—	—	Not pooled
Local/on-call reading only	1	—	—	—	Not pooled
Paraffin processing only	2	—	—	—	Not pooled
Post-Banff-2017 era only	1	—	—	—	Not pooled
Pre-Banff-2017 era only	2	—	—	—	Not pooled
QUIPS low or moderate only	1	—	—	—	Not pooled
Strict 7-day dialysis DGF	5	1.81	[0.61; 5.34]	75.6%	Executed
Wedge biopsy only	1	—	—	—	Not pooled

4/18 executed; REML/H-K for k≥3.

S4.4. Vascular injury and delayed graft function

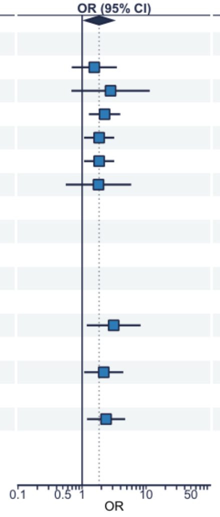

Prespecified route	k	OR	95% CI	I²	Status
Parent model	7	1.85	[1.11; 3.06]	68.4%	Parent
Analytic n ≥200	2	—	—	—	Not pooled
Baseline-adjusted only	4	1.55	[0.71; 3.37]	60.9%	Executed
Core biopsy only	3	2.79	[0.71; 10.98]	51.2%	Executed
Exclude national registries	6	2.25	[1.32; 3.80]	36.5%	Executed
Exclude uncorrected clustering	7	1.85	[1.11; 3.06]	68.4%	Executed
Exclude uncorrected paired kidneys	7	1.85	[1.11; 3.06]	68.4%	Executed
Expert/central reading only	3	1.80	[0.58; 5.64]	0.0%	Executed
Explicit DCD layer	0	—	—	—	Not pooled
Explicit ECD layer	0	—	—	—	Not pooled
Explicit KDPI >=85% layer	0	—	—	—	Not pooled
Frozen processing only	1	—	—	—	Not pooled
Local/on-call reading only	1	—	—	—	Not pooled
Paraffin processing only	3	3.12	[1.23; 7.91]	18.7%	Executed
Post-Banff-2017 era only	1	—	—	—	Not pooled
Pre-Banff-2017 era only	3	2.18	[1.12; 4.24]	0.0%	Executed
QUIPS low or moderate only	2	—	—	—	Not pooled
Strict 7-day dialysis DGF	5	2.37	[1.24; 4.54]	40.8%	Executed
Wedge biopsy only	1	—	—	—	Not pooled

9/18 executed; REML/H-K for k≥3.

Abbreviations and symbols: CI, confidence interval; DCD, donation after circulatory death; DGF, delayed graft function; ECD, expanded-criteria donor; H-K, Hartung–Knapp inference; I², percentage of total variability attributable to between-study heterogeneity; IFTA, interstitial fibrosis and tubular atrophy; k, number of cohorts in the analytic cell; KDPI, Kidney Donor Profile Index; n, reported sample size; OR, odds ratio; QUIPS, Quality In Prognosis Studies; REML, restricted maximum likelihood. Symbols mark estimates and horizontal lines their confidence intervals; Parent denotes the full pooled analysis.

Supplementary Figure S4. Prespecified sensitivity analyses (continued)

S4.5. Glomerulosclerosis and graft failure

Prespecified route	k	HR	95% CI	I²	Status
Parent model	9	2.03	[1.22; 3.39]	86.0%	Parent
Analytic n ≥200	6	2.09	[0.94; 4.66]	93.1%	Executed
Baseline-adjusted only	4	1.48	[0.69; 3.17]	82.4%	Executed
Core biopsy only	1	—	—	—	Not pooled
Exclude national registries	7	2.55	[1.42; 4.60]	64.0%	Executed
Exclude uncorrected clustering	7	2.29	[1.10; 4.76]	82.6%	Executed
Exclude uncorrected paired kidneys	9	2.03	[1.22; 3.39]	86.0%	Executed
Expert/central reading only	5	1.90	[1.57; 2.30]	0.0%	Executed
Explicit DCD layer	0	—	—	—	Not pooled
Explicit ECD layer	1	—	—	—	Not pooled
Explicit KDPI >=85% layer	1	—	—	—	Not pooled
Frozen processing only	2	—	—	—	Not pooled
Local/on-call reading only	0	—	—	—	Not pooled
Paraffin processing only	2	—	—	—	Not pooled
Post-Banff-2017 era only	0	—	—	—	Not pooled
Pre-Banff-2017 era only	5	2.27	[0.73; 7.10]	89.0%	Executed
QUIPS low or moderate only	1	—	—	—	Not pooled
Strict 7-day dialysis DGF	0	—	—	—	Not applicable
Wedge biopsy only	5	2.94	[1.18; 7.34]	65.8%	Executed

8/18 executed; REML/H-K for k≥3.

S4.6. IFTA and graft failure

Prespecified route	k	HR	95% CI	I²	Status
Parent model	6	1.64	[0.96; 2.82]	50.9%	Parent
Analytic n ≥200	5	1.77	[1.03; 3.04]	49.9%	Executed
Baseline-adjusted only	2	—	—	—	Not pooled
Core biopsy only	1	—	—	—	Not pooled
Exclude national registries	5	1.76	[0.80; 3.88]	59.9%	Executed
Exclude uncorrected clustering	4	1.83	[0.53; 6.37]	67.1%	Executed
Exclude uncorrected paired kidneys	6	1.64	[0.96; 2.82]	50.9%	Executed
Expert/central reading only	4	1.52	[0.82; 2.82]	0.0%	Executed
Explicit DCD layer	0	—	—	—	Not pooled
Explicit ECD layer	1	—	—	—	Not pooled
Explicit KDPI >=85% layer	0	—	—	—	Not pooled
Frozen processing only	1	—	—	—	Not pooled
Local/on-call reading only	0	—	—	—	Not pooled
Paraffin processing only	2	—	—	—	Not pooled
Post-Banff-2017 era only	0	—	—	—	Not pooled
Pre-Banff-2017 era only	3	2.02	[0.20; 20.80]	75.7%	Executed
QUIPS low or moderate only	1	—	—	—	Not pooled
Strict 7-day dialysis DGF	0	—	—	—	Not applicable
Wedge biopsy only	4	1.83	[0.53; 6.37]	67.1%	Executed

7/18 executed; REML/H-K for k≥3.

S4.7. Vascular injury and graft failure

Prespecified route	k	HR	95% CI	I²	Status
Parent model	9	1.48	[1.07; 2.04]	53.0%	Parent
Analytic n ≥200	7	1.49	[1.15; 1.94]	48.6%	Executed
Baseline-adjusted only	4	1.42	[0.75; 2.71]	63.4%	Executed
Core biopsy only	1	—	—	—	Not pooled
Exclude national registries	7	1.70	[1.13; 2.54]	22.0%	Executed
Exclude uncorrected clustering	6	1.83	[0.94; 3.57]	43.0%	Executed
Exclude uncorrected paired kidneys	9	1.48	[1.07; 2.04]	53.0%	Executed
Expert/central reading only	5	1.61	[0.71; 3.68]	67.7%	Executed
Explicit DCD layer	0	—	—	—	Not pooled
Explicit ECD layer	2	—	—	—	Not pooled
Explicit KDPI >=85% layer	0	—	—	—	Not pooled
Frozen processing only	1	—	—	—	Not pooled
Local/on-call reading only	0	—	—	—	Not pooled
Paraffin processing only	3	1.56	[1.08; 2.27]	0.0%	Executed
Post-Banff-2017 era only	0	—	—	—	Not pooled
Pre-Banff-2017 era only	5	1.48	[0.72; 3.07]	60.7%	Executed
QUIPS low or moderate only	1	—	—	—	Not pooled
Strict 7-day dialysis DGF	0	—	—	—	Not applicable
Wedge biopsy only	4	1.56	[0.41; 5.91]	72.1%	Executed

9/18 executed; REML/H-K for k≥3.

S4.8. Remuzzi score and delayed graft function

Prespecified route	k	OR	95% CI	I²	Status
Parent model	3	1.09	[0.63; 1.89]	12.8%	Parent
Analytic n ≥200	0	—	—	—	Not pooled
Baseline-adjusted only	1	—	—	—	Not pooled
Core biopsy only	1	—	—	—	Not pooled
Exclude national registries	3	1.09	[0.63; 1.89]	12.8%	Executed
Exclude uncorrected clustering	2	—	—	—	Not pooled
Exclude uncorrected paired kidneys	3	1.09	[0.63; 1.89]	12.8%	Executed
Expert/central reading only	2	—	—	—	Not pooled
Explicit DCD layer	0	—	—	—	Not pooled
Explicit ECD layer	0	—	—	—	Not pooled
Explicit KDPI >=85% layer	0	—	—	—	Not pooled
Frozen processing only	0	—	—	—	Not pooled
Local/on-call reading only	0	—	—	—	Not pooled
Paraffin processing only	0	—	—	—	Not pooled
Post-Banff-2017 era only	0	—	—	—	Not pooled
Pre-Banff-2017 era only	1	—	—	—	Not pooled
QUIPS low or moderate only	0	—	—	—	Not pooled
Strict 7-day dialysis DGF	2	—	—	—	Not pooled
Wedge biopsy only	1	—	—	—	Not pooled

2/18 executed; REML/H-K for k≥3.

Abbreviations and symbols: CI, confidence interval; DCD, donation after circulatory death; DGF, delayed graft function; ECD, expanded-criteria donor; H-K, Hartung–Knapp inference; HR, hazard ratio; I², percentage of total variability attributable to between-study heterogeneity; IFTA, interstitial fibrosis and tubular atrophy; k, number of cohorts in the analytic cell; KDPI, Kidney Donor Profile Index; n, reported sample size; OR, odds ratio; QUIPS, Quality In Prognosis Studies; REML, restricted maximum likelihood. Symbols mark estimates and horizontal lines their confidence intervals; Parent denotes the full pooled analysis.

Supplementary Figure S4. Prespecified sensitivity analyses (continued)

S4.9. Vascular injury per grade and delayed graft function

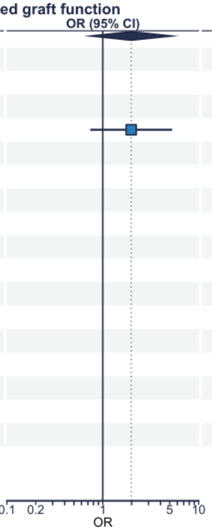

Prespecified route	k	OR	95% CI	I²	Status
Parent model	3	1.98	[0.76; 5.17]	0.0%	Parent
Analytic n ≥200	1	—	—	—	Not pooled
Baseline-adjusted only	2	—	—	—	Not pooled
Core biopsy only	1	—	—	—	Not pooled
Exclude national registries	3	1.98	[0.76; 5.17]	0.0%	Executed
Exclude uncorrected clustering	0	—	—	—	Not pooled
Exclude uncorrected paired kidneys	1	—	—	—	Not pooled
Expert/central reading only	1	—	—	—	Not pooled
Explicit DCD layer	1	—	—	—	Not pooled
Explicit ECD layer	0	—	—	—	Not pooled
Explicit KDPI >=85% layer	0	—	—	—	Not pooled
Frozen processing only	0	—	—	—	Not pooled
Local/on-call reading only	0	—	—	—	Not pooled
Paraffin processing only	2	—	—	—	Not pooled
Post-Banff-2017 era only	1	—	—	—	Not pooled
Pre-Banff-2017 era only	0	—	—	—	Not pooled
QUIPS low or moderate only	0	—	—	—	Not pooled
Strict 7-day dialysis DGF	2	—	—	—	Not pooled
Wedge biopsy only	1	—	—	—	Not pooled

1/18 executed; REML/H-K for k≥3.

S4.10. IFTA and low eGFR at 9–15 months

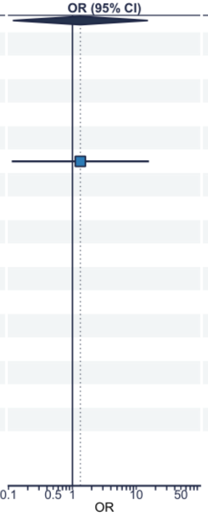

Prespecified route	k	OR	95% CI	I²	Status
Parent model	3	1.33	[0.12; 15.20]	86.5%	Parent
Analytic n ≥200	2	—	—	—	Not pooled
Baseline-adjusted only	2	—	—	—	Not pooled
Core biopsy only	0	—	—	—	Not pooled
Exclude national registries	2	—	—	—	Not pooled
Exclude uncorrected clustering	1	—	—	—	Not pooled
Exclude uncorrected paired kidneys	3	1.33	[0.12; 15.20]	86.5%	Executed
Expert/central reading only	1	—	—	—	Not pooled
Explicit DCD layer	0	—	—	—	Not pooled
Explicit ECD layer	0	—	—	—	Not pooled
Explicit KDPI >=85% layer	0	—	—	—	Not pooled
Frozen processing only	0	—	—	—	Not pooled
Local/on-call reading only	0	—	—	—	Not pooled
Paraffin processing only	1	—	—	—	Not pooled
Post-Banff-2017 era only	0	—	—	—	Not pooled
Pre-Banff-2017 era only	0	—	—	—	Not pooled
QUIPS low or moderate only	1	—	—	—	Not pooled
Strict 7-day dialysis DGF	0	—	—	—	Not applicable
Wedge biopsy only	0	—	—	—	Not pooled

1/18 executed; REML/H-K for k≥3.

S4.11. Vascular injury and low eGFR at 9–15 months

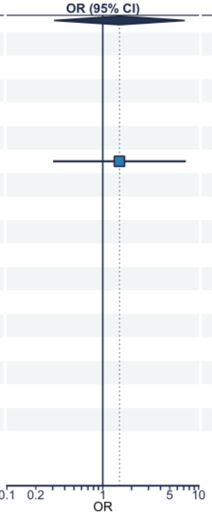

Prespecified route	k	OR	95% CI	I²	Status
Parent model	3	1.49	[0.31; 7.18]	63.9%	Parent
Analytic n ≥200	2	—	—	—	Not pooled
Baseline-adjusted only	2	—	—	—	Not pooled
Core biopsy only	0	—	—	—	Not pooled
Exclude national registries	2	—	—	—	Not pooled
Exclude uncorrected clustering	1	—	—	—	Not pooled
Exclude uncorrected paired kidneys	3	1.49	[0.31; 7.18]	63.9%	Executed
Expert/central reading only	1	—	—	—	Not pooled
Explicit DCD layer	0	—	—	—	Not pooled
Explicit ECD layer	0	—	—	—	Not pooled
Explicit KDPI >=85% layer	0	—	—	—	Not pooled
Frozen processing only	0	—	—	—	Not pooled
Local/on-call reading only	0	—	—	—	Not pooled
Paraffin processing only	1	—	—	—	Not pooled
Post-Banff-2017 era only	0	—	—	—	Not pooled
Pre-Banff-2017 era only	0	—	—	—	Not pooled
QUIPS low or moderate only	1	—	—	—	Not pooled
Strict 7-day dialysis DGF	0	—	—	—	Not applicable
Wedge biopsy only	0	—	—	—	Not pooled

1/18 executed; REML/H-K for k≥3.

S4.12. Tubular atrophy and graft failure

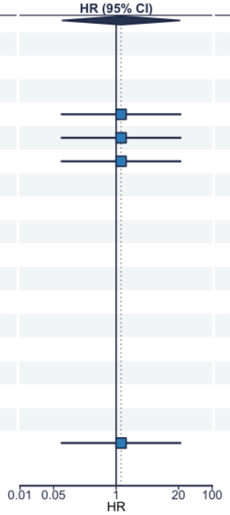

Prespecified route	k	HR	95% CI	I²	Status
Parent model	3	1.27	[0.07; 21.69]	83.2%	Parent
Analytic n ≥200	2	—	—	—	Not pooled
Baseline-adjusted only	0	—	—	—	Not pooled
Core biopsy only	0	—	—	—	Not pooled
Exclude national registries	3	1.27	[0.07; 21.69]	83.2%	Executed
Exclude uncorrected clustering	3	1.27	[0.07; 21.69]	83.2%	Executed
Exclude uncorrected paired kidneys	3	1.27	[0.07; 21.69]	83.2%	Executed
Expert/central reading only	2	—	—	—	Not pooled
Explicit DCD layer	0	—	—	—	Not pooled
Explicit ECD layer	1	—	—	—	Not pooled
Explicit KDPI >=85% layer	0	—	—	—	Not pooled
Frozen processing only	1	—	—	—	Not pooled
Local/on-call reading only	0	—	—	—	Not pooled
Paraffin processing only	1	—	—	—	Not pooled
Post-Banff-2017 era only	0	—	—	—	Not pooled
Pre-Banff-2017 era only	2	—	—	—	Not pooled
QUIPS low or moderate only	0	—	—	—	Not pooled
Strict 7-day dialysis DGF	0	—	—	—	Not applicable
Wedge biopsy only	3	1.27	[0.07; 21.69]	83.2%	Executed

4/18 executed; REML/H-K for k≥3.

Abbreviations and symbols: CI, confidence interval; DCD, donation after circulatory death; DGF, delayed graft function; ECD, expanded-criteria donor; eGFR, estimated glomerular filtration rate; H-K, Hartung–Knapp inference; HR, hazard ratio; I², percentage of total variability attributable to between-study heterogeneity; IFTA, interstitial fibrosis and tubular atrophy; k, number of cohorts in the analytic cell; KDPI, Kidney Donor Profile Index; n, reported sample size; OR, odds ratio; QUIPS, Quality In Prognosis Studies; REML, restricted maximum likelihood. Symbols mark estimates and horizontal lines their confidence intervals; Parent denotes the full pooled analysis.

Supplementary Figure S5. Adjustment-stratified analyses

S5.1. Acute tubular injury and delayed graft function

Analysis	k	OR (95% CI)	OR	95% CI	I²	Status
**Parent model**	5		**5.04**	**[0.82; 30.86]**	**86.6%**	**Parent**
Baseline-adjusted subgroup	2		—	—	—	Not pooled
Crude/recalculated subgroup	3		4.34	[0.03; 743.78]	90.0%	Executed

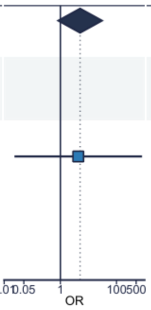

Subgroups; no moderation inference.

S5.2. Glomerulosclerosis and delayed graft function

Analysis	k	OR (95% CI)	OR	95% CI	I²	Status
**Parent model**	8		**1.13**	**[0.59; 2.17]**	**74.0%**	**Parent**
Baseline-adjusted subgroup	3		0.87	[0.67; 1.13]	0.0%	Executed
Crude/recalculated subgroup	5		1.33	[0.28; 6.23]	69.0%	Executed

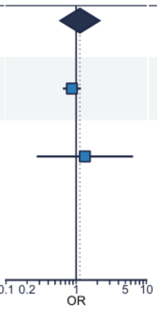

Subgroups; no moderation inference.

S5.3. IFTA and delayed graft function

Analysis	k	OR (95% CI)	OR	95% CI	I²	Status
**Parent model**	6		**1.78**	**[0.80; 3.98]**	**71.6%**	**Parent**
Baseline-adjusted subgroup	2		—	—	—	Not pooled
Crude/recalculated subgroup	4		2.06	[0.77; 5.49]	6.8%	Executed

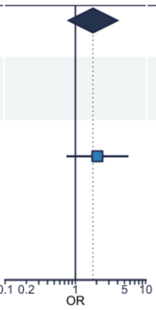

Subgroups; no moderation inference.

S5.4. Vascular injury and delayed graft function

Analysis	k	OR (95% CI)	OR	95% CI	I²	Status
**Parent model**	7		**1.85**	**[1.11; 3.06]**	**68.4%**	**Parent**
Baseline-adjusted subgroup	4		1.55	[0.71; 3.37]	60.9%	Executed
Crude/recalculated subgroup	3		2.31	[0.47; 11.37]	62.4%	Executed

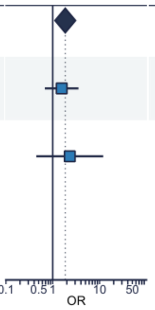

Subgroups; no moderation inference.

S5.5. Glomerulosclerosis and graft failure

Analysis	k	HR (95% CI)	HR	95% CI	I²	Status
**Parent model**	9		**2.03**	**[1.22; 3.39]**	**86.0%**	**Parent**
Baseline-adjusted subgroup	4		1.48	[0.69; 3.17]	82.4%	Executed
Crude/recalculated subgroup	5		2.61	[1.02; 6.64]	78.6%	Executed

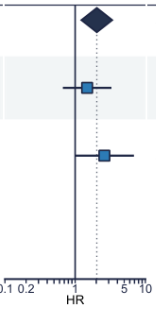

Subgroups; no moderation inference.

S5.6. IFTA and graft failure

Analysis	k	HR (95% CI)	HR	95% CI	I²	Status
**Parent model**	6		**1.64**	**[0.96; 2.82]**	**50.9%**	**Parent**
Baseline-adjusted subgroup	2		—	—	—	Not pooled
Crude/recalculated subgroup	4		1.83	[0.53; 6.37]	67.1%	Executed

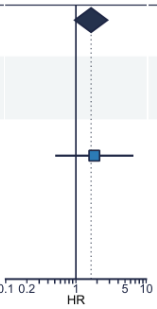

Subgroups; no moderation inference.

S5.7. Vascular injury and graft failure

Analysis	k	HR (95% CI)	HR	95% CI	I²	Status
**Parent model**	9		**1.48**	**[1.07; 2.04]**	**53.0%**	**Parent**
Baseline-adjusted subgroup	4		1.42	[0.75; 2.71]	63.4%	Executed
Crude/recalculated subgroup	5		1.57	[0.97; 2.54]	12.3%	Executed

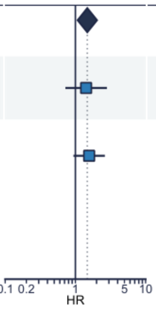

Subgroups; no moderation inference.

S5.8. Remuzzi score and delayed graft function

Analysis	k	OR (95% CI)	OR	95% CI	I²	Status
**Parent model**	3		**1.09**	**[0.63; 1.89]**	**12.8%**	**Parent**
Baseline-adjusted subgroup	1		—	—	—	Not pooled
Crude/recalculated subgroup	2		—	—	—	Not pooled

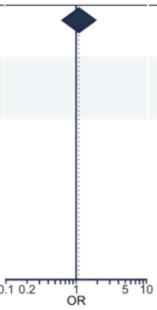

Subgroups; no moderation inference.

Abbreviations and symbols: CI, confidence interval; HR, hazard ratio; I², percentage of total variability attributable to between-study heterogeneity; IFTA, interstitial fibrosis and tubular atrophy; k, number of cohorts in the analytic cell; OR, odds ratio. Symbols mark estimates and horizontal lines their confidence intervals; Parent denotes the full pooled analysis.

Supplementary Figure S5. Adjustment-stratified analyses (continued)

S5.9. Vascular injury per grade and delayed graft function

Analysis	k	OR (95% CI)	OR	95% CI	I²	Status
**Parent model**	3		**1.98**	**[0.76; 5.17]**	**0.0%**	**Parent**
Baseline-adjusted subgroup	2		—	—	—	Not pooled
Crude/recalculated subgroup	1		—	—	—	Not pooled

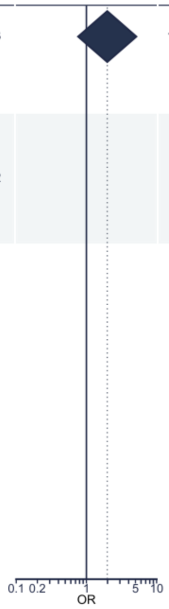

Subgroups; no moderation inference.

S5.10. IFTA and low eGFR at 9–15 months

Analysis	k	OR (95% CI)	OR	95% CI	I²	Status
**Parent model**	3		**1.33**	**[0.12; 15.20]**	**86.5%**	**Parent**
Baseline-adjusted subgroup	2		—	—	—	Not pooled
Crude/recalculated subgroup	1		—	—	—	Not pooled

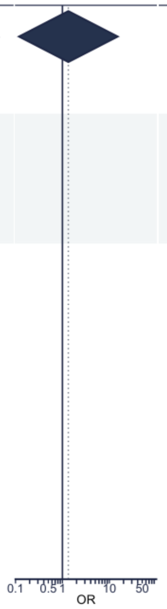

Subgroups; no moderation inference.

S5.11. Vascular injury and low eGFR at 9–15 months

Analysis	k	OR (95% CI)	OR	95% CI	I²	Status
**Parent model**	3		**1.49**	**[0.31; 7.18]**	**63.9%**	**Parent**
Baseline-adjusted subgroup	2		—	—	—	Not pooled
Crude/recalculated subgroup	1		—	—	—	Not pooled

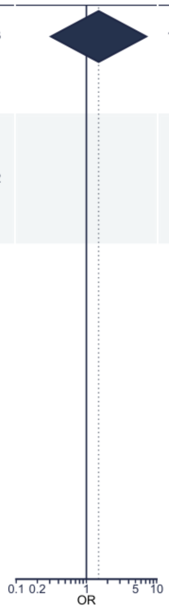

Subgroups; no moderation inference.

S5.12. Tubular atrophy and graft failure

Analysis	k	HR (95% CI)	HR	95% CI	I²	Status
**Parent model**	3		**1.27**	**[0.07; 21.69]**	**83.2%**	**Parent**
Baseline-adjusted subgroup	0		—	—	—	Not pooled
Crude/recalculated subgroup	3		1.27	[0.07; 21.69]	83.2%	Executed

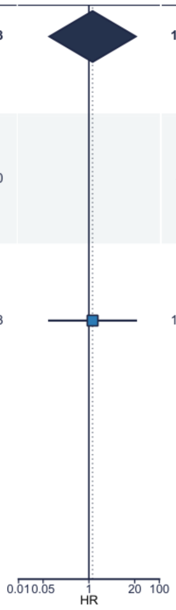

Subgroups; no moderation inference.

Abbreviations and symbols: CI, confidence interval; eGFR, estimated glomerular filtration rate; HR, hazard ratio; I², percentage of total variability attributable to between-study heterogeneity; IFTA, interstitial fibrosis and tubular atrophy; k, number of cohorts in the analytic cell; OR, odds ratio. Symbols mark estimates and horizontal lines their confidence intervals; Parent denotes the full pooled analysis.

Supplementary Figure S6. Complete leave-one-cohort-out analyses

S6.1. Acute tubular injury and delayed graft function

Cohort omitted	k	OR (95% CI)	OR	95% CI	I²	Status
**Parent model**	**5**		**5.04**	**[0.82; 30.86]**	**86.6%**	**Parent**
Omit Li, 2024	4		4.45	[0.31; 64.39]	86.7%	Executed
Omit Matignon, 2008	4		8.03	[1.19; 54.12]	69.9%	Executed
Omit Mourão, 2016	4		6.16	[0.49; 77.30]	90.4%	Executed
Omit Rohr, 1983	4		3.14	[0.62; 15.79]	79.5%	Executed
Omit Wang, 2022	4		5.08	[0.34; 75.67]	90.4%	Executed

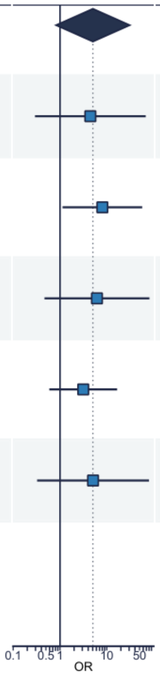

LOO; k=2 rows are not pooled.

S6.2. Glomerulosclerosis and delayed graft function

Cohort omitted	k	OR (95% CI)	OR	95% CI	I²	Status
**Parent model**	**8**		**1.13**	**[0.59; 2.17]**	**74.0%**	**Parent**
Omit Cheungpasitporn, 2020	7		1.28	[0.54; 3.04]	62.4%	Executed
Omit Lee, 2026	7		1.25	[0.51; 3.05]	65.1%	Executed
Omit Li, 2024	7		0.88	[0.73; 1.06]	0.0%	Executed
Omit Lu, 2000	7		1.24	[0.61; 2.49]	78.6%	Executed
Omit Lubuska, 2006	7		1.21	[0.60; 2.45]	79.3%	Executed
Omit Matignon, 2008	7		1.11	[0.48; 2.53]	83.3%	Executed
Omit Teixeira, 2021	7		1.11	[0.51; 2.41]	80.7%	Executed
Omit Wang, 2022	7		1.13	[0.48; 2.64]	84.8%	Executed

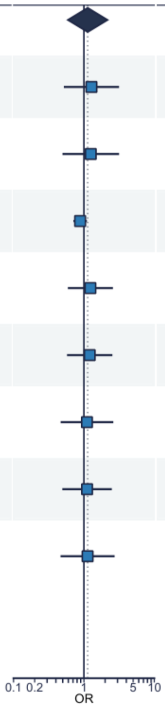

LOO; k=2 rows are not pooled.

S6.3. IFTA and delayed graft function

Cohort omitted	k	OR (95% CI)	OR	95% CI	I²	Status
**Parent model**	**6**		**1.78**	**[0.80; 3.98]**	**71.6%**	**Parent**
Omit Lee, 2026	5		2.14	[1.05; 4.35]	0.0%	Executed
Omit Li, 2024	5		1.37	[0.72; 2.62]	58.1%	Executed
Omit Lubuska, 2006	5		1.81	[0.61; 5.34]	75.6%	Executed
Omit Matignon, 2008	5		1.92	[0.65; 5.67]	75.5%	Executed
Omit Teixeira, 2021	5		1.66	[0.62; 4.47]	75.2%	Executed
Omit Wang, 2022	5		1.91	[0.68; 5.41]	78.5%	Executed

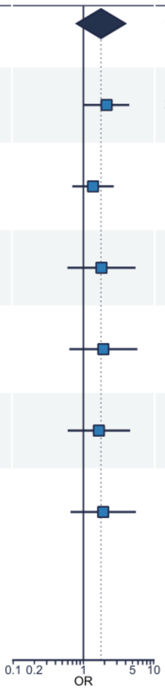

LOO; k=2 rows are not pooled.

S6.4. Vascular injury and delayed graft function

Cohort omitted	k	OR (95% CI)	OR	95% CI	I²	Status
**Parent model**	**7**		**1.85**	**[1.11; 3.06]**	**68.4%**	**Parent**
Omit Lee, 2016	6		1.74	[0.97; 3.13]	70.9%	Executed
Omit Lee, 2026	6		2.25	[1.32; 3.80]	36.5%	Executed
Omit Li, 2024	6		1.49	[0.98; 2.26]	44.7%	Executed
Omit Lubuska, 2006	6		1.90	[1.03; 3.49]	73.2%	Executed
Omit Matignon, 2008	6		1.80	[0.97; 3.34]	71.6%	Executed
Omit Teixeira, 2021	6		1.95	[1.09; 3.47]	72.5%	Executed
Omit Wang, 2022	6		1.92	[1.04; 3.52]	72.8%	Executed

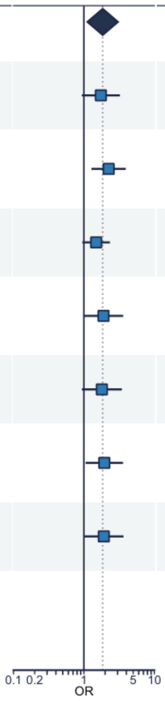

LOO; k=2 rows are not pooled.

Abbreviations and symbols: CI, confidence interval; I², percentage of total variability attributable to between-study heterogeneity; IFTA, interstitial fibrosis and tubular atrophy; k, number of cohorts in the analytic cell; LOO, leave-one-cohort-out analysis; OR, odds ratio. Symbols mark estimates and horizontal lines their confidence intervals; Parent denotes the full pooled analysis.

Supplementary Figure S6. Complete leave-one-cohort-out analyses (continued)

S6.5. Glomerulosclerosis and graft failure

Cohort omitted	k	HR (95% CI)	HR	95% CI	I²	Status
**Parent model**	**9**		**2.03**	**[1.22; 3.39]**	**86.0%**	**Parent**
Omit Chen, 2021	8		2.11	[1.18; 3.77]	88.8%	Executed
Omit Cheungpasitporn, 2020	8		2.29	[1.39; 3.76]	70.5%	Executed
Omit Lee, 2026	8		2.17	[1.19; 3.96]	85.4%	Executed
Omit Lin, 2010	8		1.98	[1.11; 3.54]	88.4%	Executed
Omit Munivenkatappa, 2008	8		2.10	[1.15; 3.85]	88.7%	Executed
Omit Pegas, 2018	8		1.63	[1.19; 2.22]	68.6%	Executed
Omit Reese, 2021	8		2.12	[1.15; 3.89]	86.6%	Executed
Omit Teixeira, 2021	8		2.05	[1.16; 3.62]	88.1%	Executed
Omit Villanego, 2022	8		1.97	[1.10; 3.53]	88.3%	Executed

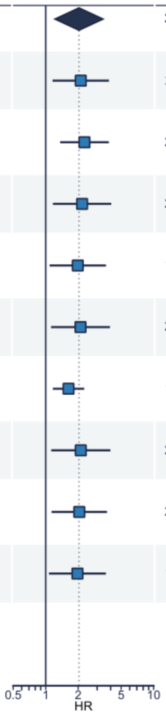

LOO; k=2 rows are not pooled.

S6.6. IFTA and graft failure

Cohort omitted	k	HR (95% CI)	HR	95% CI	I²	Status
**Parent model**	**6**		**1.64**	**[0.96; 2.82]**	**50.9%**	**Parent**
Omit Chen, 2021	5		1.77	[1.03; 3.04]	49.9%	Executed
Omit Lee, 2026	5		1.76	[0.80; 3.88]	59.9%	Executed
Omit Munivenkatappa, 2008	5		1.52	[0.81; 2.86]	46.7%	Executed
Omit Pegas, 2018	5		1.45	[1.03; 2.03]	0.0%	Executed
Omit Reese, 2021	5		1.70	[0.76; 3.80]	64.8%	Executed
Omit Villanego, 2022	5		1.73	[0.83; 3.60]	67.4%	Executed

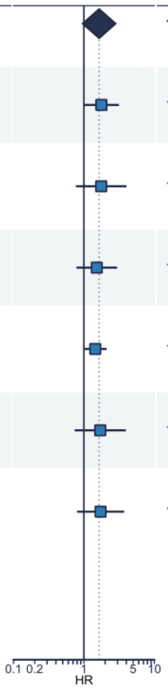

LOO; k=2 rows are not pooled.

S6.7. Vascular injury and graft failure

Cohort omitted	k	HR (95% CI)	HR	95% CI	I²	Status
**Parent model**	**9**		**1.48**	**[1.07; 2.04]**	**53.0%**	**Parent**
Omit Chen, 2021	8		1.54	[1.19; 2.00]	50.8%	Executed
Omit Lee, 2026	8		1.55	[1.14; 2.11]	26.4%	Executed
Omit Munivenkatappa, 2008	8		1.40	[1.00; 1.97]	45.8%	Executed
Omit Pegas, 2018	8		1.44	[1.03; 2.03]	54.1%	Executed
Omit Reese, 2021	8		1.52	[0.98; 2.35]	63.0%	Executed
Omit Stewart, 2022	8		1.56	[1.02; 2.39]	61.0%	Executed
Omit Teixeira, 2021	8		1.43	[1.03; 1.98]	51.0%	Executed
Omit Villanego, 2022	8		1.42	[1.02; 1.97]	48.5%	Executed
Omit Wazna, 2018	8		1.44	[1.00; 2.08]	56.8%	Executed

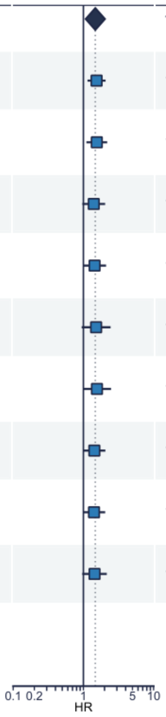

LOO; k=2 rows are not pooled.

S6.8. Remuzzi score and delayed graft function

Cohort omitted	k	OR (95% CI)	OR	95% CI	I²	Status
**Parent model**	**3**		**1.09**	**[0.63; 1.89]**	**12.8%**	**Parent**
Omit Chen, 2021	2		—	—	—	Not pooled
Omit Pavlovic, 2022	2		—	—	—	Not pooled
Omit Tan, 2024	2		—	—	—	Not pooled

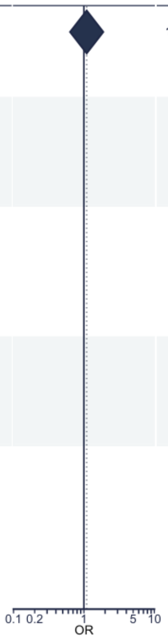

LOO; k=2 rows are not pooled.

Abbreviations and symbols: CI, confidence interval; HR, hazard ratio; I², percentage of total variability attributable to between-study heterogeneity; IFTA, interstitial fibrosis and tubular atrophy; k, number of cohorts in the analytic cell; LOO, leave-one-cohort-out analysis; OR, odds ratio. Symbols mark estimates and horizontal lines their confidence intervals; Parent denotes the full pooled analysis.

Supplementary Figure S6. Complete leave-one-cohort-out analyses (continued)

S6.9. Vascular injury per grade and delayed graft function

Cohort omitted	k	OR (95% CI)	OR	95% CI	I²	Status
**Parent model**	**3**		**1.98**	**[0.76; 5.17]**	**0.0%**	**Parent**
Omit Di Paolo, 2002	2		—	—	—	Not pooled
Omit Perez-Gutierrez, 2021	2		—	—	—	Not pooled
Omit Zheng, 2020	2		—	—	—	Not pooled

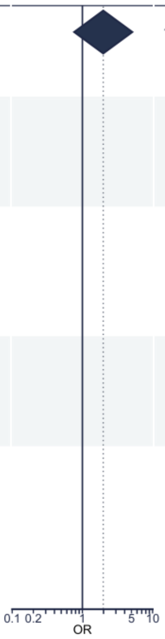

LOO; k=2 rows are not pooled.

S6.10. IFTA and low eGFR at 9–15 months

Cohort omitted	k	OR (95% CI)	OR	95% CI	I²	Status
**Parent model**	**3**		**1.33**	**[0.12; 15.20]**	**86.5%**	**Parent**
Omit Lee, 2026	2		—	—	—	Not pooled
Omit Proença, 2025	2		—	—	—	Not pooled
Omit Teixeira, 2021	2		—	—	—	Not pooled

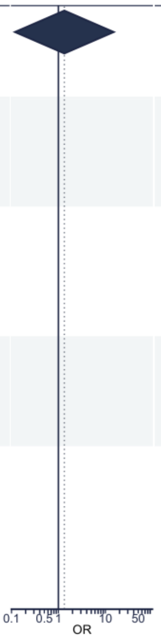

LOO; k=2 rows are not pooled.

S6.11. Vascular injury and low eGFR at 9–15 months

Cohort omitted	k	OR (95% CI)	OR	95% CI	I²	Status
**Parent model**	**3**		**1.49**	**[0.31; 7.18]**	**63.9%**	**Parent**
Omit Lee, 2026	2		—	—	—	Not pooled
Omit Proença, 2025	2		—	—	—	Not pooled
Omit Teixeira, 2021	2		—	—	—	Not pooled

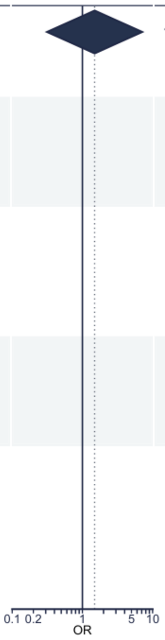

LOO; k=2 rows are not pooled.

S6.12. Tubular atrophy and graft failure

Cohort omitted	k	HR (95% CI)	HR	95% CI	I²	Status
**Parent model**	**3**		**1.27**	**[0.07; 21.69]**	**83.2%**	**Parent**
Omit Chen, 2021	2		—	—	—	Not pooled
Omit Pegas, 2018	2		—	—	—	Not pooled
Omit Villanego, 2022	2		—	—	—	Not pooled

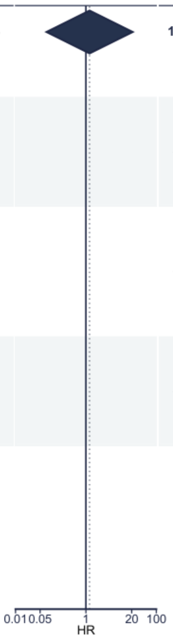

LOO; k=2 rows are not pooled.

Abbreviations and symbols: CI, confidence interval; eGFR, estimated glomerular filtration rate; HR, hazard ratio; I², percentage of total variability attributable to between-study heterogeneity; IFTA, interstitial fibrosis and tubular atrophy; k, number of cohorts in the analytic cell; LOO, leave-one-cohort-out analysis; OR, odds ratio. Symbols mark estimates and horizontal lines their confidence intervals; Parent denotes the full pooled analysis.

Supplementary Figure S7. Funnel-plot eligibility audit

All 12 meta-analyses are below k=10; no funnel plot or asymmetry inference was generated.

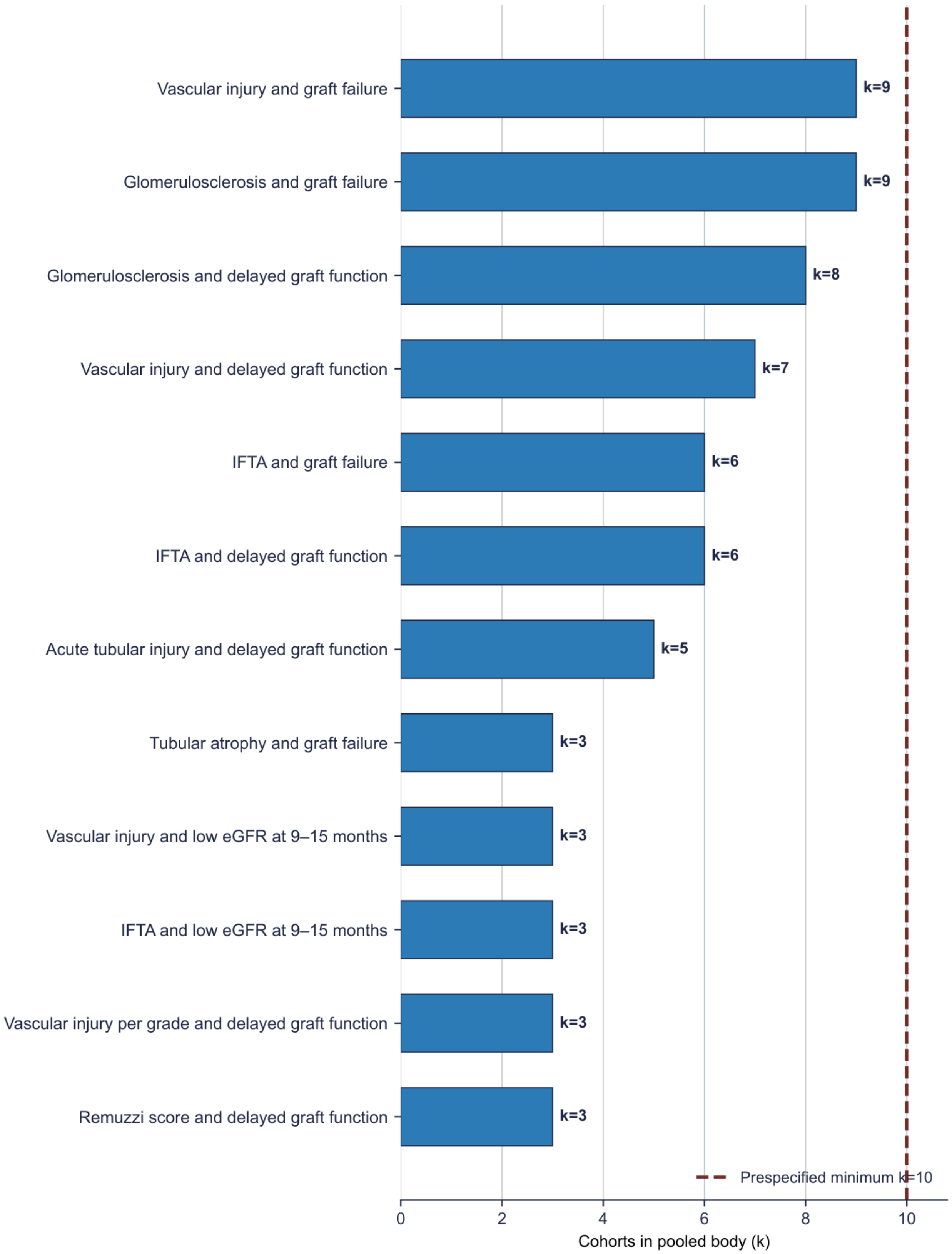

Publication bias was not statistically assessable; this does not indicate that it was absent.

Abbreviations and symbols: eGFR, estimated glomerular filtration rate; IFTA, interstitial fibrosis and tubular atrophy; k, number of cohorts in the analytic cell.

PRISMA 2020

Supplementary reporting checklists

Time-Zero Kidney Biopsy and Deceased-Donor Transplant Outcomes: A Systematic Review and Meta-Analysis of Prognostic Value, Prediction, and Clinical Utility

These six completed instruments follow the 59-page scientific tables-and-figures section of this Supplementary Material. Locations refer to the rendered 35-page main manuscript and to Supplementary Tables S1a–S1c and S2–S14 and Figures S1–S7 on the preceding pages. AMSTAR-PF is presented as a self-audit, not an external endorsement.

Item	Checklist requirement	Where reported	Status / clarification
1	Identify the report as a systematic review.	Main p. 1	Reported
2	Report the PRISMA 2020 for Abstracts items.	Main p. 1	Reported
3	Describe the rationale in the context of existing knowledge.	Main pp. 2–3	Reported
4	Provide an explicit objective or review question.	Main pp. 2–3	Reported
5	Specify inclusion/exclusion criteria and grouping for syntheses.	Main pp. 3–4	Reported
6	Specify all information sources and the last search date for each.	Main pp. 3–4; Supplement pp. 2–10 (S1a–S1c)	
7	Present full search strategies, filters, and limits.	Main pp. 3–4; Supplement pp. 2–10 (S1a–S1c)	
8	Describe selection, reviewer independence, and automation.	Main p. 4; screening decision ledger in Zenodo	Reported — Proposed exclusions were reviewed by the four coauthor groups named in the main text; the corresponding author authorized every exclusion.
9	Describe data collection, reviewer independence, investigator contact, and automation.	Main p. 4; Supplement pp. 21–34 (S3–S6)	Reported — Estimate-level extraction and targeted verification are reported.
10a	List and define all outcomes sought and rules for choosing compatible results.	Main pp. 4–5; Supplement pp. 21–45 (S3–S14)	Reported
10b	List and define other variables sought and assumptions for missing or unclear information.	Main pp. 4–5; Supplement pp. 21–45 (S3–S14)	Reported
11	Describe study risk-of-bias methods, tools, assessor independence, and automation.	Main p. 5; Figure 2; Supplement pp. 21–34 (S3–S6)	Reported — Design-matched assessments are shown.
12	Specify effect measures for each outcome.	Main pp. 4–5; Supplement pp. 21–45 (S3–S14)	Reported
13a	Describe how studies were assigned to each synthesis.	Main pp. 4–5; Supplement pp. 21–45 (S3–S14)	Reported
13b	Describe data preparation, transformations, and handling of missing summary statistics.	Main pp. 4–5; Supplement pp. 21–45 (S3–S14)	Reported
13c	Describe tabular and visual display methods.	Main pp. 4–5; Supplement pp. 21–45 (S3–S14)	Reported
13d	Describe synthesis methods, model, heterogeneity estimators, software, and rationale.	Main pp. 4–5; Supplement pp. 21–45 (S3–S14)	Reported
13e	Describe methods to explore heterogeneity.	Main pp. 4–5; Supplement pp. 21–45 (S3–S14)	Reported
13f	Describe sensitivity analyses.	Main pp. 4–5; Supplement pp. 21–45 (S3–S14)	Reported
14	Describe methods to assess risk of bias due to missing results.	Main pp. 4–5; Supplement pp. 21–45 (S3–S14)	Reported
15	Describe certainty-assessment methods.	Main pp. 4–5; Supplement pp. 21–45 (S3–S14)	Reported
16a	Report search and selection results, ideally with a flow diagram.	Main pp. 6–7; Figure 1	
16b	Cite apparently eligible excluded studies and explain exclusions.	Figure 1; screening decision ledger in Zenodo	
17	Cite each included study and present characteristics.	Main p. 8; Supplement pp. 11–20 (S2)	

Abbreviations: AMSTAR-PF, A MeaSurement Tool to Assess systematic Reviews of Prognostic Factor studies; PRISMA, Preferred Reporting Items for Systematic Reviews and Meta-Analyses.

PRISMA 2020 (continued)

Item	Checklist requirement	Where reported	Status / clarification
18	Present risk-of-bias assessments for each included study.	Main p. 9 and Figure 2; Supplement pp. 21–34 (S3–S6)	Reported — Tool-specific judgments are shown for the analytic units to which each instrument applied.
19	Present each study's effect estimate and precision for every outcome.	Main pp. 6–17 and Figures 3–7; Supplement pp. 21–59 (S3–S14, Figures S1–S7)	Reported
20a	Summarize characteristics and risk of bias for studies contributing to each synthesis.	Main pp. 6–17 and Figures 3–7; Supplement pp. 21–59 (S3–S14, Figures S1–S7)	Reported
20b	Present all statistical syntheses, precision, and heterogeneity.	Main pp. 6–17 and Figures 3–7; Supplement pp. 21–59 (S3–S14, Figures S1–S7)	Reported
20c	Present investigations of possible causes of heterogeneity.	Main pp. 6–17 and Figures 3–7; Supplement pp. 21–59 (S3–S14, Figures S1–S7)	Reported
20d	Present sensitivity analyses.	Main pp. 6–17 and Figures 3–7; Supplement pp. 21–59 (S3–S14, Figures S1–S7)	Reported
21	Present reporting-bias assessments for each synthesis assessed.	Main pp. 15 and 19; Supplement p. 43 and Figure S7	
22	Present certainty assessments for each outcome.	Main pp. 6–17, Tables 2–3 and Figure 7; Supplement pp. 44–45 (S14A–S14B)	Reported
23a	Interpret results in the context of other evidence.	Main pp. 17–19	Reported
23b	Discuss limitations of the included evidence.	Main pp. 17–19	Reported
23c	Discuss limitations of review processes.	Main pp. 17–19	Reported
23d	Discuss implications for practice, policy, and research.	Main pp. 17–19	Reported
24a	Provide registration name and number.	Main p. 3; active PROSPERO record	
24b	Indicate where the protocol can be accessed.	Main p. 3; active PROSPERO record	
24c	Describe and explain protocol or registration amendments.	Active PROSPERO record CRD420261435405	Reported — Registration history and the superseded record are described in Methods; amendments are documented in the active PROSPERO record.
25	Describe financial/non-financial support and funder role.	Main p. 20	Reported
26	Declare competing interests.	Main p. 21	Reported
27	State public availability and locations of forms, extracted data, analysis data, code, and materials.	Main p. 20; Zenodo dataset DOI	

Abbreviations: DOI, digital object identifier; PRISMA, Preferred Reporting Items for Systematic Reviews and Meta-Analyses; PROSPERO, International Prospective Register of Systematic Reviews.

PRISMA 2020 for Abstracts

Item	Checklist requirement	Where reported	Status / clarification
1	Identify the report as a systematic review.	Title and Abstract, main p. 1	Reported
2	State an explicit objective or review question.	Abstract—Background, main p. 1	Reported
3	Specify eligibility criteria.	Abstract—Methods, main p. 1	Reported — Condensed for the abstract; full criteria on main pp. 3–4.
4	Specify information sources and last search date.	Abstract—Methods, main p. 1	Reported — Eight sources; final date 25 July 2026.
5	Describe methods used to assess risk of bias.	Abstract—Methods, main p. 1	Reported — Design-matched instruments are named in full on main p. 5.
6	Describe methods used to synthesize results.	Abstract—Methods, main p. 1	Reported — REML random effects with Hartung–Knapp inference.
7	Report the number and type of included studies and participants where available.	Abstract—Results, main p. 1	Reported — Reports, studies and cohort families are distinguished.
8	Report results for the main outcomes, preferably with precision.	Abstract—Results, main p. 1	Reported — Three signals and the inconclusive glomerulosclerosis–DGF result include 95% CIs.
9	Give a brief summary of limitations of the evidence.	Abstract—Results and Conclusions, main p. 1	Reported — All 12 pooled bodies were of very low certainty and no stand-alone acceptance or discard rule is validated.
10	Provide a general interpretation and implications.	Abstract—Conclusions, main p. 1	Reported — No stand-alone acceptance or discard rule is validated.
11	Specify the primary funding source.	Funding statement, main p. 20	
12	Provide the registration name and number.	Registration, main p. 3	

Abbreviations: CI, confidence interval; DGF, delayed graft function; PRISMA, Preferred Reporting Items for Systematic Reviews and Meta-Analyses; REML, restricted maximum likelihood.

PRISMA-S

Item	Checklist requirement	Where reported	Status / clarification
1	Name each database and its platform.	Main pp. 3–4; Supplement pp. 2–10 (S1a–S1c)	
2	State platform and databases when multiple databases were searched simultaneously.	Main pp. 3–4; Supplement pp. 2–10 (S1a–S1c)	
3	List study registries searched.	Main pp. 3–4; Supplement pp. 2–10 (S1a–S1c)	
4	Describe online or print sources purposefully browsed and how.	Main pp. 3–4	
5	Describe cited-reference or citing-reference searching methods.	Main pp. 3–4	
6	Indicate whether authors, experts, manufacturers, or others were contacted.	—	Not separately reported — Investigator contact for additional information is not documented in the final review record.
7	Describe any additional information sources or search methods.	Main pp. 3–4; Supplement pp. 2–10 (S1a–S1c)	
8	Include exact search strategies for every source as run.	Main pp. 3–4; Supplement pp. 2–10 (S1a–S1c)	
9	Report and justify all limits or state none.	Main pp. 3–4; Supplement pp. 2–10 (S1a–S1c)	
10	State whether published search filters were used and cite them.	Main pp. 3–4; Supplement pp. 2–10 (S1a–S1c)	
11	Report adaptation or reuse of prior-review search strategies.	Main pp. 3–4; Supplement pp. 2–10 (S1a–S1c)	
12	Report methods used to update searches.	Main pp. 3–4; Supplement pp. 2–10 (S1a–S1c)	
13	Give the last-search date for every strategy.	Main pp. 3–4; Supplement pp. 2–10 (S1a–S1c)	
14	Describe the search peer-review process.	Main pp. 3–4; Supplement pp. 2–10 (S1a–S1c)	
15	Report records identified from each source.	Main pp. 3–4; Supplement pp. 2–10 (S1a–S1c)	
16	Describe deduplication process and software.	Main pp. 4 and 6; Figure 1	

Abbreviations: PRISMA, Preferred Reporting Items for Systematic Reviews and Meta-Analyses; PRISMA-S, PRISMA extension for reporting literature searches in systematic reviews.

SWiM

Item	Checklist requirement	Where reported	Status / clarification
1a	Describe and justify groups used in synthesis.	Main pp. 4–6, 8 and 15; Figure 6; Supplement pp. 21–29 and 35 (S3, S7)	Reported — The 116 single-cohort cells and 15 two-cohort bodies are kept separate from pooled bodies.
1b	Describe and justify post-protocol changes to synthesis groups.	Main pp. 3–4; active PROSPERO record CRD420261435405	Reported — The synthesis groups followed the prespecified review questions and cohort-family rules.
2	Describe standardized metrics and transformations for each outcome.	Main pp. 4–6, 8 and 15; Supplement pp. 21–29 (S3)	Reported with detail limitation — Effect measures are retained as reported and never combined across scales; 21 contrasts lacked a secure direction.
3	Describe and justify the synthesis method for each outcome when meta-analysis was not possible.	Main pp. 4–6, 8 and 15; Figure 6; Supplement pp. 21–29 and 35 (S3, S7)	Reported — Single-cohort cells are described by direction and precision; two-cohort bodies are displayed without a pooled summary.
4	Give justified criteria used to prioritize studies or results.	Main p. 4; Supplement p. 36 (S8)	
5	State methods used to investigate heterogeneity in non-meta-analysed effects.	Main pp. 5 and 15; Supplement pp. 21–29 and 41–43 (S3, S13)	
6	Describe certainty assessment methods for synthesis findings.	Main pp. 5 and 17; Figure 7; Supplement pp. 44–45 (S14A–S14B)	Reported with applicability limitation — Formal GRADE profiles apply to pooled bodies; unpooled cells retain design-specific risk-of-bias and precision information.
7	Describe graphical/tabular presentation and study ordering.	Main pp. 8 and 15; Figure 6; Supplement pp. 21–29 and 35 (S3, S7)	Reported — Cells are grouped by outcome and lesion, with source-linked estimates and direction.
8	Report every synthesis, its certainty, and the contributing studies.	Main pp. 6, 8 and 15; Figure 6; Supplement pp. 21–29 and 35 (S3, S7)	Reported — Every retained unpooled cell is displayed with its contributing source; 21 non-orientable contrasts remain explicit.
9	Report limitations of synthesis methods/groupings and effect on conclusions.	Main pp. 17–19 ; Supplement pp. 21–29 (S3)	Reported — Direction counts are described as evidence architecture, not as a common effect.

Abbreviations: GRADE, Grading of Recommendations Assessment, Development and Evaluation; PROSPERO, International Prospective Register of Systematic Reviews; SWiM, synthesis without meta-analysis.

TRIPOD-SRMA

Item	Checklist requirement	Where reported	Status / clarification
1	Identify a review of diagnostic/prognostic models and specify target population and predicted outcome.	Main p. 1	Not applicable — This is a four-question review, not a prediction-model review; the title identifies the population, exposure and transplant outcomes.
2	Report the TRIPOD-SRMA abstract items.	Abstract, main p. 1	Not applicable — The review is not a prediction-model review; the abstract follows PRISMA for Abstracts (model-phase detail in Table S4, p. 30).
3	Describe the rationale in context.	Main pp. 2–3	Reported — Prediction is distinguished from association and decision utility.
4	State objectives with target population, models, outcomes, prediction horizon/moment, and setting.	Main pp. 2–3	Not applicable — Population, setting, intended use and outcomes are defined; model-specific horizons apply only to the embedded prediction domain.
5	Specify prediction-model eligibility and development/validation eligibility.	Main p. 3	Reported — Development, validation and incremental-value studies were eligible.
6	Specify all sources and last search dates.	Main pp. 3–4; Supplement pp. 2–10 (S1a–S1c)	Reported — All sources and the final search date are given.
7	Present full search strategies, filters, and limits.	Main pp. 3–4; Supplement pp. 2–10 (S1a–S1c)	Reported — The prediction search stream and database translations are reproduced.
8	Describe model-study selection, reviewers, independence, and automation.	Main p. 4; screening decision ledger in Zenodo	Reported — Selection and automation are described; the four coauthor groups named in the main text reviewed all proposed exclusions under corresponding-author authority.
9	Describe data collection, reviewer independence, investigator contact, and automation.	Main p. 4; Supplement p. 30 (S4)	Reported — Items sought and verification routes are stated; data collection was divided among the four coauthor groups named in the main text, with final authorization by the corresponding author.
10a	List and define all model/study data items sought.	Main p. 4; Supplement p. 30 (S4)	Reported — Study, model, validation-phase, performance, applicability and clinical-utility fields are displayed.
10b	State model-performance measures sought.	Main pp. 4–5 and 8; Supplement p. 30 (S4)	Reported — Discrimination, calibration, overall performance and decision-utility measures are retained where reported.
10c	Describe handling of desired but unreported model data.	Supplement p. 30 (S4)	Reported with data-availability limitation — Unreported phase-specific performance is labeled 'Not reported'; no imputation or repair was performed.
11	Describe risk of bias/applicability methods separately for development and validation.	Main p. 5; Figure 2; Supplement p. 30 (S4)	Reported — CHARMS and PROBAST+AI were used by model phase.
12a	Describe methods for synthesizing performance of each model.	Main p. 15; Supplement p. 30 (S4)	Not applicable — The four extractable reports evaluated different models/phases; no model-performance meta-analysis was conducted.
12b	Describe methods to explore heterogeneity in model performance.	Main p. 15; Supplement p. 30 (S4)	Not applicable — Sparsity and non-comparability precluded a model-performance heterogeneity analysis.
12c	Describe prediction-model sensitivity analyses.	Main p. 15; Supplement p. 30 (S4)	Not applicable — No compatible prediction-model synthesis existed for sensitivity analysis.
13	Describe certainty/confidence assessment for each prediction model.	Main p. 5; Figure 2; Supplement p. 30 (S4)	Reported with framework limitation — Validation phase, risk of bias and applicability are reported; no separate model-performance certainty framework was imposed.
14	Report selection from records to studies and models, ideally with a diagram.	Main pp. 6 and 8; Figure 1; Supplement p. 30 (S4)	
15	Present study characteristics and model details and cite reports.	Main pp. 8 and 15; Supplement pp. 11–20 and 30 (S2, S4)	
16	Present risk of bias/applicability separately by development and validation.	Main pp. 9 and 15; Figure 2; Supplement p. 30 (S4)	Reported — Risk of bias and applicability are shown for every extractable model phase.
17	Present model performance estimates and confidence intervals for every evaluation and validation type.	Main p. 15; Supplement p. 30 (S4)	Reported — All reported phase-specific performance estimates are displayed; missing measures remain explicitly unreported.
18a	Present synthesis of model performance and contributing estimates.	Main p. 15; Supplement p. 30 (S4)	Not applicable — No compatible model-performance synthesis was conducted.
18b	Present investigations of heterogeneity in model performance.	Main p. 15; Supplement p. 30 (S4)	Not applicable — No compatible model-performance heterogeneity analysis was possible.
18c	Present prediction-model sensitivity analyses.	Main p. 15; Supplement p. 30 (S4)	Not applicable — No compatible prediction-model sensitivity synthesis was possible.
19	Present certainty/confidence for each model of interest.	Main pp. 5, 9 and 15; Figure 2; Supplement p. 30 (S4)	Reported with framework limitation — Model phase, risk of bias and applicability are reported; no separate certainty rating was assigned to each model.

Abbreviations: CHARMS, Critical Appraisal and Data Extraction for Systematic Reviews of Prediction Modelling Studies; PRISMA, Preferred Reporting Items for Systematic Reviews and Meta-Analyses; PROBAST+AI, Prediction model Risk Of Bias ASsessment Tool plus
Artificial Intelligence; TRIPOD-SRMA, Transparent Reporting of multivariable prediction models for Individual Prognosis Or Diagnosis in Systematic Reviews and Meta-Analyses.

TRIPOD-SRMA (continued)

Item	Checklist requirement	Where reported	Status / clarification
20	Summarize main prediction findings and evidence limitations.	Main pp. 17–19 ; Supplement p. 30 (S4)	Reported — Main findings and evidence limitations are interpreted together.
21	Discuss strengths and limitations of the review process.	Main pp. 17–19	Reported — Review-process and evidence limitations are stated.
22	Discuss implications for evidence, practice, policy, and research.	Main pp. 17–19	Reported — Implications for practice, policy and research are separated.
23a	Provide registration name and number.	Main p. 3	
23b	Indicate where the protocol can be accessed.	Main p. 3; PROSPERO CRD420261435405	Reported — The active registration number identifies the public protocol record.
23c	Describe and explain amendments.	Active PROSPERO record CRD420261435405	Reported in registry — Protocol amendments are documented in the active record.
24	Describe financial/non-financial support and funder role.	Main p. 20	
25	Declare competing interests.	Main p. 21	Reported — No commercial or financial conflicts were declared.
26	Report public availability of forms, extracted data, analysis data, code, and materials.	Main p. 20; Zenodo dataset DOI	

Abbreviations: DOI, digital object identifier; PROSPERO, International Prospective Register of Systematic Reviews; TRIPOD-SRMA, Transparent Reporting of multivariable prediction models for Individual Prognosis Or Diagnosis in Systematic Reviews and Meta-Analyses.

AMSTAR-PF self-audit

Item	Checklist requirement	Where reported	Status / clarification
1	Clearly define the research question and PICOTS.	Main pp. 2–3	Reported — Self-audit only; not an external endorsement. PICOTS and the four distinct review questions are defined.
2a	State a publicly available protocol registered before conduct.	Main p. 3; PROSPERO CRD420261435405	Reported — Self-audit only; not an external endorsement. The active record preceded execution of the final search.
2b	Justify deviations from the protocol.	Main pp. 3–4; PROSPERO CRD420261435405	Reported — Self-audit only; not an external endorsement. The active registration and implemented methods are identified.
3	Report eligible prognostic-factor study types/designs.	Main p. 3	Reported — Self-audit only; not an external endorsement. Eligible designs are specified separately for association, prediction, utility and measurement.
4	Use a comprehensive search strategy.	Main pp. 3–4; Supplement pp. 2–10 (S1a–S1c)	Reported — Self-audit only; not an external endorsement. The database/registry search, PRESS review, and backward and forward citation searching are described.
5	Use a rigorous inclusion-evaluation process.	Main p. 4; screening decision ledger in Zenodo	Reported — Self-audit only; not an external endorsement. Proposed exclusions were reviewed by the four coauthor groups named in the main text and authorized by the corresponding author.
6	List full-text exclusions and reasons.	Figure 1; screening decision ledger in Zenodo	Reported — Self-audit only; not an external endorsement. Full-text exclusions and reasons are retained.
7a	Use a rigorous data-extraction process.	Main p. 4; Supplement pp. 21–45 (S3–S14)	Reported — Self-audit only; not an external endorsement. Estimate-level extraction and targeted verification are documented.
7b	Describe included studies in adequate detail.	Main p. 8; Supplement pp. 11–34 (S2–S6)	
7c	Use appropriate methods to obtain prognostic-factor effects and precision.	Main pp. 4–5; Supplement pp. 21–45 (S3–S14)	Reported — Self-audit only; not an external endorsement. Effect derivation, variance routes, selection and precision are specified.
8a	Use a rigorous risk-of-bias assessment process.	Main pp. 5 and 9; Figure 2; Supplement pp. 21–34 (S3–S6)	Reported — Self-audit only; not an external endorsement. Design-matched appraisal is complete for analytic units.
8b	Use an appropriate risk-of-bias technique.	Main p. 5; Figure 2; Supplement pp. 21–34 (S3–S6)	Reported — Self-audit only; not an external endorsement. QUIPS was used for prognostic-factor evidence, with design-specific tools for the other domains.
9a	Use an interpretable synthesis approach.	Main pp. 4–5; Supplement pp. 35–45 (S7–S14)	Reported — Self-audit only; not an external endorsement. Compatibility, one-cohort selection and non-pooled routes are explicit.
9b	Use appropriate meta-analysis methods.	Main p. 5; Figures 3–5; Supplement pp. 36–45 (S8–S14)	
10	Adequately investigate small-study effects when quantitative synthesis permits.	Main pp. 5 and 15; Supplement p. 43 and Figure S7	Assessed per analysis; no pooled body reached the ten-cohort threshold for formal tests (main pp. 15 and 19).
11	Account for study risk of bias in interpretation.	Main pp. 9 and 17–19 ; Tables 2–3; Figure 7	Reported — Self-audit only; not an external endorsement. Risk of bias constrains every clinical interpretation.
12	Discuss observed heterogeneity.	Main pp. 9–19 ; Figures 3–5; Supplement pp. 40–43 (S12–S13)	Reported — Self-audit only; not an external endorsement. Statistical and clinical heterogeneity are described separately.
13	Report conflicts and funding for review authors and included studies.	Main pp. 20–21	Reported — Self-audit only; not an external endorsement. Review-team funding and competing interests are reported; included-study funding/conflicts were not systematically tabulated.
14	Assess certainty for key prognostic findings with appropriate methods.	Main pp. 5 and 17; Figure 7; Supplement pp. 44–45 (S14A–S14B)	Reported — Self-audit only; not an external endorsement. GRADE adapted for prognostic factors is applied to every pooled body.

Abbreviations: AMSTAR-PF, A MeaSurement Tool to Assess systematic Reviews of Prognostic Factor studies; GRADE, Grading of Recommendations Assessment, Development and Evaluation; PICOTS, population, index prognostic factor, comparator, outcomes, timing and setting; PRESS, Peer Review of Electronic Search Strategies; PROSPERO, International Prospective Register of Systematic Reviews; QUIPS, Quality In Prognosis Studies.
